# High-efficiency purification of divergent AAV serotypes using AAVX affinity chromatography

**DOI:** 10.1016/j.omtm.2022.12.009

**Published:** 2022-12-16

**Authors:** Michael Florea, Fotini Nicolaou, Simon Pacouret, Eric M. Zinn, Julio Sanmiguel, Eva Andres-Mateos, Carmen Unzu, Amy J. Wagers, Luk H. Vandenberghe

**Affiliations:** 1 Grousbeck Gene Therapy Center, Schepens Eye Research Institute and Massachusetts Eye and Ear Infirmary, Boston, MA, USA; 2 Harvard Ph.D. Program in Biological and Biomedical Sciences, Division of Medical Sciences, Harvard University, Boston, MA, USA; 3 Department of Stem Cell and Regenerative Biology, Harvard University, Cambridge, MA, USA; 4 Ocular Genomics Institute, Department of Ophthalmology, Harvard Medical School, Boston, MA, USA; 5 The Broad Institute of Harvard and MIT, Boston, MA, USA; 6 Harvard Stem Cell Institute, Harvard University, Cambridge, MA, USA; 7 Paul F. Glenn Center for the Biology of Aging, Harvard Medical School, Boston, MA, USA; 8 Section on Islet Cell and Regenerative Biology, Joslin Diabetes Center, Boston, MA, USA

**Keywords:** AAV, gene therapy, adeno-associated, purification, manufacturing, downstream, affinity chromatography, AAVX

## Abstract

The adeno-associated viral vector (AAV) provides a safe and efficient gene therapy platform with several approved products that have marked therapeutic impact for patients. However, a major bottleneck in the development and commercialization of AAV remains the efficiency, cost, and scalability of AAV production. Chromatographic methods have the potential to allow purification at increased scales and lower cost but often require optimization specific to each serotype. Here, we demonstrate that the POROS CaptureSelect AAVX affinity resin efficiently captures a panel of 15 divergent AAV serotypes, including the commonly used AAV2, AAV8, AAV9, PHP.B, and Anc80. We also find that AAVX resin can be regenerated repeatedly without loss of efficiency or carry-over contamination. While AAV preps purified with AAVX showed a higher fraction of empty capsids than preps purified using iodixanol ultracentrifugation, the potency of the AAVX purified vectors was comparable with that of iodixanol purified vectors both *in vitro* and *in vivo*. Finally, optimization of the purification protocol resulted in a process with an overall efficiency of 65%–80% across all scales and AAV serotypes tested. These data establish AAVX affinity chromatography as a versatile and efficient method for purification of a broad range of AAV serotypes.

## Graphical abstract

**Figure:**
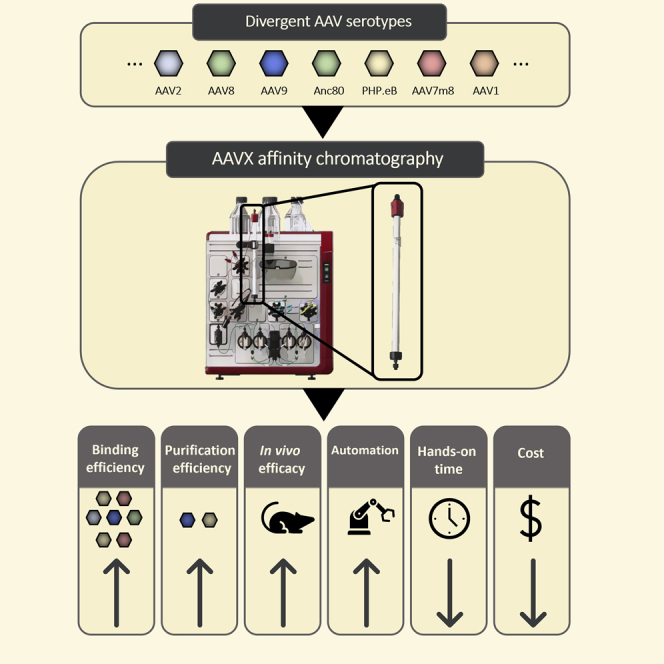

## Teaser

Florea et al. describe a versatile AAV purification method using POROS CaptureSelect AAVX affinity chromatography applicable to a large set of divergent AAV serotypes. The method is cost effective and yields high-quality AAV at laboratory scale. A detailed protocol is provided to enable ready adoption in research laboratories interested in AAV research.

## Introduction

Adeno-associated viruses (AAVs) are small, non-enveloped single-stranded DNA viruses discovered in the 1960s as contaminants of adenovirus preparations.^1^^,^^2^ They induce limited host immune response and are not associated with any known disease, yet were found to be highly efficient at delivering DNA cargo to many tissues in multiple animal species.^3^ AAV vectors are thus widely used as a gene transfer tool in basic research and in translational and clinical gene therapy.^4^ Their higher use has increased demand for AAV manufacturing both in terms of the quality of the preparation and the quantity of the material.

Currently, for research and for some clinical purposes, the commonly used AAV purification method uses ultracentrifugation of the sample on a cesium chloride (CsCl) or iodixanol density gradient.^5^^,^^6^^,^^7^ This process is appealing for two reasons: first, it is serotype agnostic with little process optimization needed for the various AAV products researchers seek to purify; and second, it remains one of the more efficient methods of separation of genome-containing (or “full”) capsids from empty or partially filled capsids. However, ultracentrifugation is a manual multi-step processes (sample concentration, preparation of density gradient, sample loading, centrifugation, and aspiration of the target layer). This makes it labor intensive and difficult to scale, and adds a requirement for precise handling.^8^ Finally, ultracentrifugation may co-purify contaminants that have the same sedimentation coefficient as AAV.^8^

Liquid chromatography provides a more scalable, less laborious, and possibly more efficient purification method, particularly under high-performance liquid chromatography (HPLC) conditions, as has been shown for the purification of proteins and small molecules.^9^ For AAV, several chromatographic methods have been developed, most using AVB Sepharose affinity, cation exchange, or anion exchange chromatography.^10^^,^^11^^,^^12^^,^^13^ While these methods demonstrate the feasibility and efficiency of AAV chromatographic purification, they also require substantial serotype-specific optimization. Thus, they are not optimal for AAV purification in a research setting, where different serotypes need to be purified for different applications in a flexible process.

Recently, several AAV binding resins have been commercially released, including AVB resin (AVB Sepharose High Performance; GE Healthcare, Chicago, IL, USA) and POROS CaptureSelect AAV8, AAV9, and AAVX resins (Thermo Fisher Scientific, Waltham, MA, USA). In the case of AVB resin, it was shown that affinity chromatography using AVB can efficiently purify AAV1, AAV2, AAV5, AAV6, and rh10, but requires serotype-specific optimization and does not bind to multiple other serotypes, including AAV8 and AAV9.^13^^,^^14^ POROS CaptureSelect AAV8 and AAV9 resins bind and were specifically developed for purification of AAV8 and AAV9, respectively, but not other serotypes (POROS CaptureSelect product datasheet).^11^^,^^13^ POROS CaptureSelect AAVX is a 50-μm resin consisting of a rigid crosslinked poly(styrene divinylbenzene) bead backbone, coated with crosslinked polyhydroxylated polymer, and linked to a camelid heavy-chain-only single-domain antibody fragment. The camelid antibody was raised against a conserved region of the AAV capsid, and the AAVX resin is marketed as a pan-AAV affinity resin capable of binding multiple different AAV serotypes (POROS CaptureSelect product datasheet).^11^ However, to date there are no independently generated published data on assessing the performance of AAVX. For this reason, we sought to evaluate the AAVX resin for its ability to bind various AAV serotypes and its utility to be incorporated into a fully integrated AAV purification process.

## Results

### AAVX binds several AAV serotypes

**Figure 1:**
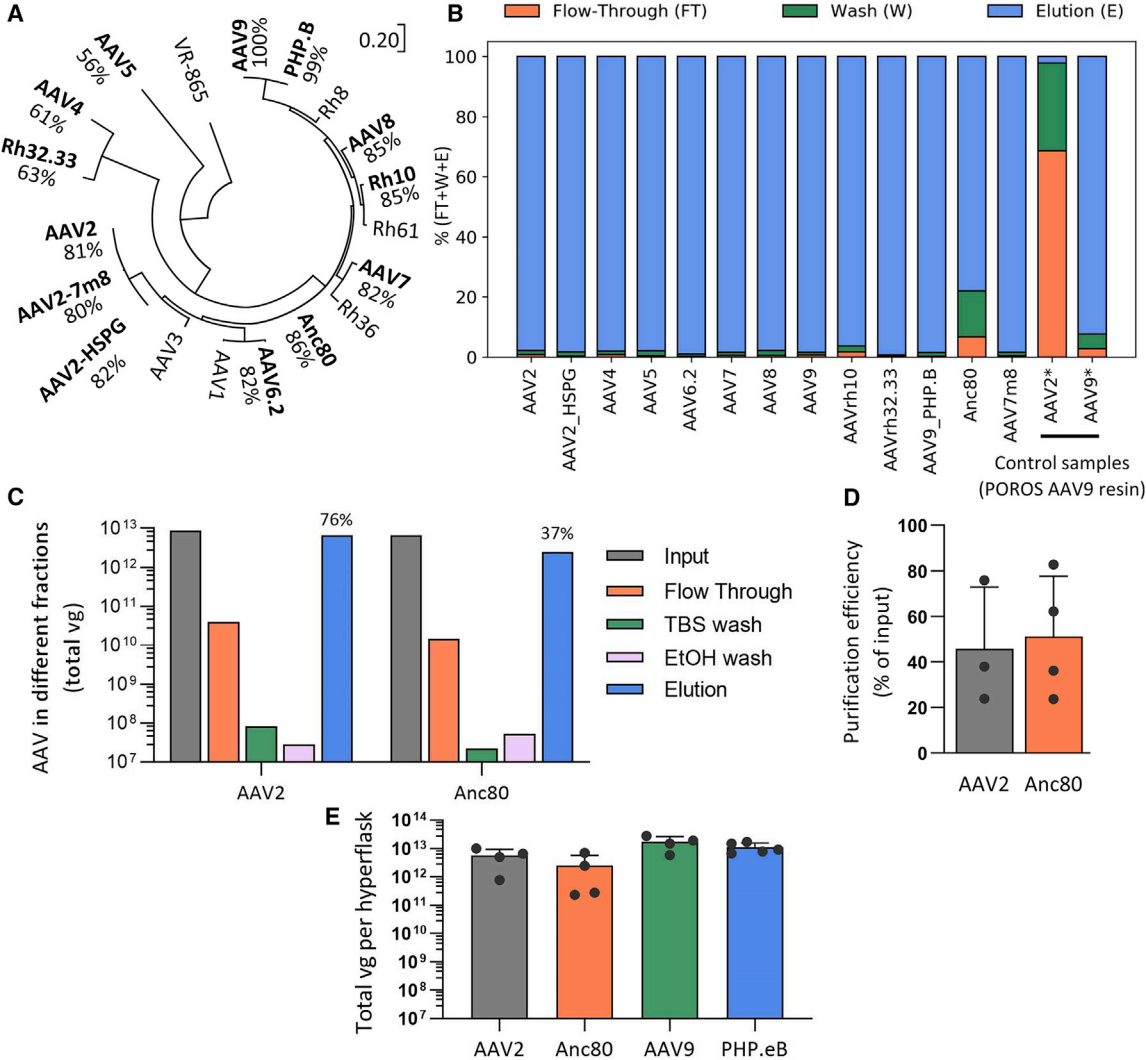
AAV purification using AAVX affinity chromatography (A) Phylogeny depicting the diversity of AAV capsids included in this report (bold) along with the percent identity (by amino acid) compared with AAV9. The tree is drawn to scale with branch lengths depicting substitutions per site. VR-865 is an avian AAV used as an outgroup. (B) Affinity of AAVX to various AAV serotypes tested in a static binding assay. The flow-through (FT), wash (W), and eluted fractions (E) were collected and analyzed by qPCR to quantify their vector genome copies. Data represented as percent vector genomes (vg) of the input. Each serotype was applied to unused AAVX resin. (C) AAV purification of AAV2 and Anc80 using AAVX resin in an HPLC setting. Fractions were taken from input, flow-through, at Tris-buffered saline (TBS) and ethanol wash steps and at elution, and AAV content was quantified using qPCR. Percent recovery for these purifications is shown above elution bars. N = 1 each. (D) Average purification efficiencies of AAV2 and Anc80 (percent recovery of AAV in the elution). (E) Total yields of purified AAV2, Anc80, AAV9, and PHP.eB preps with no optimization of the process. Each dot represents an AAV prep from one HYPERFlask (1,720 cm^2^ growth area). Error bars denote standard deviation. All purifications were carried out at room temperature, using 1-mL AAVX column at 1 mL/min flow rate. All values estimated are above qPCR limit of detection (approximately 10^5^ vg/mL).

We first sought to test, on a small scale, which serotypes bind to the POROS CaptureSelect AAVX resin (subsequently denoted as AAVX). To this end, the phylogenetically diverse AAV serotypes AAV2, AAV2_HSPG, AAV4, AAV5, AAV6.2, AAV7, AAV8, AAV9, rh10, rh32.33, PHP.B, Anc80, and AAV7m8^1^^,^^15^^,^^16^^,^^17^^,^^18^^,^^19^^,^^20^^,^^21^^,^^22^^,^^23^ (Figure 1A) were produced at small scale, purified via ultracentrifugation on an iodixanol gradient, and applied to the AAVX resin bed in a static binding assay. After incubation, resin was washed with PBS, AAV was eluted using 0.1 M citric acid and quantified in different fractions using qPCR (Figure 1B). The result of this binding assay demonstrates that AAVX binds all of the tested serotypes with relatively high efficiency, similarly to the positive control of AAV9 incubated with the POROS AAV9 resin. Recovery was >95% for all serotypes tested except for Anc80, which showed around 80% recovery. On the other hand, the control sample of AAV2 incubated with the POROS AAV9 resin showed poor (<5%) binding efficiency (Figure 1B). This suggests that the AAVX resin has broad affinity and may significantly improve the purification process for divergent serotypes.

### AAVX affinity chromatography can be used to purify AAV

Next, we aimed to determine whether AAV vectors could be purified with the AAVX resin via HPLC, choosing the Corning HYPERFlask (560 mL harvest volume, 1,720 cm^2^ surface area) as the process development vessel for scale-up production. We chose AAV2 and Anc80 because of their high sequence divergence and broad research and clinical utility, and for both serotypes we purified preparations from a single HYPERFlask using AAVX-HPLC. In short, the production and purification process consisted of triple transfection of adherent HEK293 cells, cell pellet and medium harvest and high salt lysis 3 days post transfection, benzonase treatment, clarification of lysate by centrifugation and filtration, and AAVX affinity chromatographic purification at room temperature with immediate neutralization of the eluted vector. The vector was then sterilized through 0.22-μm filtration and buffer-exchanged final buffer exchanges, and concentrated using a 50 kDa molecular weight cutoff filtration unit (Amicon Ultra-15). Recovery in each of the different chromatography fractions (Figure S1) was quantified by qPCR for DNAse-resistant vector genomes (Figure 1C). Results from these experiments indicated that the majority of input vector was found in the elution fraction, with only a minor fraction of vector lost in the flow-through or Tris-buffered saline (TBS) and ethanol fractions. Additional preps indicated that combined average purification efficiency for both AAV2 and Anc80 without serotype-specific optimization was around 50% (Figure 1D). The average yield of AAV2 and Anc80 from this initial process was 10^13^ vector genomes (vg) of AAV per HYPERFlask, which was maintained for the serotypes AAV9 and PHP.eB (Figure 1E).

### AAVX can be regenerated for re-use without loss of efficiency or carry-over contamination

**Figure 2:**
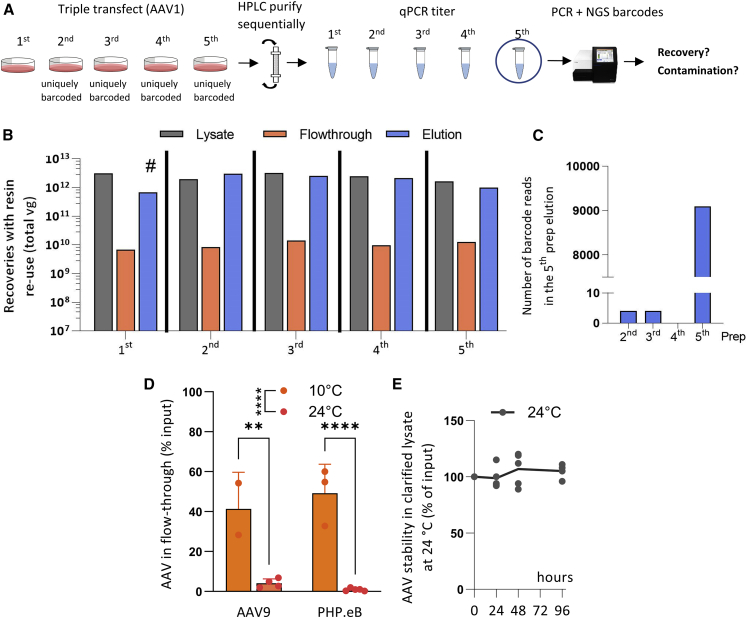
Effect of resin regeneration and temperature on purification efficiency (A) Overview of experimental design of figures (B) and (C). Five small-scale AAV1 preps were produced and purified sequentially on HPLC with AAVX resin without changing the resin between purifications. One prep contained AAV from one and a half 15-cm dishes. Preps 2–5 were identical except for a 100-bp barcode region. Vector genomes were quantified across all purifications. For the fifth prep, the barcode region was PCR amplified and next-generation sequenced, and the unique barcodes corresponding to each prep were quantified to estimate carry-over contamination from preps 2–4. AAV was applied to a column packed with 1 mL of AAVX resin at 1 mL/min flow rate at room temperature. (B) Purification efficiency with repeated resin use. Vector genomes in lysate, flow-through, and elution. Hash mark indicates that some of the sample was lost due to handling error. (C) Estimation of carry-over contamination. Barcode counts from preps 2–5, in the fifth prep estimated via next-generation sequencing. (D) Effect of purification temperature on the percentage of vector genomes found in flow-through for AAV9 and PHP.eB. Difference was assessed using two-way ANOVA with Šídák’s post hoc tests. (E) Stability of AAV (PHP.eB) in clarified lysate at 24°C over 96 h. ∗∗p < 0.01, ∗∗∗p < 0.001, ∗∗∗∗p < 0.0001. Error bars denote standard deviation.

Next, we aimed to determine whether HPLC purification of AAV with AAVX also functions at small scale and whether resin can be re-used multiple times without contamination or loss of efficiency. Re-using resin is of interest because it decreases the cost and labor associated with AAV purification and allows automatic back-to-back purification of multiple preparations. We produced five different AAV1 preps at small scale (from one and a half 15-cm dishes per prep), whereby the vectors of the second to fifth AAV prep were identical except for a unique 100-bp DNA barcode region (Figure 2A). We purified the preps consecutively from prep 1 to prep 5 using the same bed of resin. The resin was regenerated using 6 M guanidine and equilibrated with TBS and 20% ethanol washes between each run. We then quantified the vector genomes in the input lysate, the flow-through, and final elution via qPCR (Figure 2B). Throughout the experiment most of the input vector was found in the elution fraction (<2% found in flow-through), and there was no detectable loss of purification efficiency. Furthermore, next-generation sequencing of the barcode region in the fifth prep showed that the majority (99.93%) of genomes found in the elution fraction came from the correct fifth prep, not preps 2–4 (Figure 2C). These results indicate that resin can be re-used multiple times without considerable loss of efficiency or carry-over contamination. We observed similar results with different batches of AAVX resin in other experiments (see Figures S2 and S3), indicating low AAVX batch-to-batch variability.

Using the same method as described above, we also asked whether the addition of Pluronic F-68 to HPLC buffers increases purification efficiencies. Pluronic F-68 is a non-ionic surfactant that has been shown to decrease AAV non-specific binding to various surfaces including plasticware.^24^^,^^25^ As HPLC contains long and narrow plastic tubing, we reasoned that addition of Pluronic F-68 may increase purification efficiency by reducing AAV binding to plastic. To test this hypothesis, we added Pluronic F-68 to HPLC buffers to the concentration of 0.1% v/v and repeated the experiment described in Figure 2A (Figure S2A). The results indicate that Pluronic F-68 did not increase elution efficiencies for AAV1. However, it showed a trend toward increased efficiencies at the post-elution purification steps (Figures S2B and S2C) and did not increase carry-over contamination (Figures S2D and S2E). This indicates that Pluronic F-68 is a safe addition to HPLC buffers and may be considered for serotypes that are known to be strongly affected by binding to plastic.

### Purification efficiency is temperature dependent

A major challenge in AAV manufacturing during the downstream process is to prepare stable viral vectors, preventing degradation and maintaining production efficiency in a reproducible and cost-effective way. For this reason, HPLC machines are commonly housed and operated at low ambient temperatures (4°C or 10°C) to improve protein stability. As such, we evaluated the role of temperature on purification efficiency during AAV9 and PHP.eB purification. We found that a substantial proportion of vector (40%–50%) was lost in flow-through at 10°C whereas viral losses accounted for less than 5% at 24°C (Figure 2D). However, viral vector degradation due to external factors such as high ambient temperature can adversely impact stability and transduction efficiency of the viral product. We therefore tested thermal stability of clarified viral harvests during the downstream process by keeping AAV input virus at 24°C and assessing vector degradation using qPCR quantification over 4 days. The results indicate that AAV titers were stable over the 4-day timeline (Figure 2E). Overall, these results indicate that purification at ambient (24°C) temperatures reduces viral loss during purification and that AAV remains stable at these temperatures during the timeline of the purification process.

### An optimized purification protocol

**Figure 3:**
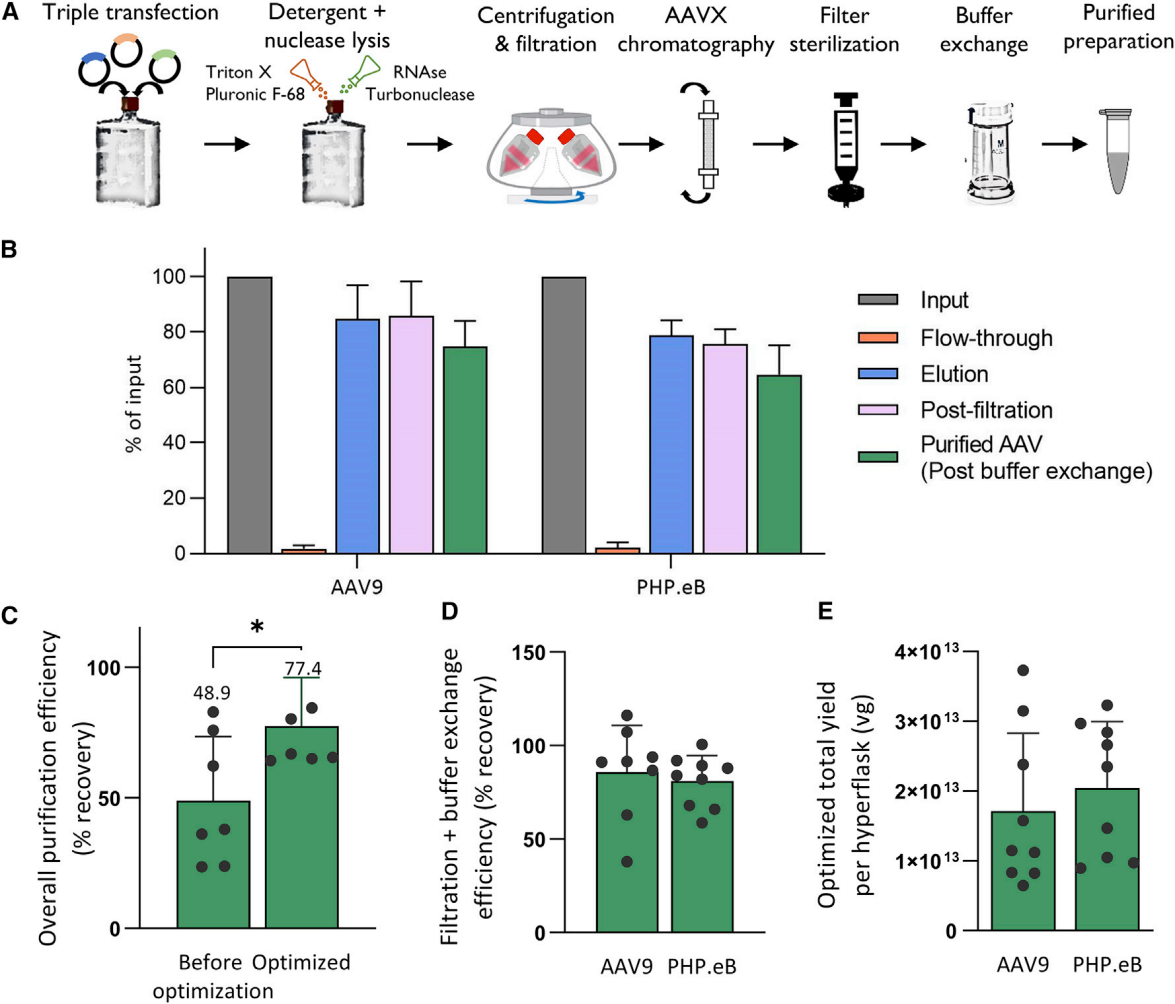
Optimized AAVX affinity purification process (A) Process steps of the protocol. (B) Stepwise recovery at each step of the purification process. Vector genomes were quantified via qPCR from aliquots of the sample at each process step and represented as normalized to the lysate. N = 6 biological replicates for both AAV9 and PHP.eB. (C) Overall purification efficiencies of the non-optimized and optimized protocols for AAV9 and PHP.eB combined. Difference was assessed using a two-tailed t test, with ∗p < 0.05. (D) Recovery after filtration + buffer exchange steps for AAV9 and PHP.eB. Note that the values above 100% fall within the range of the approximately 20% precision limit of qPCR titration, and likely do not represent actual recoveries above 100%. (E) Total yields per HYPERFlask across all vectors produced with scAAV9 and scPHP.eB and purified using this protocol. Error bars denote standard deviation in all panels. Note that this includes some vectors that have lower than average production yields. Detailed steps of the purification process are listed in supplemental protocol.

AAVX affinity purification can be utilized for a variety of viral vectors; however, optimization of the various workflow steps will enable more cost-effective, high-yield, and reproducible production. We therefore performed a granular downstream optimization process for production of a larger scale (up to 10^14^ vg) of AAV. Major challenges in the workflow included efficient lysis and the design of filtration and final formulation steps that minimize AAV loss. This optimization resulted in a process with the following components (see supplemental protocol for process details):

1. *In situ cell lysis using detergents and nucleases*. Based on the protocol described by Florencio et al. ^26^ and our own observations, *in situ* lysis using detergents and nucleases is as efficient as separate lysis of the cell pellet and may be more efficient than *in situ* lysis using hypertonic salt. To obtain one-step lysis and DNA/RNA removal, we added RNase A (4.4 μg/mL), Turbonuclease (2.5 U/mL), Triton X-(0.5% v/v), and Pluronic F-68 (0.001% v/v) to the HYPERFlask and incubated for 1 h at 37°C with orbital shaking at 150 rpm to aid lysis with mechanical forces (see supplemental protocol for details). Here, Triton X-100 and RNase A act as primary lysis agents, Turbonuclease acts to degrade plasmid and cell DNA, and Pluronic F-68 serves to decrease potential AAV binding to plastics.
2. *Addition of Pluronic F-68 to all buffers*. Based on our observation that the addition of Pluronic F-68 does not reduce HPLC purification efficiencies (Figure S2), and based on multiple anecdotal sources indicating that the coating of plastic and/or filter surfaces with surfactants may reduce protein binding, we added Pluronic F-68 at 0.01% v/v to the elution buffer and incubated all plasticware that came into contact with AAV with a Pluronic F-68 containing solution (Final Formulation Buffer [FFB]: 1× PBS, 172 mM NaCl, 0.001% Pluronic F-68) for approximately 15 min at room temperature. Additionally, pipette tips and serological pipettes are similarly coated with FFB prior to handling AAV.
3. *Stringent resin cleaning with 0*.*1 M phosphoric acid and 6 M guanidine*. While we observed no loss in AAV binding efficiencies with resin re-use at small scales with AAV1 (Figures 2 and S2), we did observe some loss of binding efficiencies with re-use at large scales, particularly for PHP.eB (data not shown). Based on the recommendations of the AAVX manufacturers (A. Becerra, Thermo Fisher Scientific, personal communication), we increased resin-cleaning stringency from a 5-min wash with 6 M guanidine alone to a 15-min wash with 0.1 M phosphoric acid (pH 1), followed by 15-min wash with 6 M guanidine-HCl. These changes restored efficient resin binding to up to at least six resin regenerations for both AAV9 and PHP.eB (Figures S3A and S3B) with no significant AAV losses in flow-through (Figure S3C).
4. *Improvement of buffer exchange*. Our analysis indicated substantial losses at the buffer exchange step (25%–50%; data not shown). This can be caused by AAV binding to plastic/filter surfaces, shear stress, or overconcentration on the filter surface during buffer exchange, leading to aggregation, sedimentation, and/or loss of functionality of AAV. To mitigate loss of AAV due to binding, we pre-treated all filters/plasticware with 0.001% Pluronic F-68 as described above. To reduce vector loss due to overconcentration and precipitation, we switched to Amicon Stirred Cell concentrators, which allow for use of higher volumes and continuous mixing during concentration, reducing aggregation and sedimentation. Alternatively, we used Amicon Ultra-15 filter concentrators with frequent (every 2 min of centrifugation) mixing and washing of the filter and did not exceed a total of approximately 2 × 10^13^ vg of AAV per one concentrator. The resulting process is summarized in Figure 3A qPCR analysis of the amount of AAV found in different fractions of the optimized process indicate high recovery efficiencies at every step, with an overall average purification efficiency of approximately 80% for AAV9 and approximately 65% for PHP.eB (Figure 3B), with a combined overall purification efficiency of approximately 75% (Figure 3C). This is driven by a considerable increase in efficiency at the filter sterilization and buffer exchange steps compared with the non-optimized protocol (Figure 3D). Using the optimized protocol, we obtained an average yield of 2 × 10^13^ vg per HYPERFlask across multiple vectors packaged with different transgenes, albeit this analysis also includes some vectors with transgenes that lead to lower than average production yields (Figure 3E). After all of the aforementioned modifications to the process were introduced, analysis of AAV loss at each step indicated that less than 5% of AAV is lost in the flow-through or at the filter sterilization steps, while 10% and 20% on average are lost at the buffer exchange and elution steps, respectively (Figure S4), indicating potential targets for future optimization.

### The yield and bioactivity of AAVX-HPLC purified AAV are comparable with those of iodixanol purified AAV

**Figure 4:**
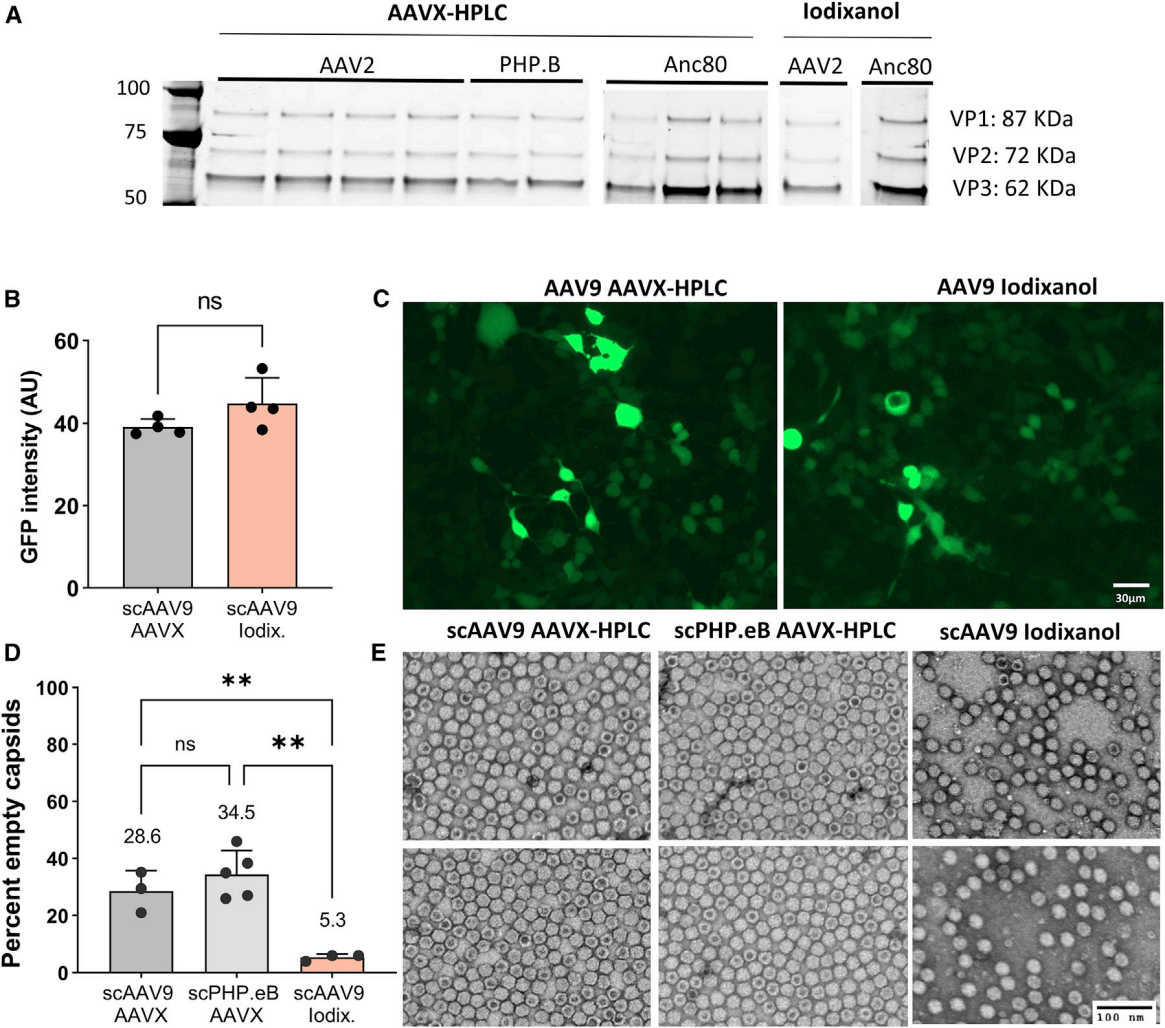
Quality and *in vitro* bioactivity of AAVX affinity-purified AAV (A) SYPRO Ruby-stained protein gel analysis of AAVX-HPLC versus iodixanol ultracentrifugation purified vectors. Most preps show clear, distinct VP1–VP3 bands, with few non-specific bands present, indicating comparable purity with iodixonal purified virus. (B) Quantification empty capsid content using negative stained TEM. Approximately N = 200 particles were counted for each prep from two separate images by two blinded researchers. Statistical significance was assessed using one-way ANOVA with follow-on Tukey’s multiple comparisons test. (C) Representative micrographs of AAVX and iodixanol purified preps used to perform the quantification, with two representative images shown for each. (D) *In vitro* infectivity of AAVX and iodixanol ultracentrifugation purified scAAV9 preps on HEK293 cells. Statistical significance was assessed using two-tailed t test. (E) Representative images used to perform the quantification in (D). ∗∗p < 0.01; ns (not significant), p > 0.05. Error bars denote standard deviation in all panels. See also Figures S5–S7 for full images of SYPRO Ruby gels, GFP micrographs, and TEM micrographs, respectively.

To determine whether HPLC purified virus is qualitatively and quantitatively comparable with that of iodixanol ultracentrifugation purified vectors, we compared HPLC purified vectors and iodixanol purified vectors with regard to purity, empty capsid content, *in vitro* bioactivity, and *in vivo* bioactivity. Analysis by gel electrophoresis and SYPRO Ruby red staining indicates that HPLC purified preps are comparable with iodixanol purified preps and consist mainly of the expected VP1–VP3 bands, with little to no unspecific bands present (Figures 4A and S5). Negative-stain transmission electron microscopy (TEM) analysis of the HPLC purified preps indicates an average of approximately 30% empty capsids, which was higher than the approximately 5% empty capsids observed in iodixanol ultracentrifugation purified preps (Figures 4B, 4C, and S7). However, *in vitro* infectivity assay of HEK293 cells indicated that HPLC and iodixanol purified vectors were equally efficient at transducing cells *in vitro*, suggesting that the higher percentage of empty capsids did not have a functional effect on bioactivity (Figures 4D, 4E, and S6).

**Figure 5:**
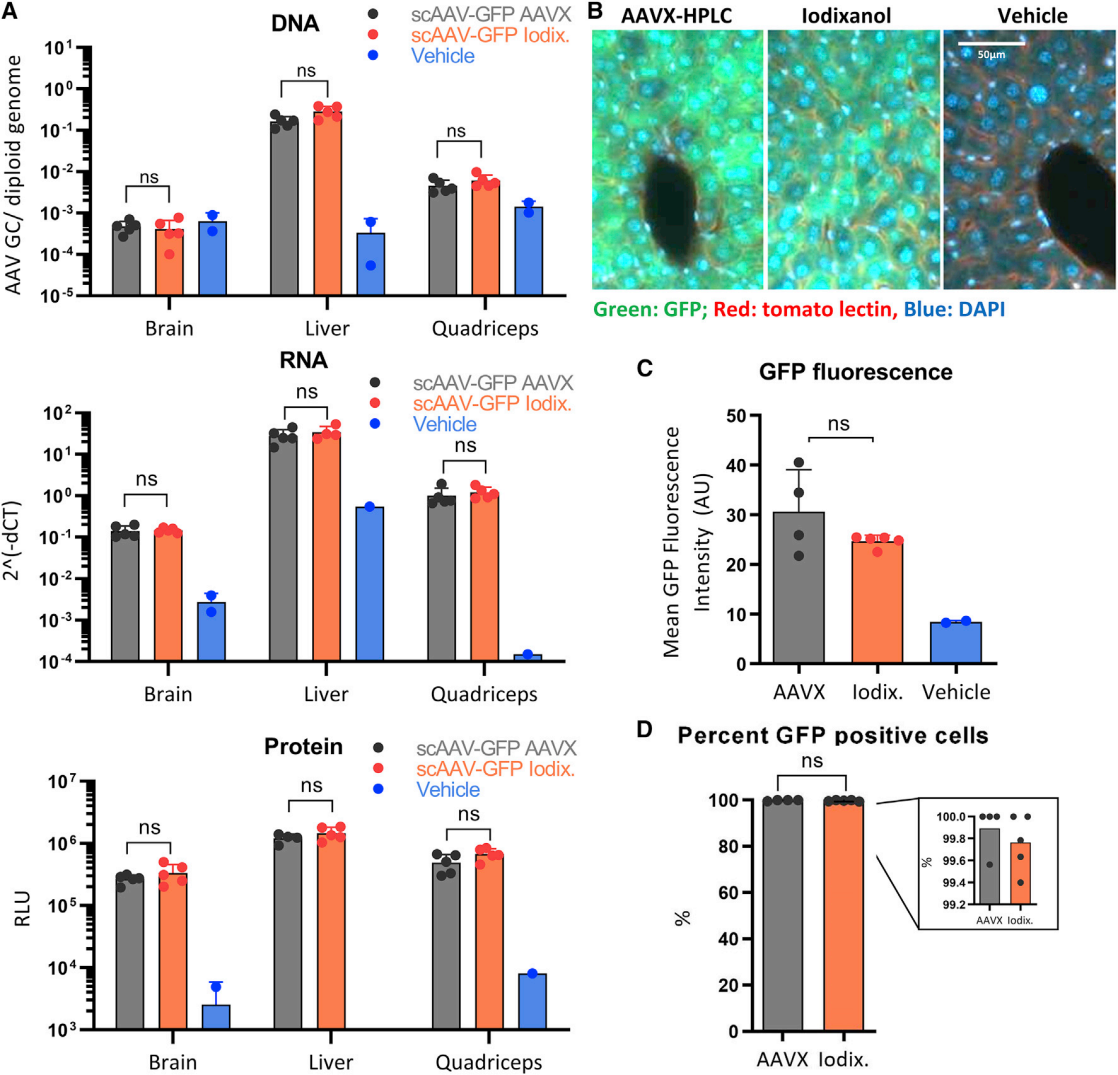
*In vivo* bioactivity of AAVX-HPLC and iodixanol ultracentrifugation purified AAV (A) Quantification of viral DNA and GFP RNA and protein levels in the liver, brain, and quadriceps of mice injected with a total of 10^11^ vg/mouse of scAAV9-Cbh-GFP. N = 5 for both scAAV-GFP AAVX and scAAV-GFP iodixinal injected mice, and N = 2 for vehicle-injected mice. DNA and RNA were quantified using qPCR and qRT-PCR, respectively, and protein using Simple Wes. Statistical significance was assessed using two-way ANOVA with Šídák’s post hoc tests. Statistically non-significant differences are not shown on the figure, except for AAVX versus iodixanol groups. Note that the AAV DNA levels in the brain were likely below the limit of quantification in this assay. (B–D) Imaging analysis of livers sectioned, stained for tomato lectin and DAPI, and imaged for native GFP fluorescence, tomato lectin, and DAPI. (C) Comparison of native GFP averaged from 400–700 cells per animal. (D) Percentage of cells that are GFP positive, counted as cells with a higher mean fluorescence intensity than the highest mean fluorescence intensity observed in the vehicle group. Statistical significance was assessed using one-way ANOVA with Tukey’s post hoc test for (C) and two-tailed t test for (D). ns, p > 0.05; ∗p < 0.05, ∗∗p < 0.01, ∗∗∗p < 0.001. Error bars denote standard deviation in all panels.

We observed similar results from our follow-on bioactivity experiments in mice. To compare *in vivo* bioactivity of HPLC and iodixanol purified viruses, we injected a total of 10^11^ vector genomes of self-complementary AAV9 carrying a Cbh-EGFP expression cassette retro-orbitally into 6-week-old wild-type male C57BL/6J mice. We euthanized mice 4 weeks post injection and assayed AAV DNA levels and biodistribution as well as GFP expression in liver, quadriceps, and brain. Transgene DNA, RNA, and protein levels did not significantly differ between AAVX-HPLC and iodixanol purified viruses for any tissues (Figure 5A). To confirm this observation, we sectioned, stained, and imaged livers of injected mice (Figure 5B). Image analysis indicates that EGFP mean fluorescence intensity does not differ significantly between animals injected with AAVX-HPLC and iodixanol purified vectors, and that vectors purified with both methods transduced almost 100% of liver cells (Figures 5C, 5D, S8, and S9). Taken together, these data indicate that AAVX-HPLC purified AAV is comparable in bioactivity with iodixanol ultracentrifugation purified AAV.

## Discussion

An increased demand in AAV research has led to the need to develop more versatile purification methods. Affinity chromatography has been considered a possible solution, but its application to AAV purification has been hampered by the lack of resins or processes that can purify multiple AAV serotypes efficiently without individual optimization.^10^^,^^11^^,^^12^^,^^13^

The main advantages of chromatographic purification are its scalability to larger volumes and reduced requirement for hands-on time, which considerably decreases costs and eases AAV manufacturing. Chromatographic resins can be scaled to high volumes, which enable input of unconcentrated large volumes of lysates. The process can also be automated and precisely controlled, monitored, and quantified, which eases troubleshooting and provides rich data about the quality of the run. For these reasons, chromatography-based methods have become the main workhorse for industrial production of biologics and small molecules.^9^ We find that AAVX affinity chromatography allows for purification of multiple AAV serotypes at multiple scales, is efficient, and results in vectors of comparable yield and bioactivity with ultracentrifugation purified vectors.

A possible disadvantage of using an AAVX affinity chromatography (or any other type of affinity chromatography) is the possibility that new and uncharacterized capsids may not bind to the resin. It is indeed possible that AAV variants that have substantial changes at the AAVX antibody binding site may have low or no affinity to the resin. This can be a particular concern for purification of libraries of diverse AAV capsid variants, for which ultracentrifugation-based methods may be more suitable. For purification of single AAV serotypes, however, this concern can be tested either experimentally or by identifying whether any of the expected changes of the novel capsid alter the AAVX binding epitope once it is definitively mapped. Nevertheless, the majority of basic and clinical research so far has been conducted with AAV capsids that we have verified to bind to AAVX in this work.^4^ As such, an AAVX affinity chromatography-based process should be broadly useful for most researchers in the field.

Another potential drawback of chromatographic purification is the co-purification of empty capsids. Indeed, several reports have described empty capsid co-purification to various degrees with affinity and other types of chromatography.^12^^,^^13^^,^^27^ In this study, we found the percentage of empty capsids in AAV9 and PHP.eB preps purified using AAVX affinity chromatography to be approximately 30%, compared with approximately 5% empty capsids in AAV9 preps purified using iodixanol ultracentrifugation (Figures 4B and 4C), suggesting that HPLC purification did not enrich for full capsids to the extent of iodixanol ultracentrifugation, if at all. However, despite the higher level of empty capsids, AAVX purified preps showed equivalent bioactivity to iodixanol purified preps both *in vitro* and *in vivo* (Figures 4D, 4E, and 5). Additionally, we have since carried out over 30 animal studies using AAVX-HPLC purified AAV and have observed satisfactory gene transfer in all of them (data not shown), indicating that the higher empty capsid content does not have an overt negative effect on efficacy.

However, for applications where maximal reduction of empty capsid content is required, various upstream or downstream steps that reduce the production of empty capsids or enrich for full capsids can be added. These include: optimization of plasmid transfection ratios;^28^ use of vector plasmids that are full length or with minimal inverted terminal repeat (ITR) deletion;^28^ use of novel engineered ITRs; use of a transfection plasmid containing both the AAV cap and transgene in *cis*;^28^ or other methods which have been reported to reduce the fraction of empty capsids in the input lysate. While we did not explore this in the present work, multiple different downstream steps to enrich for full capsids utilizing size exclusion, anion exchange, or other chromatographic methods have been recently reported.^10^^,^^11^^,^^13^^,^^29^^,^^30^^,^^31^^,^^32^^,^^33^^,^^34^^,^^35^ These can be added in series as additional steps to the process after the AAVX affinity binding step.

It should be noted that we estimated the empty capsid percentage in our preps using negative-stain TEM. Electron microscopy has the advantage of producing a clear visual of the AAV particle populations present, and when performed rigorously can match the results of analytical ultracentrifugation.^36^ While we performed the analysis based on published guidelines^36^ using two independent blinded operators, this method can nevertheless suffer from potential image noise, staining artifacts, or experimenter subjectivity at quantification.^37^ Therefore, future studies are needed to assess the impact of these methods on empty/full ratios of the yielded preparation including, e.g., analytical ultracentrifugation.

Using AAVX, we aimed to develop an integrated purification process for preps of at least 10^14^ vg. We found the main bottlenecks to be efficient cell lysis in the upstream process, and the loss or sedimentation of AAV at the buffer exchange step in the downstream process. To mitigate these, we incorporated *in situ* cell lysis using detergents and nucleases in the upstream process and buffer exchange using Amicon Stirred Cell in the downstream process (see “an optimized purification protocol”). These and other modifications increased process-wide efficiencies (from clarified lysate to purified preparation) to an average of approximately 75% while allowing resin re-use without loss of efficiency for at least six purification cycles (Figures 3C and S3). Additionally, we observed consistently high binding efficiencies between different batches of AAVX resin (Figures 2, S2, and S3) and consistently high overall purification efficiencies (Figures 3C and 3D) across all serotypes tested. This indicates that batch-to-batch variability of AAVX is low and that the protocol is overall robust and reproducible.

In summary, affinity chromatography with POROS CaptureSelect AAVX resin allows for high-efficiency purification of various AAV serotypes at multiple scales. The process developed here is primarily increased throughput and versatility applicable to laboratory studies. For clinical and/or scaled applications, further characterization on empty capsid content and elimination of the lysate clearance by centrifugation (e.g., by depth filtration or tangential-flow filtration) is needed. Here, we demonstrate the utility of AAVX in a cost- and time-effective process that does not require process modifications dependent on the serotype, thus being ideally suited for laboratory studies or centralized core facilities.

## Materials and methods

### AAV production and purification

All AAV vectors were produced in HEK293 cells via the triple plasmid transient transfection method as described previously.^6^ For small-scale preps (Figures 2A and S2), HEK293 cells were seeded in 15-cm dishes and grown to 80% confluence in Dulbecco’s modified Eagle’s medium (DMEM) containing 10% fetal bovine serum (FBS) (Gibco, 26140079) and 1% PenStrep (Thermo Fisher Scientific, 15140122). Cells were then triple transfected with the vector, AAV1 Rep/Cap (Addgene, 112862), and Ad helper plasmid (pAd delta F6 from UPenn) at a ratio of 1:1:2 (13:13:26 μg per 15-cm dish) using PEI Max 40000, pH 7.1 (Polysciences, 24765-1) at a ratio 1.375:1 of PEI/total DNA. Cells were harvested 3 days post transfection by scraping cells off the plate in their conditioned medium and lysing cells through 3× freeze-thaw cycles between 37°C and −80°C. Preps from three replicate plates were then pooled, incubated with 25 U/mL of benzonase (Millipore Sigma, E8263-25KU) at 37°C for 1 h to remove plasmid and cell DNA, centrifuged at 4°C and 4,000 × *g* for 30 min, and the supernatant filtered through a 0.22-μm polyethersulfone (PES) bottle-top filter (Corning, 431097). The filtered lysate was then split into two equal parts, with one part purified using standard HPLC purification reagents and the other part purified using reagents containing 0.1% v/v Pluronic F-68 (Thermo Fisher Scientific, 24040032) (described in Figures 2A and S2, respectively).

For HYPERFlask scale preps described in Figures 1C–1E, HEK293 cells at 80% confluence from four 15-cm dishes were seeded to a HYPERFlask (Millipore Sigma, CLS10031-4EA), grown to 80% confluence, and triple transfected with AAV vector Rep/Cap for AAV2, Anc80, PHP.eB, or AAV9 (AAV2: Addgene, 104963; Anc80: Zinn et al.;^17^ PHP.eB: Addgene, 103005; AAV9: Addgene, 112865), and pAdΔF6 at 130:130:260 μg per HYPERFlask, respectively. Three days after transfection cells were lysed, and clarified harvests (560 mL) were treated with 12,500 total units of benzonase (Millipore Sigma, E8263-25KU) for 30 min at 37°C, and this step was repeated with an additional 2,500 total units of benzonase for a further 1 h at 37°C to remove plasmid and cell DNA. The harvest was precipitated overnight at 4°C in high salt solution (80 mL of 5 M NaCl). The clarified lysate was obtained by centrifugation at 4,000 × *g* for 30 min at 4°C. The supernatant was collected and filtered using a 0.22-μm PES filter unit (130 mm diameter filter, Foxx Life Sciences, 1103-RLS) before HPLC purification. Centrifugation for lysate clarification was performed for 30–60 min at 4,000–10,000 × *g*. Ultracentrifugation was performed at 200,000–350,000 × *g* for 90–120 min (using the T70i rotor).

Iodixanol ultracentrifugation purified preps were produced in the Gene Transfer Vector Core at Schepens Eye Research Institute. HEK293 cells were seeded and transfected into HYPERFlasks, followed by benzonase (Sigma-Aldrich, E8263) treatment and high salt lysis as described above. The lysate was then clarified, concentrated using tangential-flow filtration, and purified via iodixanol gradient ultracentrifugation and buffer exchange with FFB (1× PBS, 172 mM NaCl, 0.001% Pluronic F-68).

### Static binding assay

Preparations of AAV2, AAV2_HSPG, AAV4, AAV5, AAV6.2, AAV7, AAV8, AAV9, AAVrh10, AAVrh32.33, AAV-PHP.B, Anc80, and AAV7m8 were produced and purified via ultracentrifugation on an iodixanol gradient as described above. To perform the static binding assay, the AAVX resin was first conditioned through three washes in 0.1 M NaCl (4 mL, in a 5-mL Eppendorf tube) and equilibrated through incubation in PBS. To perform the washes, resin was pulse centrifuged to pellet the resin and discard the supernatant in 5-mL Eppendorf tubes. Next, 50 μL of resin was suspended in 1 mL of PBS and 0.001% Pluronic F68, and 5 × 10^10^ vg of AAV was added. Each AAV serotype was added to a separate tube of unused resin. AAV was then incubated with the resin by rocking at room temperature for 10 min in 1.5-mL Eppendorf tubes. Flow-through was collected by pulse spinning and collecting the supernatant, and resin was washed thrice using 1 mL of PBS, with wash fractions collected. Finally, AAV was eluted twice using 1 mL of 0.1 M citric acid (pH 2) and AAV vector genomes quantified in each fraction using qPCR.

### High-efficiency purification protocol

For HYPERFlask scale preps described in Figure 3, an optimized protocol based on Florencio et al.^26^ and our own observations were used. HEK293 cells at 80% confluence from four 15-cm dishes were seeded to a HYPERFlask, grown to 80% confluence (normally approximately 48 h after seeding), and triple transfected with AAV vector Rep/Cap for AAV9 or PHP.eB and pAdΔF6 at 130:130:260 μg per HYPERFlask, respectively. Four days post transfection, supernatant from a HYPERFlask was decanted into a 1-L flask and 3 mL of Triton X-100 (Millipore Sigma, 8787-100ML), 2.5 mg of RNAse A at 1 mg/mL concentration (Millipore Sigma, 10109142001), 25 U/mL Turbonuclease (VitaScientific, ACGC80007), and 56 μL of 10% Pluronic F-68 (Thermo Fisher Scientific, 24040032) were added to the supernatant. The contents were then mixed and then poured back into the HYPERFlask, and the HYPERFlask was shaken on an orbital shaker at 150 rpm at 37°C for 1 h to lyse the cells and remove plasmid DNA. Lysate was then decanted from the HYPERFlask, and the HYPERFlask was washed with 140 mL of Dulbecco’s PBS (Life Tech, 10010072), which was added to the rest of the lysate. The total lysate was then centrifuged at 4,000 × *g* at 4°C for 30 min, and the supernatant was filtered through a 0.45-μm PES bottle-top filter (Thermo Fisher Scientific, 295-4545) before loading onto the HPLC system. Here, we used a 0.45-μm PES bottletop filter as opposed to a 0.22-μm filter that we used in the unoptimized protocol, because the 0.45-μm filter allowed for a much faster filtration and did not negatively affect follow-on HPLC purification.

### High-performance liquid chromatography

AAV purification was performed using AAVX POROS CaptureSelect (Thermo Fisher Scientific) resin bought as pre-packed 1-mL columns (Thermo Fisher Scientific, A36652) or free AAVX resin (Thermo Fisher Scientific, A36741) packed into 6.6 × 100-mm column (glass Omnifit; Kinesis USA). Columns were attached to an AKTA Pure 25 L HPLC system (GE Life Sciences, 29018224) containing an auxiliary sample pump S9 (GE Life Sciences, 29027745). The machine was housed at room temperature and all purifications were performed at room temperature (approximately 24°C), except for experiments described in Figure 3D. Column volume ([CV]) for each purification was set as 1 mL regardless of the actual volume of the resin used. For purifications using more than 1 mL of resin, a protocol with increased wash times was employed (see supplemental files). The chromatography column was pre-equilibrated with 10 [CV] of wash buffer 1× TBS (Boston Bioproducts) before application of AAV lysate. Equilibration and all subsequent washes of the column were performed at a rate of 2 mL/min.

Lysate was clarified at most 1 day prior to loading onto the HPLC and warmed up to room temperature prior to loading. Lysate was loaded at a flow-rate-to-resin-volume ratio ensuring approximately 2 min residence time in the resin, normally using 1 mL of resin and a flow rate of 0.5 mL/min, or 4 mL resin with a flow rate of 2 mL/min. At least 1 mL of resin per one HYPERFlask was used; if preps from multiple HYPERFlasks were pooled together, the volume of resin was increased accordingly.

For purifications using 1 mL of resin, the column containing bound AAV was then washed with 10 [CV] of 1× TBS, followed by washes of 5 [CV] of 2× TBS, 10 [CV] 20% ethanol, and 10 [CV] 1× TBS wash. The bound AAV was eluted using a low-pH (pH 2.5–2.9) buffer of 0.2 M glycine in 1× TBS, containing 0.01% (v/v) Pluronic F-68 at a rate of 1 mL/min. Resin was then washed with 10 [CV] of 1× TBS regenerated with 15 [CV] 0.1 M phosphoric acid (pH 1) and 15 [CV] 6 M guanidine-HCl at flow rate of 1 mL/min, and washed again with 10 [CV] 20% ethanol and 10 [CV] 1× TBS. Elution fractions were taken as 1-mL volumes per fraction. The eluted vector solution was neutralized by adding 1 M Tris-HCl (pH 8.0) at one-tenth of the fraction volume directly into the fraction collection tube prior to elution. Peak fractions based on UV (280 nm) absorption graphs were collected, filter sterilized using 0.2-μm PES syringe filters (Corning, 431229), buffer exchanged using either Amicon Ultracel 15 (Merck Millipore, UFC910008) or Amicon Stirred Cell (Merck Millipore, UFSC05001) concentrators with a molecular weight cutoff of 50 kDa or 100 kDa (Millipore, UFC905008 EMD) prior to virus titration. For Amicon Stirred Cell concentrator, high-purity nitrogen gas (NI UHP80 Airgas) was used at 40–70 psi as a pressure source. All plasticware and tips were coated or incubated with FFB for approximately 15 min at room temperature prior to applying AAV-containing solutions at any step of the purification process.

### Quantitative PCR and digital droplet PCR

In brief, genomic titer was determined by a qPCR (TaqMan, Life Technologies) as well as digital droplet PCR (ddPCR). For qPCR, real-time qPCR (7500 Real-Time PCR System; Applied Biosystems, Foster City, CA, USA) with EGFP-targeted primer-probes (AGC AAA GAC CCC AAC GAG AA, GGC GGC GGT CAC GAA, 6FAM-CGC GAT CAC ATG GTC CTG CTG G-TAMRA) were used. We used linearized CBA-EGFP DNA at a series of dilutions of known concentration as a standard. After 95°C holding stage for 10 s, two-step PCR cycling stage was performed at 95°C for 5 s, followed by 60°C for 5 s for 40 cycles. Genomic vector titers were interpolated from the standard. qPCR was used to determine titers for experiments described in Figure 1, Figure 2, Figure 3, 2, 3, and S2–S6.

For ddPCR, QX200 ddPCR system (Bio-Rad) using the same EGFP-targeted primer-probes as described above were used. ddPCR and titer estimation was performed as previously described by Sanmiguel et al.^38^ ddPCR was used to estimate titers of the vectors for experiments described in Figure 4, Figure 5A, 4B, 5, S7, and S8.

### Protein gel analysis

All materials and reagents used were purchased from Life Technologies. Equal vector genomes of AAV were loaded on a NUPAGE 4%–12% Bis-Tris polyacrylamide gel (Life Technologies, Grand Island, NJ) and subjected to electrophoresis at 150 V for 1 h 30 min. For each AAV preparation, a volume corresponding to a titer of 10^10^ vg was mixed with 5 μL of 4× NuPAGE lithium dodecyl sulfate sample buffer and 1× PBS (Corning, 21-031-CM) to 20 μL total volume and heat denatured at 70°C for 5 min.

SYPRO Ruby Protein Gel Stain (Thermo Fisher Scientific) was applied per the manufacturer’s protocol to visualize and analyze SDS-PAGE bands. In brief, the gel was fixed in 7% glacial acetic acid and 50% methanol (ACS grade, Thermo Fisher Scientific) in ultrapure water for 15 min at 21°C (room temperature) by gentle agitation. Fixation was repeated once more before gel was rinsed with ultrapure water. Gel was stained with SYPRO Ruby as follows: 30 s microwave, 30 s agitation, 30 s microwave, 5 min agitation, 30 s microwave, 23 min agitation. Gel was rinsed with ultrapure water and destained with 7% glacial acetic acid and 10% methanol for 30 min at 21°C (room temperature) by gentle agitation. Proteins stained with the dye were visualized with a 302-nm UV transilluminator (ChemiDoc XRS + Bio-Rad).

### Empty capsid estimation via transmission electron microscopy

Purified and formulated AAV from different preps was diluted to a concentration of 10^13^ vg/mL and submitted for negative stain and TEM analysis at Harvard Medical School Electron Microscopy Core. In brief, the sample was diluted in water and adsorbed onto a glow-discharged carbon or formvar/carbon-coated grid. Once the specimen was adsorbed on to the film surface, the excess liquid was blotted off using a filter paper (Whatman #1) and the grid was floated on a small drop (∼5 μL) of staining solution (most commonly 0.75% uranyl formate, 1% uranyl acetate, or 1%–2% phosphotungstatic acid). After 20 s the excess stain was blotted off and the sample was air dried briefly before examination in the transmission electron microscope. At least two images were taken per prep at 30,000× magnification, and at least 200 virions were counted manually per image by two researchers blinded to the identity of the image; empty and full ratios were averaged between resulting counts. Because of the difficulty in confidently differentiating full and partially filled capsids using electron micrographs, virions were counted as empty and full only based on the criteria described in Fu et al.^36^ On the minority of cases where a virion could not be confidently assigned to either (<1% capsids), the virion was not counted. Similarly, virions were not counted in areas of images with image noise, artifacts, clumping, or other effects that obscure a clear classification of the virion type.

### Next-generation sequencing and analysis

For Figures 2B and S2B, five different AAV1 preps were produced, where the vectors from the second to fifth prep were identical except a unique DNA barcode region. The preps were purified consecutively from prep 1 to prep 5, and the barcode region was PCR amplified in the elution fractions of the fifth preps. The amplicons were PCR amplified and submitted for Amplicon Seq at the MGH DNA Sequencing Core. Finally, the number of barcode reads corresponding to AAVs from each of the preps 2–5 was directly counted from the resulting FASTQ file. The vast majority of barcodes present came from the fifth preps (barcodes from previous preps were present at less than 0.1%).

### AAV *in vitro* studies

HEK293 cells were seeded at 1 × 10^5^ cells/well, N = 4 replicates, in 500 μL of complete DMEM containing 10% (v/v) heat-inactivated FBS, and 100 U/mL penicillin and 100 μg/mL streptomycin into a 24-well plate, and AAV was added immediately at a multiplicity of infection of 10^5^ vg/cell. The vector was self-complementary AAV9 carrying a CBh-GFP expression cassette. Cells were washed with PBS 3 days later and imaged using a Leica Observer D1 microscope, using a 10× objective. Exposure and light power were adjusted such as to place the GFP signal from vehicle transduced cells to the bottom fifth of the signal range.

### AAV *in vivo* studies

All animal procedures were performed with the approval of the Institutional Animal Care and Use Committee at Schepens Eye Research Institute. For assaying *in vivo* potency and transduction, self-complementary AAV9 carrying a Cbh-EGFP expression cassette was produced at the HYPERFlask scale and purified with AAVX-HPLC or iodixanol ultracentrifugation, concentrated in FFB, and stored at −80°C until use. Six-week-old male C57BL/6J mice (N = 5 each for cohorts injected with AAVX and iodixanol purified vectors, and N = 2 for vehicle-injected vectors) were then injected retro-orbitally with a total dose of 10^11^ vg (in 100 μL volume of FFB) per mouse. Mice were euthanized 4 weeks post injection and brain, quadriceps, and liver harvested. One part of each tissue was snap frozen in liquid nitrogen for analysis of vector DNA and EGFP RNA and protein (see below). Another part of each tissue was fixed in paraformaldehyde (PFA) for later sectioning and processing for immunofluorescence imaging (see immunofluorescence and image analysis).

### DNA, RNA, and protein quantification

Tissues were homogenized by disrupting 30 mg of tissue in 1 mL of RLT+ buffer for DNA and RNA and 1 mL of RIPA buffer containing 1× Halt protease and phosphatase inhibitors for protein (Thermo Fisher Scientific, 78444). For disruption, samples, buffer, and 1-mm zirconia/silica beads (Biospec, 11079110z) were loaded into XXtuff vials (BioSpec, 330TX) and disrupted using Mini Beadbeater 24 (BioSpec,112011) at maximum speed for 3 min. Vials were then placed on ice for 2–5 min for RNA and 1 h for protein, centrifuged at 10,000 × *g* for 3 min, and the resulting supernatant used for further procedures.

For DNA/RNA, 700 μL of supernatant was loaded onto AllPrep DNA Mini Spin columns and purified using AllPrep DNA/RNA/miRNA Universal Kit (Qiagen, 80224) for quadriceps and AllPrep DNA/RNA mini kit (Qiagen, 80204) for brain and liver. Purification was performed on Qiacube Connect (Qiagen, 9002864).

Total AAV genome copy number was assessed by qPCR using GFP primer-probe sets and quantified using linearized CBA-GFP plasmid serial dilutions as the standard for AAV copy number (AGC AAA GAC CCC AAC GAG AA, GGC GGC GGT CAC GAA, 6FAM-CGC GAT CAC ATG GTC CTG CTG G-TAMRA). Total cell genome copy number was estimated using RPII primer-probes (GTT TTC ATC ACT GTT CAT GAT GC, TCA TGG GCA TTA CTA TTC CTA C, probe: VIC-AGG ACC AGC TTC TCT GCA TTA TCA TCG TTG AAG AT-3IABkFQ) along with a standard of gDNA dilution series of known concentration. AAV copy number per diploid genome was then calculated as $copy\;number\;per\;diploid\;genome=2\times \left(\frac{total\;AAV\;copy\;number}{total\;genome\;copy\;number}\;\right)$. Efficiency and specificity of amplification for both primer-probe sets was previously established, and amplification was performed using Luna Universal Probe qPCR Master Mix (NEB, M3004L) at thermocycling conditions recommended by the manufacturer.

For quantification of GFP RNA expression, RNA extracted from tissues was first treated with DNAse (DNA-free DNA Removal Kit; Thermo Fisher Scientific, AM1906) and then reverse transcribed and amplified using Luna Universal Probe One-Step qRT-PCR Kit (NEB, E3006L) according to the manufacturer’s instructions. Primer-probe sets for GFP cDNA (AGC AAA GAC CCC AAC GAG AA, GGC GGC GGT CAC GAA, 6FAM-CGC GAT CAC ATG GTC CTG CTG G-TAMRA) and RPII cDNA (GTT TTC ATC ACT GTT CAT GAT GC, AAT CAA TGC AGG TTT TGG CGA TG, probe: VIC-AGG ACC AGC TTC TCT GCA TTA TCA TCG TTG AAG AT-3IABkFQ) were used. Controls lacking reverse transcriptase were run to preclude signal from DNA contamination. Expression of GFP RNA normalized to RPII RNA was then calculated as $2^{-\left({Ct}_{GFP}-{Ct}_{RPII}\right)}$.

For quantification of GFP protein expression, protein lysate was first diluted 5× twice in fresh RIPA + 1× Halt inhibitors buffer, and all dilutions were assayed for total protein content using a Pierce BCA Protein Assay Kit (Thermo Fisher Scientific, 23225). For each tissue type, lysates were diluted in RIPA + 1× Halt buffer to the concentrations of: liver, 0.05 μg/μL; brain, 1.5 μg/μL; quadriceps, 1.5 μg/μL. Protein levels were then assayed using anti-GFP antibody ab290 (Abcam, ab290) on Wes (Protein Simple) with the 12–230 kDa chemiluminescence assay (12–230 kDa Jess or Wes Separation Module; Protein Simple, SM-W004). Linear range for GFP quantification was previously determined by assaying GFP using Wes with ab290 antibody for dilutions ranging from ∼5 μg/μL to 0.03 μg/μL (linear range: liver <0.3 μg/μL, brain 0.3 μg/μL to ∼3 μg/μL, quadriceps 0.03 μg/μL to ∼3 μg/μL). Linear range for total protein was also previously determined by Wes 12–230 kDa Total Protein Size assay in the range of 4 μg/μL to 0.1 μg/μL using Total Protein Detection Module (Protein Simple, DM-TP01) according to the manufacturer’s instructions. Linear range was found to be < 1 μg/μL for all tissues tested. GFP and total protein levels were then quantified using Compass for SW 4.1 (Protein Simple). Finally, GFP was normalized to total protein to arrive at the final value.

### Immunofluorescence and image analysis

Tissues were fixed in 1% PFA for 4 h and then 4% PFA for 1 h at room temperature (21°C). Fixed tissues were then washed with 1× PBS three times for 5 min, placed in 30% sucrose for approximately 48 h at 4°C, and frozen in OCT blocks by submersion into isopentane cooled by liquid nitrogen. Blocks were then sectioned at 12 μm thickness using iHisto cryosectioning service (iHisto). Sections were kept at −80°C until staining. Sections were blocked using blocking buffer (10% normal goat serum, 2% BSA, 0.1% Tween 20) for 1 h, washed 3 × 5 min with PBS-T (PBS + 0.1% Tween 20), stained with tomato lectin at 10 μg/mL (Vector Laboratories, DL-1177) for 1 h, washed 3 × 5 min with PBS-T, stained with 4′,6-diamidino-2-phenylindole (DAPI) for 5 min at 1:1,000 stock concentration (Thermo Fisher Scientific, D1306), mounted for 15 min (Vector Laboratories, H-1400) and imaged for native GFP, tomato lectin, and DAPI. All actions were performed at 21°C in a dark room. Slides were imaged using a Zeiss Axio Observer D1 microscope (exposure times were set such that signal intensities from samples with the brightest signals would appear in the lower third of the histogram). Exposures were kept constant between all samples for all three colors imaged. For each tissue, two sections from the middle of the tissue were imaged, with 6–8 fields in total imaged at 200× magnification.

Three images of different sites were then selected, all cells within the images circled for regions of interest (ROIs), and cell GFP mean fluorescence intensity quantified within ROIs in Fiji.^39^ Cells were circled conservatively to make sure only individual cells were circled. A total of 400–700 cells were quantified per animal, and mean fluorescence intensity values across different cells averaged to arrive at an overall liver GFP mean fluorescence intensity per animal.

### AAV phylogenetic analysis

To generate the phylogeny, first 19 representative AAV capsids were chosen, including an avian AAV (VR-865) for use as an outgroup for eventual tree rooting. The VP1 amino acid sequences from all of these different isolates were aligned through ClustalOmega^40^ as implemented on the EMBL-EBI webserver.^41^ Substitutions models and parameters for an eventual maximum likelihood (ML) phylogenic analysis were evaluated by ProtTest3,^42^ and the best-fitting model by the Aikake Information Criterion was selected. The model best describing the set of AAV sequences was the Le and Gascuel model,^43^ with a discrete Gamma distribution (five categories) to model rate differences among sites within the alignment. This model was used to construct an ML phylogeny through MEGA X^44^ before being exported and visualized through phytools.^45^ See Figures S10–S12 for multiple sequence alignment, sequence percent identity, and Newick formatted phylogeny of the phylogeny depicted in Figure 1B.

### Statistical analysis

All data were visualized, and statistical analysis was performed using GraphPad Prism (GraphPad). Specific statistical tests used are listed in figure legends for each test, and all tests were performed with default settings unless otherwise specified.

## Data Availability

All data will be made available upon request.

## Supplemental information

Document S1. Figures S1–S12 and supplemental protocol

Document S2. Supplemental files

Document S3. Article plus supplemental information
