# Supplementary material for: High-efficiency purification of divergent AAV serotypes using AAVX affinity chromatography
Source: Mol Ther Methods Clin Dev. 2022 Dec 16;28:146–59. doi: 10.1016/j.omtm.2022.12.009 (PMC9823220; doi:10.1016/j.omtm.2022.12.009)
Supplement: Document S1. Figures S1–S12 and supplemental protocol [file mmc1.pdf]

OMTM, Volume *28*

Supplemental information

High-efficiency purification of divergent AAV serotypes using AAVX affinity chromatography

Michael Florea, Fotini Nicolaou, Simon Pacouret, Eric M. Zinn, Julio Sanmiguel, Eva Andres-Mateos, Carmen Unzu, Amy J. Wagers, and Luk H. Vandenberghe

Supplemental Figures

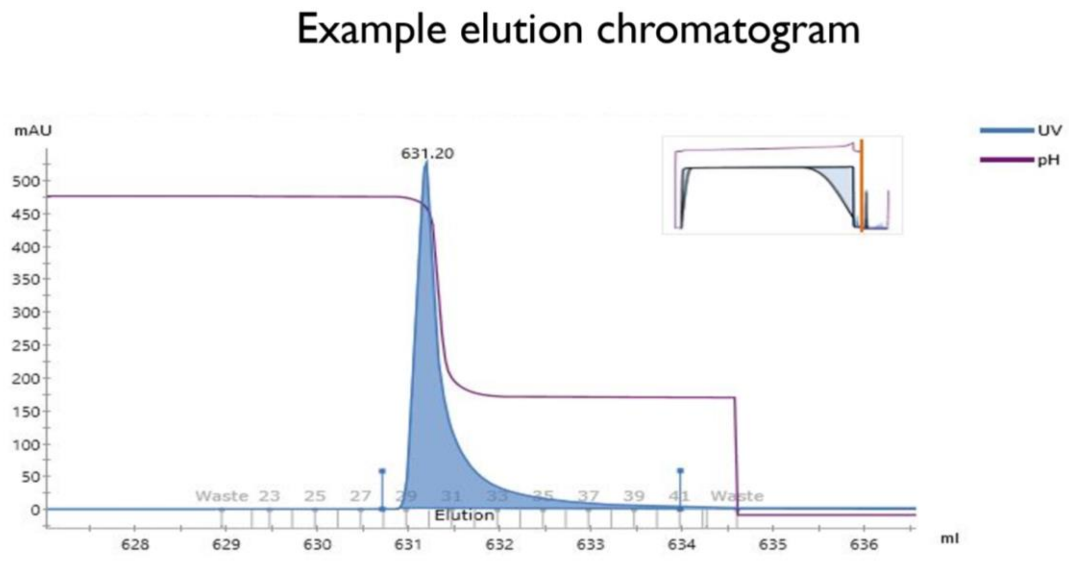

Figure S1. HPLC chromatogram of AAV2 purification from one hyperflask. Chromatogram shows tight a elution peak with a corresponding drop in the pH, as the elution buffer is applied to the column. Inset: chromatogram of the whole purification with the major UV plateau corresponding to the sample application stage.

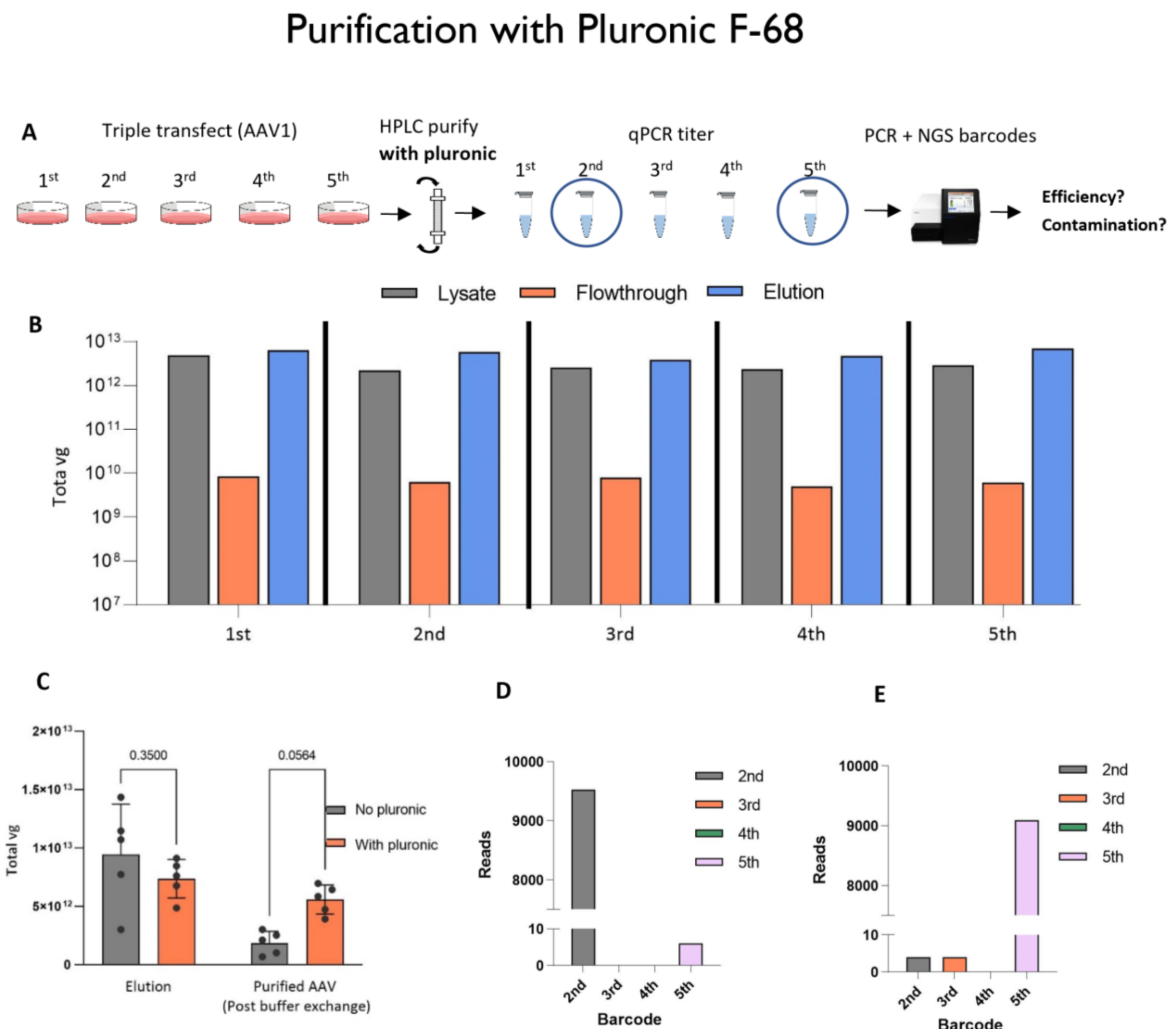

Figure S2. AAV purification at small scale over multiple cycles with Pluronic F-68 added to 0.1% vol/vol to all buffers. (A) Schematic of the experiment. **(B)** qPCR quantification of AAV vector genomes in different fractions, along preps 1-6. **(C)** Comparison of total AAV vector genomes after elution and filtration+buffer exchange with or without Pluronic F-68. Addition of Pluronic F-68 does not increase yields at the elution step, but shows a trend towards increased yields at the filtration+buffer exchange step. **(D-E)** NGS quantification of unique barcode count from the elution fractions of the 2nd prep (D) and 5th prep (E). Majority of barcodes come from the target prep, indicating low carryover contamination. P-values indicated above bars, determined via two-way ANOVA with Šídák's multiple comparisons test.

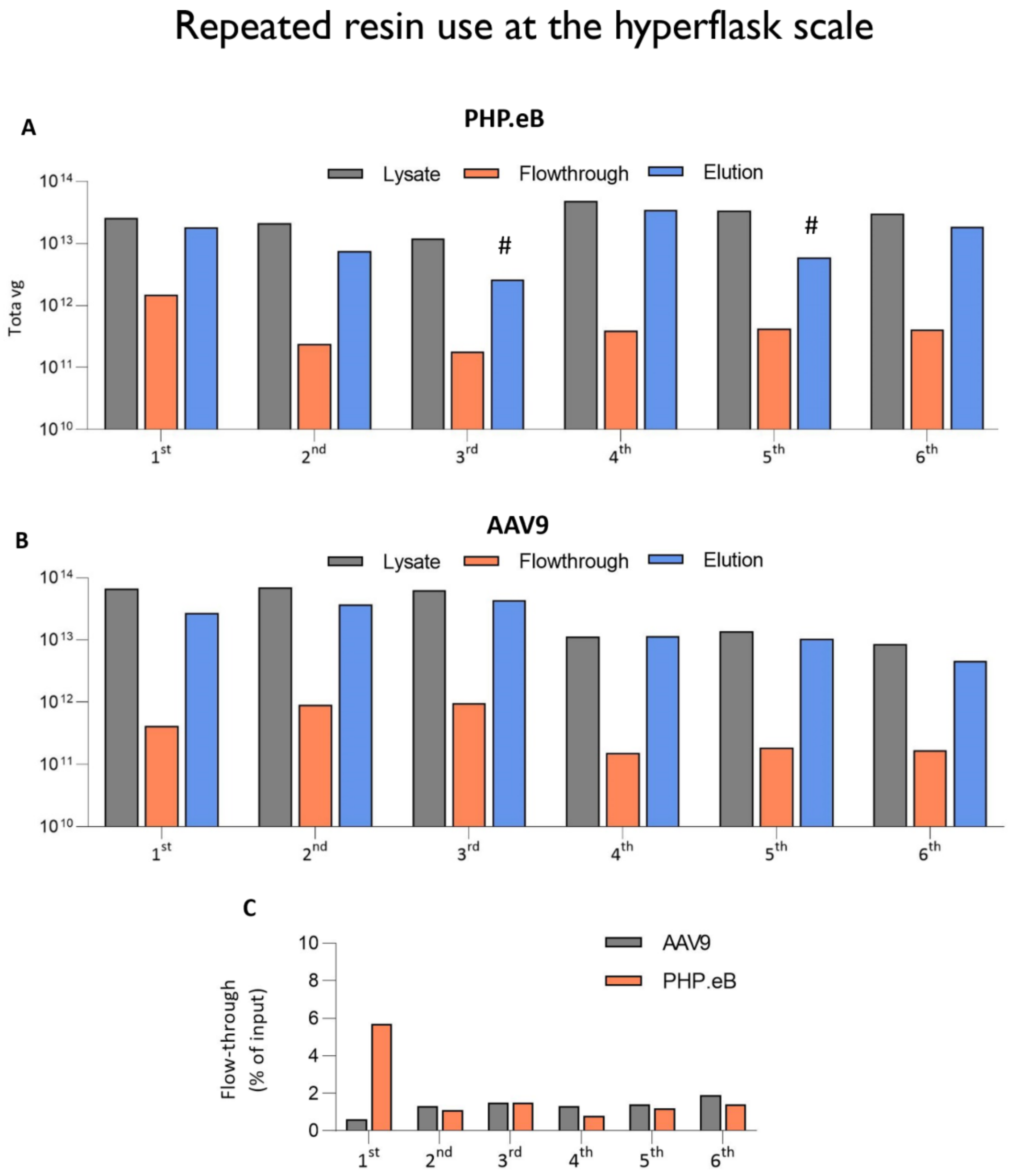

Figure S3. Stringent resin cleaning enables repeated resin re-use at large scale. Input from 1 hyperflask at each step was purified without changing the resin and AAV in input lysate, flow-through and elution tittered using qPCR. The process was repeated for PHP.eB **(A)** and AAV9 using new batches of resin for each **(B)**. AAV applied at room temperature, at 2 min residence time, 3 mL resin, eluted using pH 2.5 Glycine and resin regenerated using 1ml/min flow of 0.1M pH1 Phosphoric acid followed by 1ml/min flow of 6M Guanidine HCl for 15 minutes each. **(C)** No increase in % of AAV in flow-through was seen throughout 6 cycles. # some eluate lost due to operator error. # - some sample was lost due to handling error

AAV loss at each purification step

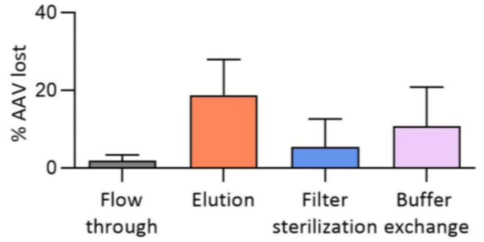

Figure S4. Percent AAV lost at each step of high-efficiency protocol. Largest losses occur at the elution (~20% of input) and buffer exchange (~10% of input) steps. Data from Fig. 4 with AAV9 and PHP.eB combined, with N=6 for each.

Full protein gel images

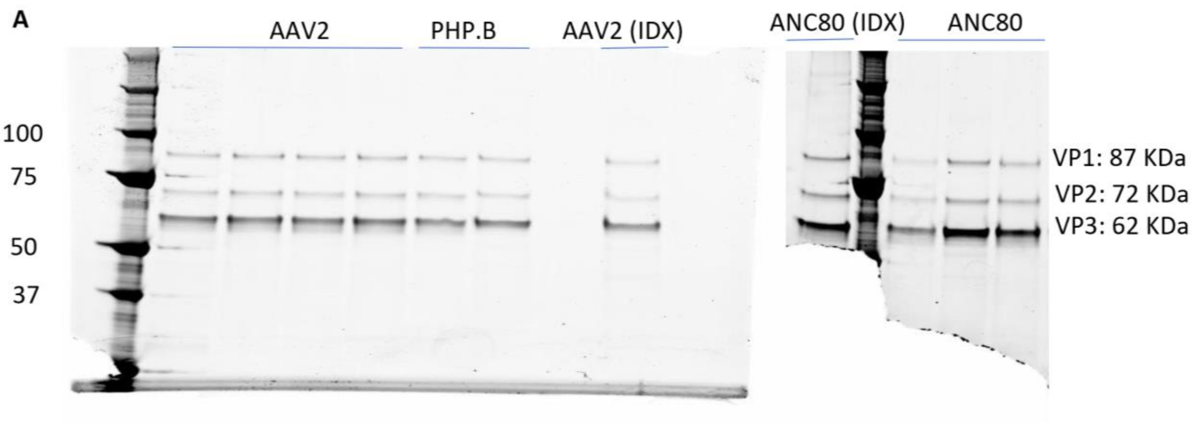

Figure S5. Uncropped gels of silver stain analysis of AAV capsids from Figure 4A.

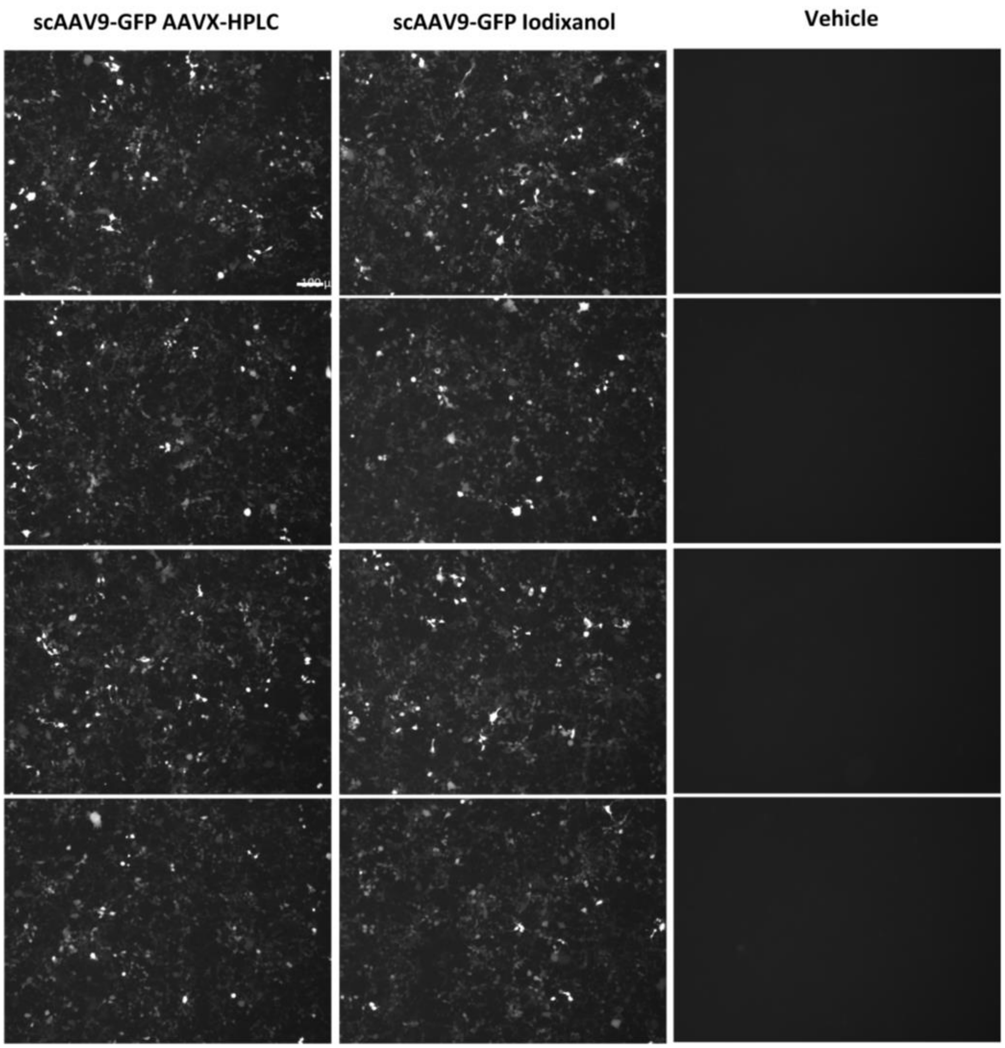

Figure S6. Full GFP images from Figure 4 B-C.

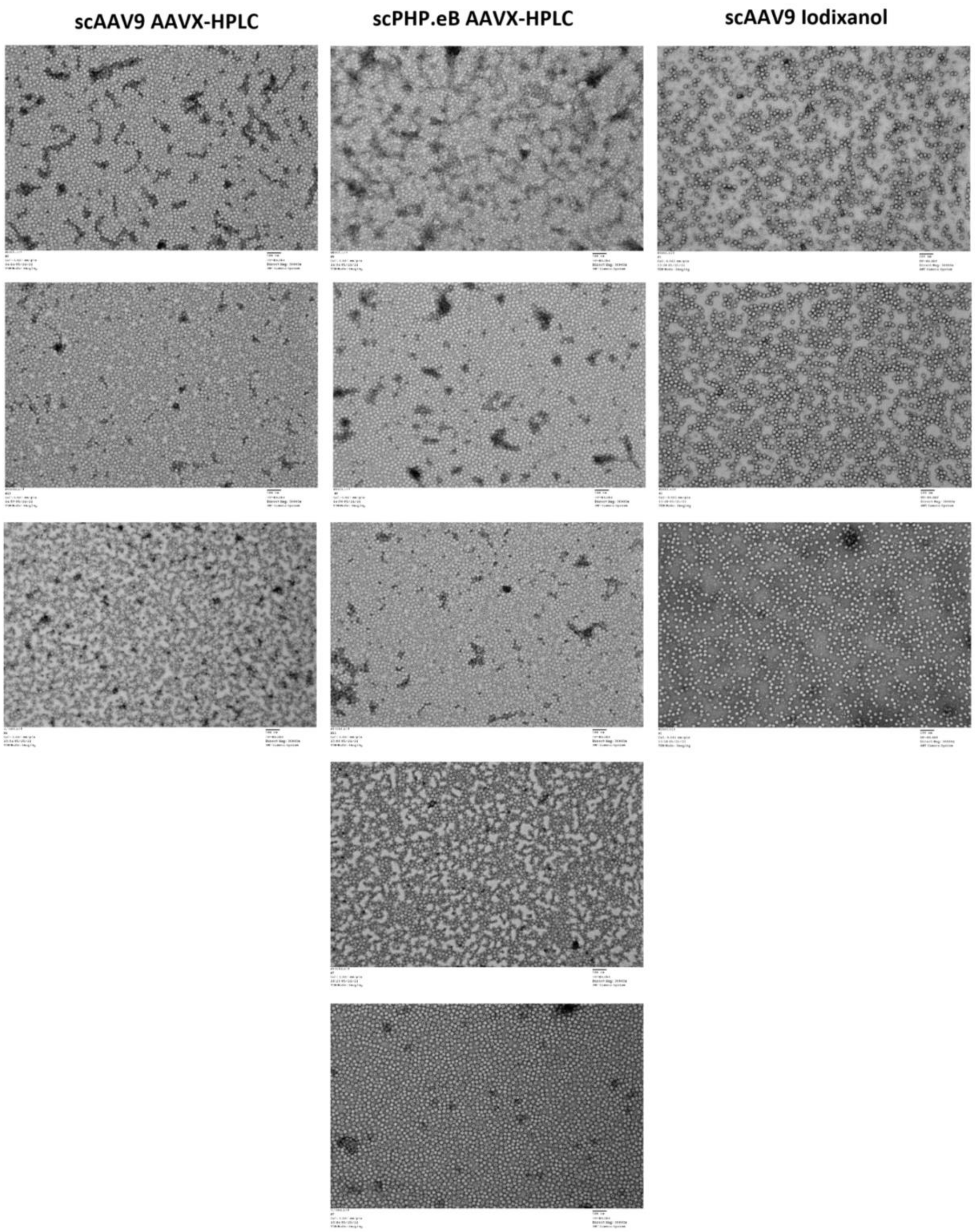

Figure S7. Full negative stain SEM images of scPHP.eB and scAAV9 preps described in Fig. 4D-E. Each image represents a separate prep. In quantification, a minimum of two images were taken and quantified for each prep.

Full liver fluorescence images

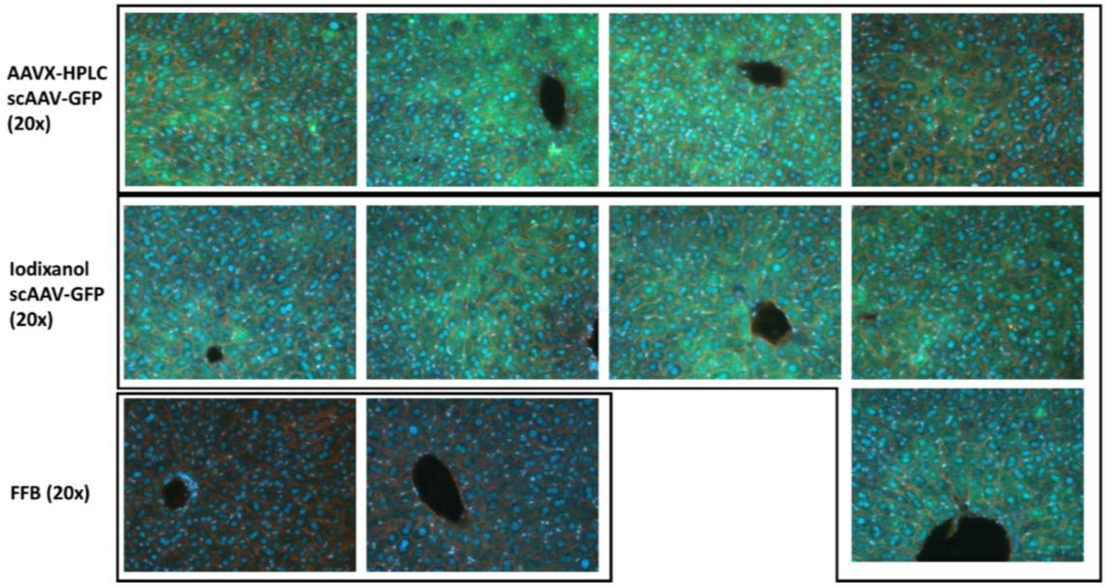

Figure S8. Images used for GFP fluorescence intensity analysis shown on Figure 5B. Every image corresponds to a different animal within the groups denoted on the left.

Individual cell GFP fluorescence

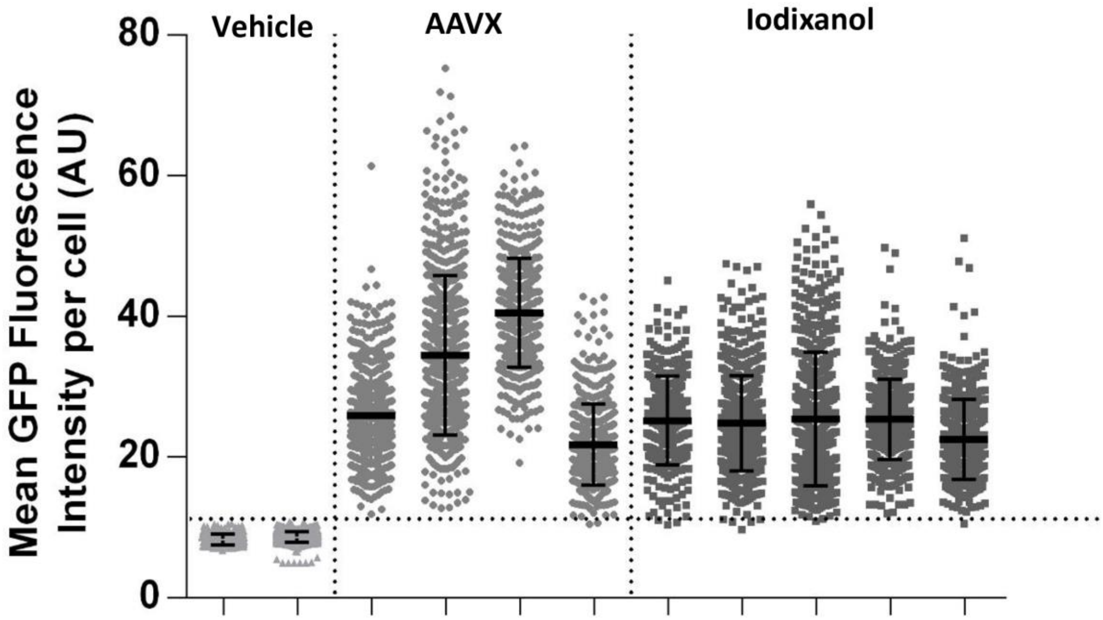

Figure S9. Individual cell GFP mean fluorescence intensities of animals injected with AAVX-HPLC or iodixanol ultracentrifugation purified AAV. Every column represents one animal and 3 images were used per animal, resulting in a total of 400-700 cells analysed per animal. Horizontal dotted line represents the mean fluorescence intensity above which cells were counted as GFP positive.

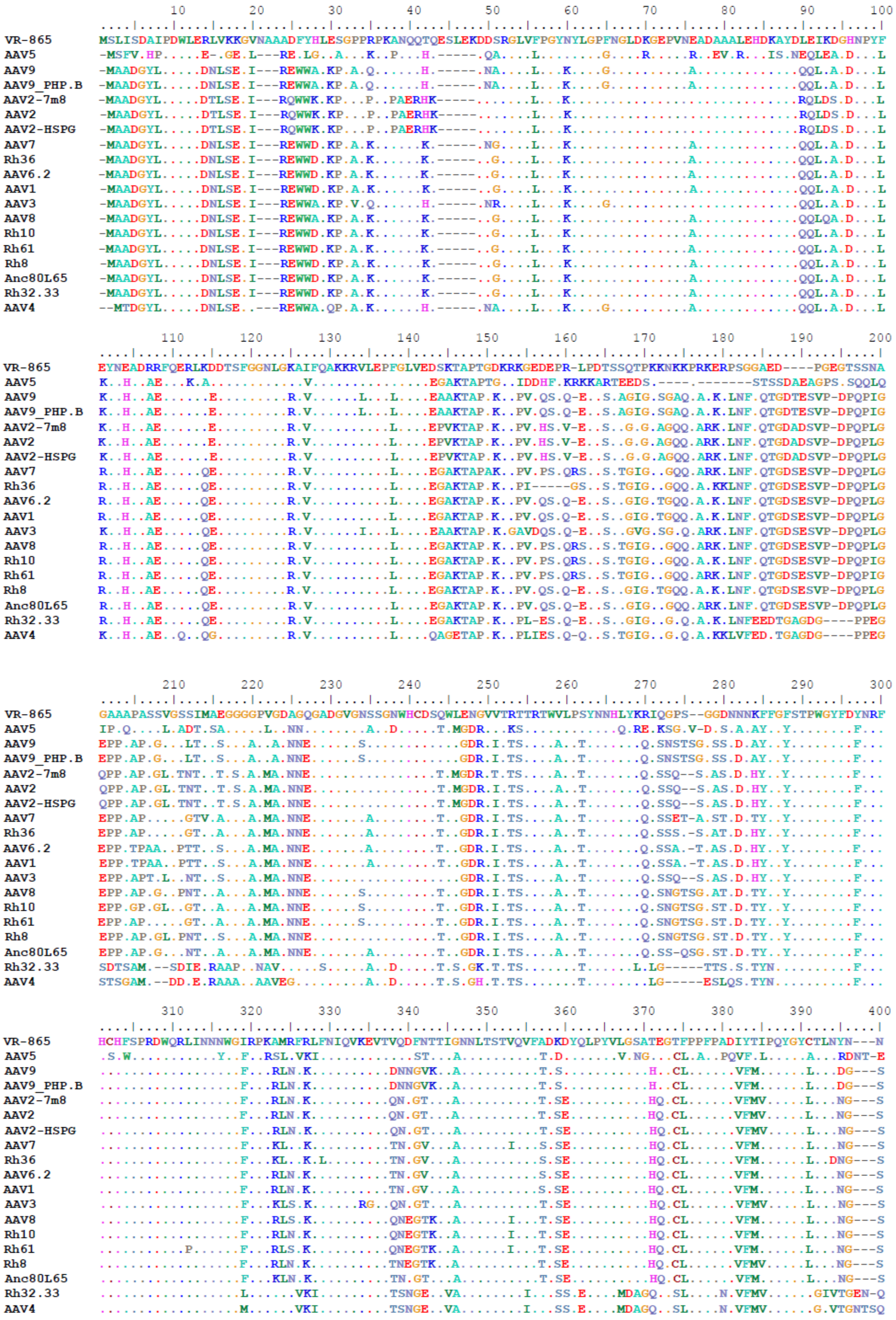

Figure S10. Multiple Sequence Alignment of AAVs used to construct the phylogenetic tree depicted in Fig. 1B.

```
                     410       420       430       440       450       460       470       480       490       500
             ....|....|....|....|....|....|....|....|....|....|....|....|....|....|....|....|....|....|....|....|
VR-865       EAVDRSAFYCLDYFPSDMLRTGNNFEFTYTFEDVPFHSMFAHNQTLDRLMNPLVDQYLWAFSSVSQA---GSSGRALHYSRATKTNMAAQYRNWLPGPFF
AAV5         NPTE..S.F..E....K...........N..E.....S..PS.N.FK.A........YR.V.TNNT-------GGVQFNKNLAGRY.NT.K..F...MG
AAV9         Q..G..S....E....Q........Q.S.E..N.....SY..S.S........I....YYL.KTIN--GS.QNQQT.KF.V.GPS...V.G..YI...SY
AAV9_PHP.B   Q..G..S....E....Q........Q.S.E..N.....SY..S.S........I....YYL.RTIN--GS.QNQQT.KF.V.GPS...V.G..YI...SY
AAV2-7m8     Q..G..S....E....Q........T.S..........SY..S.S........I....YYL.RTNT-PSGTTTQSR.QF.Q.GASDIRD.S.......CY
AAV2         Q..G..S....E....Q........T.S..........SY..S.S........I....YYL.RTNT-PSGTTTQSR.QF.Q.GASDIRD.S.......CY
AAV2-HSPG    Q..G..S....E....Q........T.S..........SY..S.S........I....YYL.RTNT-PSGTTTQSR.QF.Q.GASDIRD.S.......CY
AAV7         QS.G..S....E....Q..........S.S........SY..S.S........I....YYLARTQSNPGGTAGN.E.QFYQGGPST..E.AK......C.
Rh36         QS.G..S....E....Q..........S.S........SY..S.S........I....YYLARTQSTTGS---T.E.QFHQ.GPNT..E.SK......CY
AAV6.2       Q..G..S....E....Q........T.S..........SY..S.S........I....YYLNRTQNQ-SGSAQNKD.LF..GSPAG.SV.PK......CY
AAV1         Q..G..S....E....Q........T.S....E.....SY..S.S........I....YYLNRTQNQ-SGSAQNKD.LF..GSPAG.SV.PK......CY
AAV3         Q..G..S....E....Q........Q.S..........SY..S.S........I....YYLNRTQGTTSGTTNQSR.LF.Q.GPQS.SL.A.......CY
AAV8         Q..G..S....E....Q........Q............SY..S.S........I....YYL.RTQT-TGGTANTQT.GF.QGGPNT..N.AK......CY
Rh10         Q..G..S....E....Q..........S.Q........SY..S.S........I....YYL.RTQS-TGGTAGTQQ.LF.Q.GPN..S..AK......CY
Rh61         Q..G..S....E....Q........S.S.P........SY..S.S........I....YYL.RTQS-TGGTAGTQQ.LF.Q.GPS..S..A.......CY
Rh8          Q.LG..S....E....Q........Q.S..........SY..S.S........I....YYLVRTQT--TGTGGTQT.AF.Q.GPSS..N.A...V...CY
Anc80L65     Q..G..S....E....Q........Q.S..........SY..S.S........I....YYL.RTQT-TSGTAGN.T.QF.Q.GPSS..N.AK......CY
Rh32.33      NQT..N.....E....Q.........MA.N..K......Y..S.S........L.....HLQ.TTSGETLNQGNA.TTFGKIRSGDF.FYRK......CV
AAV4         QQT..N.....E....Q..........I..S..K......Y..S.S........I.....GLQ.TTTGTTLNAGTATTNFTKLRP..FSNFKK......SI

                     510       520       530       540       550       560       570       580       590       600
             ....|....|....|....|....|....|....|....|....|....|....|....|....|....|....|....|....|....|....|....|
VR-865       RDQQIFTGASNIT---KNNVFSVWEKGKQWELDNRTNLMQPGPAAATTFSGEPDRQAMQNTLAFSRTVYDQ-TTATTDRNQILITNEDEIRPTNSVGIDA
AAV5         .T.GWNL.SGVNRAS-----V.AFATTNRM..EGASYQVP.Q.NGM.NNLQGSNTY.LE..MI.NSQPANPG....YLEGNM...S.S.TQ.V.R.AYNV
AAV9         .Q.RVS.TVTQNNNS-----EFA.PGASS.A.NG.NS..N....M.SHKE..DRFFPLSGS.I.GKQGTGR---DNV.ADKVM....E..KT..P.ATES
AAV9_PHP.B   .Q.RVS.TVTQNNNS-----EFA.PGASS.A.NG.NS..N....M.SHKE..DRFFPLSGS.I.GKQGTGR---DNV.ADKVM....E..KT..P.ATES
AAV2-7m8     .Q.RVSKTSADNNNS-----EYS.TGATKYH.NG.DS.VN....M.SHKDD.EKFFPQSGV.I.GKQGSEK---TNV.IEKVM..D.E...T..P.ATEQ
AAV2         .Q.RVSKTSADNNNS-----EYS.TGATKYH.NG.DS.VN....M.SHKDD.EKFFPQSGV.I.GKQGSEK---TNV.IEKVM..D.E...T..P.ATEQ
AAV2-HSPG    .Q.RVSKTSADNNNS-----EYS.TGATKYH.NG.DS.VN....M.SHKDD.EKFFPQSGV.I.GKQGSEK---TNV.IEKVM..D.E...T..P.ATEQ
AAV7         .Q.RVSKTLDQNNNS-----NFA.TGATKYH.NG.NS.VN..V.M..HKDD.DRFFPSSGV.I.GK.GATN----K.TLENV.M...E......P.ATEE
Rh36         .Q.RLSKNLDFNNNS-----NFA.TAATKYH.NG.NS.TN..IPM..NKDD.DQFFPINGV.V.GK.GAAN----K.TLENV.M.S.E..KT..P.ATEE
AAV6.2       .Q.RVSKTKTDNNNS-----NFT.TGASKYN.NG.ESIIN..T.M.SHKDDKDKFFP.SGVMI.GKESAGA---SN.ALDNVM..D.E..KA..P.ATER
AAV1         .Q.RVSKTKTDNNNS-----NFT.TGASKYN.NG.ESIIN..T.M.SHKDD.DKFFP.SGVMI.GKESAGA---SN.ALDNVM..D.E..KA..P.ATER
AAV3         .Q.RLSKT.NDNNNS-----NFP.TAASKYH.NG.DS.VN....M.SHKDD.EKFFP.HGN.I.GKEGTTA---SNAELDNVM..D.E...T..P.ATEQ
AAV8         .Q.RVS.TTGQNNNS-----NFA.TA.TKYH.NG.NS.AN..I.M..HKDD.ERFFPSNGI.I.GKQNAAR---DNA.YSDVML.S.E..KT..P.ATEE
Rh10         .Q.RVS.TL.QNNNS-----NFA.TGATKYH.NG.DS.VN..V.M..HKDD.ERFFPSSGV.M.GKQGAGK---DNV.YSSVML.S.E..KT..P.ATEQ
Rh61         .Q.RVS.TL.QNNNS-----NFA.TGATKYH.NG.DS.VN..V.M..NKDD.DRFFPSSGI.M.GKQGAGK---DNV.YSNVML.S.E..KT..P.ATEQ
Rh8          .Q.RVS.TTNQNNNS-----NFA.TGAAKFK.NG.DS..N..V.M.SHKDDDDRFFPSSGV.I.GKQGAGN---DGV.YS.V...D.E..KA..P.ATEE
Anc80L65     .Q.RVSKTTNQNNNS-----NFA.TGATKYH.NG.DS.VN....M..HKDD.DKFFP.SGV.I.GKQGAGN---SNV.LDNVM....E..KT..P.ATEE
Rh32.33      KQ.RFSKT..QNYKIPASGGNALLKYDTHYT.N..WSNIA...PM..AGPSDG.FS-NAQLIFPGPS.TGN---T..SA.NL.F.S.E..AA..PRDT.M
AAV4         KQ.GFSKT.NQNYKIPATGSD.LIKYETHST..G.WSALT...PM..AGPADSKFS-NSQLIFAGPKQNGN---TA.VPGTLIF.S.E.LAA..ATDT.M

                     610       620       630       640       650       660       670       680       690       700
             ....|....|....|....|....|....|....|....|....|....|....|....|....|....|....|....|....|....|....|....|
VR-865       WGAVPTNNQSIVTP----------GTRAAVNNQGALPGMVWQNRDIYPTGTHLAKIPDTDNHFHPSPLIGRFGCKHPPPQIFIKNTPVPANPSETFQTAK
AAV5         G.QMA.....ST.A----------PATGTY.L.EIV..S..ME..V.LQ.PIW....E.GA......AM.G..L.....MML.......G.I-TS.SDVP
AAV9         Y.Q.A..H..AQ----------AQAQTGW.Q...I.......D..V.LQ.PIW....H..GN......M.G..M.......L........D.PTA.NKD.
AAV9_PHP.B   Y.Q.A..H..AQ.LAV---PFKAQAQTGW.Q...I.......D..V.LQ.PIW....H..GN......M.G..M.......L........D.PTA.NKD.
AAV2-7m8     Y.S.S..L.RGNLALGETTRPARQAAT.D..T..V.......D..V.LQ.PIW....H..G.......M.G..L.......L...........T..SA..
AAV2         Y.S.S..L.RGN----------RQAAT.D..T..V.......D..V.LQ.PIW....H..G.......M.G..L.......L...........T..SA..
AAV2-HSPG    Y.S.S..L..GN----------TQAAT.D..T..V.......D..V.LQ.PIW....H..G.......M.G..L.......L...........T..SA..
AAV7         Y.I.SS.L.AAN.A----------AQTQV...............V.LQ.PIW....H..GN......M.G..L.......L..........P.V.TP..
Rh36         Y.V.SS.L..STAG----------PQSQTI.S............V.LQ.PIW....H..GN......M.G..L.......L..........P.V.TP..
AAV6.2       F.T.AV.L..SS.D----------PATGD.HVM.........D..V.LQ.PIW....H..G.......M.G..L.......L..........PAE.SAT.
AAV1         F.T.AV.F..SS.D----------PATGD.HAM.........D..V.LQ.PIW....H..G.......M.G..L.N.....L..........PAE.SAT.
AAV3         Y.T.AN.L..SN.A----------P.TGT..H..........D..V.LQ.PIW....H..G.......M.G..L.......M..........PT..SP..
AAV8         Y.I.AD.L.QQN.A----------PQIGT..S............V.LQ.PIW....H..GN......M.G..L.......L........D.PT..NQS.
Rh10         Y.V.AD.L.QQNAA----------PIVG...S............V.LQ.PIW....H..GN......M.G..L.......L........D.PT..SQ..
Rh61         Y.V.AD.L.QQD.A----------PIVG...S............V.LQ.PIW....H..GN......M.G..L......VL........D.PTA.NQ..
Rh8          Y...AI...AAN.Q----------AQTGL.H...VI........V.LQ.PIW....H..GN......M.G..L.......L........D.PL..NQ..
Anc80L65     Y.T.A..L..AN.A----------PATGT..S..........D..V.LQ.PIW....H..G.......M.G..L.......L..........PT..SP..
Rh32.33      F.QIAD...NAT.A----------PITGN.TAM.V...........YQ.PIW....HA.G.........G..L.................AT..TA.R
AAV4         ..NL.GGD..NSNL----------P.VDRLTAL..V..........YQ.PIW....H..G.........G..L.................AT..SSTP

                     710       720       730       740       750       760       770
             ....|....|....|....|....|....|....|....|....|....|....|....|....|....|
VR-865       VASFINQYSTGQCTVEIFWELKKETSKRWNPEIQFTSNFGNAADIQFAVSDTGSYSEPRPIGTRYLTKPL
AAV5         .S...T......V...ME......N.........Y.N.YNDPQFVD..PDS..E.RTT.........R..
AAV9         LN...T......VS...E...Q..N.........Y...YYKSNNVE...NTE.V.............RN.
AAV9_PHP.B   LN...T......VS...E...Q..N.........Y...YYKSNNVE...NTE.V.............RN.
AAV2-7m8     F....T......VS...E...Q..N.........Y...YNKSINVD.T.DTN.V.............RN.
AAV2         F....T......VS...E...Q..N.........Y...YNKSVNVD.T.DTN.V.............RN.
AAV2-HSPG    F....T......VS...E...Q..N.........Y...YNKSVNVD.T.DTN.V.............RN.
AAV7         F....T......VS...E...Q..N.........Y....EKQTGVD...DSQ.V.............RN.
Rh36         F....T......VS...E...Q..N.........Y...YAKSNNVE...NPD.V.T...........RN.
AAV6.2       F....T......VS...E...Q..N.......V.Y...YAKS.NVD.T.DNN.L.T...........R..
AAV1         F....T......VS...E...Q..N.......V.Y...YAKS.NVD.T.DNN.L.T...........R..
AAV3         F....T......VS...E...Q..N.........Y...YNKSVNVD.T.DTN.V.............RN.
AAV8         LN...T......VS...E...Q..N.........Y...YYKSTSVD...NTE.V.............RN.
Rh10         L....T......VS...E...Q..N.........Y...YYKSTNVD...NTD.T.............RN.
Rh61         LN...T......VS...E...Q..N.........Y...YYKSTNVD...NTE.V.............RN.
Rh8          LN...T......VS...E...Q..N.........Y...YYKSTNVD...NTE.V.............RN.
Anc80L65     F....T......VS...E...Q..N.........Y...YNKSTNVD...DTN.V.............RN.
Rh32.33      .D...T......VA.Q.E..IE..R.......V.....Y..QSSMLW.PDT..K.T...V..S....NH.
AAV4         .N...T......VS.Q.D..IQ..R.......V.....Y.QQNSLLW.PDAA.K.T...A.......HH.
```

Figure S10 continued

Seq->	VR-865	AAV5	AAV9	AAV9_PHP.B	AAV2-7m8	AAV2	AAV2-HSPG	AAV7	Rh36	AAV6.2	AAV1	AAV3	AAV8	Rh10	Rh61	Rh8	Anc80L65	Rh32.33	AAV4
VR-865	ID	0.518	0.562	0.56	0.551	0.557	0.558	0.567	0.558	0.554	0.553	0.561	0.554	0.561	0.558	0.559	0.574	0.532	0.521
AAV5	0.518	ID	0.56	0.557	0.561	0.566	0.567	0.571	0.572	0.569	0.569	0.571	0.569	0.563	0.562	0.564	0.589	0.52	0.512
AAV9	0.562	0.56	ID	0.989	0.801	0.814	0.815	0.815	0.8	0.817	0.817	0.829	0.85	0.851	0.856	0.867	0.855	0.625	0.613
AAV9_PHP.B	0.56	0.557	0.989	ID	0.803	0.807	0.809	0.812	0.796	0.814	0.814	0.826	0.846	0.846	0.853	0.864	0.852	0.622	0.61
AAV2-7m8	0.551	0.561	0.801	0.803	ID	0.983	0.981	0.812	0.792	0.82	0.817	0.859	0.816	0.828	0.819	0.827	0.863	0.617	0.591
AAV2	0.557	0.566	0.814	0.807	0.983	ID	0.997	0.821	0.802	0.83	0.828	0.869	0.825	0.837	0.828	0.837	0.872	0.621	0.596
AAV2-HSPG	0.558	0.567	0.815	0.809	0.981	0.997	ID	0.821	0.804	0.832	0.829	0.871	0.825	0.837	0.828	0.837	0.874	0.621	0.597
AAV7	0.567	0.571	0.815	0.812	0.812	0.821	0.821	ID	0.906	0.852	0.849	0.842	0.879	0.886	0.887	0.871	0.907	0.66	0.627
Rh36	0.558	0.572	0.8	0.796	0.792	0.802	0.804	0.906	ID	0.844	0.841	0.833	0.86	0.866	0.861	0.837	0.878	0.653	0.627
AAV6.2	0.554	0.569	0.817	0.814	0.82	0.83	0.832	0.852	0.844	ID	0.993	0.865	0.84	0.849	0.843	0.857	0.894	0.659	0.625
AAV1	0.553	0.569	0.817	0.814	0.817	0.828	0.829	0.849	0.841	0.993	ID	0.862	0.837	0.847	0.84	0.854	0.891	0.659	0.625
AAV3	0.561	0.571	0.829	0.826	0.859	0.869	0.871	0.842	0.833	0.865	0.862	ID	0.851	0.851	0.849	0.848	0.899	0.633	0.616
AAV8	0.554	0.569	0.85	0.846	0.816	0.825	0.825	0.879	0.86	0.84	0.837	0.851	ID	0.934	0.936	0.906	0.907	0.646	0.623
Rh10	0.561	0.563	0.851	0.846	0.828	0.837	0.837	0.886	0.866	0.849	0.847	0.851	0.934	ID	0.97	0.907	0.913	0.654	0.627
Rh61	0.558	0.562	0.856	0.853	0.819	0.828	0.828	0.887	0.861	0.843	0.84	0.849	0.936	0.97	ID	0.906	0.909	0.65	0.621
Rh8	0.559	0.564	0.867	0.864	0.827	0.837	0.837	0.871	0.837	0.857	0.854	0.848	0.906	0.907	0.906	ID	0.913	0.656	0.623
Anc80L65	0.574	0.589	0.855	0.852	0.863	0.872	0.874	0.907	0.878	0.894	0.891	0.899	0.907	0.913	0.909	0.913	ID	0.672	0.641
Rh32.33	0.532	0.52	0.625	0.622	0.617	0.621	0.621	0.66	0.653	0.659	0.659	0.633	0.646	0.654	0.65	0.656	0.672	ID	0.814
AAV4	0.521	0.512	0.613	0.61	0.591	0.596	0.597	0.627	0.627	0.625	0.625	0.616	0.623	0.627	0.621	0.623	0.641	0.814	ID

Figure S11. Sequence ID of AAVs depicted in Fig. 1B. Rows and Columns are different capsids and the cells represent the % identity (amino acid of course) between the two proteins.

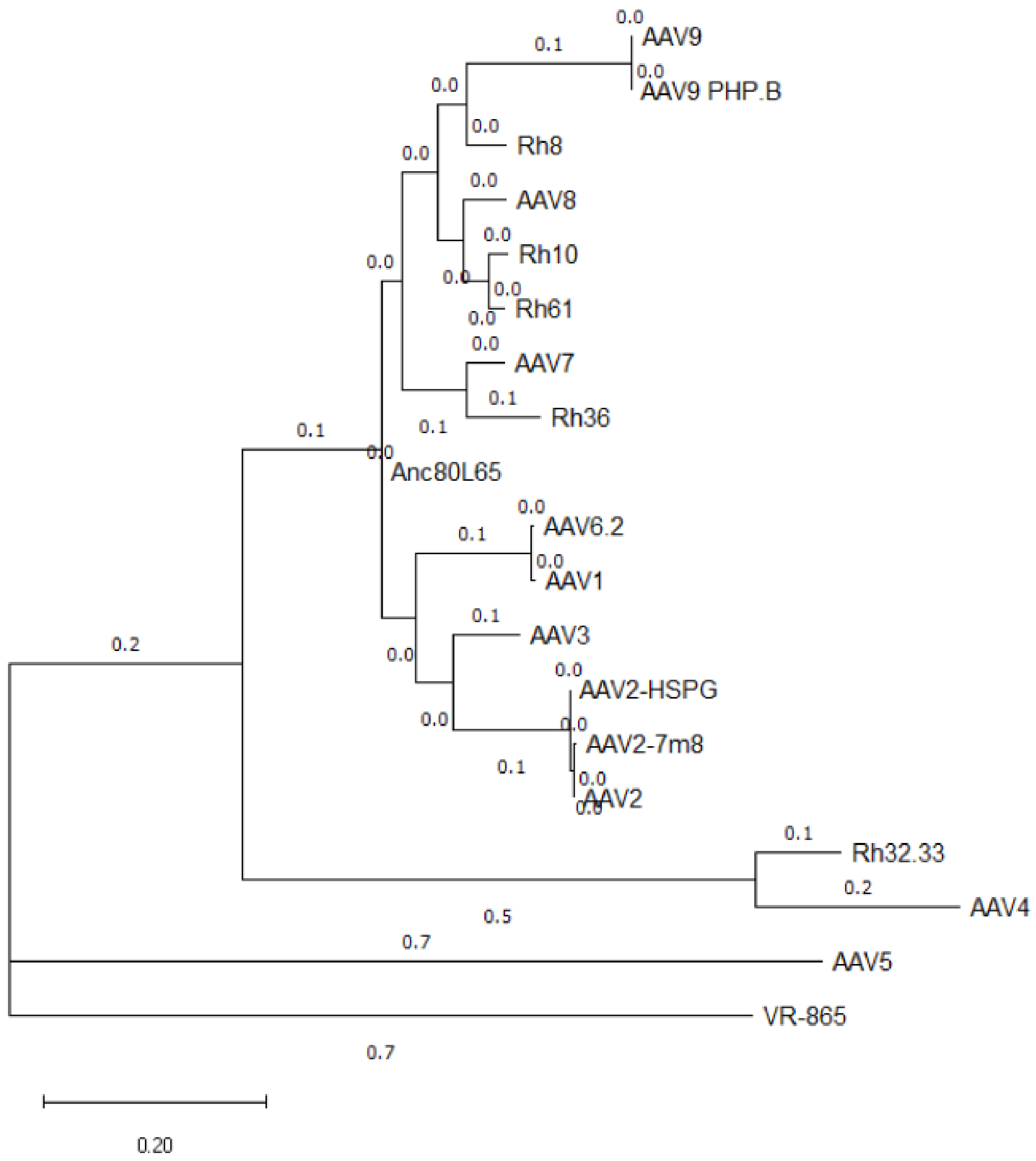

Figure S12. Newick formatted phylogeny of the phylogenetic tree depicted on Fig. 1B.

Supplemental Protocol: Production of AAV using hyperflasks and AAVX affinity chromatography

- For 1-4 hyperflask, the protocol takes approximately 10-14 days.
- We recommend ordering hyperflasks 2-3 months in advance, as they are commonly heavily backordered.
- All DNA used for transfection needs to be endotoxin free; we recommend Qiagen Endo Free kits or outsourcing to endo free companies (PureSyn); we DO NOT recommend using Zymo Endo Free kits due to their high variability in performance.
- Transgene plasmid needs to contain intact ITRs: we recommend testing ITR integrity with SmaI/XmaI digests and/or next generation sequencing for each DNA prep.
- For buffer exchange, we recommend use of Amicon Stirred Cell concentrators, particularly for purification of PHP.B and related variants, which tend to strongly sediment. Buffer exchange using Amicon Stirred Cell results in more consistently high recovery and low sedimentation. However Amicon Stirred Cell is more time-consuming, not disposable and requires an upfront investment to set up the system; therefore use of Amicon Ultra 15 Centrifugal filter devices can be used with low titer preps (<$1x10^{13}$), given careful handling (see *Buffer exchange and titration*).

Required items (per hyperflask):

Approximately 1 month prior to the start of AAV production, ensure you have all the required reagents (below). Order any that are missing and produce DNA with endo-toxin free kits.

Tissue culture and transfection:

- Cap DNA: 130 µg
- deltaF6 or other packaging plasmid: 260 µg (UPenn Vector Core)
- Transgene DNA: 130 µg
- 1L sterile filtered DMEM high glucose with 10% FBS, 1% Penstrep (11965118 Thermo Fisher, 15-140-122 Fisher Scientific)
- 600mL of filtered DMEM high glucose with 1% Penstrep **without FBS**
- Low passage HEK293T cells
- 5x 15cm tissue culture dishes
- 1x hyperflask (any variant is fine) (CLS10031-4EA Millipore Sigma or others)
- 715 µg PEIMax (1mg/ml, pH 2.6, sterile filtered) (24765-1 Polysciences Inc.)

Lysis and clarification:

- 2x 1L bottle-top PES 0.45µm filters (1143-RLS, Foxx Life Sciences)
- 3ml Triton-X 100 (T8787-100ML, Sigma)
- 2.5 mg RNAse A (1mg/ml concentration)
- 56 µl of Turbonuclease (at 250U/µl starting concentration, to a final of 25U/mL in the media) (ACGC80008, Vita Scientific)
- 56 µl of 10% Pluronic F68 (final concentration 0.001%)
- A centrifuge that can spin 2x 500ml tubes at 4000g or more (or spread the media across more tubes)
- 2x 500 ml centrifuge tubes

HPLC:

- POROS CaptureSelect AAVX resin (prepacked or free): (A36739 Thermo Fisher, A36652 Thermo Fisher)
- AKTA Pure 25 HPLC system with sample pump or equivalent. Housed at room temperature.

HPLC buffers:

- A1: 2L 1x TBS (28358 Thermo Fisher)
- A2: 1L 20%EtOH-1x TBS
- A3: 1L 2X TBS
- A4: 500mL 6M Guanidine (SRE0066-100ML, Sigma Aldrich or make from G3272-2KG Sigma Aldrich in distilled water)
- A5: 1L 20% EtOH
- A6: 500mL Phosphoric acid 0.1M, pH1 (PX0996-6 Sigma Aldrich)
- B1: 500mL 0.2M Glycine, pH 2 to pH 2.5, 0.01% vol/vol Pluronic F68 elution buffer
- 1M Tris pH8, 0.1% vol/vol Pluronic F68 neutralization buffer
- 20 x15mL tubes
- 1L 0.1M NaOH
- 1L H2O
- 1L EtOH 20%
- 0.1M and 1M NaCl for packing and column qualification
- Final Formulation Buffer (1xPBS, 35mM NaCl and 0.001% Pluronic F68) – filter sterilize with 0.2 µm filter
- **NB! Filter all HPLC buffers using a 0.45 µm or 0.2 µm bottle top filters.** This is required to avoid introduction of sedimented salts or other particulate matter into the HPLC, which can create clogs in the flow path.
- **NB! 6M Guanidine and 0.1M Phosphoric acid are hazardous: use proper PPE and precautions in handling and disposal.**

Buffer exchange:

- 0.2 µM PES syringe filters (CLS431229-50EA, Sigma Aldrich) with 20 – 50mL syringes
- **For preps <1x10^{13}vg of AAV**: 50 or 100 kDA, Amicon Ultra 15 Centrifugal Filter devices (UFC905008 or UFC910008, EMD Millipore)
- **For preps >1x10^{13}vg of AAV**:
- Ultrafiltration Discs, 100 kDa (NMW PLHK04310 Millipore Sigma)
- 500mL of 5% hydrogen peroxide in PBS – filter sterilized. Make this fresh every time, as hydrogen peroxide activity/stability decreases in neutralized pH.
- 500mL of 70% ethanol - filter sterilized
- 500mL of Final Formulation Buffer - filter sterilized.
- Amicon Stirred Cell (UFSC05001OR) with a system for providing sterile nitrogen gas pressure, such as:
    - NI UHP80 (NITROGEN UHP GR 5.0 SIZE 80 CGA) from Airgas
    - High purity pressure regulator Y11N245D580-AG (REGULATOR FIRST STAGE HIGH PURITY 3500/100 BRP DIAMETER VALVE 1/4"C CGA580CV)
    - In line sterilizing filter Y40-LF811P (FILTER 1/2T 0.003 MICRON 750 PSIG PTFE 10R STAINLESS STEEL)

- Sterile magnetic stirrer for use under a tissue culture hood, such as Mini Stirrer (VWR 10153-304) or equivalent
- Tube fittings to connect Amicon Stirred Cell to the nitrogen source:
  - We strongly recommend contacting representatives of Airgas or Swagelok (or equivalent) to verify exact details of the tube fittings and the procedure of safely connecting and operating the nitrogen tank
    - Stainless Steel Tubing Insert, 1/4 in. OD x 0.17 in. ID (SS-405-170 Swagelok)
    - 316 Stainless Steel Front Ferrule for 1/4 in. (SS-403-1 Swagelok)
    - 316 Stainless Steel Back Ferrule for 1/4 in. (SS-404-1 – Swagelok)
    - Connect the Amicon Stirred Cell inlet tube to nitrogen outlet valve by:
      - Inserting stainless steel tubing insert into the tube.
      - Place the nut-shaped tube fitting onto the tube.
      - Place front ferrule, then back ferrule onto the tube.
      - Insert tube into the nitrogen outlet valve (which should be Swagelok pressure fitting).
      - Using a wrench, screw the nut-shaped tube fitting onto the outlet valve. The front ferrule should displace back ferrule in a manner that compresses the tube securely in place. Test that the tube is tightly secured.
      - If using the in line sterilizing filter, cut the tube to create two parts, or add an additional 1/4 in. tube, then connect to the filter as described above
      - Catalogue for further reference: https://www.swagelok.com/downloads/webcatalogs/EN/MS-01-140.PDF

Protocol

AAV production

Cell seeding

- Thaw a vial of low passage HEK293T cells, expand to four 15cm dishes, at 70-80% confluency in DMEM 10% FBS 1% PenStrep;
  - Filter sterilize all media
  - Warm all media to 37 °C before use
  - Do not let cells get more than 90% confluent at any stage during expansion, and seed cells dropwise evenly across the plates + mix gently 10x in a star pattern to ensure that cells are always evenly distributed.
  - Check that they are not confluent anywhere under microscope the next day and discard any plates that highly confluent on one side and empty on another.
  - Ideally, passage cells every 48 hours, to avoid acidification of media. 72h is still okay.
- Pool cells from four 15cm dishes into 560mL of DMEM 10% FBS 1% PenStrep, mix gently and thoroughly by pipetting to create a homogenous solution with minimal bubbling, pour into hyperflask by placing the hyperflask upright, tilted to the side as per manufacturer's instructions (https://www.youtube.com/watch?v=uo3jEQGz8Z0).
- Grow cells until they reach ~80% confluency. This commonly takes 48 hours. Check that the cells are 70-80% confluent and evenly distributed under a microscope before transfection. Transfecting 100%

confluent cells results in ~3-10x reduced AAV production levels. Transfecting 40-50% confluent cells results in a ~30-50% decrease in titers.

Transfection

- Mix 10mL DMEM (sterile filtered, room temp) with DNA (vector:cap:deltaF6 at 130μg:130μg:260μg).
- Mix 10mL DMEM (sterile filtered, room temp) with 715μg of PEIMax (1mg/ml, pH 2.6, sterile filtered).
- Mix the two solutions together, shake and vortex immediately for 15 seconds (pulse vortexing, not continuously), incubate at RT for 15 minutes.
- Add the solution to 560 ml of **DMEM-1%Penstrep** (sterile filtered, warmed to 37 C), mix thoroughly and gently with a serological pipette or swirling. **NB! Do not include serum here! Inclusion of serum in the transfection mix reduces AAV yields by 3-10x.**
- Remove the hyperflask from the incubator, pour out media carefully so as not to disturb the cells. (https://www.youtube.com/watch?v=1B_3Luum-ME)
- Gently pour the transfection mix in DMEM-1% PenStrep into the hyperflask, top up with DMEM-1% PenStrep and remove bubbles as needed.
- Put the hyperflask back into the incubator, incubate for four days. 3-5 days is also fine. Interestingly, the optimal amount of time for incubation varies between different reports, so currently there is no published consensus I am aware of. Anecdotally, 3-5 days results in similar yields.

Harvesting and lysis

- After 3-5 days of incubation, pour media from the hyperflask into a 1L bottle.
- Add lysis reagents to the media in the bottle:
  - 3ml Triton-X 100
  - 250 μl of RNAse A (at 10mg/ml concentration, a total of 2.5 mg RNAse A)
  - 56 μl of 25U/mL of Turbonuclease
  - 56 μl of 10% Pluronic F68 (final concentration 0.001%)
- Mix with a serological pipette until the solution is clear; avoid introducing air or swirling, as it generates foam. Pour media back into the hyperflask slowly (otherwise generates foam). Store any volume that is left over in a 50mL tube.
  - The above is necessary, because adding lysis reagents directly into the hyperflask does not allow them to be rapidly uniformly distributed across cells.
- Incubate the hyperflask at 150rpm shaking for 30 min – 1 hour at 37 °C along with left-over volume in the 50mL tube. Shaking incubation aids with mechanical lysis of cells.
  - If you do not have access to a sterile shaking incubator, we recommend hand shaking for ~5 min and subsequent incubation at 37 °C or carefully double bagging the hyperflask and incubating in a non-sterile incubator, then disinfecting the outer bags with bleach and ethanol prior to proceeding.
- Decant lysate in the hyperflask into a 1L bottle, wash the hyperflask with 140 mL of PBS and add it to rest of the lysate – forming 700 mL total.
- If not proceeding to the HPLC purification on the same day, store at 4 °C (1-2 days) or -20 °C.
- Clarify the lysate (below). Clarification is critical to prevent clogging of the HPLC during the run. We recommend performing this on the day of loading onto the HPLC, to minimize re-formation of aggregates during storage.

- Divide the lysate evenly between two 500mL centrifuge tubes, centrifuge at 4000g or higher at for 30 minutes.
- Decant supernatant into 1L 0.45uM CA/PES filter, filter everything.
- Take aliquots of the clarified lysate for qPCR/ddPCR titration, store aliquots at -20°C…-80°C. Titer these aliquots along with your final purified AAV later. This provides very helpful information in troubleshooting, allowing you to determine your purification efficiency, acting as a sanity check (i.e the amount of purified AAV cannot be greater than the amount of AAV in input lysate, accounting for the accuracy of your qPCR/ddPCR titration) and in the case of low yields allows you to pinpoint whether the failure was at transfection or purification.

HPLC purification and buffer exchange

A full introduction into the usage and theory of HPLC is out of the scope of this protocol. The below is intended for users with basic training and capacity to operate HPLC machines and is based on the Akta Pure 25 system using pre-packed 1mL AAVX columns. Regardless of the machine used, the below parameters are critical for efficient purification:

- **Purification is carried out at room temperature.** Purification at cold temperatures substantially decreases binding efficiencies of the resin (Fig. 2D). For this purpose, both the clarified lysate containing AAV and the HPLC machine need to be brought to room temperature prior to purification. If the HPLC machine is housed in a fridge, we recommend not running the machine inside the fridge with cooling turned off and a closed door, since this can cause considerable increase of the temperature inside the fridge. Instead, we recommend either 1) placing the machine outside the fridge, 2) running the machine with fridge turned off and door left open or 3) hooking the fridge up to an external temperature controller (such as BN-LINK Digital Cooling Thermostat Controller, Amazon) and setting the set-point to 22°C to 24°C.
- **AAVX resin binding capacity is not exceeded.** Thermo Fisher indicates a binding capacity of up to $1x10^{14}$ vg/ml which varies between serotypes. A safe rule of thumb is to use 1mL of resin per 1-2 hyperflasks, depending on the yield. When pooling AAV from multiple hyperflasks, we recommend packing your own columns with AAVX resin at a higher volume.
- **Lysate application speed does not exceed 1mL/min for a 1mL resin – i.e. the residence time is no less than 1 min.** Resin dynamic binding capacity decreases with increasing loading speed and may result in more AAV in the flow-through. **We recommend a 2min residence time, or 0.5mL/min loading speed for a 1mL resin for maximum recovery.**
- **Elution is performed in up-flow.** Elution in down-flow (or the same flow direction as lysate application) results in approximately 20% less AAV in the elution. This is most likely because a majority of AAV binds close to the inlet on the resin; therefore eluting in the opposite direction avoids AAV re-binding of the resin at the elution step.
- **Elution pH is less than 2.9, with pH 2-2.5 optimal.** AAV is acid stable at below pH 3 and will not elute above pH 2.9. Some serotypes can have strong binding affinities to the resin (such as AAV9 to the POROS AAV9 resin) and decreasing pH down to pH 2 can help increase recovery for such serotypes (Thermo Fisher – personal communication).
- **Resin regeneration is carried out with at least 15 min of 0.1M pH1 Phosphoric acid, followed by at least 15 min of 6M Guanidine.** While not a concern with small-scale preps, at large scale preps ($3x10^{13}$ … $2x10^{14}$) we commonly observed decreased binding efficiencies with resin re-use when 10 min of 6M

Guanidine only was used, particularly for PHP.eB. The AAVX resin is highly acid stable, and increasing regeneration to 15 min of 0.1M pH1 Phosphoric acid, followed 15 min of 6M Guanidine restored binding efficiencies at large scales for at least 6 runs (Fig. S3).

- **The AAVX resin is NOT stable in high pH solutions.** Accidental treatment of the resin with 0.1M NaOH will destroy the resin.

HPLC purification protocol:

- Bring the HPLC machine and the sample to room temperature
- Prepare **filtered** HPLC buffers as described in the Required Items section. **NB! 6M Guanidine and 0.1M Phosphoric acid are hazardous: use proper PPE and precautions in handling and disposal.**
- Connect the AAVX column to the machine using wet connection.
  - (Manually flow some liquid out of the inlet, connect the column inlet connector in the wet environment to avoid introducing air into the column; repeat for column outlet).
- Place buffer lines A1 to A6 and B1 into the corresponding buffers. Place Sample line (S1) and Sample Buffer line into 1x TBS.
  - Tape the lines to the buffer bottles if they do not come with weights.
  - Cover the bottles with parafilm to avoid evaporation and contamination
- Prime inlets and purge pumps (see Akta Pure user manual section 5.4, pages 160-171)
  - Prime the Sample inlet (S1) with TBS to avoid loss of your sample
- Optional but recommended: place a 1L bottle in the HPLC Outlet line to collect flow-through.
  - This is useful if for whatever reason AAV fails to bind to the resin (such as fouling, low temp, or operator error), as it allows re-purification of the prep.
- Pipette 0.11 mL of 1M Tris pH8 + 0.1% vol/vol Pluronic F68 neutralization buffer into 25 15mL tubes; place them into the fraction collector, and set the fraction collector position to 1.
- Place sample line (S1) into the AAV clarified lysate
  - Place the bottle on the machine tilted and place the S1 line at the bottom most area, to be able to collect 100% of the lysate.
- Ensure that there is sufficient volume of buffer for all buffers, and that all inlet lines are fully submerged.
- Adjust the volume, speed or other parameters of the AAVX_HPLC_S1 Akta run protocol as necessary.
  - Import the AAVX_HPLC_S1 protocol file on Akta Pure. Created with Unicorn v7.1.
  - For other HPLC systems, see full overview of the run protocol below (*HPLC run method*)
- **Print out and go through the starting checklist every time before starting a new run (below).**
- **Open the and start the AAVX_HPLC_S1 protocol.**
  - Depending on the volume of lysate and run speed, the run may take anywhere between 1 hour and 36 hours. We recommend doing the calculation before-hand to pick up elution fractions soon after the run is done.
  - We recommend staying with the machine for the first 10-15 minutes in every new run to ensure that no leaks are present, that the sample inlet contains no air bubbles (which can cause pre-mature termination of loading, see *Troubleshooting*), and that sample application reaches a steady plateau.
  - See *Troubleshooting* for examples of successful and unsuccessful runs

- After the run is complete, remove the elution fractions containing AAV, store them at 4°C for short term or -20°C for long term.
- Multiple preps can be automatically purified back-to-back by copying the protocol, changing sample input to S2..S7, saving the new protocols, and starting them during the run of the first protocol. This adds them to the run cue, and the machine will automatically continue to these protocols after the previous ones are complete.
    - Note that purification of multiple preps back-to-back removes the ability to collect flow-through with the Outlet tube, as the machine unfortunately contains only a single outlet line.
    - In this case, place the Outlet tube into waste bin to avoid overflowing the collection bottle.
- After the last run, Remove the AAVX column, cap the tubes and store it at 4°C
- When the machine will not be expected to be used for longer than a week, perform System CIP (cleaning in place):
    - Place all inlets used in the run into 1L 0.1M NaOH
    - Start the System CIP protocol or manually run 20 mL liquid through all lines
    - Repeat for H_20 and 20% ethanol
    - Store all lines in 20% ethanol

Buffer exchange

- Before pipetting AAV containing solutions, we recommend coating all pipette tips and serologicals with Final Formulation Buffer (1xPBS, 35mM NaCl and 0.001% Pluronic F68) or another Pluronic F68 containing buffer to minimize AAV binding to plastic. We also recommend usage of low-retention tips if available.
- Thaw elution fractions if frozen previously.
- From here on, work in sterile conditions.

Protocol for buffer exchange using Amicon Stirred Cell

- While fractions are thawing, place 50mL tubes on a rack in a TC hood and remove caps.
- Attach 0.2 µm PES filter disks on top of the tubes by wrapping parafilm around the edge of the filter tightly.
- Remove plungers from 50mL syringes, and insert the syringes into the filter disks.
- Pipette 2mL of Final Formulation Buffer (1xPBS, 35mM NaCl and 0.001% Pluronic F68) right onto the filter inlet, ensuring the buffer wets the filter.
- Incubate for 15 min or more.
- Assemble Amicon Stirred Cell manifold with the 100 kDa ultrafiltration disk at the bottom, glossy side facing upwards, inside the TC hood.
    - See User guide here https://www.merckmillipore.com/FI/en/product/Amicon-Stirred-Cell-50mL,MM_NF-UFSC05001 and here: https://www.merckmillipore.com/FI/en/product/Ultrafiltration-Discs-100kDa-NMW,MM_NF-PLHK04310?bd=1#anchor_BRO
    - Before first use, we recommend sterilizing the manifold and the filter disk. The filter disk can be sterilized in 70% ethanol for 5 minutes. For manifold sterilization, see Decontaminate and clean at the end of this section.
- Place the Amicon on a magnetic stirrer and connect to the nitrogen tank.

    - Tape the tube down if it does not stay in place, to keep the manifold on the magnetic stirrer.
- Remove the top of the manifold and pour 50mL of Final Formulation Buffer into the Amicon manifold.
- Incubate for 15 min or more.
- Open the nitrogen flow to pressurize the system.
    - Slowly open the nitrogen tank first, then slowly open the course stage regulator on the right, finally slowly open the fine stage regulator to allow nitrogen flow into the manifold. Always follow manufacturer safety instructions.
    - Be mindful to not exceed Amicon and filtration membrane maximum pressure limits – 75 psi and 70 psi respectively.
    - Turn on magnetic stirrer at low speed to check the functioning of the entire system.
    - Turn off nitrogen flow from fine stage regulator when approximately 2-3 mL of liquid is left.
    - **NB! Turning off nitrogen flow does not immediately eliminate pressure from the manifold! To immediately eliminate pressure, slowly open the blue valve on the manifold cap.**

- Once elution fractions are thawed, centrifuge tubes containing elution fractions briefly to spin down the liquid.
- Sterilize tubes with 70% ethanol and bring them to the tissue culture hood.
- Pool all elution fractions containing AAV into a single 50mL tube.
    - Coat all pipette tips with sterile Final Formulation Buffer before aspirating AAV.
    - Before aspirating liquid from a fraction, mix it by pipetting briefly to ensure there is no concentrated layer of AAV that remains at the bottom.
- Bring the volume up to 48mL with Final Formulation Buffer and mix.
- Pour or pipette the volume into the 50mL syringe filter, insert plunger and filter through the disk.
    - Bringing the volume up to a total of 48 mL effectively dilutes the AAV, minimizing losses at the filtration step.
- Pour or pipette the filtered solution into the previously assembled Amicon manifold.
- Seal the Amicon cap, turn on the magnetic stirrer at low to medium speed, and open nitrogen flow to pressurize the system.
    - Adjust the nitrogen pressure to provide continuous but not too rapid filtration. The filtration should take 5 minutes or more to completion. Rapid filtration results in high local densities of AAV on the filter surface, which leads to AAV aggregate formation and subsequent loss of titer and/or bioactivity. The stirring action by magnetic stir bar mitigates this substantially but would be reduced by very rapid filtration.
    - Filtration speed is proportional to the concentration of AAV (and other molecules above 100kDA). Thus, more concentrated preps will require longer time to filter – up to 15-20 minutes in our hands.
- Stop the filtration once 2-5mL of liquid is left.
    - Turn off the nitrogen gas, then slowly and gently lift the blue valve to de-pressurize
    - **NB. Do not allow filtration to proceed to overconcentration at this step – this can cause AAV sedimentation and loss.** Always aim to stop the filtration before 1mL of liquid is left.
- Remove the Amicon cap, pour in Final Formulation Buffer to 50mL, repeat filtration
- Repeat filtration until a total of >1000x dilution is achieved. This normally takes 2-3 filtration cycles.
    - When leaving 5mL of filtrate left, adding 45mL of Final Formulation buffer results in 10x dilution. In this case 1000x dilution is achieved in three cycles.

- This can also be achieved in two steps if starting with <5mL of volume of elution fractions (approximately 10x dilution at the filtration step) and repeating the buffer exchange twice with >10x dilution.

- At the last filtration cycle, to obtain an accurate desired volume of final AAV, carefully observe the liquid level and stop and de-pressurize the system at 5mL.
- Stop the stirrer and estimate liquid volume by eye or by measuring with a serological pipette.
- Remove the waste tube (keeping the tube connector attached to the manifold) and place a 5mL tube underneath the waste outlet.
- Continue filtration to the desired amount with stir bar turned on low, by estimating remaining volume in the Amicon manifold through observing the volume of waste in the 5mL tube.
    - This is required because the Amicon manifold is flat and accurate volume estimations at less than 2mL are difficult. By estimating starting volume and volume in waste, an accurate estimation of volume left in the Amicon manifold can be made.
    - Removal of the waste tube is necessary because it contributes to dead volume (approximately 2mL), which can be filled with air to various degree, making accurate waste volume estimation difficult.
- Alternatively, achieve desired final AAV volume by stopping the filtration at various points and measuring left-over volume with a pipette or serological.
- The solution can accurately be concentrated to a few hundred microliters this way. However, we recommend keeping AAV at the highest possible volume allowed by downstream experimental requirements, particularly for PHP.B and its derivatives, as high concentrations of AAV will aggregate more readily during freeze-thaw cycles (Wright JF et al. Mol Ther. 2005 Jul;12(1):171-8.).
- Depressurize Amicon, remove stir bar and aspirate AAV into a new 1.5mL or 2mL tube.
    - Optionally wash the filter membrane with additional 100 µL of Final Formulation Buffer and add to AAV.
- Measure the volume of final AAV solution with a P1000 pipette or serological.
    - For P1000, turn the pipette down to low volume, aspirate, then turn the pipette to higher volumes while the tip is submerged in the solution. When the solution is fully aspirated this way, the volume can be read from the pipette.
- Record the volume or bring up to a desired volume with Final Formulation Buffer.
- Take and store a 15 µl aliquot for titration
    - Take a higher volume if further tests, such as protein electrophoresis are performed.
    - This is to avoid unnecessarily thawing AAV designated for experimental applications.
    - Do not open AAV tubes meant for experimental applications under non-sterile conditions.
- Aliquot AAV into separate smaller aliquots unless the full amount is expected to be used in a single experiment.
- Store AAV and the titration aliquot (if not immediately used for titration) at -80 °C.
- Decontaminate and clean the Amicon manifold between concentration of different AAV preps, and at the end:
    - Disconnect the Amicon manifold from the nitrogen source
    - Disassemble the manifold, and place all parts into a 2L beaker filled with 500mL of 5% hydrogen peroxide
    - Incubate with slight shaking for 5 minutes
    - Repeat with 70% ethanol and Final Formulation Buffer
    - Dry before storage or concentration of a new prep

- Alternatively, based on manufacturer's datasheet, Amicon Stirred Cells are compatible with standard sterilizing gas mixtures or can be autoclaved for at least 10 cycles at 121 °C, 1 bar (250 °F, 15 psi) for 30 minutes.

Protocol for buffer exchange using Amicon Ultra 15 Centrifuge devices

- **NB. Amicon Ultra 15 Centrifuge devices and their equivalents from competitors do NOT come sterilized.** We recommend sterilization with sterilizing gases. If these cannot be used, 70% ethanol or UV can be attempted, but these may adversely affect the membrane (depending on the membrane type) and are likely of low efficacy.
- While fractions are thawing, place the Amicon Ultra 15 tubes on a rack in a TC hood and remove caps.
- Pipette 2mL of Final Formulation buffer onto the membrane of the Amicon tubes.
- Attach 0.2 µm PES filter disks on top of the tubes by wrapping parafilm around the edge of the filter tightly.
- Remove plungers from 20mL syringes and insert the syringes into the filter disks.
- Pipette 2mL of Final Formulation Buffer right onto the filter inlet, ensuring the buffer wets the filter.
- Incubate for 15 min or more.
- Once elution fractions are thawed, centrifuge tubes containing elution fractions briefly to spin down the liquid.
- Sterilize tubes with 70% ethanol and bring them to the tissue culture hood.
- Pool all elution fractions containing AAV into a single 15-50mL tube.
    - Coat all pipette tips with sterile Final Formulation Buffer before aspirating AAV.
    - Before aspirating liquid from a fraction, mix it by pipetting briefly to ensure there is no concentrated layer of AAV that remains at the bottom.
- Bring the volume up to 10mL with Final Formulation Buffer and mix.
- Pour or pipette the volume into the 20mL syringe filter, insert plunger and filter through the disk.
    - Bringing the volume up to max volume dilutes the AAV, minimizing losses at the filtration step.
- Cap the Amicon tubes and centrifuge to concentrate:
    - Continuous centrifugation will result in high local density of AAV at the filter membrane, causing aggregation and sedimentation out of the solution, which decreases titers and bioactivity.
    - We recommend centrifuging for 1-2 minutes, removing the tubes from the centrifuge, opening them under the TC hood and mixing/washing the membrane with a P1000. This substantially reduces AAV aggregation although does not eliminate it for high concentration preps.
    - **NB. Aim to not concentrate below 1mL, as this increases AAV aggregation.**
    - **NB. Do not centrifuge at speeds higher than 5000g!**
- Concentrate to 1mL.
- Decontaminate and bring the Amicon into a TC hood.
- Fill the tube with Final Formation Buffer to 14 ml and mix.
- Repeat until a total of >1000x dilution and desired final volume is achieved.
    - The solution can be concentrated to a few hundred microliters. However, we recommend keeping AAV at the highest possible volume allowed by downstream experimental requirements, particularly for PHP.B and its derivatives, as high concentrations of AAV will

aggregate more readily during buffer exchange as well as freeze-thaw cycles (Wright JF et al. Mol Ther. 2005 Jul;12(1):171-8.).

- Measure the volume of final AAV solution with a P1000 pipette or serological.
    - For P1000, turn the pipette down to low volume, aspirate, then turn the pipette to higher volumes while the tip is submerged in the solution. When the solution is fully aspirated this way, the volume can be read from the pipette.
- Record the volume or bring up to a desired volume with Final Formulation Buffer.
- Take and store a 15 µl aliquot for titration.
    - Take a higher volume if further tests, such as protein electrophoresis are performed.
    - This is to avoid unnecessarily thawing AAV designated for experimental applications.
    - Do not open AAV tubes meant for experimental applications under non-sterile conditions.
- Aliquot AAV into separate smaller aliquots unless the full amount is expected to be used in a single experiment.
- Freeze at -80C.

Titration

A thorough and detailed protocol for AAV titration using qPCR or ddPCR is described in Sanmiguel, J., Gao, G., & Vandenberghe, L. H. (2019). Quantitative and Digital Droplet-Based AAV Genome Titration. Adeno-Associated Virus Vectors, 51–83. doi:10.1007/978-1-4939-9139-6_4. We recommend performing AAV titrations based these protocols.

Troubleshooting

A single hyperflask in our hands consistently yields an average of $3x10^{13}$ vg of purified AAV with well producing transgenes such as CMV-GFP for both single stranded vectors and self-complementary vectors. While some transgenes or serotypes may inherently produce at lower yields, yields below $1x10^{13}$ vg per hyperflask likely indicate a technical issue somewhere in the process.

Elution UV peak height roughly correlates with AAV yield. A peak of approximately the height (given efficient packing of the column) of the loading UV plateau for a single hyperflask generally indicates a yield of $1\text{-}3x10^{13}$ vg. Elution peaks much lower than (or with a lower area under the curve) indicate inefficient AAV production or purification. When no or very low elution peak is present, it is recommended to troubleshoot before proceeding to buffer exchange to save time and resources.

Examples:

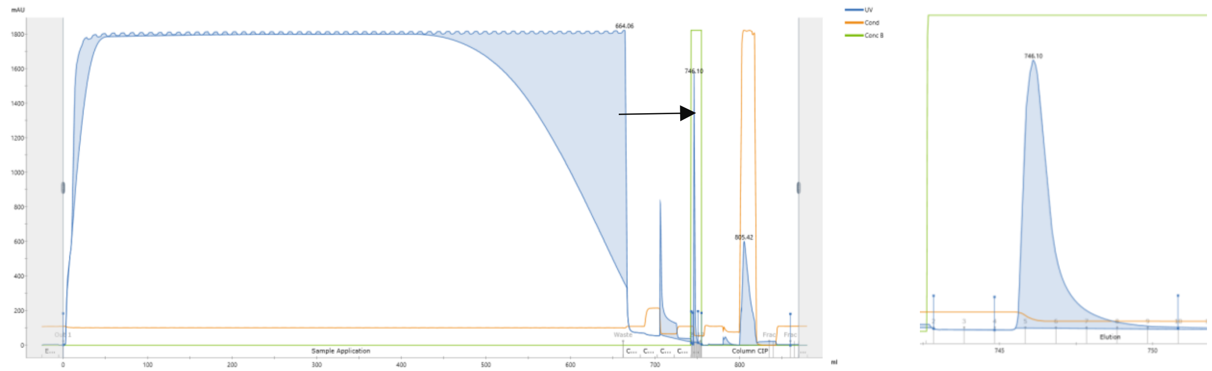

Reasonably high elution peak - efficient production and purification. Left: chromatogram of the full run; right: magnified elution UV peak.

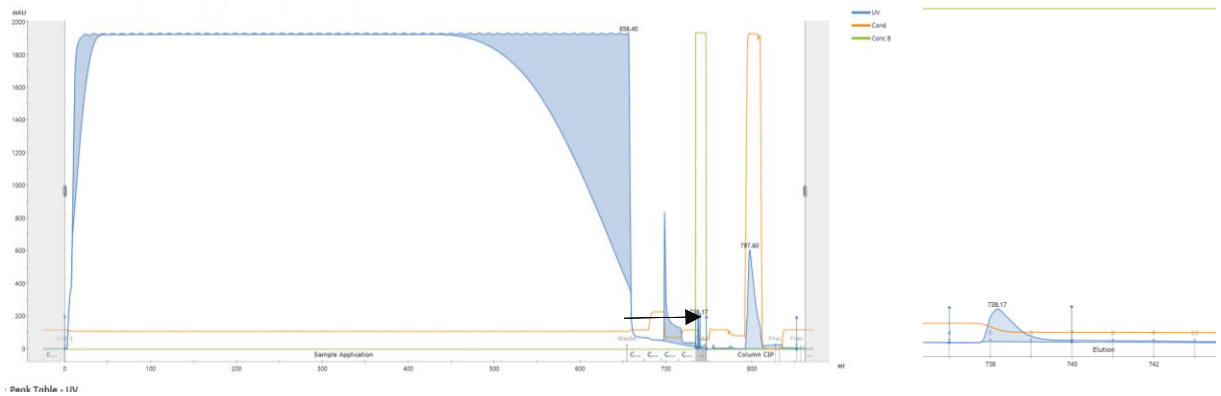

Low elution peak - inefficient production and/or purification. Left: chromatogram of the full run; right: magnified elution UV peak.

Troubleshooting

Low elution peak or low final AAV yield

- Titer aliquots taken from the pre-purification lysate, flow-through and purified AAV to identify the step at which AAV was lost.
- If pre-purification lysate contains low amounts of AAV, it is likely an AAV production issue.
- If flow-through contains high amounts of AAV, it is likely an HPLC purification issue.
- If pre-purification lysate contains high amounts of AAV and flow-through contains little AAV, it is likely a buffer exchange issue. Troubleshoot below accordingly.

Low AAV production yield

- Were HEK293T cells used?
- Were cells of low passage?
- Were cells not allowed to grow to confluency at any point during culture?
- Were cells uniformly distributed during culture?

- Was transfection performed with cells at 70-80% confluency?
- Was transfection performed with reagents at room temperature (not 4 degC)?
- Was FBS excluded from the transfection mix?
- Were all three plasmids (helper, cap and transgene) present at 260 µg:130 µg:130 µg ratios?
- Was PEI**max** used?
- Was PEImax used at the correct amount? (715 µg)
- Were all three plasmids of the correct identity?
- Did the transgene plasmid contain intact ITRs?
    - Mutated ITRs are one of the most common reasons for low yields. Mutated ITRs exist at some percent of the total population in most DNA preps. When the plasmid prep contains a high percent of mutated ITRs, yields are substantially reduced (up to 10-fold or more) and empty capsid percentage is increased. We recommend against use of such AAV preps to maintain experimental consistency and always checking for ITR integrity with SmaI/XmaI digests and/or next generation sequencing. For this reason, if it is known that a transgene will be extensively used in experimental studies, we recommend producing a Mega or Giga scale DNA prep, validating the integrity of the ITRs of this prep, aliquoting and using it for all subsequent AAV production runs.
- Was the detergent and nuclease lysis performed correctly (containing all components, and not for longer than 2 hours)?

High amounts of AAV in the flow-through and other HPLC purification issues

- Was purification carried out at room temperature?
- Was the sample brough to room temperature prior to purification?
- Was the resin/column new?
- Was the AAVX resin not allowed to dry out during storage?
- If not, was the resin/column previously regenerated with >15min pH1 Phosphoric acid and >15min 6M Guanidine?
- If so, has the resin been used more than 10 times?
    - While some data indicate that properly regenerated resins can be used for up to at least 20 times, we recommend switching to a new resin if flow-through issues emerge after roughly 10 uses.
- Did the resin get exposed to 0.1M NaOH or other strong alkaline agents?
- Is this a serotype validated to bind to AAVX or a new untested serotype?
- Clogged column:
    - Was the lysate clarified with centrifugation and filtration before loading onto the HPLC?
    - Was upflow selected during elution and column regeneration?
- Premature termination of sample loading:
    - Was the HPLC protocol set to "Inject all sample using air sensor" in the Sample Application step (on by default in the AAVX_HPLC_S1 protocol)?
    - If so, the system likely detected an air bubble in the sample line and proceeded to the following steps. We recommend purging the sample line as described in the Akta manual and repeating the purification.

    - Alternatively, Sample Application can be set to "Inject Fixed Sample Volume" – in this case sample volume must be accurately measured before to prevent underloading or loading air onto the column.
- Continuously increasing preC pressure/sample pump pressure during loading:
    - The column is being clogged. If the lysate was clarified with centrifugation and filtration before loading onto the HPLC and resin regenerated correctly, this could be a frit issue, if self-packed columns are used. We recommend replacing frits. If pre-packed column was used, it is a manufacturer issue or a column reaching the end of its lifespan. Either way, we recommend using a new column.
- Fluctuating UV line/preC line during loading:
    - There is likely an air bubble in the sample line. While purification can still work, it runs the risk of premature terminating the loading if "Inject all sample using air sensor" in the Sample Application step is selected. We recommend terminating the program, placing the Sample line into a separate tube of TBS, manually performing priming and purging, placing the line back into sample and re-starting the run.

Low final AAV yield/buffer exchange issues

- Was upflow used during HPLC elution?
    - Downflow decreases yields by approximately 20%.
- Was Pluronic F68 used in the elution buffer?
- Were elution fractions neutralized with pH8 Tris- 0.01% Pluronic F68?
- Were filters, plasticware and pipette tips coated with Final Formulation Buffer (or other Pluronic F68 containing buffer) during handling?
- Was buffer exchange carried out according to instructions, preventing overconcentration?
- AAV sedimentation (white cloudy particle formation):
    - Was buffer exchange carried out according to instructions, preventing overconcentration?
    - Some reports suggest some serotypes can be re-solubilized by gentle overnight shaking at room temperature. While most AAV serotypes are stable at room temperature for that duration, it is up to the user to decide whether this is worth trying.
- Broken Amicon Ultra 15 tubes during centrifugation:
    - Was centrifugation speed kept below 5000g?
- Low filtration speed at buffer exchange:
    - This is likely a high concentration prep, or contains additional molecules that are retained by the buffer exchange membrane. We recommend patiently continuing purification as reasonable or high yields can still be obtained. Alternatively, the sample can be diluted and split between more buffer exchange units if available.
- High filtration speed at buffer exchange:
    - The prep likely contains little to no AAV. Pure PBS filters fully in seconds. Decrease pressure or centrifugation speed.
- Liquid leakage under the cap of Amicon Ultra 15 units during centrifugation:
    - Cap overtightened or more than 14mL of media loaded onto the Amicon.

    - This is a particular problem with fixed-angle rotors. We recommend using a swinging bucket rotor if available.
- Low buffer exchange efficiencies despite no clear technical flaw:
    - Take aliquots of all purification and buffer exchange steps to determine the exact step where loss occurs.
    - For high concentration preps, splitting the prep between multiple filtration/buffer exchange units can reduce loss.

Starting checklist

-Sample filtered?
-Aliquot of sample (cleared lysate) taken?
-Column attached?
    -Correct orientation?
    -Correct resin?
-All inlets in correct buffers?
    -A1: TBS
    -A2: 20%EtOH-TBS
    -A3: 2X TBS
    -A4: 6M Guanidine
    -A5: 20% EtOH
    -A6: Phosphoric acid 0.1M, pH1
    -B1: 0.2M Glycine-0.01% Pluronic F68
    -S1: Sample 1
    -S2: Sample 2...
    -Buffer: TBS
    -Outlet in outlet tube
-Enough buffer in each tube?
-Outlet in outlet?
-All inlets primed?
-All pump heads purged?
    -Purge confirmed?
-Sample pump purged?
    -Purge confirmed?
-Fractionation:
    -Fraction collector set to position 0?
    -Enough tubes added for fractions? Apprx 20 tubes per run
    -Tris-Pluronic added to collection tubes?
-Method
    -Correct method selected?
    -Correct outlets selected?
    -Correct sample application volume selected?

-Correct fraction volumes selected?
-Correct location for save file?
-Enough volume in the sample to match method?
-Waste empty?
-Everything double-checked?

HPLC run method

For users with Akta Pure systems we highly recommend importing the AAVX_HPLC_S1 and System_CIP protocols to avoid unwanted errors. The below is intended as a complete specification of run parameters for users of other HPLC systems.

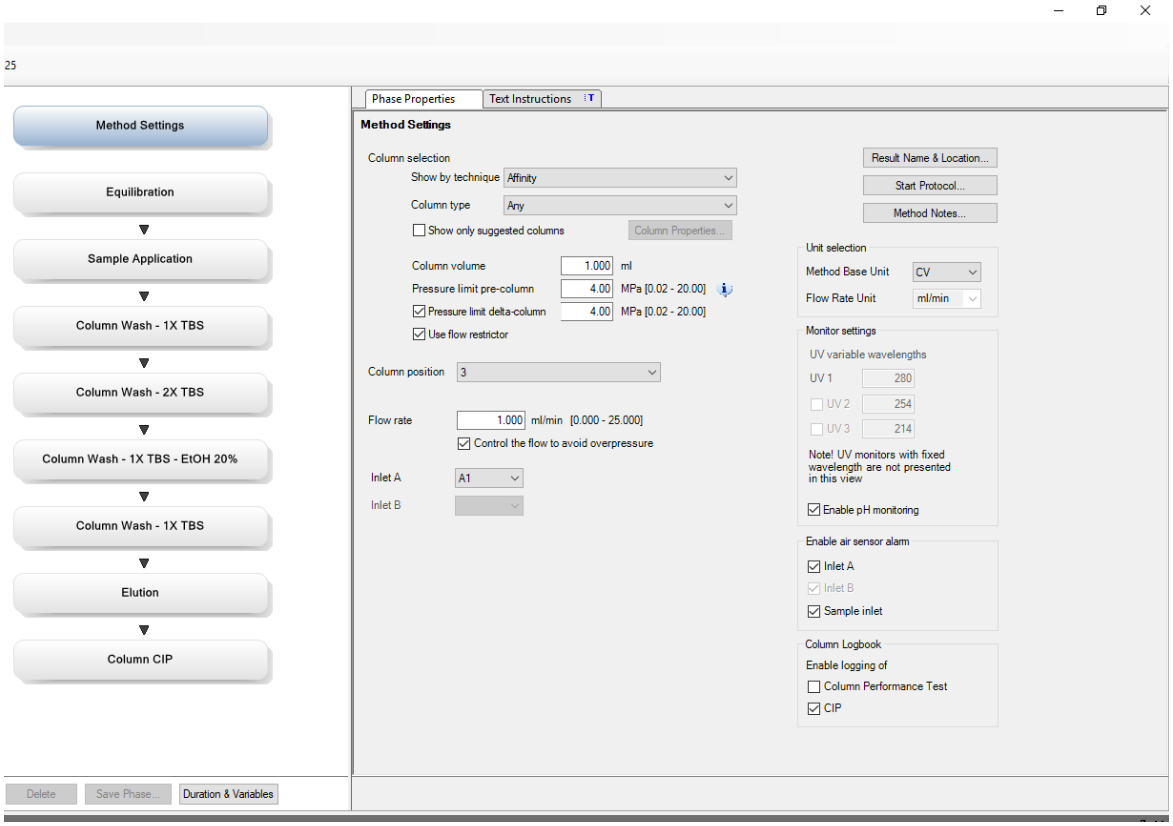

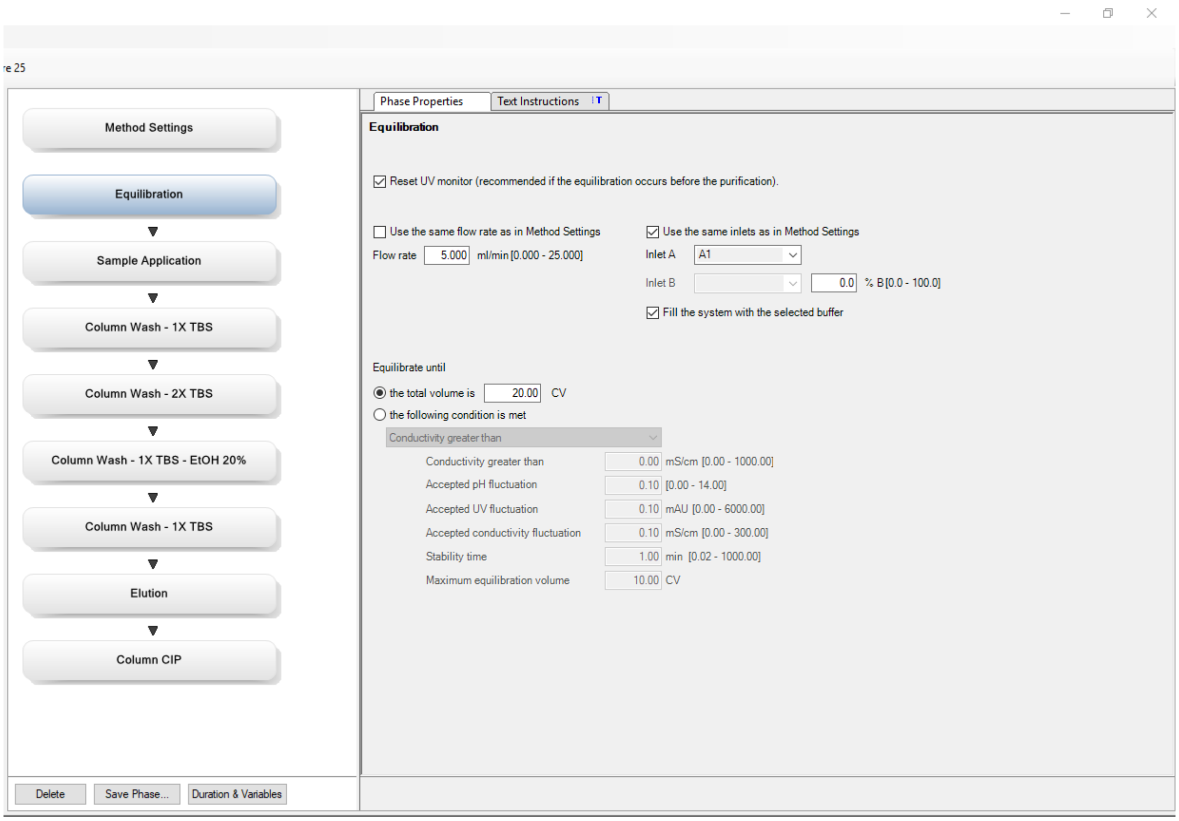
re 25
Method Settings
Equilibration
Sample Application
Column Wash - 1X TBS
Column Wash - 2X TBS
Column Wash - 1X TBS - EtOH 20%
Column Wash - 1X TBS
Elution
Column CIP
Delete
Save Phase...
Duration & Variables
Phase Properties
Text Instructions
Equilibration
Reset UV monitor (recommended if the equilibration occurs before the purification).
Use the same flow rate as in Method Settings
Flow rate 5.000 ml/min [0.000 - 25.000]
Use the same inlets as in Method Settings
Inlet A A1
Inlet B
0.0 % B [0.0 - 100.0]
Fill the system with the selected buffer
Equilibrate until
the total volume is 20.00 CV
the following condition is met
Conductivity greater than
Conductivity greater than 0.00 mS/cm [0.00 - 1000.00]
Accepted pH fluctuation 0.10 [0.00 - 14.00]
Accepted UV fluctuation 0.10 mAU [0.00 - 6000.00]
Accepted conductivity fluctuation 0.10 mS/cm [0.00 - 300.00]
Stability time 1.00 min [0.02 - 1000.00]
Maximum equilibration volume 10.00 CV

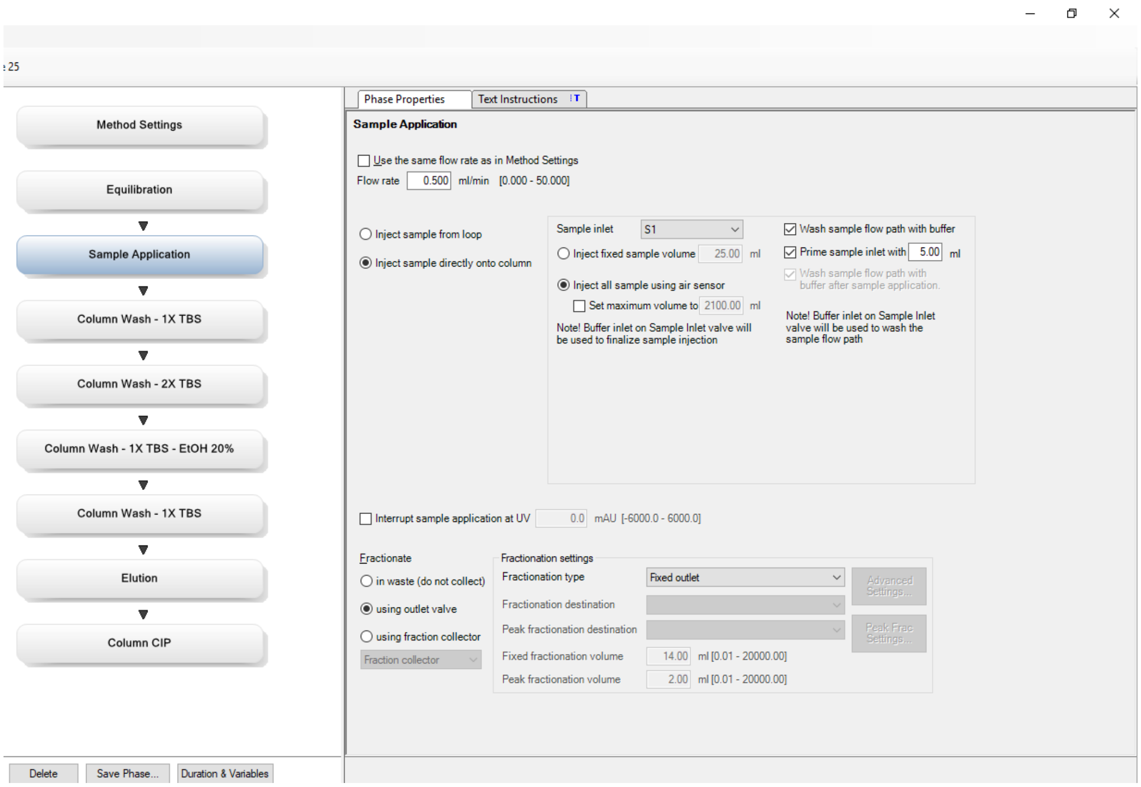
25
Method Settings
Equilibration
Sample Application
Column Wash - 1X TBS
Column Wash - 2X TBS
Column Wash - 1X TBS - EtOH 20%
Column Wash - 1X TBS
Elution
Column CIP
Delete
Save Phase...
Duration & Variables
Phase Properties
Text Instructions
Sample Application
Use the same flow rate as in Method Settings
Flow rate 0.500 ml/min [0.000 - 50.000]
Inject sample from loop
Inject sample directly onto column
Sample inlet S1
Inject fixed sample volume 25.00 ml
Inject all sample using air sensor
Set maximum volume to 2100.00 ml
Note! Buffer inlet on Sample Inlet valve will be used to finalize sample injection
Wash sample flow path with buffer
Prime sample inlet with 5.00 ml
Wash sample flow path with buffer after sample application.
Note! Buffer inlet on Sample Inlet valve will be used to wash the sample flow path
Interrupt sample application at UV 0.0 mAU [-6000.0 - 6000.0]
Fractionate
in waste (do not collect)
using outlet valve
using fraction collector
Fraction collector
Fractionation settings
Fractionation type Fixed outlet
Fractionation destination
Peak fractionation destination
Fixed fractionation volume 14.00 ml [0.01 - 20000.00]
Peak fractionation volume 2.00 ml [0.01 - 20000.00]
Advanced Settings...
Peak Frac Settings...

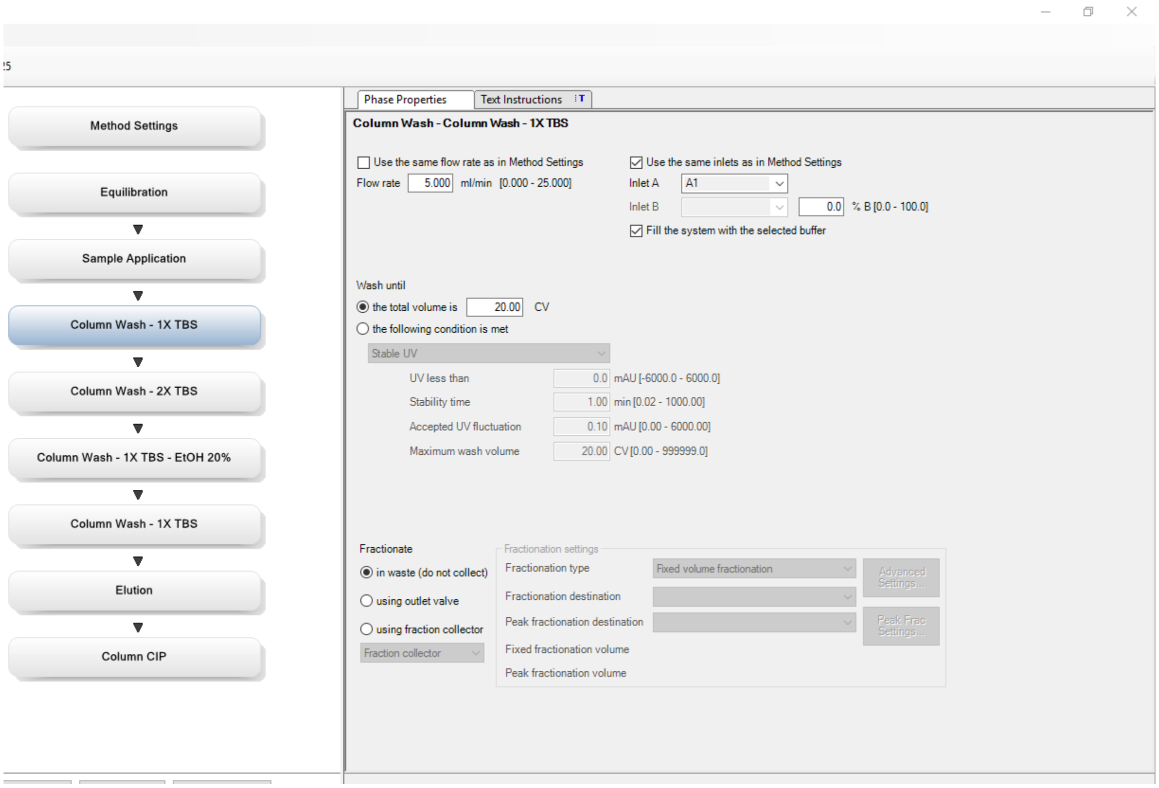
Method Settings
Equilibration
Sample Application
Column Wash - 1X TBS
Column Wash - 2X TBS
Column Wash - 1X TBS - EtOH 20%
Column Wash - 1X TBS
Elution
Column CIP
Phase Properties
Text Instructions
Column Wash - Column Wash - 1X TBS
Use the same flow rate as in Method Settings
Flow rate 5.000 ml/min [0.000 - 25.000]
Use the same inlets as in Method Settings
Inlet A A1
Inlet B 0.0 % B [0.0 - 100.0]
Fill the system with the selected buffer
Wash until
the total volume is 20.00 CV
the following condition is met
Stable UV
UV less than 0.0 mAU [-6000.0 - 6000.0]
Stability time 1.00 min [0.02 - 1000.00]
Accepted UV fluctuation 0.10 mAU [0.00 - 6000.00]
Maximum wash volume 20.00 CV [0.00 - 999999.0]
Fractionate
in waste (do not collect)
using outlet valve
using fraction collector
Fraction collector
Fractionation settings
Fractionation type
Fixed volume fractionation
Fractionation destination
Peak fractionation destination
Fixed fractionation volume
Peak fractionation volume

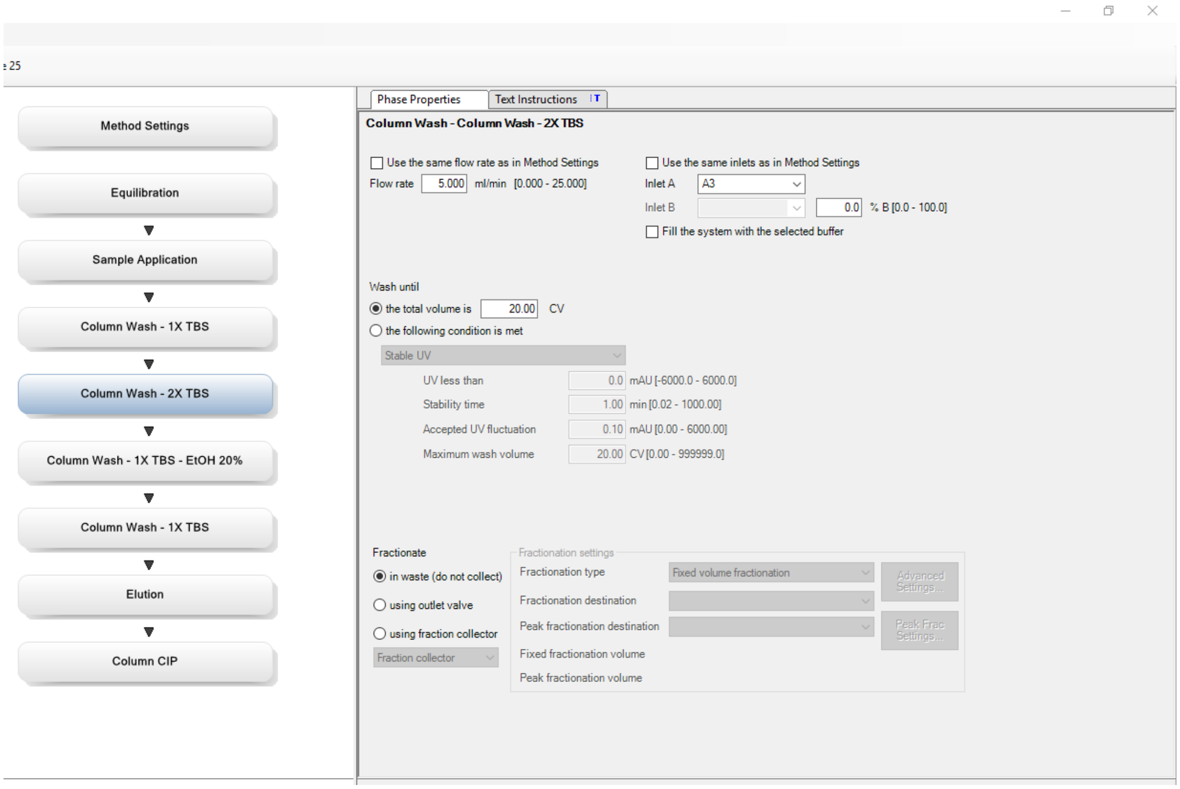
Method Settings
Equilibration
Sample Application
Column Wash - 1X TBS
Column Wash - 2X TBS
Column Wash - 1X TBS - EtOH 20%
Column Wash - 1X TBS
Elution
Column CIP
Phase Properties
Text Instructions
Column Wash - Column Wash - 2X TBS
Use the same flow rate as in Method Settings
Flow rate 5.000 ml/min [0.000 - 25.000]
Use the same inlets as in Method Settings
Inlet A A3
Inlet B 0.0 % B [0.0 - 100.0]
Fill the system with the selected buffer
Wash until
the total volume is 20.00 CV
the following condition is met
Stable UV
UV less than 0.0 mAU [-6000.0 - 6000.0]
Stability time 1.00 min [0.02 - 1000.00]
Accepted UV fluctuation 0.10 mAU [0.00 - 6000.00]
Maximum wash volume 20.00 CV [0.00 - 999999.0]
Fractionate
in waste (do not collect)
using outlet valve
using fraction collector
Fraction collector
Fractionation settings
Fractionation type
Fixed volume fractionation
Fractionation destination
Peak fractionation destination
Fixed fractionation volume
Peak fractionation volume

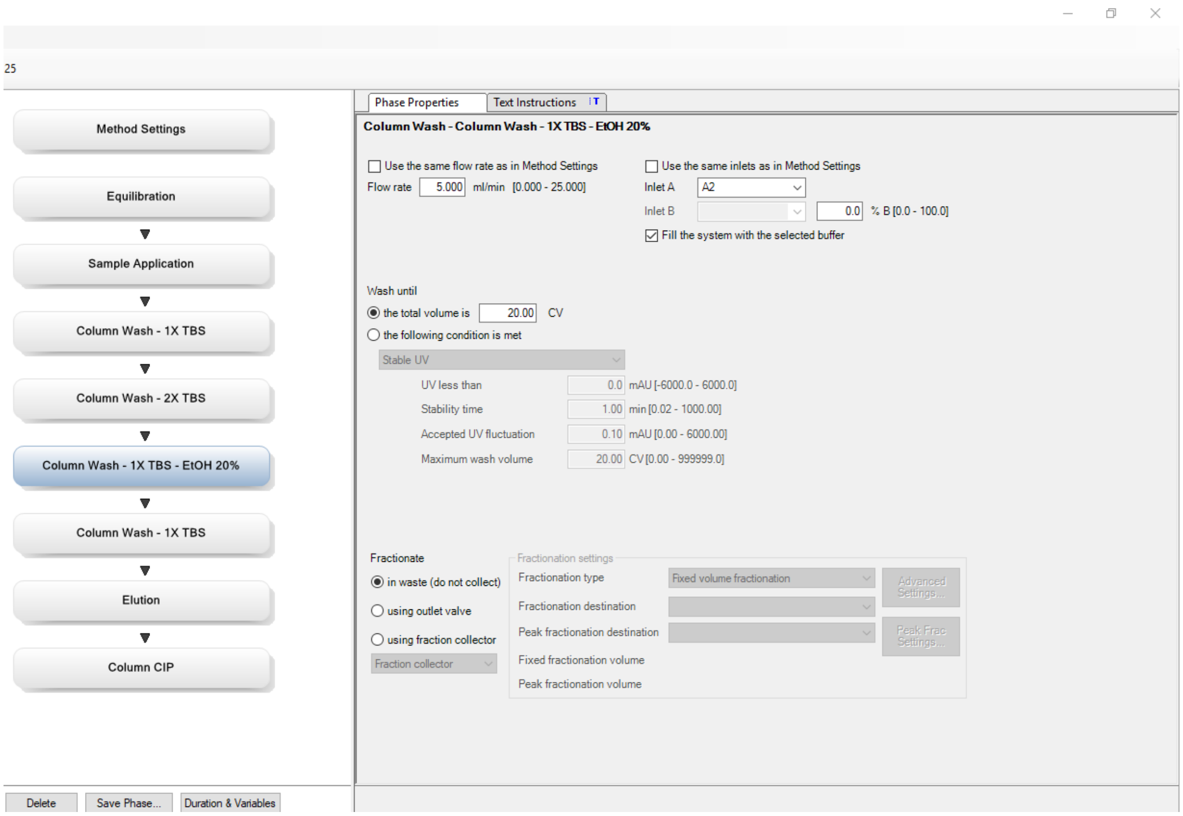
25
Method Settings
Equilibration
Sample Application
Column Wash - 1X TBS
Column Wash - 2X TBS
Column Wash - 1X TBS - EtOH 20%
Column Wash - 1X TBS
Elution
Column CIP
Delete
Save Phase...
Duration & Variables
Phase Properties
Text Instructions
Column Wash - Column Wash - 1X TBS - EtOH 20%
Use the same flow rate as in Method Settings
Flow rate 5.000 ml/min [0.000 - 25.000]
Use the same inlets as in Method Settings
Inlet A A2
Inlet B
0.0 % B [0.0 - 100.0]
Fill the system with the selected buffer
Wash until
the total volume is 20.00 CV
the following condition is met
Stable UV
UV less than 0.0 mAU [-6000.0 - 6000.0]
Stability time 1.00 min [0.02 - 1000.00]
Accepted UV fluctuation 0.10 mAU [0.00 - 6000.00]
Maximum wash volume 20.00 CV [0.00 - 999999.0]
Fractionate
in waste (do not collect)
using outlet valve
using fraction collector
Fraction collector
Fractionation settings
Fractionation type
Fixed volume fractionation
Fractionation destination
Peak fractionation destination
Fixed fractionation volume
Peak fractionation volume
Advanced Settings...
Peak Frac Settings...

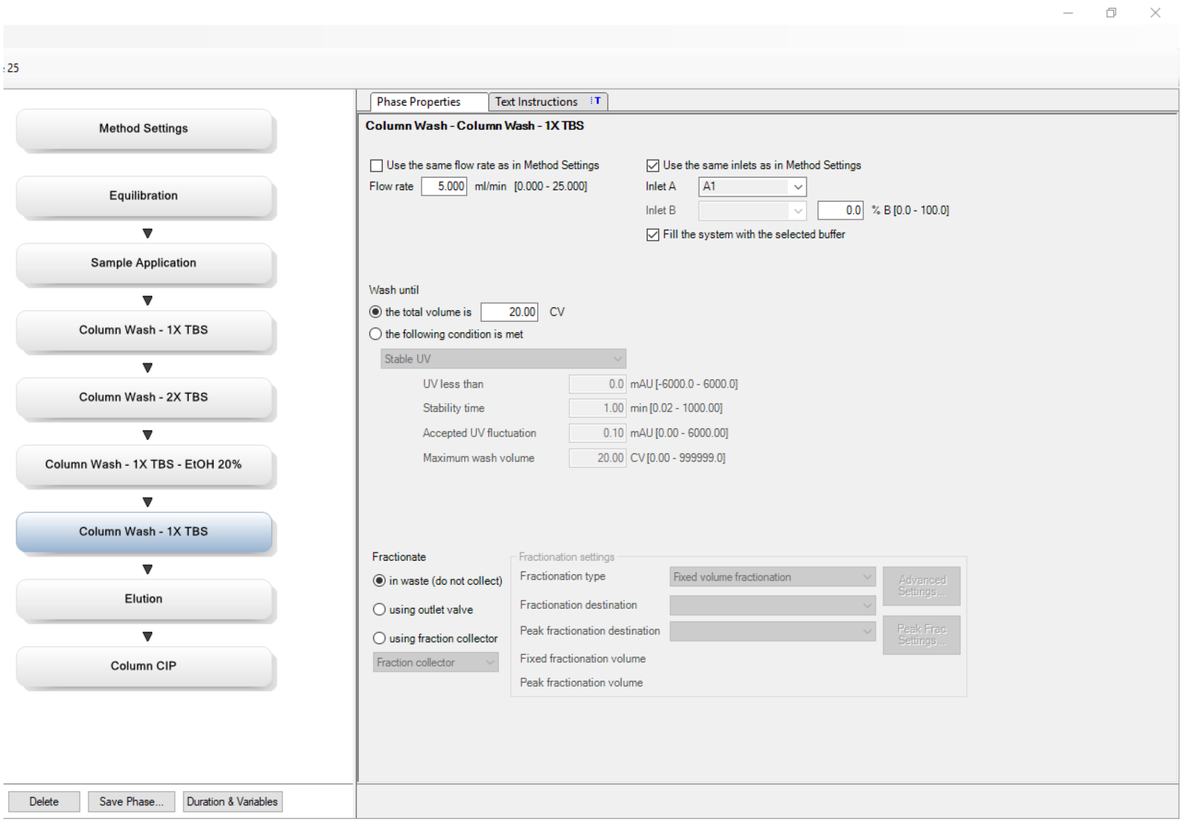
25
Method Settings
Equilibration
Sample Application
Column Wash - 1X TBS
Column Wash - 2X TBS
Column Wash - 1X TBS - EtOH 20%
Column Wash - 1X TBS
Elution
Column CIP
Delete
Save Phase...
Duration & Variables
Phase Properties
Text Instructions
Column Wash - Column Wash - 1X TBS
Use the same flow rate as in Method Settings
Flow rate 5.000 ml/min [0.000 - 25.000]
Use the same inlets as in Method Settings
Inlet A A1
Inlet B
0.0 % B [0.0 - 100.0]
Fill the system with the selected buffer
Wash until
the total volume is 20.00 CV
the following condition is met
Stable UV
UV less than 0.0 mAU [-6000.0 - 6000.0]
Stability time 1.00 min [0.02 - 1000.00]
Accepted UV fluctuation 0.10 mAU [0.00 - 6000.00]
Maximum wash volume 20.00 CV [0.00 - 999999.0]
Fractionate
in waste (do not collect)
using outlet valve
using fraction collector
Fraction collector
Fractionation settings
Fractionation type
Fixed volume fractionation
Fractionation destination
Peak fractionation destination
Fixed fractionation volume
Peak fractionation volume
Advanced Settings...
Peak Frac Settings...

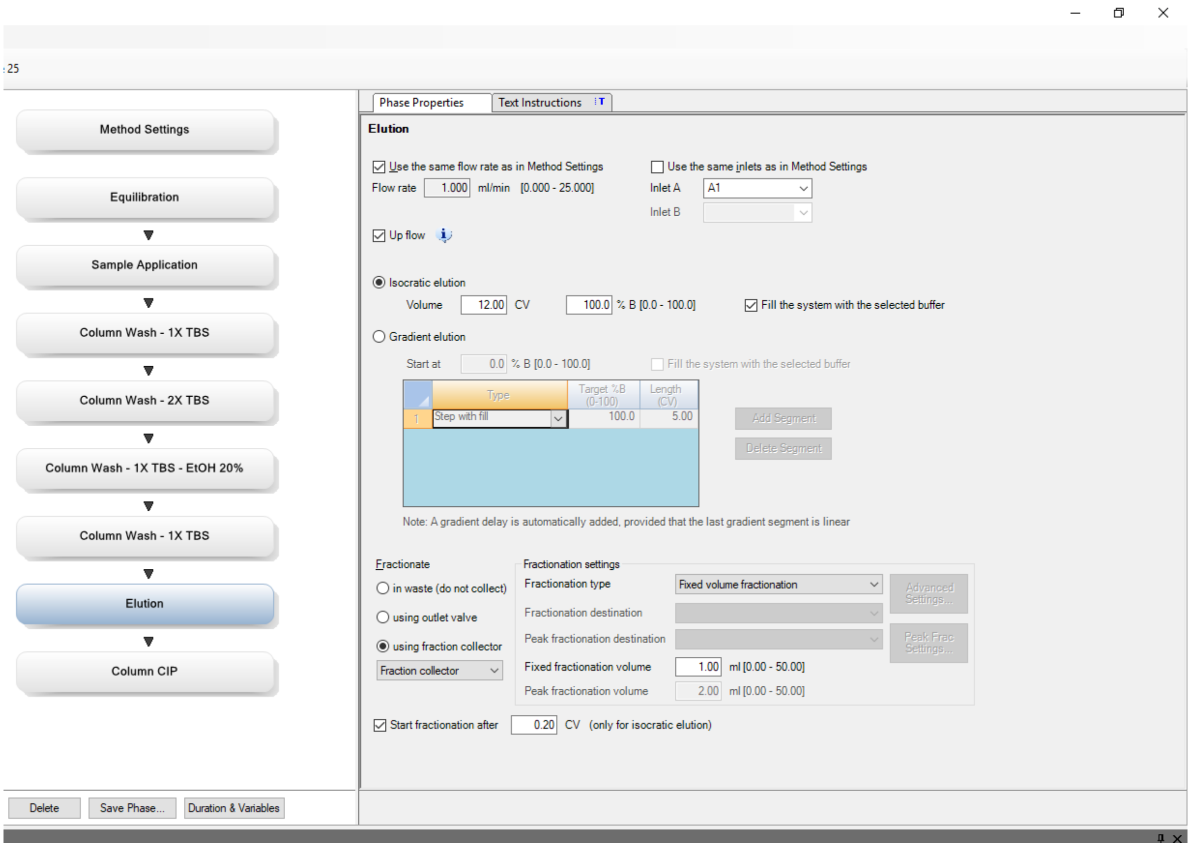
:25
Method Settings
Equilibration
Sample Application
Column Wash - 1X TBS
Column Wash - 2X TBS
Column Wash - 1X TBS - EtOH 20%
Column Wash - 1X TBS
Elution
Column CIP
Phase Properties
Text Instructions
Elution
Use the same flow rate as in Method Settings
Flow rate 1.000 ml/min [0.000 - 25.000]
Use the same inlets as in Method Settings
Inlet A A1
Inlet B
Up flow
Isocratic elution
Volume 12.00 CV 100.0 % B [0.0 - 100.0]
Fill the system with the selected buffer
Gradient elution
Start at 0.0 % B [0.0 - 100.0]
Fill the system with the selected buffer
Type
Target %B (0-100)
Length (CV)
Step with fill 100.0 5.00
Add Segment
Delete Segment
Note: A gradient delay is automatically added, provided that the last gradient segment is linear
Fractionate
in waste (do not collect)
using outlet valve
using fraction collector
Fraction collector
Fractionation settings
Fractionation type Fixed volume fractionation
Fractionation destination
Peak fractionation destination
Fixed fractionation volume 1.00 ml [0.00 - 50.00]
Peak fractionation volume 2.00 ml [0.00 - 50.00]
Advanced Settings...
Peak Frac Settings...
Start fractionation after 0.20 CV (only for isocratic elution)
Delete
Save Phase...
Duration & Variables

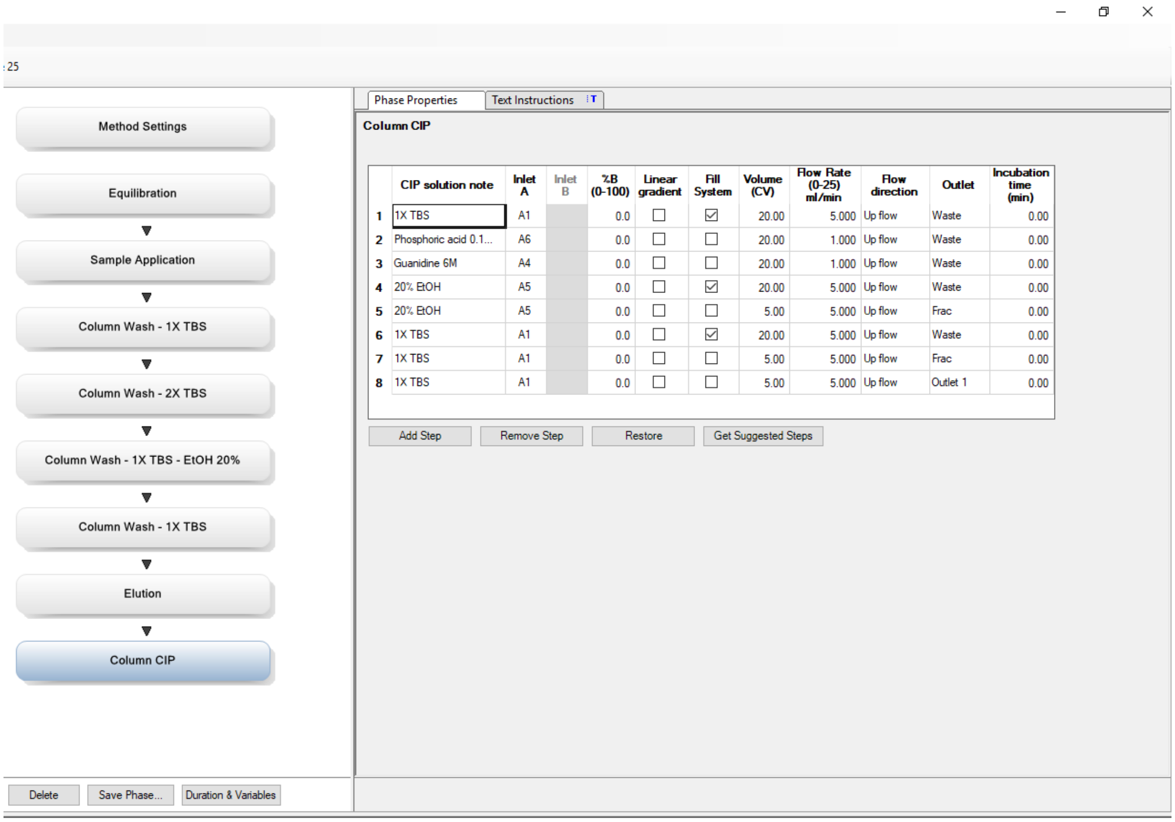
:25
Method Settings
Equilibration
Sample Application
Column Wash - 1X TBS
Column Wash - 2X TBS
Column Wash - 1X TBS - EtOH 20%
Column Wash - 1X TBS
Elution
Column CIP
Phase Properties
Text Instructions
Column CIP
CIP solution note
Inlet A
Inlet B
%B (0-100)
Linear gradient
Fill System
Volume (CV)
Flow Rate (0-25) ml/min
Flow direction
Outlet
Incubation time (min)
1 1X TBS A1 0.0 20.00 5.000 Up flow Waste 0.00
2 Phosphoric acid 0.1... A6 0.0 20.00 1.000 Up flow Waste 0.00
3 Guanidine 6M A4 0.0 20.00 1.000 Up flow Waste 0.00
4 20% EtOH A5 0.0 20.00 5.000 Up flow Waste 0.00
5 20% EtOH A5 0.0 5.00 5.000 Up flow Frac 0.00
6 1X TBS A1 0.0 20.00 5.000 Up flow Waste 0.00
7 1X TBS A1 0.0 5.00 5.000 Up flow Frac 0.00
8 1X TBS A1 0.0 5.00 5.000 Up flow Outlet 1 0.00
Add Step
Remove Step
Restore
Get Suggested Steps
Delete
Save Phase...
Duration & Variables

System CIP

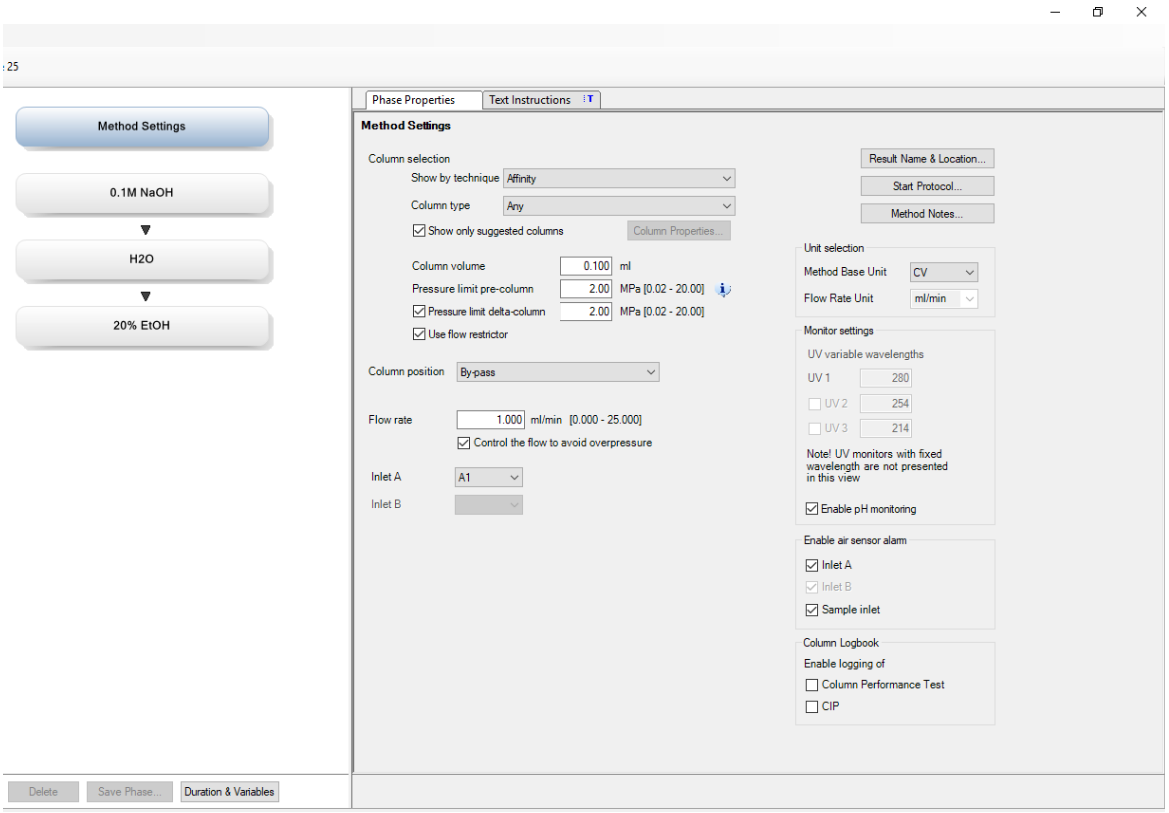
25
Method Settings
0.1M NaOH
H2O
20% EtOH
Delete
Save Phase...
Duration & Variables
Phase Properties
Text Instructions
Method Settings
Column selection
Show by technique
Affinity
Column type
Any
Show only suggested columns
Column Properties...
Column volume
0.100
ml
Pressure limit pre-column
2.00
MPa [0.02 - 20.00]
Pressure limit delta-column
2.00
MPa [0.02 - 20.00]
Use flow restrictor
Column position
By-pass
Flow rate
1.000
ml/min [0.000 - 25.000]
Control the flow to avoid overpressure
Inlet A
A1
Inlet B
Result Name & Location...
Start Protocol...
Method Notes...
Unit selection
Method Base Unit
CV
Flow Rate Unit
ml/min
Monitor settings
UV variable wavelengths
UV 1
280
UV 2
254
UV 3
214
Note! UV monitors with fixed wavelength are not presented in this view
Enable pH monitoring
Enable air sensor alarm
Inlet A
Inlet B
Sample inlet
Column Logbook
Enable logging of
Column Performance Test
CIP

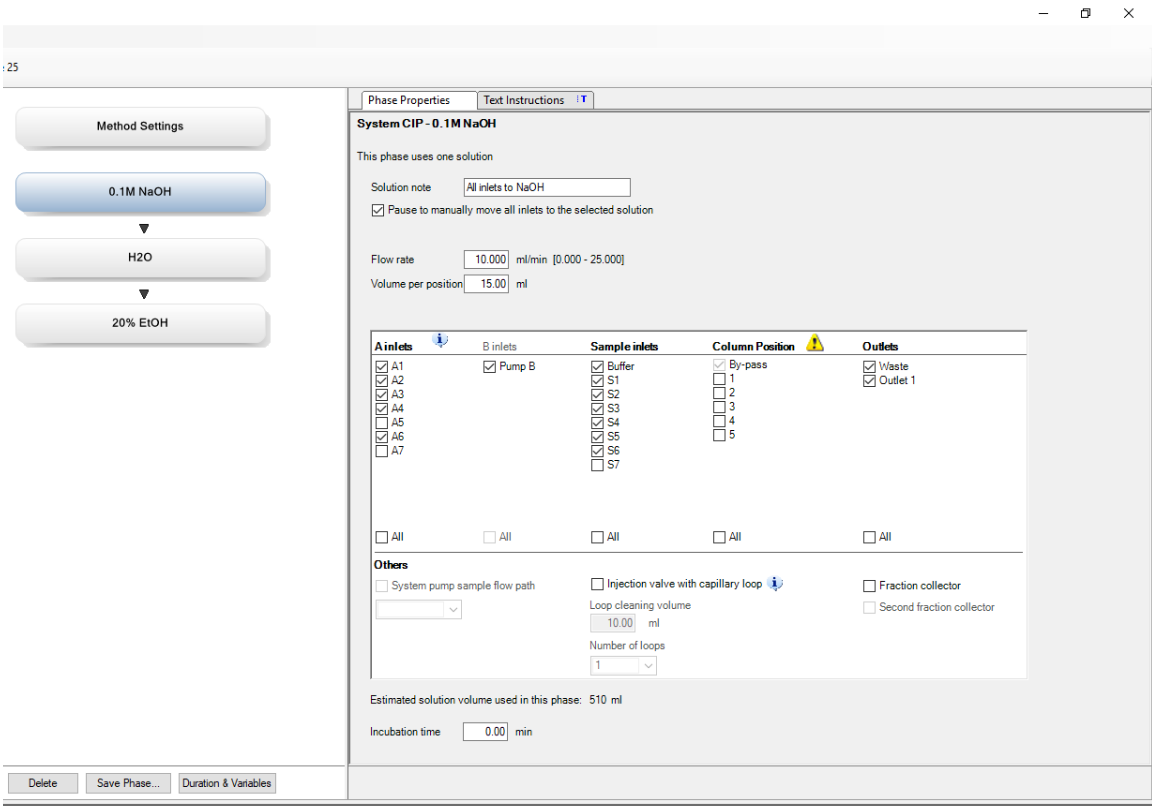
Method Settings
0.1M NaOH
H2O
20% EtOH
Phase Properties
Text Instructions
System CIP - 0.1M NaOH
This phase uses one solution
Solution note
All inlets to NaOH
Pause to manually move all inlets to the selected solution
Flow rate 10.000 ml/min [0.000 - 25.000]
Volume per position 15.00 ml
A inlets
B inlets
Sample inlets
Column Position
Outlets
A1 A2 A3 A4 A5 A6 A7
Pump B
Buffer S1 S2 S3 S4 S5 S6 S7
By-pass 1 2 3 4 5
Waste
Outlet 1
All
Others
System pump sample flow path
Injection valve with capillary loop
Loop cleaning volume 10.00 ml
Number of loops 1
Fraction collector
Second fraction collector
Estimated solution volume used in this phase: 510 ml
Incubation time 0.00 min
Delete
Save Phase...
Duration & Variables

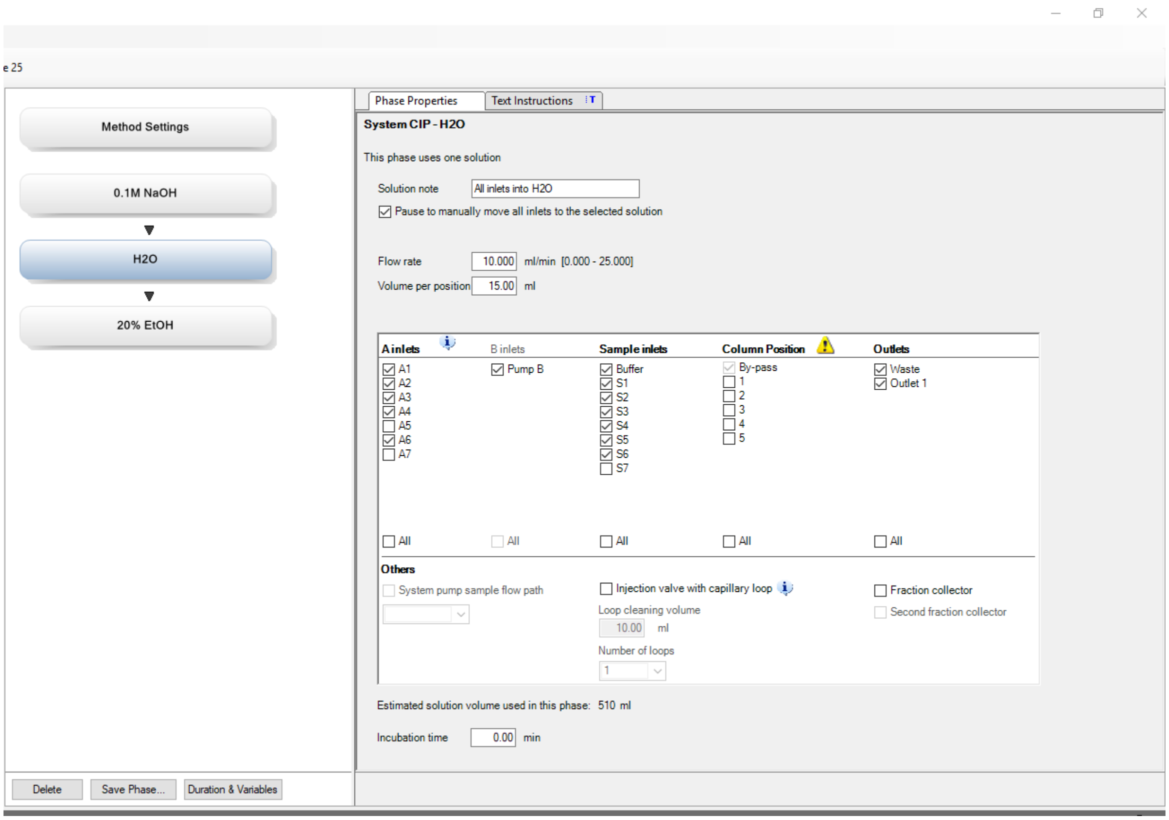
Method Settings
0.1M NaOH
H2O
20% EtOH
Delete
Save Phase...
Duration & Variables
Phase Properties
Text Instructions
System CIP - H2O
This phase uses one solution
Solution note
All inlets into H2O
Pause to manually move all inlets to the selected solution
Flow rate 10.000 ml/min [0.000 - 25.000]
Volume per position 15.00 ml
A inlets
B inlets
Sample inlets
Column Position
Outlets
A1 A2 A3 A4 A5 A6 A7
Pump B
Buffer S1 S2 S3 S4 S5 S6 S7
By-pass 1 2 3 4 5
Waste
Outlet 1
All
Others
System pump sample flow path
Injection valve with capillary loop
Loop cleaning volume 10.00 ml
Number of loops 1
Fraction collector
Second fraction collector
Estimated solution volume used in this phase: 510 ml
Incubation time 0.00 min

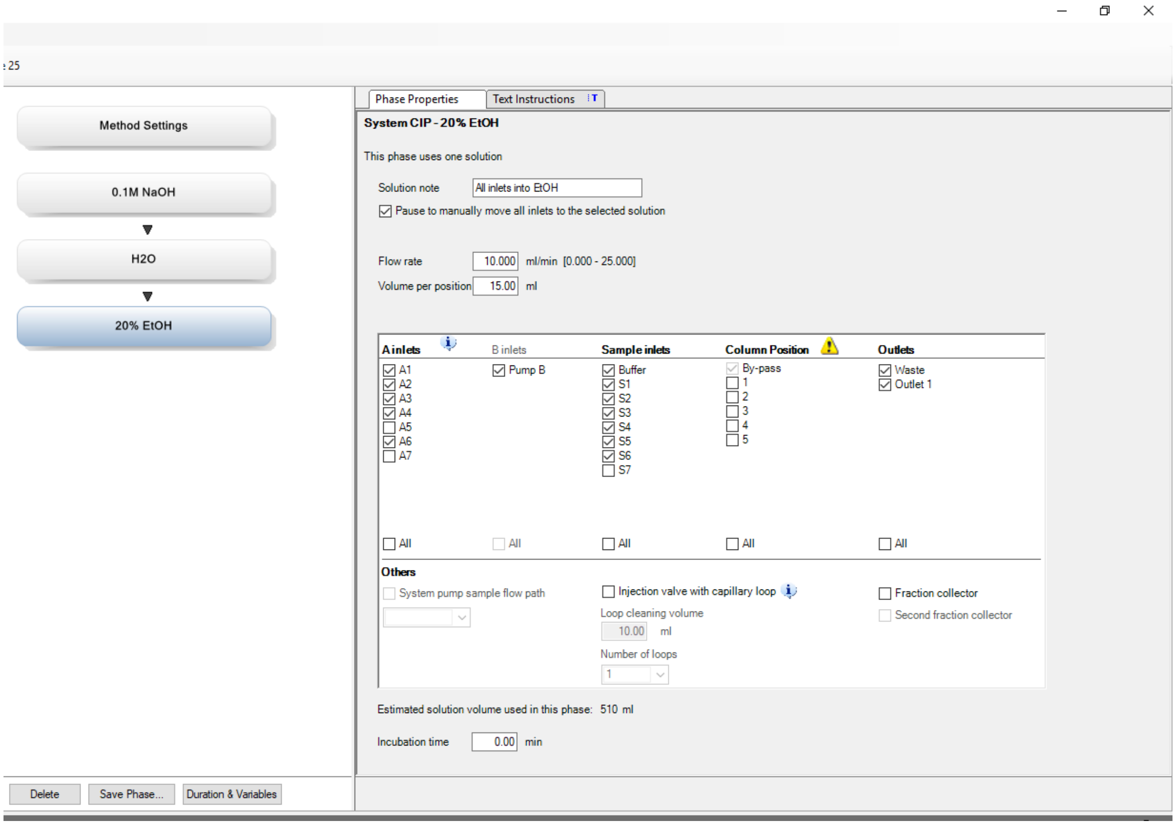
Method Settings
0.1M NaOH
H2O
20% EtOH
Delete
Save Phase...
Duration & Variables
Phase Properties
Text Instructions
System CIP - 20% EtOH
This phase uses one solution
Solution note
All inlets into EtOH
Pause to manually move all inlets to the selected solution
Flow rate 10.000 ml/min [0.000 - 25.000]
Volume per position 15.00 ml
A inlets
B inlets
Sample inlets
Column Position
Outlets
A1 A2 A3 A4 A5 A6 A7
Pump B
Buffer S1 S2 S3 S4 S5 S6 S7
By-pass 1 2 3 4 5
Waste
Outlet 1
All
Others
System pump sample flow path
Injection valve with capillary loop
Loop cleaning volume 10.00 ml
Number of loops 1
Fraction collector
Second fraction collector
Estimated solution volume used in this phase: 510 ml
Incubation time 0.00 min
