# Supplementary material for: High-efficiency purification of divergent AAV serotypes using AAVX affinity chromatography
Source: Mol Ther Methods Clin Dev. 2022 Dec 16;28:146–59. doi: 10.1016/j.omtm.2022.12.009 (PMC9823220; doi:10.1016/j.omtm.2022.12.009)
Supplement: Document S3. Article plus supplemental information [file mmc3.pdf]

Molecular Therapy
Methods & Clinical Development
Original Article

High-efficiency purification of divergent AAV serotypes using AAVX affinity chromatography

Michael Florea,[1,2,3] Fotini Nicolaou,[1] Simon Pacouret,[1] Eric M. Zinn,[1] Julio Sanmiguel,[1] Eva Andres-Mateos,[1] Carmen Unzu,[1] Amy J. Wagers,[3,6,7,8] and Luk H. Vandenberghe[1,4,5,6]

[1]Grousbeck Gene Therapy Center, Schepens Eye Research Institute and Massachusetts Eye and Ear Infirmary, Boston, MA, USA; [2]Harvard Ph.D. Program in Biological and Biomedical Sciences, Division of Medical Sciences, Harvard University, Boston, MA, USA; [3]Department of Stem Cell and Regenerative Biology, Harvard University, Cambridge, MA, USA; [4]Ocular Genomics Institute, Department of Ophthalmology, Harvard Medical School, Boston, MA, USA; [5]The Broad Institute of Harvard and MIT, Boston, MA, USA; [6]Harvard Stem Cell Institute, Harvard University, Cambridge, MA, USA; [7]Paul F. Glenn Center for the Biology of Aging, Harvard Medical School, Boston, MA, USA; [8]Section on Islet Cell and Regenerative Biology, Joslin Diabetes Center, Boston, MA, USA

The adeno-associated viral vector (AAV) provides a safe and efficient gene therapy platform with several approved products that have marked therapeutic impact for patients. However, a major bottleneck in the development and commercialization of AAV remains the efficiency, cost, and scalability of AAV production. Chromatographic methods have the potential to allow purification at increased scales and lower cost but often require optimization specific to each serotype. Here, we demonstrate that the POROS CaptureSelect AAVX affinity resin efficiently captures a panel of 15 divergent AAV serotypes, including the commonly used AAV2, AAV8, AAV9, PHP.B, and Anc80. We also find that AAVX resin can be regenerated repeatedly without loss of efficiency or carry-over contamination. While AAV preps purified with AAVX showed a higher fraction of empty capsids than preps purified using iodixanol ultracentrifugation, the potency of the AAVX purified vectors was comparable with that of iodixanol purified vectors both *in vitro* and *in vivo*. Finally, optimization of the purification protocol resulted in a process with an overall efficiency of 65%–80% across all scales and AAV serotypes tested. These data establish AAVX affinity chromatography as a versatile and efficient method for purification of a broad range of AAV serotypes.

INTRODUCTION

Adeno-associated viruses (AAVs) are small, non-enveloped single-stranded DNA viruses discovered in the 1960s as contaminants of adenovirus preparations.[1,2] They induce limited host immune response and are not associated with any known disease, yet were found to be highly efficient at delivering DNA cargo to many tissues in multiple animal species.[3] AAV vectors are thus widely used as a gene transfer tool in basic research and in translational and clinical gene therapy.[4] Their higher use has increased demand for AAV manufacturing both in terms of the quality of the preparation and the quantity of the material.

Currently, for research and for some clinical purposes, the commonly used AAV purification method uses ultracentrifugation of the sample on a cesium chloride (CsCl) or iodixanol density gradient.[5–7] This process is appealing for two reasons: first, it is serotype agnostic with little process optimization needed for the various AAV products researchers seek to purify; and second, it remains one of the more efficient methods of separation of genome-containing (or "full") capsids from empty or partially filled capsids. However, ultracentrifugation is a manual multi-step processes (sample concentration, preparation of density gradient, sample loading, centrifugation, and aspiration of the target layer). This makes it labor intensive and difficult to scale, and adds a requirement for precise handling.[8] Finally, ultracentrifugation may co-purify contaminants that have the same sedimentation coefficient as AAV.[8]

Liquid chromatography provides a more scalable, less laborious, and possibly more efficient purification method, particularly under high-performance liquid chromatography (HPLC) conditions, as has been shown for the purification of proteins and small molecules.[9] For AAV, several chromatographic methods have been developed, most using AVB Sepharose affinity, cation exchange, or anion exchange chromatography.[10–13] While these methods demonstrate the feasibility and efficiency of AAV chromatographic purification, they also require substantial serotype-specific optimization. Thus, they are not optimal for AAV purification in a research setting, where different serotypes need to be purified for different applications in a flexible process.

Recently, several AAV binding resins have been commercially released, including AVB resin (AVB Sepharose High Performance;

Received 28 April 2022; accepted 12 December 2022;
https://doi.org/10.1016/j.omtm.2022.12.009.

Correspondence: Luk H. Vandenberghe, Grousbeck Gene Therapy Center, Schepens Eye Research Institute and Massachusetts Eye and Ear Infirmary, Boston, MA, USA.
E-mail: luk_vandenberghe@meei.harvard.edu

 © 2022 The Authors.
This is an open access article under the CC BY license (http://creativecommons.org/licenses/by/4.0/).

GE Healthcare, Chicago, IL, USA) and POROS CaptureSelect AAV8, AAV9, and AAVX resins (Thermo Fisher Scientific, Waltham, MA, USA). In the case of AVB resin, it was shown that affinity chromatography using AVB can efficiently purify AAV1, AAV2, AAV5, AAV6, and rh10, but requires serotype-specific optimization and does not bind to multiple other serotypes, including AAV8 and AAV9.[13,14] POROS CaptureSelect AAV8 and AAV9 resins bind and were specifically developed for purification of AAV8 and AAV9, respectively, but not other serotypes (POROS CaptureSelect product datasheet).[11,13] POROS CaptureSelect AAVX is a 50-µm resin consisting of a rigid crosslinked poly(styrene divinylbenzene) bead backbone, coated with crosslinked polyhydroxylated polymer, and linked to a camelid heavy-chain-only single-domain antibody fragment. The camelid antibody was raised against a conserved region of the AAV capsid, and the AAVX resin is marketed as a pan-AAV affinity resin capable of binding multiple different AAV serotypes (POROS CaptureSelect product datasheet).[11] However, to date there are no independently generated published data on assessing the performance of AAVX. For this reason, we sought to evaluate the AAVX resin for its ability to bind various AAV serotypes and its utility to be incorporated into a fully integrated AAV purification process.

RESULTS

AAVX binds several AAV serotypes

We first sought to test, on a small scale, which serotypes bind to the POROS CaptureSelect AAVX resin (subsequently denoted as AAVX). To this end, the phylogenetically diverse AAV serotypes AAV2, AAV2_HSPG, AAV4, AAV5, AAV6.2, AAV7, AAV8, AAV9, rh10, rh32.33, PHP.B, Anc80, and AAV7m8[1,15–23] (Figure 1A) were produced at small scale, purified via ultracentrifugation on an iodixanol gradient, and applied to the AAVX resin bed in a static binding assay. After incubation, resin was washed with PBS, AAV was eluted using 0.1 M citric acid and quantified in different fractions using qPCR (Figure 1B). The result of this binding assay demonstrates that AAVX binds all of the tested serotypes with relatively high efficiency, similarly to the positive control of AAV9 incubated with the POROS AAV9 resin. Recovery was >95% for all serotypes tested except for Anc80, which showed around 80% recovery. On the other hand, the control sample of AAV2 incubated with the POROS AAV9 resin showed poor (<5%) binding efficiency (Figure 1B). This suggests that the AAVX resin has broad affinity and may significantly improve the purification process for divergent serotypes.

AAVX affinity chromatography can be used to purify AAV

Next, we aimed to determine whether AAV vectors could be purified with the AAVX resin via HPLC, choosing the Corning HYPERFlask (560 mL harvest volume, 1,720 cm^2 surface area) as the process development vessel for scale-up production. We chose AAV2 and Anc80 because of their high sequence divergence and broad research and clinical utility, and for both serotypes we purified preparations from a single HYPERFlask using AAVX-HPLC. In short, the production and purification process consisted of triple transfection of adherent HEK293 cells, cell pellet and medium harvest and high salt lysis 3 days post transfection, benzonase treatment, clarification of lysate by centrifugation and filtration, and AAVX affinity chromatographic purification at room temperature with immediate neutralization of the eluted vector. The vector was then sterilized through 0.22-µm filtration and buffer-exchanged final buffer exchanges, and concentrated using a 50 kDa molecular weight cutoff filtration unit (Amicon Ultra-15). Recovery in each of the different chromatography fractions (Figure S1) was quantified by qPCR for DNAse-resistant vector genomes (Figure 1C). Results from these experiments indicated that the majority of input vector was found in the elution fraction, with only a minor fraction of vector lost in the flow-through or Tris-buffered saline (TBS) and ethanol fractions. Additional preps indicated that combined average purification efficiency for both AAV2 and Anc80 without serotype-specific optimization was around 50% (Figure 1D). The average yield of AAV2 and Anc80 from this initial process was 10^{13} vector genomes (vg) of AAV per HYPERFlask, which was maintained for the serotypes AAV9 and PHP.eB (Figure 1E).

AAVX can be regenerated for re-use without loss of efficiency or carry-over contamination

Next, we aimed to determine whether HPLC purification of AAV with AAVX also functions at small scale and whether resin can be re-used multiple times without contamination or loss of efficiency. Re-using resin is of interest because it decreases the cost and labor associated with AAV purification and allows automatic back-to-back purification of multiple preparations. We produced five different AAV1 preps at small scale (from one and a half 15-cm dishes per prep), whereby the vectors of the second to fifth AAV prep were identical except for a unique 100-bp DNA barcode region (Figure 2A). We purified the preps consecutively from prep 1 to prep 5 using the same bed of resin. The resin was regenerated using 6 M guanidine and equilibrated with TBS and 20% ethanol washes between each run. We then quantified the vector genomes in the input lysate, the flow-through, and final elution via qPCR (Figure 2B). Throughout the experiment most of the input vector was found in the elution fraction (<2% found in flow-through), and there was no detectable loss of purification efficiency. Furthermore, next-generation sequencing of the barcode region in the fifth prep showed that the majority (99.93%) of genomes found in the elution fraction came from the correct fifth prep, not preps 2–4 (Figure 2C). These results indicate that resin can be re-used multiple times without considerable loss of efficiency or carry-over contamination. We observed similar results with different batches of AAVX resin in other experiments (see Figures S2 and S3), indicating low AAVX batch-to-batch variability.

Using the same method as described above, we also asked whether the addition of Pluronic F-68 to HPLC buffers increases purification efficiencies. Pluronic F-68 is a non-ionic surfactant that has been shown to decrease AAV non-specific binding to various surfaces including plasticware.[24,25] As HPLC contains long and narrow plastic tubing, we reasoned that addition of Pluronic F-68 may increase purification efficiency by reducing AAV binding to plastic. To test this hypothesis, we added Pluronic F-68 to HPLC buffers to the concentration of 0.1%

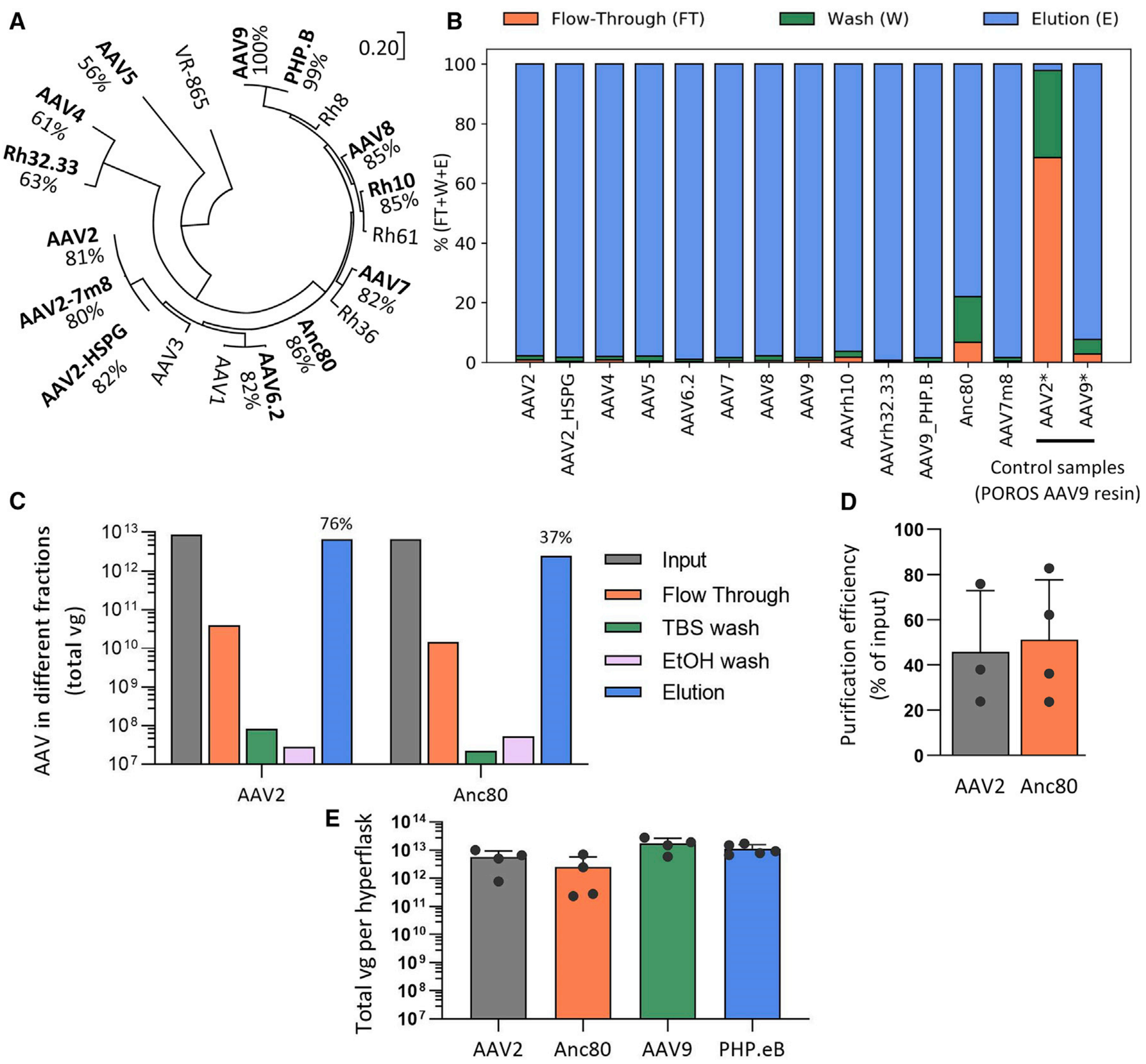

Figure 1. AAV purification using AAVX affinity chromatography

(A) Phylogeny depicting the diversity of AAV capsids included in this report (bold) along with the percent identity (by amino acid) compared with AAV9. The tree is drawn to scale with branch lengths depicting substitutions per site. VR-865 is an avian AAV used as an outgroup. (B) Affinity of AAVX to various AAV serotypes tested in a static binding assay. The flow-through (FT), wash (W), and eluted fractions (E) were collected and analyzed by qPCR to quantify their vector genome copies. Data represented as percent vector genomes (vg) of the input. Each serotype was applied to unused AAVX resin. (C) AAV purification of AAV2 and Anc80 using AAVX resin in an HPLC setting. Fractions were taken from input, flow-through, at Tris-buffered saline (TBS) and ethanol wash steps and at elution, and AAV content was quantified using qPCR. Percent recovery for these purifications is shown above elution bars. N = 1 each. (D) Average purification efficiencies of AAV2 and Anc80 (percent recovery of AAV in the elution). (E) Total yields of purified AAV2, Anc80, AAV9, and PHP.eB preps with no optimization of the process. Each dot represents an AAV prep from one HYPERFlask (1,720 cm^2 growth area). Error bars denote standard deviation. All purifications were carried out at room temperature, using 1-mL AAVX column at 1 mL/min flow rate. All values estimated are above qPCR limit of detection (approximately 10^5 vg/mL).

v/v and repeated the experiment described in Figure 2A (Figure S2A). The results indicate that Pluronic F-68 did not increase elution efficiencies for AAV1. However, it showed a trend toward increased efficiencies at the post-elution purification steps (Figures S2B and S2C) and did not increase carry-over contamination (Figures S2D and S2E). This indicates that Pluronic F-68 is a safe addition to HPLC buffers and may be considered for serotypes that are known to be strongly affected by binding to plastic.

Purification efficiency is temperature dependent

A major challenge in AAV manufacturing during the downstream process is to prepare stable viral vectors, preventing degradation and maintaining production efficiency in a reproducible and cost-effective way. For this reason, HPLC machines are commonly housed and operated at low ambient temperatures (4°C or 10°C) to improve protein stability. As such, we evaluated the role of temperature on purification efficiency during AAV9 and PHP.eB purification. We found

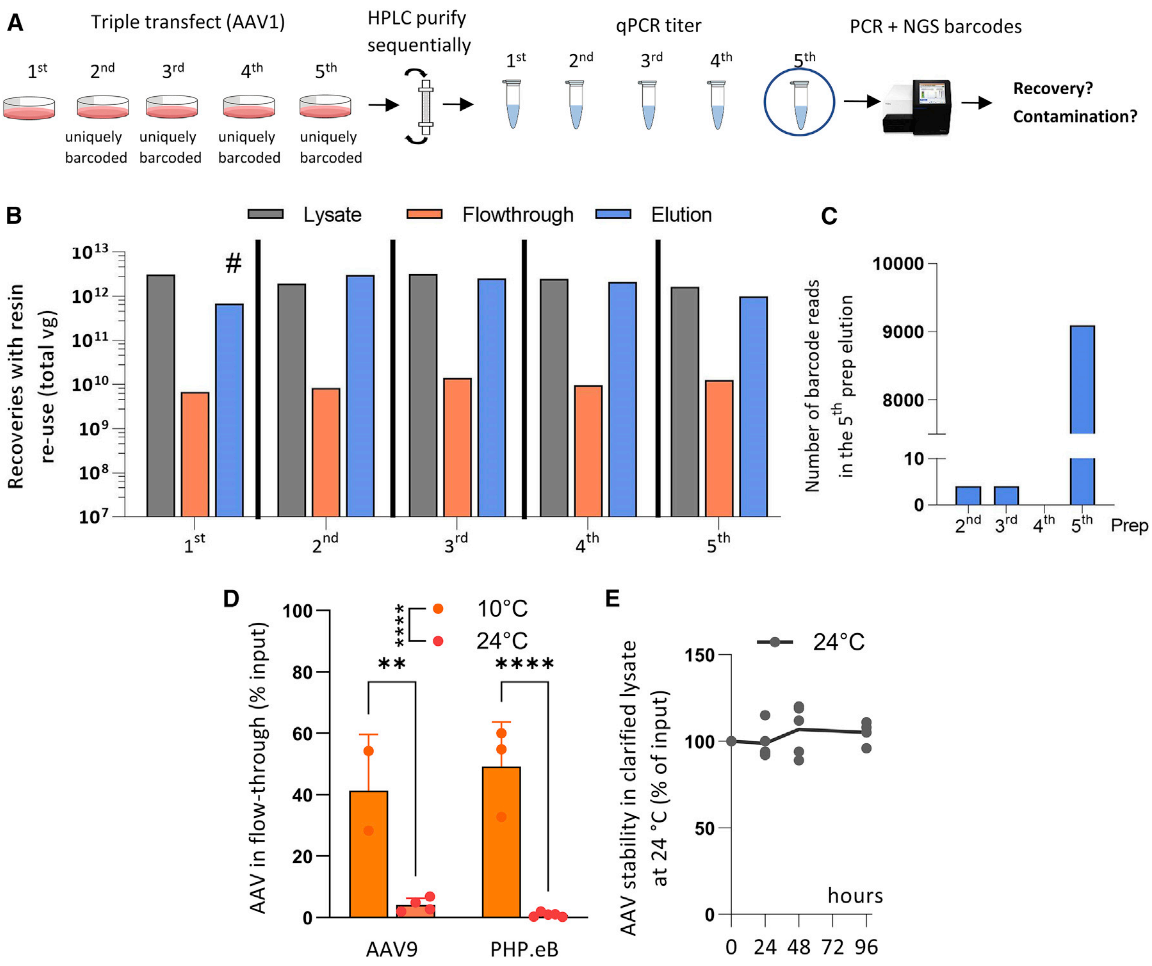

Figure 2. Effect of resin regeneration and temperature on purification efficiency

(A) Overview of experimental design of figures (B) and (C). Five small-scale AAV1 preps were produced and purified sequentially on HPLC with AAVX resin without changing the resin between purifications. One prep contained AAV from one and a half 15-cm dishes. Preps 2–5 were identical except for a 100-bp barcode region. Vector genomes were quantified across all purifications. For the fifth prep, the barcode region was PCR amplified and next-generation sequenced, and the unique barcodes corresponding to each prep were quantified to estimate carry-over contamination from preps 2–4. AAV was applied to a column packed with 1 mL of AAVX resin at 1 mL/min flow rate at room temperature. (B) Purification efficiency with repeated resin use. Vector genomes in lysate, flow-through, and elution. Hash mark indicates that some of the sample was lost due to handling error. (C) Estimation of carry-over contamination. Barcode counts from preps 2–5, in the fifth prep estimated via next-generation sequencing. (D) Effect of purification temperature on the percentage of vector genomes found in flow-through for AAV9 and PHP.eB. Difference was assessed using two-way ANOVA with Šídák's post hoc tests. (E) Stability of AAV (PHP.eB) in clarified lysate at 24°C over 96 h. **$p < 0.01$, ***$p < 0.001$, ****$p < 0.0001$. Error bars denote standard deviation.

that a substantial proportion of vector (40%–50%) was lost in flow-through at 10°C whereas viral losses accounted for less than 5% at 24°C (Figure 2D). However, viral vector degradation due to external factors such as high ambient temperature can adversely impact stability and transduction efficiency of the viral product. We therefore tested thermal stability of clarified viral harvests during the downstream process by keeping AAV input virus at 24°C and assessing vector degradation using qPCR quantification over 4 days. The results indicate that AAV titers were stable over the 4-day timeline (Figure 2E). Overall, these results indicate that purification at ambient (24°C) temperatures reduces viral loss during purification and that AAV remains stable at these temperatures during the timeline of the purification process.

An optimized purification protocol

AAVX affinity purification can be utilized for a variety of viral vectors; however, optimization of the various workflow steps will enable more cost-effective, high-yield, and reproducible production. We therefore performed a granular downstream optimization process for production of a larger scale (up to 10^{14} vg) of AAV. Major challenges in the workflow included efficient lysis and the design of filtration and final formulation steps that minimize AAV loss. This optimization resulted in a process with the following components (see supplemental protocol for process details):

1. *In situ cell lysis using detergents and nucleases.* Based on the protocol described by Florencio et al. [26] and our own observations,

in situ lysis using detergents and nucleases is as efficient as separate lysis of the cell pellet and may be more efficient than *in situ* lysis using hypertonic salt. To obtain one-step lysis and DNA/RNA removal, we added RNase A (4.4 μg/mL), Turbonuclease (2.5 U/mL), Triton X-(0.5% v/v), and Pluronic F-68 (0.001% v/v) to the HYPERFlask and incubated for 1 h at 37°C with orbital shaking at 150 rpm to aid lysis with mechanical forces (see supplemental protocol for details). Here, Triton X-100 and RNase A act as primary lysis agents, Turbonuclease acts to degrade plasmid and cell DNA, and Pluronic F-68 serves to decrease potential AAV binding to plastics.

2. *Addition of Pluronic F-68 to all buffers*. Based on our observation that the addition of Pluronic F-68 does not reduce HPLC purification efficiencies (Figure S2), and based on multiple anecdotal sources indicating that the coating of plastic and/or filter surfaces with surfactants may reduce protein binding, we added Pluronic F-68 at 0.01% v/v to the elution buffer and incubated all plasticware that came into contact with AAV with a Pluronic F-68 containing solution (Final Formulation Buffer [FFB]: 1× PBS, 172 mM NaCl, 0.001% Pluronic F-68) for approximately 15 min at room temperature. Additionally, pipette tips and serological pipettes are similarly coated with FFB prior to handling AAV.
3. *Stringent resin cleaning with 0.1 M phosphoric acid and 6 M guanidine*. While we observed no loss in AAV binding efficiencies with resin re-use at small scales with AAV1 (Figures 2 and S2), we did observe some loss of binding efficiencies with re-use at large scales, particularly for PHP.eB (data not shown). Based on the recommendations of the AAVX manufacturers (A. Becerra, Thermo Fisher Scientific, personal communication), we increased resin-cleaning stringency from a 5-min wash with 6 M guanidine alone to a 15-min wash with 0.1 M phosphoric acid (pH 1), followed by 15-min wash with 6 M guanidine-HCl. These changes restored efficient resin binding to up to at least six resin regenerations for both AAV9 and PHP.eB (Figures S3A and S3B) with no significant AAV losses in flow-through (Figure S3C).
4. *Improvement of buffer exchange*. Our analysis indicated substantial losses at the buffer exchange step (25%–50%; data not shown). This can be caused by AAV binding to plastic/filter surfaces, shear stress, or overconcentration on the filter surface during buffer exchange, leading to aggregation, sedimentation, and/or loss of functionality of AAV. To mitigate loss of AAV due to binding, we pretreated all filters/plasticware with 0.001% Pluronic F-68 as described above. To reduce vector loss due to overconcentration and precipitation, we switched to Amicon Stirred Cell concentrators, which allow for use of higher volumes and continuous mixing during concentration, reducing aggregation and sedimentation. Alternatively, we used Amicon Ultra-15 filter concentrators with frequent (every 2 min of centrifugation) mixing and washing of the filter and did not exceed a total of approximately 2×10^{13} vg of AAV per one concentrator. The resulting process is summarized in Figure 3A qPCR analysis of the amount of AAV found in different fractions of the optimized process indicate high recovery efficiencies at every step, with an overall average purification efficiency of approximately 80% for AAV9 and approximately 65% for PHP.eB (Figure 3B), with a combined overall purification efficiency of approximately 75% (Figure 3C). This is driven by a considerable increase in efficiency at the filter sterilization and buffer exchange steps compared with the non-optimized protocol (Figure 3D). Using the optimized protocol, we obtained an average yield of 2×10^{13} vg per HYPERFlask across multiple vectors packaged with different transgenes, albeit this analysis also includes some vectors with transgenes that lead to lower than average production yields (Figure 3E). After all of the aforementioned modifications to the process were introduced, analysis of AAV loss at each step indicated that less than 5% of AAV is lost in the flow-through or at the filter sterilization steps, while 10% and 20% on average are lost at the buffer exchange and elution steps, respectively (Figure S4), indicating potential targets for future optimization.

The yield and bioactivity of AAVX-HPLC purified AAV are comparable with those of iodixanol purified AAV

To determine whether HPLC purified virus is qualitatively and quantitatively comparable with that of iodixanol ultracentrifugation purified vectors, we compared HPLC purified vectors and iodixanol purified vectors with regard to purity, empty capsid content, *in vitro* bioactivity, and *in vivo* bioactivity. Analysis by gel electrophoresis and SYPRO Ruby red staining indicates that HPLC purified preps are comparable with iodixanol purified preps and consist mainly of the expected VP1–VP3 bands, with little to no unspecific bands present (Figures 4A and S5). Negative-stain transmission electron microscopy (TEM) analysis of the HPLC purified preps indicates an average of approximately 30% empty capsids, which was higher than the approximately 5% empty capsids observed in iodixanol ultracentrifugation purified preps (Figures 4B, 4C, and S7). However, *in vitro* infectivity assay of HEK293 cells indicated that HPLC and iodixanol purified vectors were equally efficient at transducing cells *in vitro*, suggesting that the higher percentage of empty capsids did not have a functional effect on bioactivity (Figures 4D, 4E, and S6).

We observed similar results from our follow-on bioactivity experiments in mice. To compare *in vivo* bioactivity of HPLC and iodixanol purified viruses, we injected a total of 10^{11} vector genomes of self-complementary AAV9 carrying a Cbh-EGFP expression cassette retro-orbitally into 6-week-old wild-type male C57BL/6J mice. We euthanized mice 4 weeks post injection and assayed AAV DNA levels and biodistribution as well as GFP expression in liver, quadriceps, and brain. Transgene DNA, RNA, and protein levels did not significantly differ between AAVX-HPLC and iodixanol purified viruses for any tissues (Figure 5A). To confirm this observation, we sectioned, stained, and imaged livers of injected mice (Figure 5B). Image analysis indicates that EGFP mean fluorescence intensity does not differ significantly between animals injected with AAVX-HPLC and iodixanol purified vectors, and that vectors purified with both methods transduced almost 100% of liver cells (Figures 5C, 5D, S8, and S9). Taken together, these data indicate that AAVX-HPLC purified AAV is comparable in bioactivity with iodixanol ultracentrifugation purified AAV.

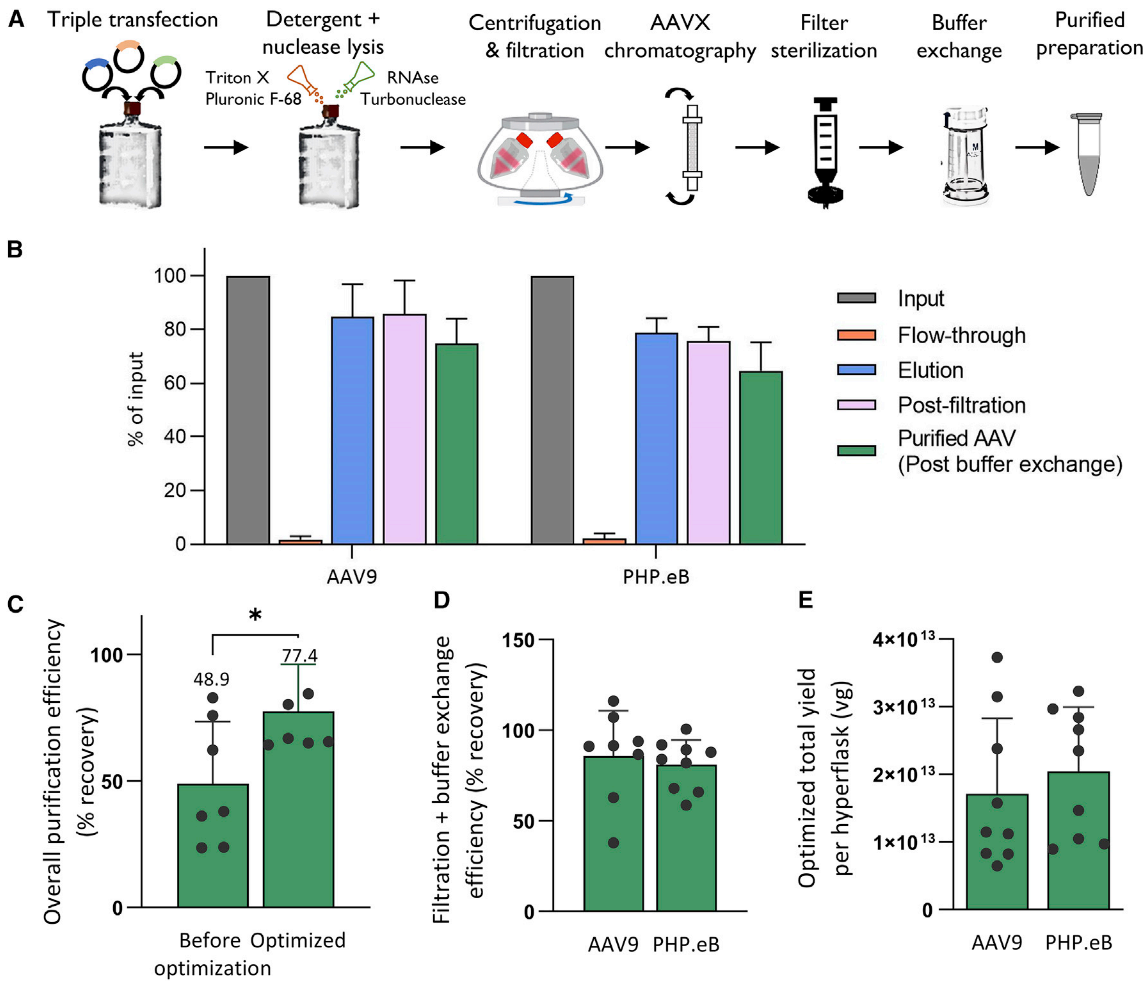

Figure 3. Optimized AAVX affinity purification process

(A) Process steps of the protocol. (B) Stepwise recovery at each step of the purification process. Vector genomes were quantified via qPCR from aliquots of the sample at each process step and represented as normalized to the lysate. N = 6 biological replicates for both AAV9 and PHP.eB. (C) Overall purification efficiencies of the non-optimized and optimized protocols for AAV9 and PHP.eB combined. Difference was assessed using a two-tailed t test, with $^*p < 0.05$. (D) Recovery after filtration + buffer exchange steps for AAV9 and PHP.eB. Note that the values above 100% fall within the range of the approximately 20% precision limit of qPCR titration, and likely do not represent actual recoveries above 100%. (E) Total yields per HYPERFlask across all vectors produced with scAAV9 and scPHP.eB and purified using this protocol. Error bars denote standard deviation in all panels. Note that this includes some vectors that have lower than average production yields. Detailed steps of the purification process are listed in supplemental protocol.

DISCUSSION

An increased demand in AAV research has led to the need to develop more versatile purification methods. Affinity chromatography has been considered a possible solution, but its application to AAV purification has been hampered by the lack of resins or processes that can purify multiple AAV serotypes efficiently without individual optimization.[10–13]

The main advantages of chromatographic purification are its scalability to larger volumes and reduced requirement for hands-on time, which considerably decreases costs and eases AAV manufacturing. Chromatographic resins can be scaled to high volumes, which enable input of unconcentrated large volumes of lysates. The process can also be automated and precisely controlled, monitored, and quantified, which eases troubleshooting and provides rich data about the quality of the run. For these reasons, chromatography-based methods have become the main workhorse for industrial production of biologics and small molecules.[9] We find that AAVX affinity chromatography allows for purification of multiple AAV serotypes at multiple scales, is efficient, and results in vectors of comparable yield and bioactivity with ultracentrifugation purified vectors.

A possible disadvantage of using an AAVX affinity chromatography (or any other type of affinity chromatography) is the possibility that

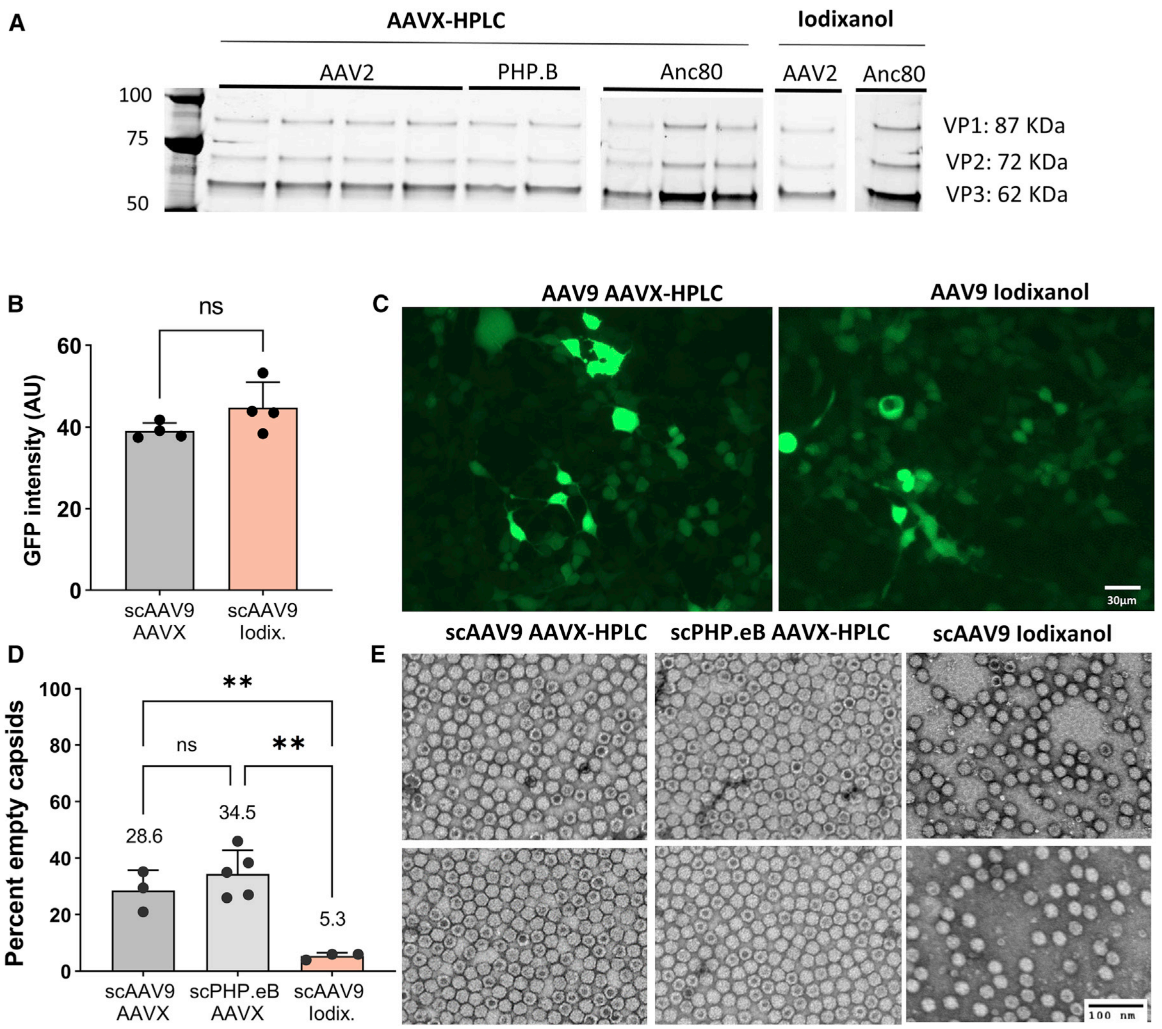

Figure 4. Quality and *in vitro* bioactivity of AAVX affinity-purified AAV

(A) SYPRO Ruby-stained protein gel analysis of AAVX-HPLC versus iodixanol ultracentrifugation purified vectors. Most preps show clear, distinct VP1–VP3 bands, with few non-specific bands present, indicating comparable purity with iodixonal purified virus. (B) Quantification empty capsid content using negative stained TEM. Approximately N = 200 particles were counted for each prep from two separate images by two blinded researchers. Statistical significance was assessed using one-way ANOVA with follow-on Tukey's multiple comparisons test. (C) Representative micrographs of AAVX and iodixanol purified preps used to perform the quantification, with two representative images shown for each. (D) *In vitro* infectivity of AAVX and iodixanol ultracentrifugation purified scAAV9 preps on HEK293 cells. Statistical significance was assessed using two-tailed t test. (E) Representative images used to perform the quantification in (D). **p < 0.01; ns (not significant), p > 0.05. Error bars denote standard deviation in all panels. See also Figures S5–S7 for full images of SYPRO Ruby gels, GFP micrographs, and TEM micrographs, respectively.

new and uncharacterized capsids may not bind to the resin. It is indeed possible that AAV variants that have substantial changes at the AAVX antibody binding site may have low or no affinity to the resin. This can be a particular concern for purification of libraries of diverse AAV capsid variants, for which ultracentrifugation-based methods may be more suitable. For purification of single AAV serotypes, however, this concern can be tested either experimentally or by identifying whether any of the expected changes of the novel capsid alter the AAVX binding epitope once it is definitively mapped. Nevertheless, the majority of basic and clinical research so far has been conducted with AAV capsids that we have verified to bind to AAVX in this work.[4] As such, an AAVX affinity chromatography-based process should be broadly useful for most researchers in the field.

Another potential drawback of chromatographic purification is the co-purification of empty capsids. Indeed, several reports have described empty capsid co-purification to various degrees with affinity and other types of chromatography.[12,13,27] In this study, we found the percentage of empty capsids in AAV9 and PHP.eB preps purified using AAVX affinity chromatography to be approximately 30%, compared with approximately 5% empty capsids in AAV9

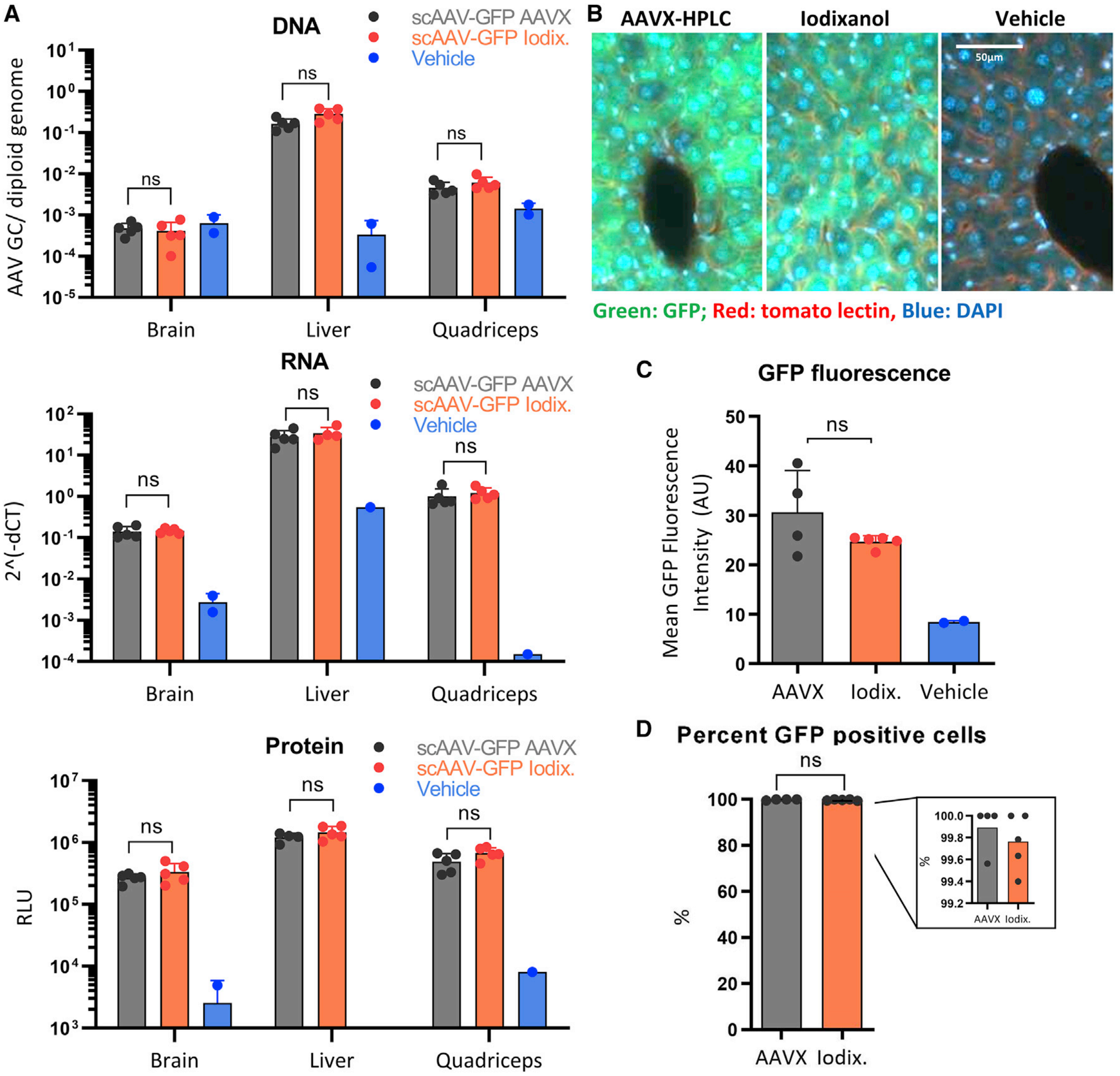

Figure 5. *In vivo* bioactivity of AAVX-HPLC and iodixanol ultracentrifugation purified AAV

(A) Quantification of viral DNA and GFP RNA and protein levels in the liver, brain, and quadriceps of mice injected with a total of 10^{11} vg/mouse of scAAV9-Cbh-GFP. N = 5 for both scAAV-GFP AAVX and scAAV-GFP iodixinal injected mice, and N = 2 for vehicle-injected mice. DNA and RNA were quantified using qPCR and qRT-PCR, respectively, and protein using Simple Wes. Statistical significance was assessed using two-way ANOVA with Šídák's post hoc tests. Statistically non-significant differences are not shown on the figure, except for AAVX versus iodixanol groups. Note that the AAV DNA levels in the brain were likely below the limit of quantification in this assay. (B–D) Imaging analysis of livers sectioned, stained for tomato lectin and DAPI, and imaged for native GFP fluorescence, tomato lectin, and DAPI. (C) Comparison of native GFP averaged from 400–700 cells per animal. (D) Percentage of cells that are GFP positive, counted as cells with a higher mean fluorescence intensity than the highest mean fluorescence intensity observed in the vehicle group. Statistical significance was assessed using one-way ANOVA with Tukey's post hoc test for (C) and two-tailed t test for (D). ns, $p > 0.05$; *$p < 0.05$, **$p < 0.01$, ***$p < 0.001$. Error bars denote standard deviation in all panels.

preps purified using iodixanol ultracentrifugation (Figures 4B and 4C), suggesting that HPLC purification did not enrich for full capsids to the extent of iodixanol ultracentrifugation, if at all. However, despite the higher level of empty capsids, AAVX purified preps showed equivalent bioactivity to iodixanol purified preps both *in vitro* and *in vivo* (Figures 4D, 4E, and 5). Additionally, we have since carried out over 30 animal studies using AAVX-HPLC purified AAV and have observed satisfactory gene transfer in all of them (data not shown), indicating that the higher empty capsid content does not have an overt negative effect on efficacy.

However, for applications where maximal reduction of empty capsid content is required, various upstream or downstream steps that reduce the production of empty capsids or enrich for full capsids

can be added. These include: optimization of plasmid transfection ratios;[28] use of vector plasmids that are full length or with minimal inverted terminal repeat (ITR) deletion;[28] use of novel engineered ITRs; use of a transfection plasmid containing both the AAV cap and transgene in *cis*;[28] or other methods which have been reported to reduce the fraction of empty capsids in the input lysate. While we did not explore this in the present work, multiple different downstream steps to enrich for full capsids utilizing size exclusion, anion exchange, or other chromatographic methods have been recently reported.[10,11,13,29–35] These can be added in series as additional steps to the process after the AAVX affinity binding step.

It should be noted that we estimated the empty capsid percentage in our preps using negative-stain TEM. Electron microscopy has the advantage of producing a clear visual of the AAV particle populations present, and when performed rigorously can match the results of analytical ultracentrifugation.[36] While we performed the analysis based on published guidelines[36] using two independent blinded operators, this method can nevertheless suffer from potential image noise, staining artifacts, or experimenter subjectivity at quantification.[37] Therefore, future studies are needed to assess the impact of these methods on empty/full ratios of the yielded preparation including, e.g., analytical ultracentrifugation.

Using AAVX, we aimed to develop an integrated purification process for preps of at least 10^{14} vg. We found the main bottlenecks to be efficient cell lysis in the upstream process, and the loss or sedimentation of AAV at the buffer exchange step in the downstream process. To mitigate these, we incorporated *in situ* cell lysis using detergents and nucleases in the upstream process and buffer exchange using Amicon Stirred Cell in the downstream process (see "an optimized purification protocol"). These and other modifications increased process-wide efficiencies (from clarified lysate to purified preparation) to an average of approximately 75% while allowing resin re-use without loss of efficiency for at least six purification cycles (Figures 3C and S3). Additionally, we observed consistently high binding efficiencies between different batches of AAVX resin (Figures 2, S2, and S3) and consistently high overall purification efficiencies (Figures 3C and 3D) across all serotypes tested. This indicates that batch-to-batch variability of AAVX is low and that the protocol is overall robust and reproducible.

In summary, affinity chromatography with POROS CaptureSelect AAVX resin allows for high-efficiency purification of various AAV serotypes at multiple scales. The process developed here is primarily increased throughput and versatility applicable to laboratory studies. For clinical and/or scaled applications, further characterization on empty capsid content and elimination of the lysate clearance by centrifugation (e.g., by depth filtration or tangential-flow filtration) is needed. Here, we demonstrate the utility of AAVX in a cost- and time-effective process that does not require process modifications dependent on the serotype, thus being ideally suited for laboratory studies or centralized core facilities.

MATERIALS AND METHODS

AAV production and purification

All AAV vectors were produced in HEK293 cells via the triple plasmid transient transfection method as described previously.[6] For small-scale preps (Figures 2A and S2), HEK293 cells were seeded in 15-cm dishes and grown to 80% confluence in Dulbecco's modified Eagle's medium (DMEM) containing 10% fetal bovine serum (FBS) (Gibco, 26140079) and 1% PenStrep (Thermo Fisher Scientific, 15140122). Cells were then triple transfected with the vector, AAV1 Rep/Cap (Addgene, 112862), and Ad helper plasmid (pAd delta F6 from UPenn) at a ratio of 1:1:2 (13:13:26 μg per 15-cm dish) using PEI Max 40000, pH 7.1 (Polysciences, 24765-1) at a ratio 1.375:1 of PEI/total DNA. Cells were harvested 3 days post transfection by scraping cells off the plate in their conditioned medium and lysing cells through 3× freeze-thaw cycles between 37°C and −80°C. Preps from three replicate plates were then pooled, incubated with 25 U/mL of benzonase (Millipore Sigma, E8263-25KU) at 37°C for 1 h to remove plasmid and cell DNA, centrifuged at 4°C and 4,000 × *g* for 30 min, and the supernatant filtered through a 0.22-μm polyethersulfone (PES) bottle-top filter (Corning, 431097). The filtered lysate was then split into two equal parts, with one part purified using standard HPLC purification reagents and the other part purified using reagents containing 0.1% v/v Pluronic F-68 (Thermo Fisher Scientific, 24040032) (described in Figures 2A and S2, respectively).

For HYPERFlask scale preps described in Figures 1C–1E, HEK293 cells at 80% confluence from four 15-cm dishes were seeded to a HYPERFlask (Millipore Sigma, CLS10031-4EA), grown to 80% confluence, and triple transfected with AAV vector Rep/Cap for AAV2, Anc80, PHP.eB, or AAV9 (AAV2: Addgene, 104963; Anc80: Zinn et al.;[17] PHP.eB: Addgene, 103005; AAV9: Addgene, 112865), and pAdΔF6 at 130:130:260 μg per HYPERFlask, respectively. Three days after transfection cells were lysed, and clarified harvests (560 mL) were treated with 12,500 total units of benzonase (Millipore Sigma, E8263-25KU) for 30 min at 37°C, and this step was repeated with an additional 2,500 total units of benzonase for a further 1 h at 37°C to remove plasmid and cell DNA. The harvest was precipitated overnight at 4°C in high salt solution (80 mL of 5 M NaCl). The clarified lysate was obtained by centrifugation at 4,000 × *g* for 30 min at 4°C. The supernatant was collected and filtered using a 0.22-μm PES filter unit (130 mm diameter filter, Foxx Life Sciences, 1103-RLS) before HPLC purification. Centrifugation for lysate clarification was performed for 30–60 min at 4,000–10,000 × *g*. Ultracentrifugation was performed at 200,000–350,000 × *g* for 90–120 min (using the T70i rotor).

Iodixanol ultracentrifugation purified preps were produced in the Gene Transfer Vector Core at Schepens Eye Research Institute. HEK293 cells were seeded and transfected into HYPERFlasks, followed by benzonase (Sigma-Aldrich, E8263) treatment and high salt lysis as described above. The lysate was then clarified, concentrated using tangential-flow filtration, and purified via iodixanol gradient ultracentrifugation and buffer exchange with FFB (1× PBS, 172 mM NaCl, 0.001% Pluronic F-68).

Static binding assay

Preparations of AAV2, AAV2_HSPG, AAV4, AAV5, AAV6.2, AAV7, AAV8, AAV9, AAVrh10, AAVrh32.33, AAV-PHP.B, Anc80, and AAV7m8 were produced and purified via ultracentrifugation on an iodixanol gradient as described above. To perform the static binding assay, the AAVX resin was first conditioned through three washes in 0.1 M NaCl (4 mL, in a 5-mL Eppendorf tube) and equilibrated through incubation in PBS. To perform the washes, resin was pulse centrifuged to pellet the resin and discard the supernatant in 5-mL Eppendorf tubes. Next, 50 μL of resin was suspended in 1 mL of PBS and 0.001% Pluronic F68, and 5×10^{10} vg of AAV was added. Each AAV serotype was added to a separate tube of unused resin. AAV was then incubated with the resin by rocking at room temperature for 10 min in 1.5-mL Eppendorf tubes. Flow-through was collected by pulse spinning and collecting the supernatant, and resin was washed thrice using 1 mL of PBS, with wash fractions collected. Finally, AAV was eluted twice using 1 mL of 0.1 M citric acid (pH 2) and AAV vector genomes quantified in each fraction using qPCR.

High-efficiency purification protocol

For HYPERFlask scale preps described in Figure 3, an optimized protocol based on Florencio et al.[26] and our own observations were used. HEK293 cells at 80% confluence from four 15-cm dishes were seeded to a HYPERFlask, grown to 80% confluence (normally approximately 48 h after seeding), and triple transfected with AAV vector Rep/Cap for AAV9 or PHP.eB and pAdΔF6 at 130:130:260 μg per HYPERFlask, respectively. Four days post transfection, supernatant from a HYPERFlask was decanted into a 1-L flask and 3 mL of Triton X-100 (Millipore Sigma, 8787-100ML), 2.5 mg of RNAse A at 1 mg/mL concentration (Millipore Sigma, 10109142001), 25 U/mL Turbonuclease (VitaScientific, ACGC80007), and 56 μL of 10% Pluronic F-68 (Thermo Fisher Scientific, 24040032) were added to the supernatant. The contents were then mixed and then poured back into the HYPERFlask, and the HYPERFlask was shaken on an orbital shaker at 150 rpm at 37°C for 1 h to lyse the cells and remove plasmid DNA. Lysate was then decanted from the HYPERFlask, and the HYPERFlask was washed with 140 mL of Dulbecco's PBS (Life Tech, 10010072), which was added to the rest of the lysate. The total lysate was then centrifuged at $4{,}000 \times g$ at 4°C for 30 min, and the supernatant was filtered through a 0.45-μm PES bottle-top filter (Thermo Fisher Scientific, 295-4545) before loading onto the HPLC system. Here, we used a 0.45-μm PES bottletop filter as opposed to a 0.22-μm filter that we used in the unoptimized protocol, because the 0.45-μm filter allowed for a much faster filtration and did not negatively affect follow-on HPLC purification.

High-performance liquid chromatography

AAV purification was performed using AAVX POROS CaptureSelect (Thermo Fisher Scientific) resin bought as pre-packed 1-mL columns (Thermo Fisher Scientific, A36652) or free AAVX resin (Thermo Fisher Scientific, A36741) packed into 6.6 × 100-mm column (glass Omnifit; Kinesis USA). Columns were attached to an AKTA Pure 25 L HPLC system (GE Life Sciences, 29018224) containing an auxiliary sample pump S9 (GE Life Sciences, 29027745). The machine was housed at room temperature and all purifications were performed at room temperature (approximately 24°C), except for experiments described in Figure 3D. Column volume ([CV]) for each purification was set as 1 mL regardless of the actual volume of the resin used. For purifications using more than 1 mL of resin, a protocol with increased wash times was employed (see supplemental files). The chromatography column was pre-equilibrated with 10 [CV] of wash buffer 1× TBS (Boston Bioproducts) before application of AAV lysate. Equilibration and all subsequent washes of the column were performed at a rate of 2 mL/min.

Lysate was clarified at most 1 day prior to loading onto the HPLC and warmed up to room temperature prior to loading. Lysate was loaded at a flow-rate-to-resin-volume ratio ensuring approximately 2 min residence time in the resin, normally using 1 mL of resin and a flow rate of 0.5 mL/min, or 4 mL resin with a flow rate of 2 mL/min. At least 1 mL of resin per one HYPERFlask was used; if preps from multiple HYPERFlasks were pooled together, the volume of resin was increased accordingly.

For purifications using 1 mL of resin, the column containing bound AAV was then washed with 10 [CV] of 1× TBS, followed by washes of 5 [CV] of 2× TBS, 10 [CV] 20% ethanol, and 10 [CV] 1× TBS wash. The bound AAV was eluted using a low-pH (pH 2.5–2.9) buffer of 0.2 M glycine in 1× TBS, containing 0.01% (v/v) Pluronic F-68 at a rate of 1 mL/min. Resin was then washed with 10 [CV] of 1× TBS regenerated with 15 [CV] 0.1 M phosphoric acid (pH 1) and 15 [CV] 6 M guanidine-HCl at flow rate of 1 mL/min, and washed again with 10 [CV] 20% ethanol and 10 [CV] 1× TBS. Elution fractions were taken as 1-mL volumes per fraction. The eluted vector solution was neutralized by adding 1 M Tris-HCl (pH 8.0) at one-tenth of the fraction volume directly into the fraction collection tube prior to elution. Peak fractions based on UV (280 nm) absorption graphs were collected, filter sterilized using 0.2-μm PES syringe filters (Corning, 431229), buffer exchanged using either Amicon Ultracel 15 (Merck Millipore, UFC910008) or Amicon Stirred Cell (Merck Millipore, UFSC05001) concentrators with a molecular weight cutoff of 50 kDa or 100 kDa (Millipore, UFC905008 EMD) prior to virus titration. For Amicon Stirred Cell concentrator, high-purity nitrogen gas (NI UHP80 Airgas) was used at 40–70 psi as a pressure source. All plasticware and tips were coated or incubated with FFB for approximately 15 min at room temperature prior to applying AAV-containing solutions at any step of the purification process.

Quantitative PCR and digital droplet PCR

In brief, genomic titer was determined by a qPCR (TaqMan, Life Technologies) as well as digital droplet PCR (ddPCR). For qPCR, real-time qPCR (7500 Real-Time PCR System; Applied Biosystems, Foster City, CA, USA) with EGFP-targeted primer-probes (AGC AAA GAC CCC AAC GAG AA, GGC GGC GGT CAC GAA, 6FAM-CGC GAT CAC ATG GTC CTG CTG G-TAMRA) were used. We used linearized CBA-EGFP DNA at a series of dilutions of known concentration as a standard. After 95°C holding stage for 10 s, two-step PCR cycling stage was performed at 95°C for 5 s,

followed by 60°C for 5 s for 40 cycles. Genomic vector titers were interpolated from the standard. qPCR was used to determine titers for experiments described in Figures 1, 2, 3, and S2–S6.

For ddPCR, QX200 ddPCR system (Bio-Rad) using the same EGFP-targeted primer-probes as described above were used. ddPCR and titer estimation was performed as previously described by Sanmiguel et al.[38] ddPCR was used to estimate titers of the vectors for experiments described in Figures 4A, 4B, 5, S7, and S8.

Protein gel analysis

All materials and reagents used were purchased from Life Technologies. Equal vector genomes of AAV were loaded on a NUPAGE 4%–12% Bis-Tris polyacrylamide gel (Life Technologies, Grand Island, NJ) and subjected to electrophoresis at 150 V for 1 h 30 min. For each AAV preparation, a volume corresponding to a titer of 10^{10} vg was mixed with 5 μL of 4× NuPAGE lithium dodecyl sulfate sample buffer and 1× PBS (Corning, 21-031-CM) to 20 μL total volume and heat denatured at 70°C for 5 min.

SYPRO Ruby Protein Gel Stain (Thermo Fisher Scientific) was applied per the manufacturer's protocol to visualize and analyze SDS-PAGE bands. In brief, the gel was fixed in 7% glacial acetic acid and 50% methanol (ACS grade, Thermo Fisher Scientific) in ultrapure water for 15 min at 21°C (room temperature) by gentle agitation. Fixation was repeated once more before gel was rinsed with ultrapure water. Gel was stained with SYPRO Ruby as follows: 30 s microwave, 30 s agitation, 30 s microwave, 5 min agitation, 30 s microwave, 23 min agitation. Gel was rinsed with ultrapure water and destained with 7% glacial acetic acid and 10% methanol for 30 min at 21°C (room temperature) by gentle agitation. Proteins stained with the dye were visualized with a 302-nm UV transilluminator (ChemiDoc XRS + Bio-Rad).

Empty capsid estimation via transmission electron microscopy

Purified and formulated AAV from different preps was diluted to a concentration of 10^{13} vg/mL and submitted for negative stain and TEM analysis at Harvard Medical School Electron Microscopy Core. In brief, the sample was diluted in water and adsorbed onto a glow-discharged carbon or formvar/carbon-coated grid. Once the specimen was adsorbed on to the film surface, the excess liquid was blotted off using a filter paper (Whatman #1) and the grid was floated on a small drop (~5 μL) of staining solution (most commonly 0.75% uranyl formate, 1% uranyl acetate, or 1%–2% phosphotungstatic acid). After 20 s the excess stain was blotted off and the sample was air dried briefly before examination in the transmission electron microscope. At least two images were taken per prep at 30,000× magnification, and at least 200 virions were counted manually per image by two researchers blinded to the identity of the image; empty and full ratios were averaged between resulting counts. Because of the difficulty in confidently differentiating full and partially filled capsids using electron micrographs, virions were counted as empty and full only based on the criteria described in Fu et al.[36] On the minority of cases where a virion could not be confidently assigned to either (<1% capsids), the virion was not counted. Similarly, virions were not counted in areas of images with image noise, artifacts, clumping, or other effects that obscure a clear classification of the virion type.

Next-generation sequencing and analysis

For Figures 2B and S2B, five different AAV1 preps were produced, where the vectors from the second to fifth prep were identical except a unique DNA barcode region. The preps were purified consecutively from prep 1 to prep 5, and the barcode region was PCR amplified in the elution fractions of the fifth preps. The amplicons were PCR amplified and submitted for Amplicon Seq at the MGH DNA Sequencing Core. Finally, the number of barcode reads corresponding to AAVs from each of the preps 2–5 was directly counted from the resulting FASTQ file. The vast majority of barcodes present came from the fifth preps (barcodes from previous preps were present at less than 0.1%).

AAV *in vitro* studies

HEK293 cells were seeded at 1 × 10^5 cells/well, N = 4 replicates, in 500 μL of complete DMEM containing 10% (v/v) heat-inactivated FBS, and 100 U/mL penicillin and 100 μg/mL streptomycin into a 24-well plate, and AAV was added immediately at a multiplicity of infection of 10^5 vg/cell. The vector was self-complementary AAV9 carrying a CBh-GFP expression cassette. Cells were washed with PBS 3 days later and imaged using a Leica Observer D1 microscope, using a 10× objective. Exposure and light power were adjusted such as to place the GFP signal from vehicle transduced cells to the bottom fifth of the signal range.

AAV *in vivo* studies

All animal procedures were performed with the approval of the Institutional Animal Care and Use Committee at Schepens Eye Research Institute. For assaying *in vivo* potency and transduction, self-complementary AAV9 carrying a Cbh-EGFP expression cassette was produced at the HYPERFlask scale and purified with AAVX-HPLC or iodixanol ultracentrifugation, concentrated in FFB, and stored at −80°C until use. Six-week-old male C57BL/6J mice (N = 5 each for cohorts injected with AAVX and iodixanol purified vectors, and N = 2 for vehicle-injected vectors) were then injected retro-orbitally with a total dose of 10^{11} vg (in 100 μL volume of FFB) per mouse. Mice were euthanized 4 weeks post injection and brain, quadriceps, and liver harvested. One part of each tissue was snap frozen in liquid nitrogen for analysis of vector DNA and EGFP RNA and protein (see below). Another part of each tissue was fixed in paraformaldehyde (PFA) for later sectioning and processing for immunofluorescence imaging (see immunofluorescence and image analysis).

DNA, RNA, and protein quantification

Tissues were homogenized by disrupting 30 mg of tissue in 1 mL of RLT+ buffer for DNA and RNA and 1 mL of RIPA buffer containing 1× Halt protease and phosphatase inhibitors for protein (Thermo Fisher Scientific, 78444). For disruption, samples, buffer, and 1-mm zirconia/silica beads (Biospec, 11079110z) were loaded into XXtuff vials (BioSpec, 330TX) and disrupted using Mini Beadbeater 24

(BioSpec,112011) at maximum speed for 3 min. Vials were then placed on ice for 2–5 min for RNA and 1 h for protein, centrifuged at 10,000 × *g* for 3 min, and the resulting supernatant used for further procedures.

For DNA/RNA, 700 μL of supernatant was loaded onto AllPrep DNA Mini Spin columns and purified using AllPrep DNA/RNA/miRNA Universal Kit (Qiagen, 80224) for quadriceps and AllPrep DNA/RNA mini kit (Qiagen, 80204) for brain and liver. Purification was performed on Qiacube Connect (Qiagen, 9002864).

Total AAV genome copy number was assessed by qPCR using GFP primer-probe sets and quantified using linearized CBA-GFP plasmid serial dilutions as the standard for AAV copy number (AGC AAA GAC CCC AAC GAG AA, GGC GGC GGT CAC GAA, 6FAM-CGC GAT CAC ATG GTC CTG CTG G-TAMRA). Total cell genome copy number was estimated using RPII primer-probes (GTT TTC ATC ACT GTT CAT GAT GC, TCA TGG GCA TTA CTA TTC CTA C, probe: VIC-AGG ACC AGC TTC TCT GCA TTA TCA TCG TTG AAG AT-3IABkFQ) along with a standard of gDNA dilution series of known concentration. AAV copy number per diploid genome was then calculated as copy number per diploid genome $= 2\times\left(\frac{\text{total AAV copy number}}{\text{total genome copy number}}\right)$. Efficiency and specificity of amplification for both primer-probe sets was previously established, and amplification was performed using Luna Universal Probe qPCR Master Mix (NEB, M3004L) at thermocycling conditions recommended by the manufacturer.

For quantification of GFP RNA expression, RNA extracted from tissues was first treated with DNAse (DNA-free DNA Removal Kit; Thermo Fisher Scientific, AM1906) and then reverse transcribed and amplified using Luna Universal Probe One-Step qRT-PCR Kit (NEB, E3006L) according to the manufacturer's instructions. Primer-probe sets for GFP cDNA (AGC AAA GAC CCC AAC GAG AA, GGC GGC GGT CAC GAA, 6FAM-CGC GAT CAC ATG GTC CTG CTG G-TAMRA) and RPII cDNA (GTT TTC ATC ACT GTT CAT GAT GC, AAT CAA TGC AGG TTT TGG CGA TG, probe: VIC-AGG ACC AGC TTC TCT GCA TTA TCA TCG TTG AAG AT-3IABkFQ) were used. Controls lacking reverse transcriptase were run to preclude signal from DNA contamination. Expression of GFP RNA normalized to RPII RNA was then calculated as $2^{-(Ct_{GFP}-Ct_{RPII})}$.

For quantification of GFP protein expression, protein lysate was first diluted 5× twice in fresh RIPA + 1× Halt inhibitors buffer, and all dilutions were assayed for total protein content using a Pierce BCA Protein Assay Kit (Thermo Fisher Scientific, 23225). For each tissue type, lysates were diluted in RIPA + 1× Halt buffer to the concentrations of: liver, 0.05 μg/μL; brain, 1.5 μg/μL; quadriceps, 1.5 μg/μL. Protein levels were then assayed using anti-GFP antibody ab290 (Abcam, ab290) on Wes (Protein Simple) with the 12–230 kDa chemiluminescence assay (12–230 kDa Jess or Wes Separation Module; Protein Simple, SM-W004). Linear range for GFP quantification was previously determined by assaying GFP using Wes with ab290 antibody for dilutions ranging from ~5 μg/μL to 0.03 μg/μL (linear range: liver <0.3 μg/μL, brain 0.3 μg/μL to ~3 μg/μL, quadriceps 0.03 μg/μL to ~3 μg/μL). Linear range for total protein was also previously determined by Wes 12–230 kDa Total Protein Size assay in the range of 4 μg/μL to 0.1 μg/μL using Total Protein Detection Module (Protein Simple, DM-TP01) according to the manufacturer's instructions. Linear range was found to be < 1 μg/μL for all tissues tested. GFP and total protein levels were then quantified using Compass for SW 4.1 (Protein Simple). Finally, GFP was normalized to total protein to arrive at the final value.

Immunofluorescence and image analysis

Tissues were fixed in 1% PFA for 4 h and then 4% PFA for 1 h at room temperature (21°C). Fixed tissues were then washed with 1× PBS three times for 5 min, placed in 30% sucrose for approximately 48 h at 4°C, and frozen in OCT blocks by submersion into isopentane cooled by liquid nitrogen. Blocks were then sectioned at 12 μm thickness using iHisto cryosectioning service (iHisto). Sections were kept at −80°C until staining. Sections were blocked using blocking buffer (10% normal goat serum, 2% BSA, 0.1% Tween 20) for 1 h, washed 3 × 5 min with PBS-T (PBS + 0.1% Tween 20), stained with tomato lectin at 10 μg/mL (Vector Laboratories, DL-1177) for 1 h, washed 3 × 5 min with PBS-T, stained with 4′,6-diamidino-2-phenylindole (DAPI) for 5 min at 1:1,000 stock concentration (Thermo Fisher Scientific, D1306), mounted for 15 min (Vector Laboratories, H-1400) and imaged for native GFP, tomato lectin, and DAPI. All actions were performed at 21°C in a dark room. Slides were imaged using a Zeiss Axio Observer D1 microscope (exposure times were set such that signal intensities from samples with the brightest signals would appear in the lower third of the histogram). Exposures were kept constant between all samples for all three colors imaged. For each tissue, two sections from the middle of the tissue were imaged, with 6–8 fields in total imaged at 200× magnification.

Three images of different sites were then selected, all cells within the images circled for regions of interest (ROIs), and cell GFP mean fluorescence intensity quantified within ROIs in Fiji.[39] Cells were circled conservatively to make sure only individual cells were circled. A total of 400–700 cells were quantified per animal, and mean fluorescence intensity values across different cells averaged to arrive at an overall liver GFP mean fluorescence intensity per animal.

AAV phylogenetic analysis

To generate the phylogeny, first 19 representative AAV capsids were chosen, including an avian AAV (VR-865) for use as an outgroup for eventual tree rooting. The VP1 amino acid sequences from all of these different isolates were aligned through ClustalOmega[40] as implemented on the EMBL-EBI webserver.[41] Substitutions models and parameters for an eventual maximum likelihood (ML) phylogenic analysis were evaluated by ProtTest3,[42] and the best-fitting model by the Aikake Information Criterion was selected. The model best describing the set of AAV sequences was the Le and Gascuel model,[43] with a discrete Gamma distribution (five categories) to model rate

differences among sites within the alignment. This model was used to construct an ML phylogeny through MEGA X[44] before being exported and visualized through phytools.[45] See Figures S10–S12 for multiple sequence alignment, sequence percent identity, and Newick formatted phylogeny of the phylogeny depicted in Figure 1B.

Statistical analysis

All data were visualized, and statistical analysis was performed using GraphPad Prism (GraphPad). Specific statistical tests used are listed in figure legends for each test, and all tests were performed with default settings unless otherwise specified.

DATA AVAILABILITY

All data will be made available upon request.

SUPPLEMENTAL INFORMATION

Supplemental information can be found online at https://doi.org/10.1016/j.omtm.2022.12.009.

ACKNOWLEDGMENTS

We thank Ru Xiao, Allison Cucalon, Abigail Sheridan, and Ruchi Chauhan from the Grousbeck Gene Transfer Vector Core for assistance and advice with AAV production, Jennifer Santos-Franceschini and Reynette Estelien from the Vandenberghe lab for assistance with mouse work, Maria Ericsson from Harvard Medical School Electron Microscopy Facility for advice and assistance with TEM, David Anderson from Wagers lab for help with electron microscopy analysis, and Alejandro Becerra from Thermo Fisher Scientific for helpful discussions and suggestions for increasing AAVX resin reuse capacity and elution efficiencies. This work was supported in part by grants from the Paul F. Glenn Foundation and NIH DP1 AG063419 (to A.J.W.).

AUTHOR CONTRIBUTIONS

S.P. and F.N. performed the static binding assay and established HPLC purification parameters in the unoptimized protocol. F.N. produced, purified, and tested purification efficiencies, capsid purity, and *in vitro* infectivity of AAV2 and Anc80, and M.F. did likewise for AAV9 and PHP.eB in Figure 1. E.M.Z. conducted AAV phylogenetic analysis. M.F. conducted empty capsid analysis. M.F. produced, purified, and quantified AAV1 for small-scale purification experiments and AAV9 and PHP.eB for large-scale experiments in Figures 2 and 3. M.F. and C.U. conducted *in vivo* gene transfer assays. M.F. optimized AAV purification for large-scale high-efficiency protocols. J.S. titered vectors described in Figures 2, 4, and S6. M.F., F.N., L.H.V., S.P., E.A.-M., A.J.W., and C.U. conceived and planned the experiments. M.F. and L.H.V. wrote the manuscript, and M.F., L.H.V., A.J.W., F.N., E.A.-M., and C.U. edited the manuscript.

DECLARATION OF INTERESTS

L.H.V. is an employee of ciendias bio and is a paid advisor to Novartis, Akouos, and Affinia Therapeutics, and serves on the Board of Directors of ciendias bio, Affinia, Addgene, and Odylia Therapeutics. L.H.V. holds equity in ciendias bio, Akouos, and Affinia and receives sponsored research funding from Albamunity Inc., to which he was an unpaid consultant. L.H.V. is an inventor on various patents licensed to multiple biopharmaceutical companies that are relevant to gene therapy including AAV manufacturing. M.F., A.J.W., and L.H.V. are named inventors on a patent covering some methods described in this work. E.A.-M. is an employee and holds equity in Akouos Inc. A.J.W. is a scientific advisor for Kate Therapeutics and Frequency Therapeutics and a co-founder, advisor, and holder of private equity in Elevian, Inc., which also provides sponsored research to the Wagers lab. A.J.W. and M.F. are inventors on various patents that include the use of AAVs for research and therapeutic applications. F.N. is an employee at Takeda Pharmaceutical Company Limited.

REFERENCES

1. Atchison, R.W., Casto, B.C., and Hammon, W.M. (1965). Adenovirus-associated defective virus particles. Science *149*, 754–756.
2. Hoggan, M.D., Blacklow, N.R., and Rowe, W.P. (1966). Studies of small DNA viruses found in various adenovirus preparations: physical, biological, and immunological characteristics. Proc. Natl. Acad. Sci. USA *55*, 1467–1474.
3. Samulski, R.J., and Muzyczka, N. (2014). AAV-mediated gene therapy for research and therapeutic purposes. Annu. Rev. Virol. *1*, 427–451.
4. Ginn, S.L., Amaya, A.K., Alexander, I.E., Edelstein, M., and Abedi, M.R. (2018). Gene therapy clinical trials worldwide to 2017: an update. J. Gene Med. *20*, e3015.
5. Crosson, S.M., Dib, P., Smith, J.K., and Zolotukhin, S. (2018). Helper-free production of laboratory grade AAV and purification by iodixanol density gradient centrifugation. Mol. Ther. Methods Clin. Dev. *10*, 1–7.
6. Lock, M., Alvira, M., Vandenberghe, L.H., Samanta, A., Toelen, J., Debyser, Z., and Wilson, J.M. (2010). Rapid, simple, and versatile manufacturing of recombinant adeno-associated viral vectors at scale. Hum. Gene Ther. *21*, 1259–1271.
7. Strobel, B., Miller, F.D., Rist, W., and Lamla, T. (2015). Comparative analysis of cesium chloride- and iodixanol-based purification of recombinant adeno-associated viral vectors for preclinical applications. Hum. Gene Ther. Methods *26*, 147–157.
8. Clément, N., and Grieger, J.C. (2016). Manufacturing of recombinant adeno-associated viral vectors for clinical trials. Mol. Ther. Methods Clin. Dev. *3*, 16002.
9. Łącki, K.M., and Riske, F.J. (2020). Affinity chromatography: an enabling technology for large-scale bioprocessing. Biotechnol. J. *15*, 1800397.
10. Rieser, R., Koch, J., Faccioli, G., Richter, K., Menzen, T., Biel, M., Winter, G., and Michalakis, S. (2021). Comparison of different liquid chromatography-based purification strategies for adeno-associated virus vectors. Pharmaceutics *13*, 748.
11. Terova, O., Soltys, S., Hermans, P., De Rooij, J., and Detmers, F. (2018). Overcoming downstream purification challenges for viral vector manufacturing: enabling advancement of gene therapies in the clinic. Cell Gene Therapy Insights *4*, 101–111.
12. Potter, M., Lins, B., Mietzsch, M., Heilbronn, R., Van Vliet, K., Chipman, P., Agbandje-McKenna, M., Cleaver, B.D., Clément, N., Byrne, B.J., et al. (2014). A simplified purification protocol for recombinant adeno-associated virus vectors. Mol. Ther. Methods Clin. Dev. *1*, 14034.
13. Nass, S.A., Mattingly, M.A., Woodcock, D.A., Burnham, B.L., Ardinger, J.A., Osmond, S.E., Frederick, A.M., Scaria, A., Cheng, S.H., and O'Riordan, C.R. (2018). Universal method for the purification of recombinant AAV vectors of differing serotypes. Mol. Ther. Methods Clin. Dev. *9*, 33–46.
14. Wang, Q., Lock, M., Prongay, A.J., Alvira, M.R., Petkov, B., and Wilson, J.M. (2015). Identification of an adeno-associated virus binding epitope for AVB sepharose affinity resin. Mol. Ther. Methods Clin. Dev. *2*, 15040.
15. Deverman, B.E., Pravdo, P.L., Simpson, B.P., Kumar, S.R., Chan, K.Y., Banerjee, A., Wu, W.-L., Yang, B., Huber, N., Pasca, S.P., and Gradinaru, V. (2016). Cre-dependent selection yields AAV variants for widespread gene transfer to the adult brain. Nat. Biotechnol. *34*, 204–209.
16. Limberis, M.P., Vandenberghe, L.H., Zhang, L., Pickles, R.J., and Wilson, J.M. (2009). Transduction efficiencies of novel AAV vectors in mouse airway epithelium in vivo and human ciliated airway epithelium in vitro. Mol. Ther. *17*, 294–301.

17. Zinn, E., Pacouret, S., Khaychuk, V., Turunen, H.T., Carvalho, L.S., Andres-Mateos, E., Shah, S., Shelke, R., Maurer, A.C., Plovie, E., et al. (2015). In silico reconstruction of the viral evolutionary lineage yields a potent gene therapy vector. Cell Rep. *12*, 1056–1068.

18. Gao, G.P., Alvira, M.R., Wang, L., Calcedo, R., Johnston, J., and Wilson, J.M. (2002). Novel adeno-associated viruses from rhesus monkeys as vectors for human gene therapy. Proc. Natl. Acad. Sci. USA *99*, 11854–11859.

19. Lin, J., Calcedo, R., Vandenberghe, L.H., Bell, P., Somanathan, S., and Wilson, J.M. (2009). A new genetic vaccine platform based on an adeno-associated virus isolated from a rhesus macaque. J. Virol. *83*, 12738–12750.

20. Dalkara, D., Byrne, L.C., Klimczak, R.R., Visel, M., Yin, L., Merigan, W.H., Flannery, J.G., and Schaffer, D.V. (2013). In vivo directed evolution of a new adeno-associated virus for therapeutic outer retinal gene delivery from the vitreous. Sci. Transl. Med. *5*, 189ra76.

21. Chiorini, J.A., Kim, F., Yang, L., and Kotin, R.M. (1999). Cloning and characterization of adeno-associated virus type 5. J. Virol. *73*, 1309–1319.

22. Chiorini, J.A., Yang, L., Liu, Y., Safer, B., and Kotin, R.M. (1997). Cloning of adeno-associated virus type 4 (AAV4) and generation of recombinant AAV4 particles. J. Virol. *71*, 6823–6833.

23. Vandenberghe, L.H., Wang, L., Somanathan, S., Zhi, Y., Figueredo, J., Calcedo, R., Sanmiguel, J., Desai, R.A., Chen, C.S., Johnston, J., et al. (2006). Heparin binding directs activation of T cells against adeno-associated virus serotype 2 capsid. Nat. Med. *12*, 967–971.

24. Bennicelli, J., Wright, J.F., Komaromy, A., Jacobs, J.B., Hauck, B., Zelenaia, O., Mingozzi, F., Hui, D., Chung, D., Rex, T.S., et al. (2008). Reversal of blindness in animal models of leber congenital amaurosis using optimized AAV2-mediated gene transfer. Mol. Ther. *16*, 458–465.

25. Patrício, M.I., Cox, C.I., Blue, C., Barnard, A.R., Martinez-Fernandez de la Camara, C., and MacLaren, R.E. (2020). Inclusion of PF68 surfactant improves stability of rAAV titer when passed through a surgical device used in retinal gene therapy. Mol. Ther. Methods Clin. Dev. *17*, 99–106.

26. Dias Florencio, G., Precigout, G., Beley, C., Buclez, P.O., Garcia, L., and Benchaouir, R. (2015). Simple downstream process based on detergent treatment improves yield and in vivo transduction efficacy of adeno-associated virus vectors. Mol. Ther. Methods Clin. Dev. *2*, 15024.

27. Burova, E., and Ioffe, E. (2005). Chromatographic purification of recombinant adenoviral and adeno-associated viral vectors: methods and implications. Gene Ther. *12*, S5–S17.

28. Savy, A., Dickx, Y., Nauwynck, L., Bonnin, D., Merten, O.W., and Galibert, L. (2017). Impact of inverted terminal repeat integrity on rAAV8 production using the baculovirus/sf9 cells system. Hum. Gene Ther. Methods *28*, 277–289.

29. Wang, C., Mulagapati, S.H.R., Chen, Z., Du, J., Zhao, X., Xi, G., Chen, L., Linke, T., Gao, C., Schmelzer, A.E., and Liu, D. (2019). Developing an anion exchange chromatography assay for determining empty and full capsid contents in AAV6.2. Mol. Ther. Methods Clin. Dev. *15*, 257–263.

30. Khatwani, S.L., Pavlova, A., and Pirot, Z. (2021). Anion-exchange HPLC assay for separation and quantification of empty and full capsids in multiple adeno-associated virus serotypes. Mol. Ther. Methods Clin. Dev. *21*, 548–558.

31. Joshi, P.R.H., Bernier, A., Moço, P.D., Schrag, J., Chahal, P.S., and Kamen, A. (2021). Development Of A Scalable And Robust AEX Method For Enriched rAAV Preparations In Genome-Containing Vcs Of Serotypes 5, 6, 8, And 9. Mol. Therapy - Methods & Clinical Dev. *21*, 341–356.

32. Urabe, M., Xin, K.Q., Obara, Y., Nakakura, T., Mizukami, H., Kume, A., Okuda, K., and Ozawa, K. (2006). Removal of empty capsids from type 1 adeno-associated virus vector stocks by anion-exchange chromatography potentiates transgene expression. Mol. Ther. *13*, 823–828.

33. Qu, G., Bahr-Davidson, J., Prado, J., Tai, A., Cataniag, F., McDonnell, J., Zhou, J., Hauck, B., Luna, J., Sommer, J.M., et al. (2007). Separation of adeno-associated virus type 2 empty particles from genome containing vectors by anion-exchange column chromatography. J. Virol. Methods *140*, 183–192.

34. McIntosh, N.L., Berguig, G.Y., Karim, O.A., Cortesio, C.L., De Angelis, R., Khan, A.A., Gold, D., Maga, J.A., and Bhat, V.S. (2022). Comprehensive characterization and quantification of adeno associated vectors by size exclusion chromatography and multi angle light scattering. Sci. Rep. *12*, 19754.

35. Gagnon, P., Leskovec, M., Prebil, S.D., Žigon, R., Štokelj, M., Raspor, A., Peljhan, S., and Štrancar, A. (2021). Removal of empty capsids from adeno-associated virus preparations by multimodal metal affinity chromatography. J. Chromatogr. A *1649*, 462210.

36. Fu, X., Chen, W.C., Argento, C., Clarner, P., Bhatt, V., Dickerson, R., Bou-Assaf, G., Bakhshayeshi, M., Lu, X., Bergelson, S., and Pieracci, J. (2019). Analytical strategies for quantification of adeno-associated virus empty capsids to support process development. Hum. Gene Ther. Methods *30*, 144–152.

37. Gimpel, A.L., Katsikis, G., Sha, S., Maloney, A.J., Hong, M.S., Nguyen, T.N.T., Wolfrum, J., Springs, S.L., Sinskey, A.J., Manalis, S.R., et al. (2021). Analytical methods for process and product characterization of recombinant adeno-associated virus-based gene therapies. Mol. Ther. Methods Clin. Dev. *20*, 740–754.

38. Sanmiguel, J., Gao, G., and Vandenberghe, L.H. (2019). Quantitative and digital droplet-based AAV genome titration. Methods Mol. Biol. *1950*, 51–83.

39. Schindelin, J., Arganda-Carreras, I., Frise, E., Kaynig, V., Longair, M., Pietzsch, T., Preibisch, S., Rueden, C., Saalfeld, S., Schmid, B., et al. (2012). Fiji: an open-source platform for biological-image analysis. Nat. Methods *9*, 676–682.

40. Sievers, F., Wilm, A., Dineen, D., Gibson, T.J., Karplus, K., Li, W., Lopez, R., McWilliam, H., Remmert, M., Söding, J., et al. (2011). Fast, scalable generation of high-quality protein multiple sequence alignments using Clustal Omega. Mol. Syst. Biol. *7*, 539.

41. Goujon, M., McWilliam, H., Li, W., Valentin, F., Squizzato, S., Paern, J., and Lopez, R. (2010). A new bioinformatics analysis tools framework at EMBL-EBI. Nucleic Acids Res. *38*, W695–W699.

42. Darriba, D., Taboada, G.L., Doallo, R., and Posada, D. (2011). ProtTest 3: fast selection of best-fit models of protein evolution. Bioinformatics *27*, 1164–1165.

43. Le, S.Q., and Gascuel, O. (2008). An improved general amino acid replacement matrix. Mol. Biol. Evol. *25*, 1307–1320.

44. Kumar, S., Stecher, G., Li, M., Knyaz, C., and Tamura, K. (2018). Mega X: molecular evolutionary genetics analysis across computing platforms. Mol. Biol. Evol. *35*, 1547–1549.

45. Revell, L.J. (2012). phytools: an R package for phylogenetic comparative biology (and other things. Methods Ecol. Evol. *3*, 217–223.

OMTM, Volume *28*

Supplemental information

High-efficiency purification of divergent AAV serotypes using AAVX affinity chromatography

Michael Florea, Fotini Nicolaou, Simon Pacouret, Eric M. Zinn, Julio Sanmiguel, Eva Andres-Mateos, Carmen Unzu, Amy J. Wagers, and Luk H. Vandenberghe

Supplemental Figures

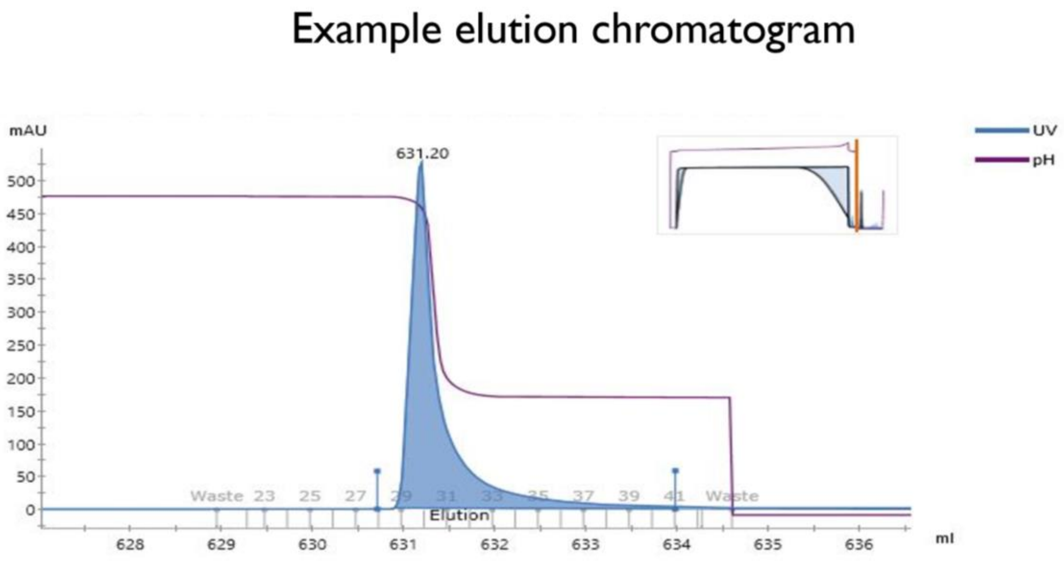

Figure S1. HPLC chromatogram of AAV2 purification from one hyperflask. Chromatogram shows tight a elution peak with a corresponding drop in the pH, as the elution buffer is applied to the column. Inset: chromatogram of the whole purification with the major UV plateau corresponding to the sample application stage.

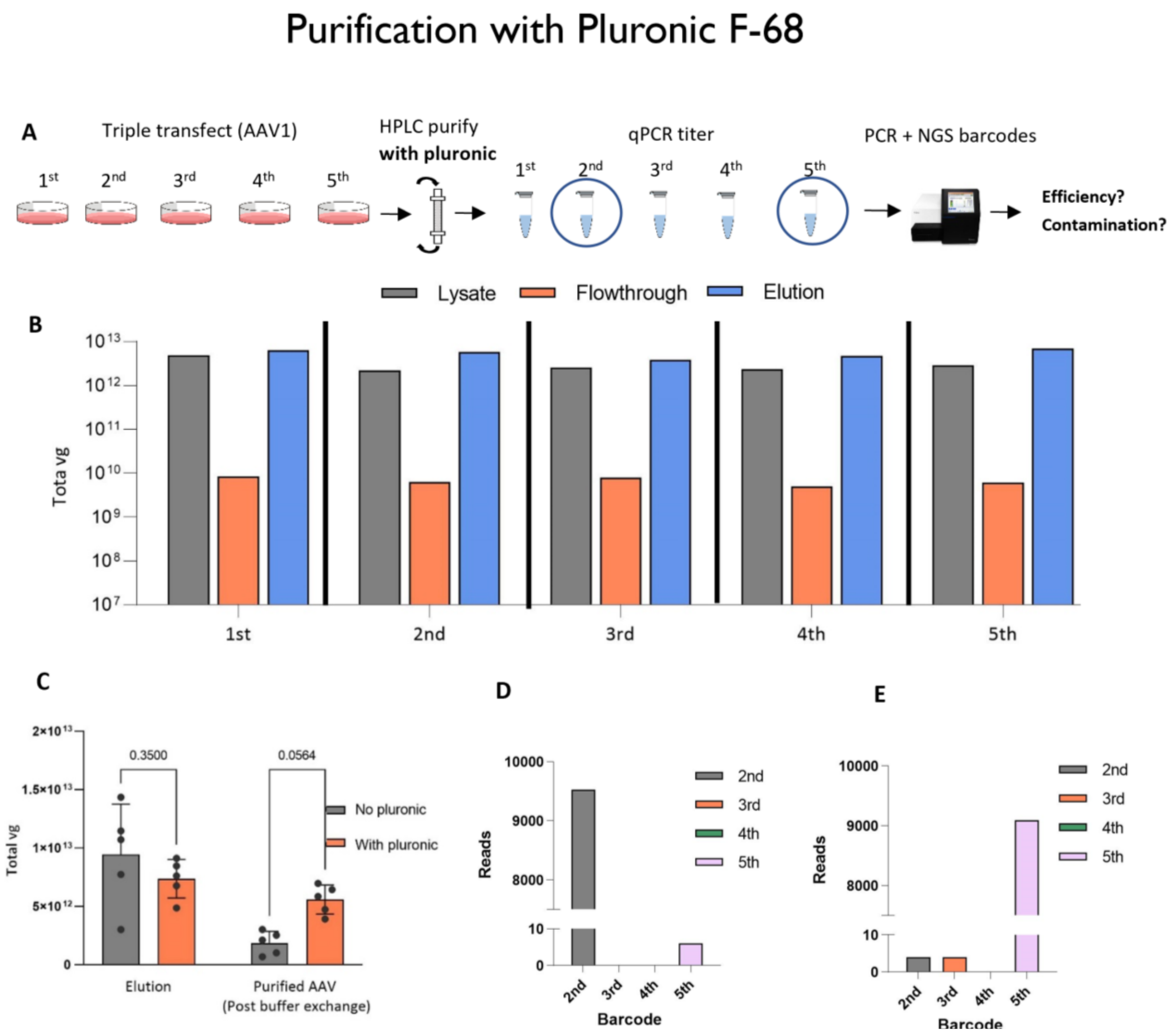

Figure S2. AAV purification at small scale over multiple cycles with Pluronic F-68 added to 0.1% vol/vol to all buffers. (A) Schematic of the experiment. **(B)** qPCR quantification of AAV vector genomes in different fractions, along preps 1-6. **(C)** Comparison of total AAV vector genomes after elution and filtration+buffer exchange with or without Pluronic F-68. Addition of Pluronic F-68 does not increase yields at the elution step, but shows a trend towards increased yields at the filtration+buffer exchange step. **(D-E)** NGS quantification of unique barcode count from the elution fractions of the 2nd prep (D) and 5th prep (E). Majority of barcodes come from the target prep, indicating low carryover contamination. P-values indicated above bars, determined via two-way ANOVA with Šídák's multiple comparisons test.

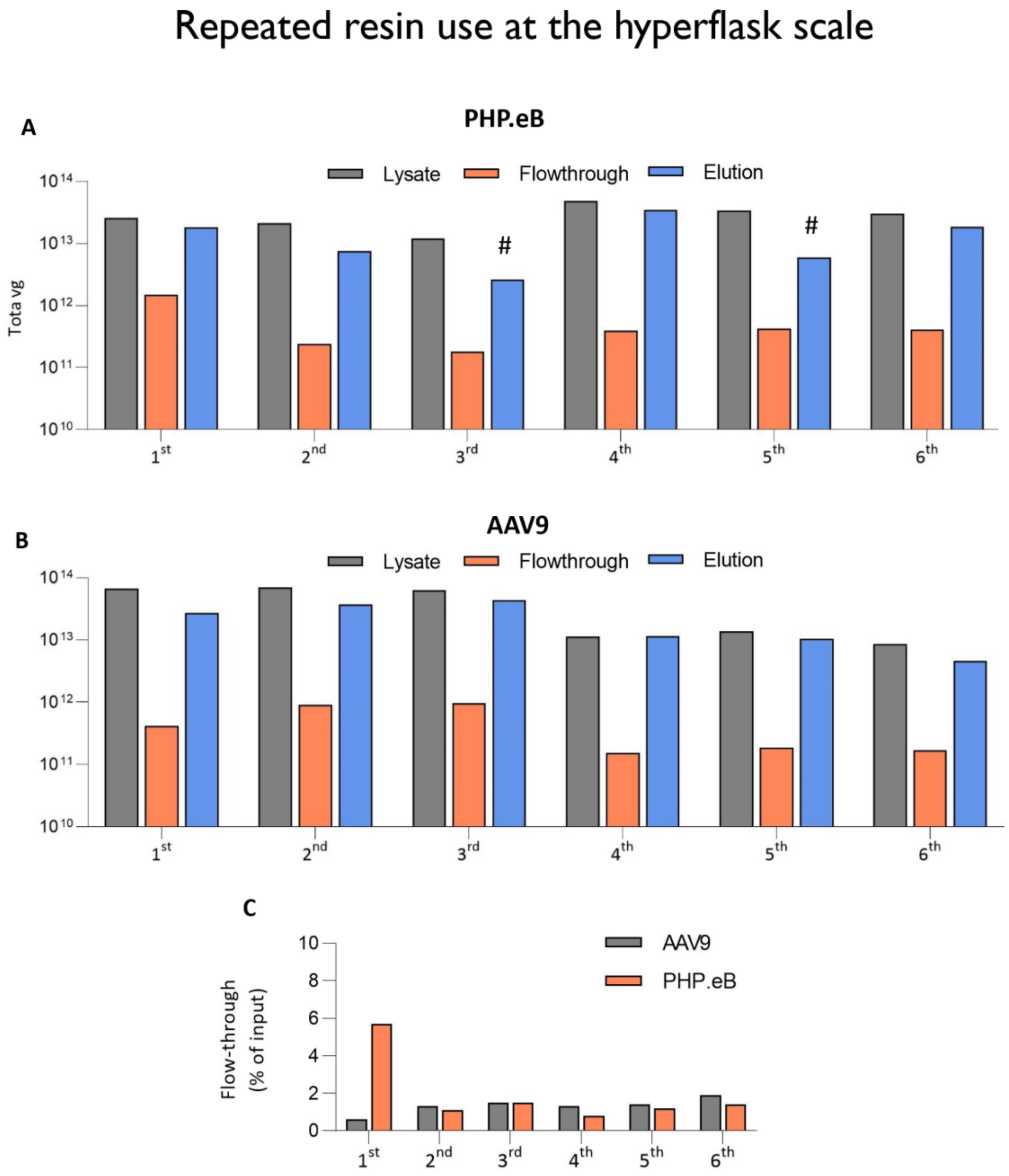

Figure S3. Stringent resin cleaning enables repeated resin re-use at large scale. Input from 1 hyperflask at each step was purified without changing the resin and AAV in input lysate, flow-through and elution tittered using qPCR. The process was repeated for PHP.eB **(A)** and AAV9 using new batches of resin for each **(B)**. AAV applied at room temperature, at 2 min residence time, 3 mL resin, eluted using pH 2.5 Glycine and resin regenerated using 1ml/min flow of 0.1M pH1 Phosphoric acid followed by 1ml/min flow of 6M Guanidine HCl for 15 minutes each. **(C)** No increase in % of AAV in flow-through was seen throughout 6 cycles. # some eluate lost due to operator error. # - some sample was lost due to handling error

AAV loss at each purification step

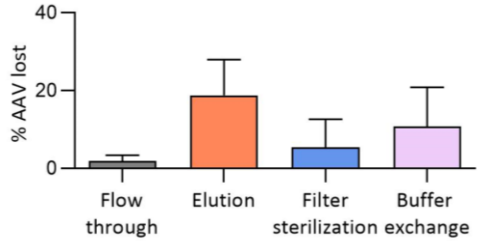

Figure S4. Percent AAV lost at each step of high-efficiency protocol. Largest losses occur at the elution (~20% of input) and buffer exchange (~10% of input) steps. Data from Fig. 4 with AAV9 and PHP.eB combined, with N=6 for each.

Full protein gel images

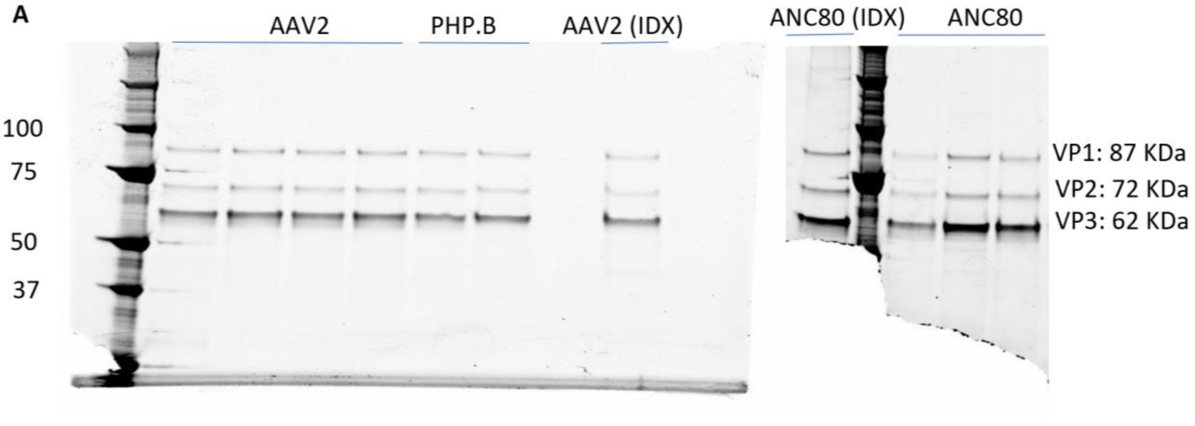

Figure S5. Uncropped gels of silver stain analysis of AAV capsids from Figure 4A.

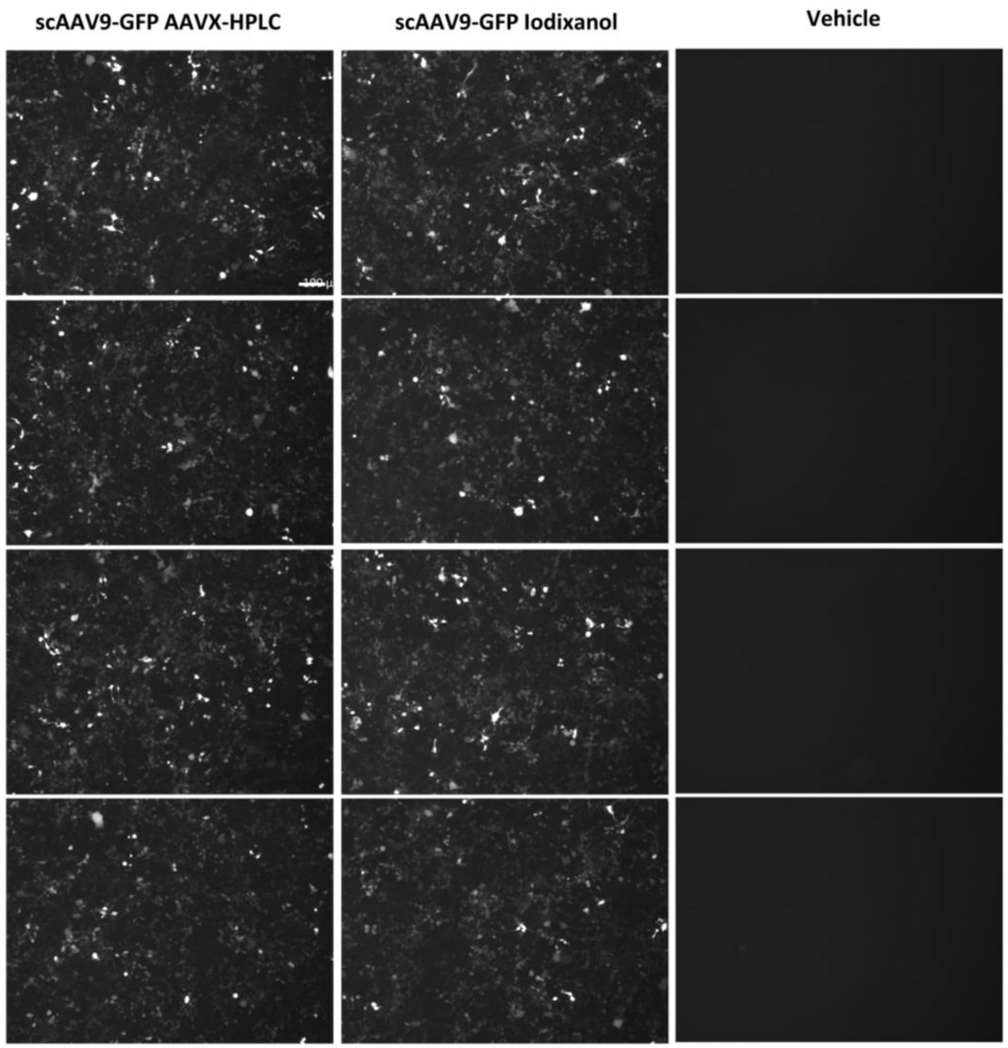

Figure S6. Full GFP images from Figure 4 B-C.

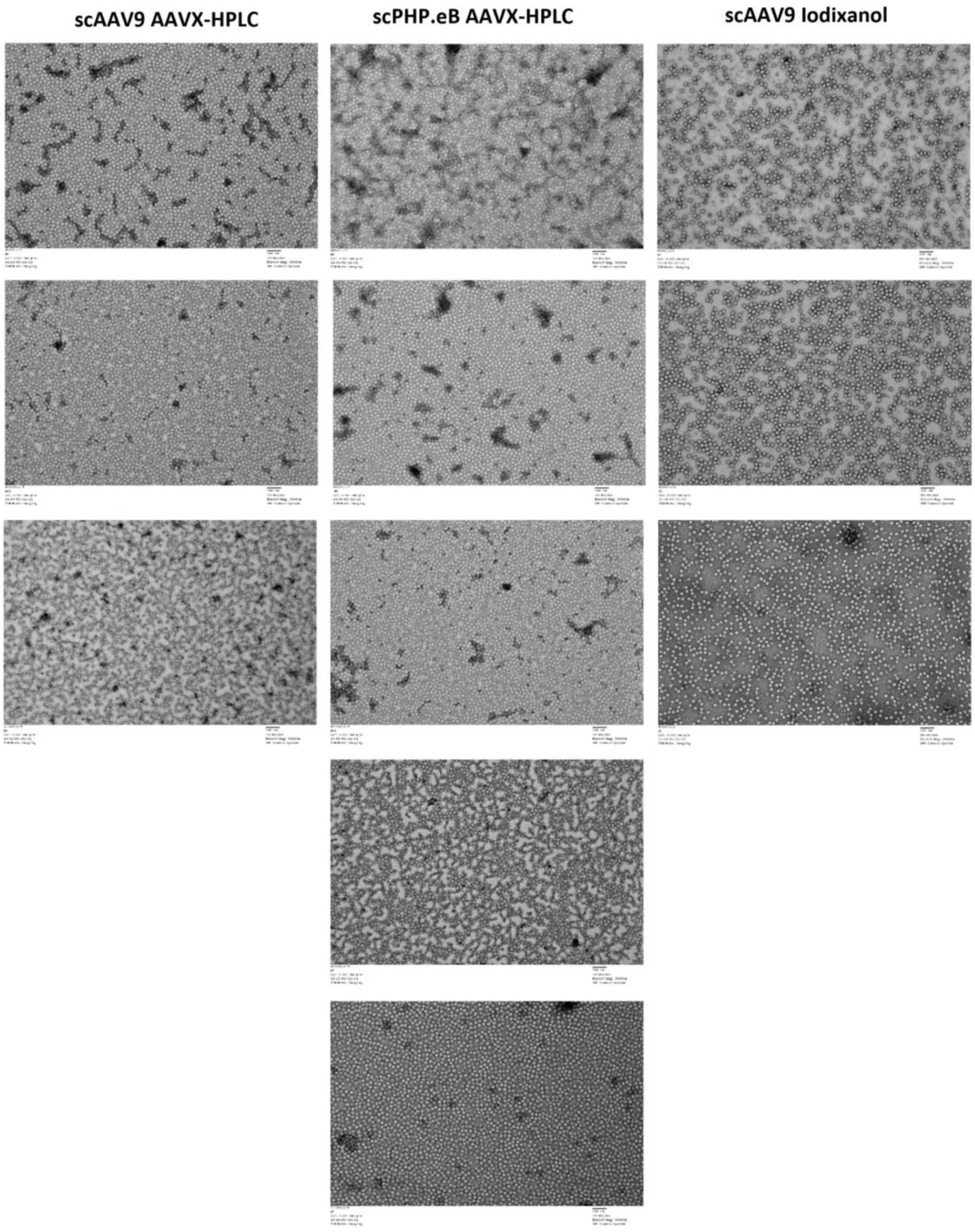

Figure S7. Full negative stain SEM images of scPHP.eB and scAAV9 preps described in Fig. 4D-E. Each image represents a separate prep. In quantification, a minimum of two images were taken and quantified for each prep.

Full liver fluorescence images

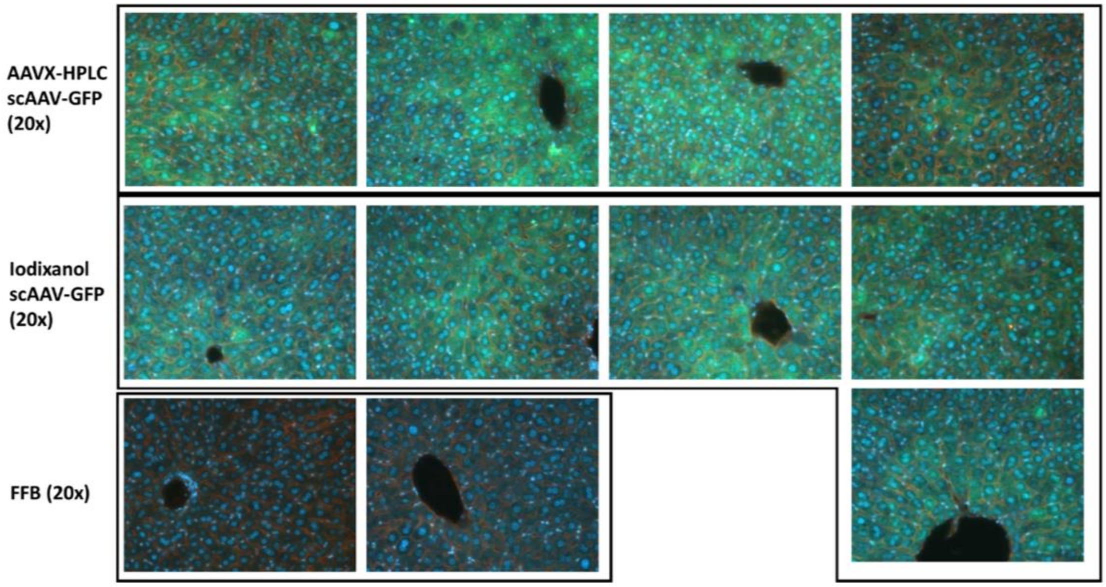

Figure S8. Images used for GFP fluorescence intensity analysis shown on Figure 5B. Every image corresponds to a different animal within the groups denoted on the left.

Individual cell GFP fluorescence

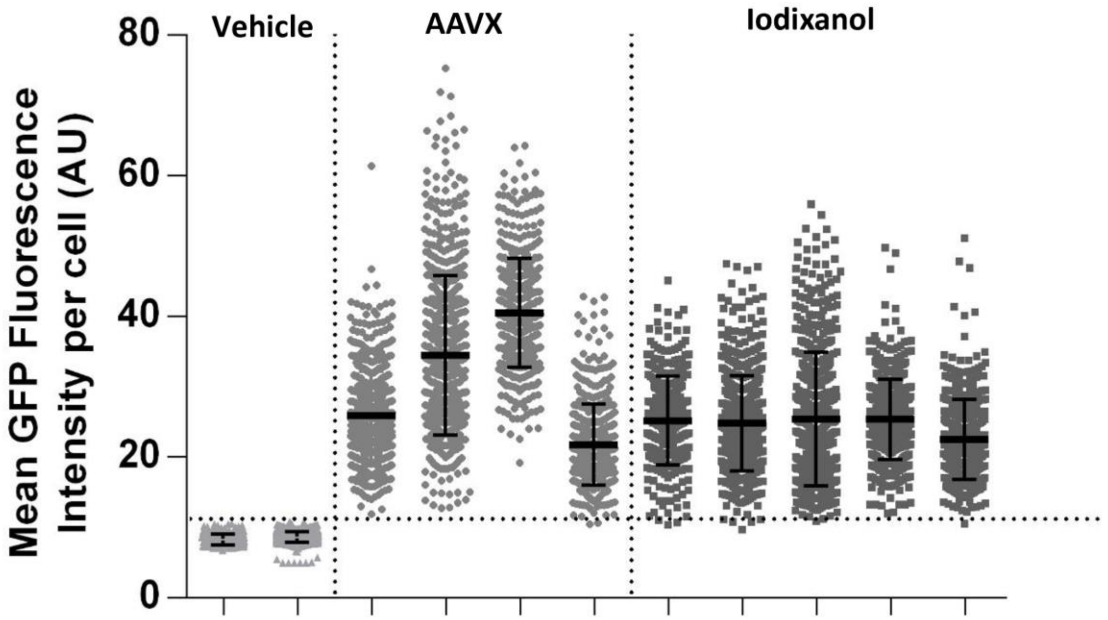

Figure S9. Individual cell GFP mean fluorescence intensities of animals injected with AAVX-HPLC or iodixanol ultracentrifugation purified AAV. Every column represents one animal and 3 images were used per animal, resulting in a total of 400-700 cells analysed per animal. Horizontal dotted line represents the mean fluorescence intensity above which cells were counted as GFP positive.

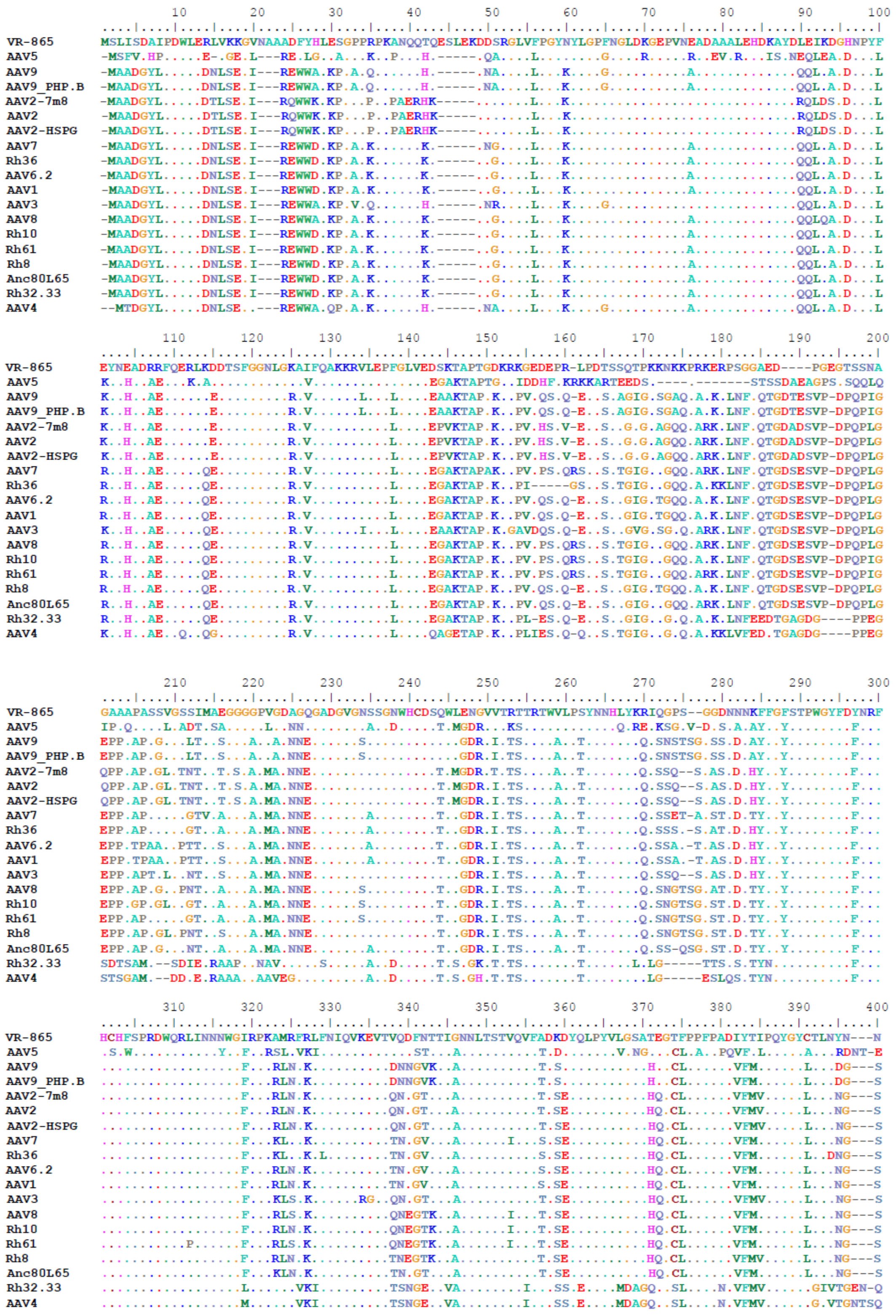

Figure S10. Multiple Sequence Alignment of AAVs used to construct the phylogenetic tree depicted in Fig. 1B.

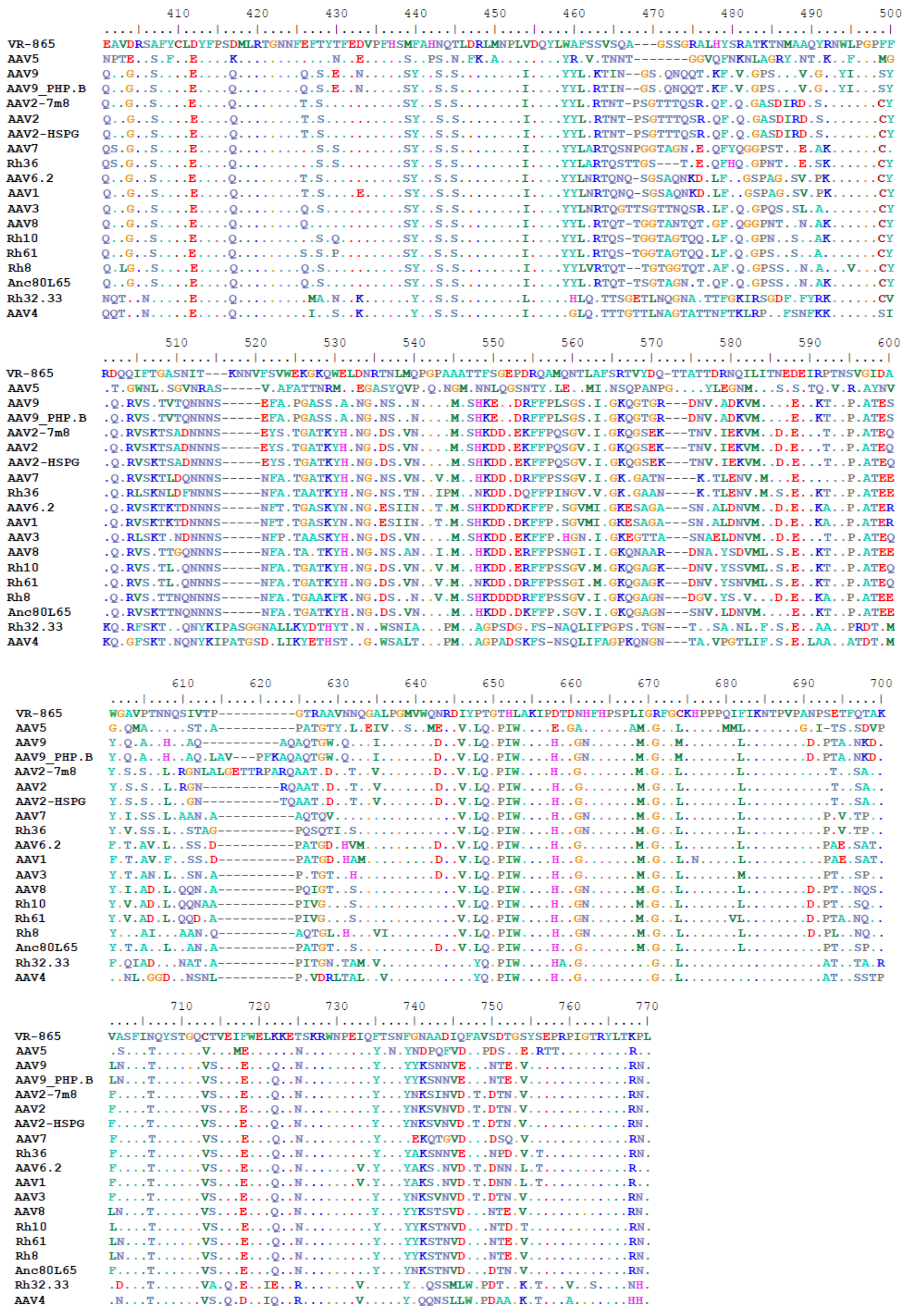

Figure S10 continued

Seq->	VR-865	AAV5	AAV9	AAV9_PHP.B	AAV2-7m8	AAV2	AAV2-HSPG	AAV7	Rh36	AAV6.2	AAV1	AAV3	AAV8	Rh10	Rh61	Rh8	Anc80L65	Rh32.33	AAV4
VR-865	ID	0.518	0.562	0.56	0.551	0.557	0.558	0.567	0.558	0.554	0.553	0.561	0.554	0.561	0.558	0.559	0.574	0.532	0.521
AAV5	0.518	ID	0.56	0.557	0.561	0.566	0.567	0.571	0.572	0.569	0.569	0.571	0.569	0.563	0.562	0.564	0.589	0.52	0.512
AAV9	0.562	0.56	ID	0.989	0.801	0.814	0.815	0.815	0.8	0.817	0.817	0.829	0.85	0.851	0.856	0.867	0.855	0.625	0.613
AAV9_PHP.B	0.56	0.557	0.989	ID	0.803	0.807	0.809	0.812	0.796	0.814	0.814	0.826	0.846	0.846	0.853	0.864	0.852	0.622	0.61
AAV2-7m8	0.551	0.561	0.801	0.803	ID	0.983	0.981	0.812	0.792	0.82	0.817	0.859	0.816	0.828	0.819	0.827	0.863	0.617	0.591
AAV2	0.557	0.566	0.814	0.807	0.983	ID	0.997	0.821	0.802	0.83	0.828	0.869	0.825	0.837	0.828	0.837	0.872	0.621	0.596
AAV2-HSPG	0.558	0.567	0.815	0.809	0.981	0.997	ID	0.821	0.804	0.832	0.829	0.871	0.825	0.837	0.828	0.837	0.874	0.621	0.597
AAV7	0.567	0.571	0.815	0.812	0.812	0.821	0.821	ID	0.906	0.852	0.849	0.842	0.879	0.886	0.887	0.871	0.907	0.66	0.627
Rh36	0.558	0.572	0.8	0.796	0.792	0.802	0.804	0.906	ID	0.844	0.841	0.833	0.86	0.866	0.861	0.837	0.878	0.653	0.627
AAV6.2	0.554	0.569	0.817	0.814	0.82	0.83	0.832	0.852	0.844	ID	0.993	0.865	0.84	0.849	0.843	0.857	0.894	0.659	0.625
AAV1	0.553	0.569	0.817	0.814	0.817	0.828	0.829	0.849	0.841	0.993	ID	0.862	0.837	0.847	0.84	0.854	0.891	0.659	0.625
AAV3	0.561	0.571	0.829	0.826	0.859	0.869	0.871	0.842	0.833	0.865	0.862	ID	0.851	0.851	0.849	0.848	0.899	0.633	0.616
AAV8	0.554	0.569	0.85	0.846	0.816	0.825	0.825	0.879	0.86	0.84	0.837	0.851	ID	0.934	0.936	0.906	0.907	0.646	0.623
Rh10	0.561	0.563	0.851	0.846	0.828	0.837	0.837	0.886	0.866	0.849	0.847	0.851	0.934	ID	0.97	0.907	0.913	0.654	0.627
Rh61	0.558	0.562	0.856	0.853	0.819	0.828	0.828	0.887	0.861	0.843	0.84	0.849	0.936	0.97	ID	0.906	0.909	0.65	0.621
Rh8	0.559	0.564	0.867	0.864	0.827	0.837	0.837	0.871	0.837	0.857	0.854	0.848	0.906	0.907	0.906	ID	0.913	0.656	0.623
Anc80L65	0.574	0.589	0.855	0.852	0.863	0.872	0.874	0.907	0.878	0.894	0.891	0.899	0.907	0.913	0.909	0.913	ID	0.672	0.641
Rh32.33	0.532	0.52	0.625	0.622	0.617	0.621	0.621	0.66	0.653	0.659	0.659	0.633	0.646	0.654	0.65	0.656	0.672	ID	0.814
AAV4	0.521	0.512	0.613	0.61	0.591	0.596	0.597	0.627	0.627	0.625	0.625	0.616	0.623	0.627	0.621	0.623	0.641	0.814	ID

Figure S11. Sequence ID of AAVs depicted in Fig. 1B. Rows and Columns are different capsids and the cells represent the % identity (amino acid of course) between the two proteins.

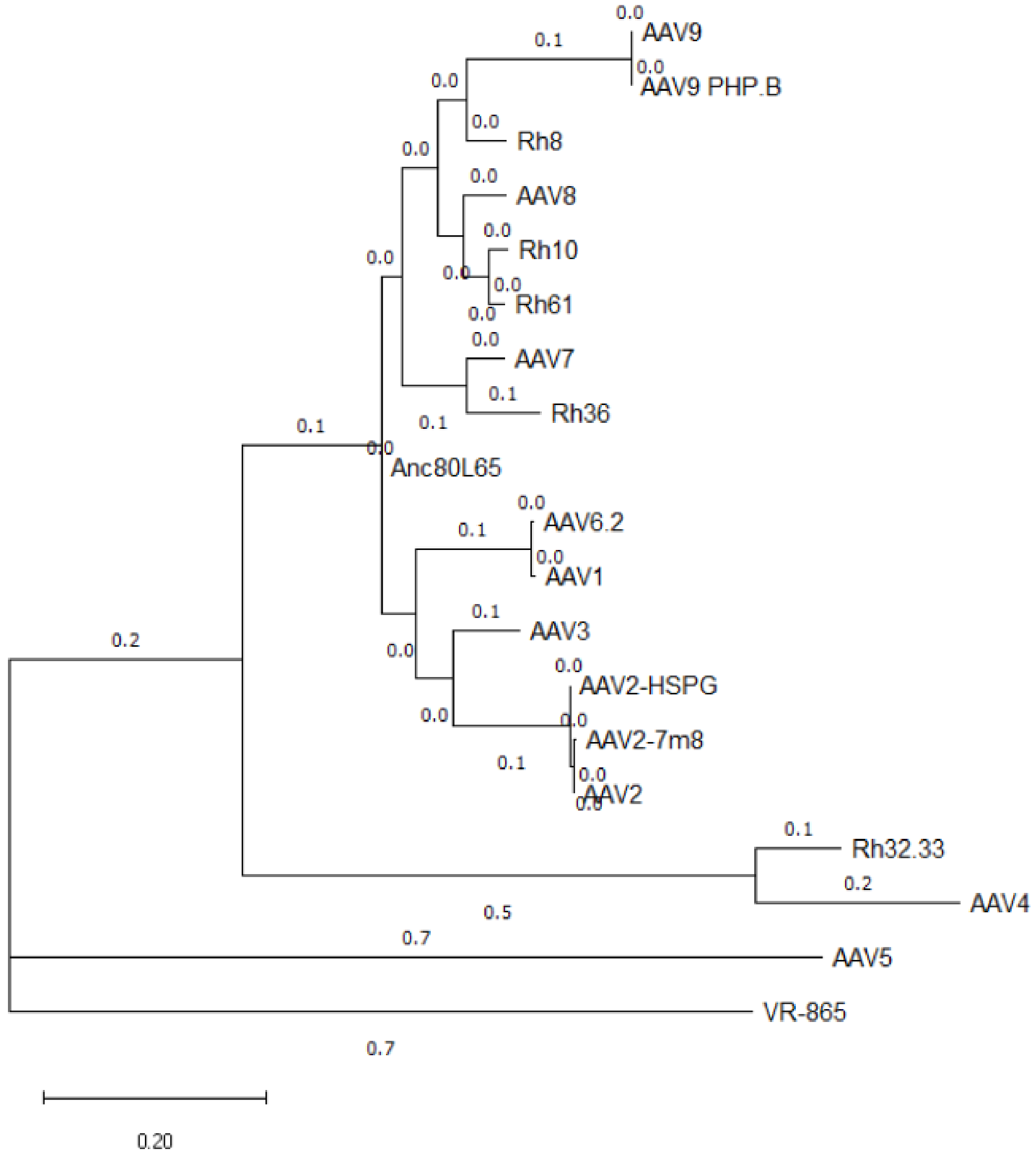

Figure S12. Newick formatted phylogeny of the phylogenetic tree depicted on Fig. 1B.

Supplemental Protocol: Production of AAV using hyperflasks and AAVX affinity chromatography

- For 1-4 hyperflask, the protocol takes approximately 10-14 days.
- We recommend ordering hyperflasks 2-3 months in advance, as they are commonly heavily backordered.
- All DNA used for transfection needs to be endotoxin free; we recommend Qiagen Endo Free kits or outsourcing to endo free companies (PureSyn); we DO NOT recommend using Zymo Endo Free kits due to their high variability in performance.
- Transgene plasmid needs to contain intact ITRs: we recommend testing ITR integrity with SmaI/XmaI digests and/or next generation sequencing for each DNA prep.
- For buffer exchange, we recommend use of Amicon Stirred Cell concentrators, particularly for purification of PHP.B and related variants, which tend to strongly sediment. Buffer exchange using Amicon Stirred Cell results in more consistently high recovery and low sedimentation. However Amicon Stirred Cell is more time-consuming, not disposable and requires an upfront investment to set up the system; therefore use of Amicon Ultra 15 Centrifugal filter devices can be used with low titer preps (<$1x10^{13}$), given careful handling (see *Buffer exchange and titration*).

Required items (per hyperflask):

Approximately 1 month prior to the start of AAV production, ensure you have all the required reagents (below). Order any that are missing and produce DNA with endo-toxin free kits.

Tissue culture and transfection:

- Cap DNA: 130 µg
- deltaF6 or other packaging plasmid: 260 µg (UPenn Vector Core)
- Transgene DNA: 130 µg
- 1L sterile filtered DMEM high glucose with 10% FBS, 1% Penstrep (11965118 Thermo Fisher, 15-140-122 Fisher Scientific)
- 600mL of filtered DMEM high glucose with 1% Penstrep **without FBS**
- Low passage HEK293T cells
- 5x 15cm tissue culture dishes
- 1x hyperflask (any variant is fine) (CLS10031-4EA Millipore Sigma or others)
- 715 µg PEIMax (1mg/ml, pH 2.6, sterile filtered) (24765-1 Polysciences Inc.)

Lysis and clarification:

- 2x 1L bottle-top PES 0.45µm filters (1143-RLS, Foxx Life Sciences)
- 3ml Triton-X 100 (T8787-100ML, Sigma)
- 2.5 mg RNAse A (1mg/ml concentration)
- 56 µl of Turbonuclease (at 250U/µl starting concentration, to a final of 25U/mL in the media) (ACGC80008, Vita Scientific)
- 56 µl of 10% Pluronic F68 (final concentration 0.001%)
- A centrifuge that can spin 2x 500ml tubes at 4000g or more (or spread the media across more tubes)
- 2x 500 ml centrifuge tubes

HPLC:

- POROS CaptureSelect AAVX resin (prepacked or free): (A36739 Thermo Fisher, A36652 Thermo Fisher)
- AKTA Pure 25 HPLC system with sample pump or equivalent. Housed at room temperature.

HPLC buffers:

- A1: 2L 1x TBS (28358 Thermo Fisher)
- A2: 1L 20%EtOH-1x TBS
- A3: 1L 2X TBS
- A4: 500mL 6M Guanidine (SRE0066-100ML, Sigma Aldrich or make from G3272-2KG Sigma Aldrich in distilled water)
- A5: 1L 20% EtOH
- A6: 500mL Phosphoric acid 0.1M, pH1 (PX0996-6 Sigma Aldrich)
- B1: 500mL 0.2M Glycine, pH 2 to pH 2.5, 0.01% vol/vol Pluronic F68 elution buffer
- 1M Tris pH8, 0.1% vol/vol Pluronic F68 neutralization buffer
- 20 x15mL tubes
- 1L 0.1M NaOH
- 1L H2O
- 1L EtOH 20%
- 0.1M and 1M NaCl for packing and column qualification
- Final Formulation Buffer (1xPBS, 35mM NaCl and 0.001% Pluronic F68) – filter sterilize with 0.2 µm filter
- **NB! Filter all HPLC buffers using a 0.45 µm or 0.2 µm bottle top filters.** This is required to avoid introduction of sedimented salts or other particulate matter into the HPLC, which can create clogs in the flow path.
- **NB! 6M Guanidine and 0.1M Phosphoric acid are hazardous: use proper PPE and precautions in handling and disposal.**

Buffer exchange:

- 0.2 µM PES syringe filters (CLS431229-50EA, Sigma Aldrich) with 20 – 50mL syringes
- **For preps <1x10^{13}vg of AAV**: 50 or 100 kDA, Amicon Ultra 15 Centrifugal Filter devices (UFC905008 or UFC910008, EMD Millipore)
- **For preps >1x10^{13}vg of AAV**:
- Ultrafiltration Discs, 100 kDa (NMW PLHK04310 Millipore Sigma)
- 500mL of 5% hydrogen peroxide in PBS – filter sterilized. Make this fresh every time, as hydrogen peroxide activity/stability decreases in neutralized pH.
- 500mL of 70% ethanol - filter sterilized
- 500mL of Final Formulation Buffer - filter sterilized.
- Amicon Stirred Cell (UFSC05001OR) with a system for providing sterile nitrogen gas pressure, such as:
  - NI UHP80 (NITROGEN UHP GR 5.0 SIZE 80 CGA) from Airgas
  - High purity pressure regulator Y11N245D580-AG (REGULATOR FIRST STAGE HIGH PURITY 3500/100 BRP DIAMETER VALVE 1/4"C CGA580CV)
  - In line sterilizing filter Y40-LF811P (FILTER 1/2T 0.003 MICRON 750 PSIG PTFE 10R STAINLESS STEEL)

- Sterile magnetic stirrer for use under a tissue culture hood, such as Mini Stirrer (VWR 10153-304) or equivalent
- Tube fittings to connect Amicon Stirred Cell to the nitrogen source:
  - We strongly recommend contacting representatives of Airgas or Swagelok (or equivalent) to verify exact details of the tube fittings and the procedure of safely connecting and operating the nitrogen tank
    - Stainless Steel Tubing Insert, 1/4 in. OD x 0.17 in. ID (SS-405-170 Swagelok)
    - 316 Stainless Steel Front Ferrule for 1/4 in. (SS-403-1 Swagelok)
    - 316 Stainless Steel Back Ferrule for 1/4 in. (SS-404-1 – Swagelok)
    - Connect the Amicon Stirred Cell inlet tube to nitrogen outlet valve by:
      - Inserting stainless steel tubing insert into the tube.
      - Place the nut-shaped tube fitting onto the tube.
      - Place front ferrule, then back ferrule onto the tube.
      - Insert tube into the nitrogen outlet valve (which should be Swagelok pressure fitting).
      - Using a wrench, screw the nut-shaped tube fitting onto the outlet valve. The front ferrule should displace back ferrule in a manner that compresses the tube securely in place. Test that the tube is tightly secured.
      - If using the in line sterilizing filter, cut the tube to create two parts, or add an additional 1/4 in. tube, then connect to the filter as described above
      - Catalogue for further reference: https://www.swagelok.com/downloads/webcatalogs/EN/MS-01-140.PDF

Protocol

AAV production

Cell seeding

- Thaw a vial of low passage HEK293T cells, expand to four 15cm dishes, at 70-80% confluency in DMEM 10% FBS 1% PenStrep;
  - Filter sterilize all media
  - Warm all media to 37 °C before use
  - Do not let cells get more than 90% confluent at any stage during expansion, and seed cells dropwise evenly across the plates + mix gently 10x in a star pattern to ensure that cells are always evenly distributed.
  - Check that they are not confluent anywhere under microscope the next day and discard any plates that highly confluent on one side and empty on another.
  - Ideally, passage cells every 48 hours, to avoid acidification of media. 72h is still okay.
- Pool cells from four 15cm dishes into 560mL of DMEM 10% FBS 1% PenStrep, mix gently and thoroughly by pipetting to create a homogenous solution with minimal bubbling, pour into hyperflask by placing the hyperflask upright, tilted to the side as per manufacturer's instructions (https://www.youtube.com/watch?v=uo3jEQGz8Z0).
- Grow cells until they reach ~80% confluency. This commonly takes 48 hours. Check that the cells are 70-80% confluent and evenly distributed under a microscope before transfection. Transfecting 100%

confluent cells results in ~3-10x reduced AAV production levels. Transfecting 40-50% confluent cells results in a ~30-50% decrease in titers.

Transfection

- Mix 10mL DMEM (sterile filtered, room temp) with DNA (vector:cap:deltaF6 at 130μg:130μg:260μg).
- Mix 10mL DMEM (sterile filtered, room temp) with 715μg of PEIMax (1mg/ml, pH 2.6, sterile filtered).
- Mix the two solutions together, shake and vortex immediately for 15 seconds (pulse vortexing, not continuously), incubate at RT for 15 minutes.
- Add the solution to 560 ml of **DMEM-1%Penstrep** (sterile filtered, warmed to 37 C), mix thoroughly and gently with a serological pipette or swirling. **NB! Do not include serum here! Inclusion of serum in the transfection mix reduces AAV yields by 3-10x.**
- Remove the hyperflask from the incubator, pour out media carefully so as not to disturb the cells. (https://www.youtube.com/watch?v=1B_3Luum-ME)
- Gently pour the transfection mix in DMEM-1% PenStrep into the hyperflask, top up with DMEM-1% PenStrep and remove bubbles as needed.
- Put the hyperflask back into the incubator, incubate for four days. 3-5 days is also fine. Interestingly, the optimal amount of time for incubation varies between different reports, so currently there is no published consensus I am aware of. Anecdotally, 3-5 days results in similar yields.

Harvesting and lysis

- After 3-5 days of incubation, pour media from the hyperflask into a 1L bottle.
- Add lysis reagents to the media in the bottle:
  - 3ml Triton-X 100
  - 250 µl of RNAse A (at 10mg/ml concentration, a total of 2.5 mg RNAse A)
  - 56 µl of 25U/mL of Turbonuclease
  - 56 µl of 10% Pluronic F68 (final concentration 0.001%)
- Mix with a serological pipette until the solution is clear; avoid introducing air or swirling, as it generates foam. Pour media back into the hyperflask slowly (otherwise generates foam). Store any volume that is left over in a 50mL tube.
  - The above is necessary, because adding lysis reagents directly into the hyperflask does not allow them to be rapidly uniformly distributed across cells.
- Incubate the hyperflask at 150rpm shaking for 30 min – 1 hour at 37 °C along with left-over volume in the 50mL tube. Shaking incubation aids with mechanical lysis of cells.
  - If you do not have access to a sterile shaking incubator, we recommend hand shaking for ~5 min and subsequent incubation at 37 °C or carefully double bagging the hyperflask and incubating in a non-sterile incubator, then disinfecting the outer bags with bleach and ethanol prior to proceeding.
- Decant lysate in the hyperflask into a 1L bottle, wash the hyperflask with 140 mL of PBS and add it to rest of the lysate – forming 700 mL total.
- If not proceeding to the HPLC purification on the same day, store at 4 °C (1-2 days) or -20 °C.
- Clarify the lysate (below). Clarification is critical to prevent clogging of the HPLC during the run. We recommend performing this on the day of loading onto the HPLC, to minimize re-formation of aggregates during storage.

- Divide the lysate evenly between two 500mL centrifuge tubes, centrifuge at 4000g or higher at for 30 minutes.
- Decant supernatant into 1L 0.45uM CA/PES filter, filter everything.
- Take aliquots of the clarified lysate for qPCR/ddPCR titration, store aliquots at -20°C…-80°C. Titer these aliquots along with your final purified AAV later. This provides very helpful information in troubleshooting, allowing you to determine your purification efficiency, acting as a sanity check (i.e the amount of purified AAV cannot be greater than the amount of AAV in input lysate, accounting for the accuracy of your qPCR/ddPCR titration) and in the case of low yields allows you to pinpoint whether the failure was at transfection or purification.

HPLC purification and buffer exchange

A full introduction into the usage and theory of HPLC is out of the scope of this protocol. The below is intended for users with basic training and capacity to operate HPLC machines and is based on the Akta Pure 25 system using pre-packed 1mL AAVX columns. Regardless of the machine used, the below parameters are critical for efficient purification:

- **Purification is carried out at room temperature.** Purification at cold temperatures substantially decreases binding efficiencies of the resin (Fig. 2D). For this purpose, both the clarified lysate containing AAV and the HPLC machine need to be brought to room temperature prior to purification. If the HPLC machine is housed in a fridge, we recommend not running the machine inside the fridge with cooling turned off and a closed door, since this can cause considerable increase of the temperature inside the fridge. Instead, we recommend either 1) placing the machine outside the fridge, 2) running the machine with fridge turned off and door left open or 3) hooking the fridge up to an external temperature controller (such as BN-LINK Digital Cooling Thermostat Controller, Amazon) and setting the set-point to 22°C to 24°C.
- **AAVX resin binding capacity is not exceeded.** Thermo Fisher indicates a binding capacity of up to $1x10^{14}$ vg/ml which varies between serotypes. A safe rule of thumb is to use 1mL of resin per 1-2 hyperflasks, depending on the yield. When pooling AAV from multiple hyperflasks, we recommend packing your own columns with AAVX resin at a higher volume.
- **Lysate application speed does not exceed 1mL/min for a 1mL resin – i.e. the residence time is no less than 1 min.** Resin dynamic binding capacity decreases with increasing loading speed and may result in more AAV in the flow-through. **We recommend a 2min residence time, or 0.5mL/min loading speed for a 1mL resin for maximum recovery.**
- **Elution is performed in up-flow.** Elution in down-flow (or the same flow direction as lysate application) results in approximately 20% less AAV in the elution. This is most likely because a majority of AAV binds close to the inlet on the resin; therefore eluting in the opposite direction avoids AAV re-binding of the resin at the elution step.
- **Elution pH is less than 2.9, with pH 2-2.5 optimal.** AAV is acid stable at below pH 3 and will not elute above pH 2.9. Some serotypes can have strong binding affinities to the resin (such as AAV9 to the POROS AAV9 resin) and decreasing pH down to pH 2 can help increase recovery for such serotypes (Thermo Fisher – personal communication).
- **Resin regeneration is carried out with at least 15 min of 0.1M pH1 Phosphoric acid, followed by at least 15 min of 6M Guanidine.** While not a concern with small-scale preps, at large scale preps ($3x10^{13}$ … $2x10^{14}$) we commonly observed decreased binding efficiencies with resin re-use when 10 min of 6M

Guanidine only was used, particularly for PHP.eB. The AAVX resin is highly acid stable, and increasing regeneration to 15 min of 0.1M pH1 Phosphoric acid, followed 15 min of 6M Guanidine restored binding efficiencies at large scales for at least 6 runs (Fig. S3).

- **The AAVX resin is NOT stable in high pH solutions.** Accidental treatment of the resin with 0.1M NaOH will destroy the resin.

HPLC purification protocol:

- Bring the HPLC machine and the sample to room temperature
- Prepare **filtered** HPLC buffers as described in the Required Items section. **NB! 6M Guanidine and 0.1M Phosphoric acid are hazardous: use proper PPE and precautions in handling and disposal.**
- Connect the AAVX column to the machine using wet connection.
  - (Manually flow some liquid out of the inlet, connect the column inlet connector in the wet environment to avoid introducing air into the column; repeat for column outlet).
- Place buffer lines A1 to A6 and B1 into the corresponding buffers. Place Sample line (S1) and Sample Buffer line into 1x TBS.
  - Tape the lines to the buffer bottles if they do not come with weights.
  - Cover the bottles with parafilm to avoid evaporation and contamination
- Prime inlets and purge pumps (see Akta Pure user manual section 5.4, pages 160-171)
  - Prime the Sample inlet (S1) with TBS to avoid loss of your sample
- Optional but recommended: place a 1L bottle in the HPLC Outlet line to collect flow-through.
  - This is useful if for whatever reason AAV fails to bind to the resin (such as fouling, low temp, or operator error), as it allows re-purification of the prep.
- Pipette 0.11 mL of 1M Tris pH8 + 0.1% vol/vol Pluronic F68 neutralization buffer into 25 15mL tubes; place them into the fraction collector, and set the fraction collector position to 1.
- Place sample line (S1) into the AAV clarified lysate
  - Place the bottle on the machine tilted and place the S1 line at the bottom most area, to be able to collect 100% of the lysate.
- Ensure that there is sufficient volume of buffer for all buffers, and that all inlet lines are fully submerged.
- Adjust the volume, speed or other parameters of the AAVX_HPLC_S1 Akta run protocol as necessary.
  - Import the AAVX_HPLC_S1 protocol file on Akta Pure. Created with Unicorn v7.1.
  - For other HPLC systems, see full overview of the run protocol below (*HPLC run method*)
- **Print out and go through the starting checklist every time before starting a new run (below).**
- **Open the and start the AAVX_HPLC_S1 protocol.**
  - Depending on the volume of lysate and run speed, the run may take anywhere between 1 hour and 36 hours. We recommend doing the calculation before-hand to pick up elution fractions soon after the run is done.
  - We recommend staying with the machine for the first 10-15 minutes in every new run to ensure that no leaks are present, that the sample inlet contains no air bubbles (which can cause pre-mature termination of loading, see *Troubleshooting*), and that sample application reaches a steady plateau.
  - See *Troubleshooting* for examples of successful and unsuccessful runs

- After the run is complete, remove the elution fractions containing AAV, store them at 4°C for short term or -20°C for long term.
- Multiple preps can be automatically purified back-to-back by copying the protocol, changing sample input to S2..S7, saving the new protocols, and starting them during the run of the first protocol. This adds them to the run cue, and the machine will automatically continue to these protocols after the previous ones are complete.
    - Note that purification of multiple preps back-to-back removes the ability to collect flow-through with the Outlet tube, as the machine unfortunately contains only a single outlet line.
    - In this case, place the Outlet tube into waste bin to avoid overflowing the collection bottle.
- After the last run, Remove the AAVX column, cap the tubes and store it at 4°C
- When the machine will not be expected to be used for longer than a week, perform System CIP (cleaning in place):
    - Place all inlets used in the run into 1L 0.1M NaOH
    - Start the System CIP protocol or manually run 20 mL liquid through all lines
    - Repeat for H_20 and 20% ethanol
    - Store all lines in 20% ethanol

Buffer exchange

- Before pipetting AAV containing solutions, we recommend coating all pipette tips and serologicals with Final Formulation Buffer (1xPBS, 35mM NaCl and 0.001% Pluronic F68) or another Pluronic F68 containing buffer to minimize AAV binding to plastic. We also recommend usage of low-retention tips if available.
- Thaw elution fractions if frozen previously.
- From here on, work in sterile conditions.

Protocol for buffer exchange using Amicon Stirred Cell

- While fractions are thawing, place 50mL tubes on a rack in a TC hood and remove caps.
- Attach 0.2 µm PES filter disks on top of the tubes by wrapping parafilm around the edge of the filter tightly.
- Remove plungers from 50mL syringes, and insert the syringes into the filter disks.
- Pipette 2mL of Final Formulation Buffer (1xPBS, 35mM NaCl and 0.001% Pluronic F68) right onto the filter inlet, ensuring the buffer wets the filter.
- Incubate for 15 min or more.
- Assemble Amicon Stirred Cell manifold with the 100 kDa ultrafiltration disk at the bottom, glossy side facing upwards, inside the TC hood.
    - See User guide here https://www.merckmillipore.com/FI/en/product/Amicon-Stirred-Cell-50mL,MM_NF-UFSC05001 and here: https://www.merckmillipore.com/FI/en/product/Ultrafiltration-Discs-100kDa-NMW,MM_NF-PLHK04310?bd=1#anchor_BRO
    - Before first use, we recommend sterilizing the manifold and the filter disk. The filter disk can be sterilized in 70% ethanol for 5 minutes. For manifold sterilization, see Decontaminate and clean at the end of this section.
- Place the Amicon on a magnetic stirrer and connect to the nitrogen tank.

    - Tape the tube down if it does not stay in place, to keep the manifold on the magnetic stirrer.
- Remove the top of the manifold and pour 50mL of Final Formulation Buffer into the Amicon manifold.
- Incubate for 15 min or more.
- Open the nitrogen flow to pressurize the system.
  - Slowly open the nitrogen tank first, then slowly open the course stage regulator on the right, finally slowly open the fine stage regulator to allow nitrogen flow into the manifold. Always follow manufacturer safety instructions.
  - Be mindful to not exceed Amicon and filtration membrane maximum pressure limits – 75 psi and 70 psi respectively.
  - Turn on magnetic stirrer at low speed to check the functioning of the entire system.
  - Turn off nitrogen flow from fine stage regulator when approximately 2-3 mL of liquid is left.
  - **NB! Turning off nitrogen flow does not immediately eliminate pressure from the manifold! To immediately eliminate pressure, slowly open the blue valve on the manifold cap.**

- Once elution fractions are thawed, centrifuge tubes containing elution fractions briefly to spin down the liquid.
- Sterilize tubes with 70% ethanol and bring them to the tissue culture hood.
- Pool all elution fractions containing AAV into a single 50mL tube.
  - Coat all pipette tips with sterile Final Formulation Buffer before aspirating AAV.
  - Before aspirating liquid from a fraction, mix it by pipetting briefly to ensure there is no concentrated layer of AAV that remains at the bottom.
- Bring the volume up to 48mL with Final Formulation Buffer and mix.
- Pour or pipette the volume into the 50mL syringe filter, insert plunger and filter through the disk.
  - Bringing the volume up to a total of 48 mL effectively dilutes the AAV, minimizing losses at the filtration step.
- Pour or pipette the filtered solution into the previously assembled Amicon manifold.
- Seal the Amicon cap, turn on the magnetic stirrer at low to medium speed, and open nitrogen flow to pressurize the system.
  - Adjust the nitrogen pressure to provide continuous but not too rapid filtration. The filtration should take 5 minutes or more to completion. Rapid filtration results in high local densities of AAV on the filter surface, which leads to AAV aggregate formation and subsequent loss of titer and/or bioactivity. The stirring action by magnetic stir bar mitigates this substantially but would be reduced by very rapid filtration.
  - Filtration speed is proportional to the concentration of AAV (and other molecules above 100kDA). Thus, more concentrated preps will require longer time to filter – up to 15-20 minutes in our hands.
- Stop the filtration once 2-5mL of liquid is left.
  - Turn off the nitrogen gas, then slowly and gently lift the blue valve to de-pressurize
  - **NB. Do not allow filtration to proceed to overconcentration at this step – this can cause AAV sedimentation and loss.** Always aim to stop the filtration before 1mL of liquid is left.
- Remove the Amicon cap, pour in Final Formulation Buffer to 50mL, repeat filtration
- Repeat filtration until a total of >1000x dilution is achieved. This normally takes 2-3 filtration cycles.
  - When leaving 5mL of filtrate left, adding 45mL of Final Formulation buffer results in 10x dilution. In this case 1000x dilution is achieved in three cycles.

  - This can also be achieved in two steps if starting with <5mL of volume of elution fractions (approximately 10x dilution at the filtration step) and repeating the buffer exchange twice with >10x dilution.
- At the last filtration cycle, to obtain an accurate desired volume of final AAV, carefully observe the liquid level and stop and de-pressurize the system at 5mL.
- Stop the stirrer and estimate liquid volume by eye or by measuring with a serological pipette.
- Remove the waste tube (keeping the tube connector attached to the manifold) and place a 5mL tube underneath the waste outlet.
- Continue filtration to the desired amount with stir bar turned on low, by estimating remaining volume in the Amicon manifold through observing the volume of waste in the 5mL tube.
  - This is required because the Amicon manifold is flat and accurate volume estimations at less than 2mL are difficult. By estimating starting volume and volume in waste, an accurate estimation of volume left in the Amicon manifold can be made.
  - Removal of the waste tube is necessary because it contributes to dead volume (approximately 2mL), which can be filled with air to various degree, making accurate waste volume estimation difficult.
- Alternatively, achieve desired final AAV volume by stopping the filtration at various points and measuring left-over volume with a pipette or serological.
- The solution can accurately be concentrated to a few hundred microliters this way. However, we recommend keeping AAV at the highest possible volume allowed by downstream experimental requirements, particularly for PHP.B and its derivatives, as high concentrations of AAV will aggregate more readily during freeze-thaw cycles (Wright JF et al. Mol Ther. 2005 Jul;12(1):171-8.).
- Depressurize Amicon, remove stir bar and aspirate AAV into a new 1.5mL or 2mL tube.
  - Optionally wash the filter membrane with additional 100 µL of Final Formulation Buffer and add to AAV.
- Measure the volume of final AAV solution with a P1000 pipette or serological.
  - For P1000, turn the pipette down to low volume, aspirate, then turn the pipette to higher volumes while the tip is submerged in the solution. When the solution is fully aspirated this way, the volume can be read from the pipette.
- Record the volume or bring up to a desired volume with Final Formulation Buffer.
- Take and store a 15 µl aliquot for titration
  - Take a higher volume if further tests, such as protein electrophoresis are performed.
  - This is to avoid unnecessarily thawing AAV designated for experimental applications.
  - Do not open AAV tubes meant for experimental applications under non-sterile conditions.
- Aliquot AAV into separate smaller aliquots unless the full amount is expected to be used in a single experiment.
- Store AAV and the titration aliquot (if not immediately used for titration) at -80 °C.
- Decontaminate and clean the Amicon manifold between concentration of different AAV preps, and at the end:
  - Disconnect the Amicon manifold from the nitrogen source
  - Disassemble the manifold, and place all parts into a 2L beaker filled with 500mL of 5% hydrogen peroxide
  - Incubate with slight shaking for 5 minutes
  - Repeat with 70% ethanol and Final Formulation Buffer
  - Dry before storage or concentration of a new prep

    - Alternatively, based on manufacturer's datasheet, Amicon Stirred Cells are compatible with standard sterilizing gas mixtures or can be autoclaved for at least 10 cycles at 121 °C, 1 bar (250 °F, 15 psi) for 30 minutes.

Protocol for buffer exchange using Amicon Ultra 15 Centrifuge devices

- **NB. Amicon Ultra 15 Centrifuge devices and their equivalents from competitors do NOT come sterilized.** We recommend sterilization with sterilizing gases. If these cannot be used, 70% ethanol or UV can be attempted, but these may adversely affect the membrane (depending on the membrane type) and are likely of low efficacy.
- While fractions are thawing, place the Amicon Ultra 15 tubes on a rack in a TC hood and remove caps.
- Pipette 2mL of Final Formulation buffer onto the membrane of the Amicon tubes.
- Attach 0.2 µm PES filter disks on top of the tubes by wrapping parafilm around the edge of the filter tightly.
- Remove plungers from 20mL syringes and insert the syringes into the filter disks.
- Pipette 2mL of Final Formulation Buffer right onto the filter inlet, ensuring the buffer wets the filter.
- Incubate for 15 min or more.
- Once elution fractions are thawed, centrifuge tubes containing elution fractions briefly to spin down the liquid.
- Sterilize tubes with 70% ethanol and bring them to the tissue culture hood.
- Pool all elution fractions containing AAV into a single 15-50mL tube.
    - Coat all pipette tips with sterile Final Formulation Buffer before aspirating AAV.
    - Before aspirating liquid from a fraction, mix it by pipetting briefly to ensure there is no concentrated layer of AAV that remains at the bottom.
- Bring the volume up to 10mL with Final Formulation Buffer and mix.
- Pour or pipette the volume into the 20mL syringe filter, insert plunger and filter through the disk.
    - Bringing the volume up to max volume dilutes the AAV, minimizing losses at the filtration step.
- Cap the Amicon tubes and centrifuge to concentrate:
    - Continuous centrifugation will result in high local density of AAV at the filter membrane, causing aggregation and sedimentation out of the solution, which decreases titers and bioactivity.
    - We recommend centrifuging for 1-2 minutes, removing the tubes from the centrifuge, opening them under the TC hood and mixing/washing the membrane with a P1000. This substantially reduces AAV aggregation although does not eliminate it for high concentration preps.
    - **NB. Aim to not concentrate below 1mL, as this increases AAV aggregation.**
    - **NB. Do not centrifuge at speeds higher than 5000g!**
- Concentrate to 1mL.
- Decontaminate and bring the Amicon into a TC hood.
- Fill the tube with Final Formation Buffer to 14 ml and mix.
- Repeat until a total of >1000x dilution and desired final volume is achieved.
    - The solution can be concentrated to a few hundred microliters. However, we recommend keeping AAV at the highest possible volume allowed by downstream experimental requirements, particularly for PHP.B and its derivatives, as high concentrations of AAV will

aggregate more readily during buffer exchange as well as freeze-thaw cycles (Wright JF et al. Mol Ther. 2005 Jul;12(1):171-8.).

- Measure the volume of final AAV solution with a P1000 pipette or serological.
    - For P1000, turn the pipette down to low volume, aspirate, then turn the pipette to higher volumes while the tip is submerged in the solution. When the solution is fully aspirated this way, the volume can be read from the pipette.
- Record the volume or bring up to a desired volume with Final Formulation Buffer.
- Take and store a 15 µl aliquot for titration.
    - Take a higher volume if further tests, such as protein electrophoresis are performed.
    - This is to avoid unnecessarily thawing AAV designated for experimental applications.
    - Do not open AAV tubes meant for experimental applications under non-sterile conditions.
- Aliquot AAV into separate smaller aliquots unless the full amount is expected to be used in a single experiment.
- Freeze at -80C.

Titration

A thorough and detailed protocol for AAV titration using qPCR or ddPCR is described in Sanmiguel, J., Gao, G., & Vandenberghe, L. H. (2019). Quantitative and Digital Droplet-Based AAV Genome Titration. Adeno-Associated Virus Vectors, 51–83. doi:10.1007/978-1-4939-9139-6_4. We recommend performing AAV titrations based these protocols.

Troubleshooting

A single hyperflask in our hands consistently yields an average of $3x10^{13}$ vg of purified AAV with well producing transgenes such as CMV-GFP for both single stranded vectors and self-complementary vectors. While some transgenes or serotypes may inherently produce at lower yields, yields below $1x10^{13}$ vg per hyperflask likely indicate a technical issue somewhere in the process.

Elution UV peak height roughly correlates with AAV yield. A peak of approximately the height (given efficient packing of the column) of the loading UV plateau for a single hyperflask generally indicates a yield of $1\text{-}3x10^{13}$ vg. Elution peaks much lower than (or with a lower area under the curve) indicate inefficient AAV production or purification. When no or very low elution peak is present, it is recommended to troubleshoot before proceeding to buffer exchange to save time and resources.

Examples:

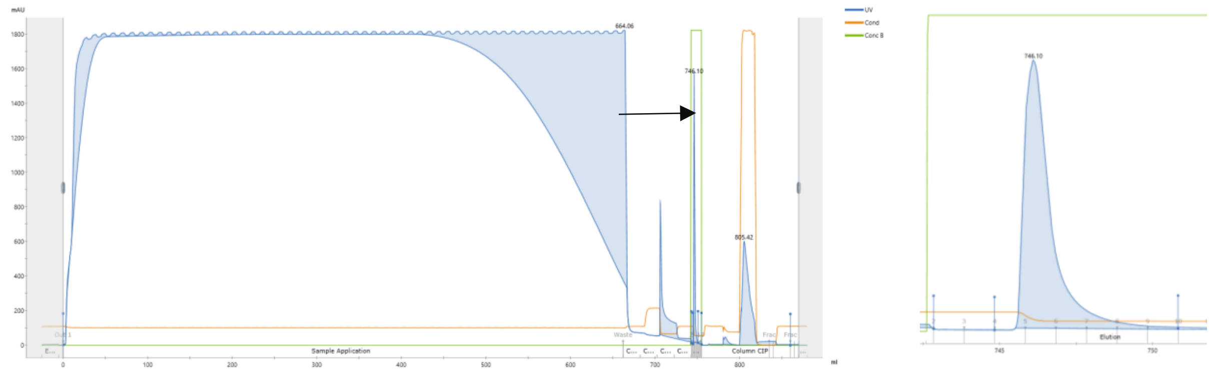

Reasonably high elution peak - efficient production and purification. Left: chromatogram of the full run; right: magnified elution UV peak.

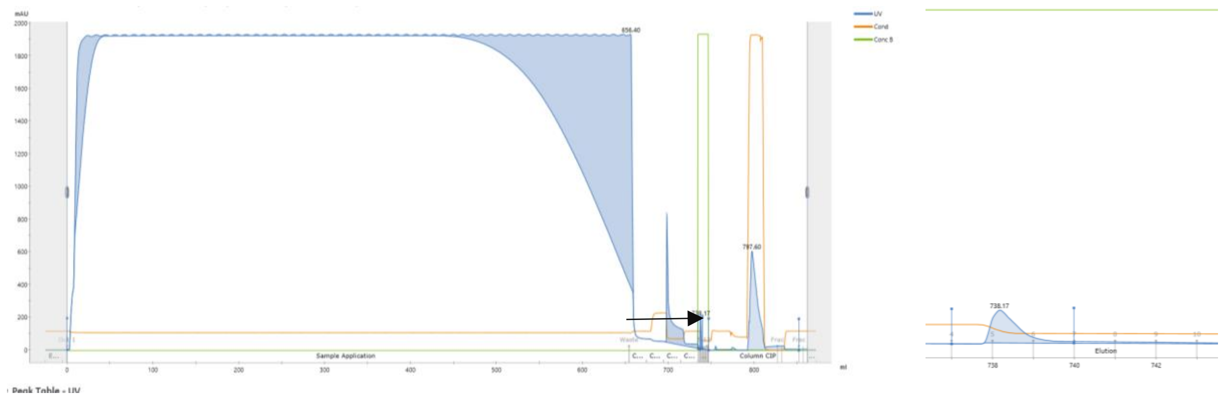

Low elution peak - inefficient production and/or purification. Left: chromatogram of the full run; right: magnified elution UV peak.

Troubleshooting

Low elution peak or low final AAV yield

- Titer aliquots taken from the pre-purification lysate, flow-through and purified AAV to identify the step at which AAV was lost.
- If pre-purification lysate contains low amounts of AAV, it is likely an AAV production issue.
- If flow-through contains high amounts of AAV, it is likely an HPLC purification issue.
- If pre-purification lysate contains high amounts of AAV and flow-through contains little AAV, it is likely a buffer exchange issue. Troubleshoot below accordingly.

Low AAV production yield

- Were HEK293T cells used?
- Were cells of low passage?
- Were cells not allowed to grow to confluency at any point during culture?
- Were cells uniformly distributed during culture?

- Was transfection performed with cells at 70-80% confluency?
- Was transfection performed with reagents at room temperature (not 4 degC)?
- Was FBS excluded from the transfection mix?
- Were all three plasmids (helper, cap and transgene) present at 260 µg:130 µg:130 µg ratios?
- Was PEI**max** used?
- Was PEImax used at the correct amount? (715 µg)
- Were all three plasmids of the correct identity?
- Did the transgene plasmid contain intact ITRs?
    - Mutated ITRs are one of the most common reasons for low yields. Mutated ITRs exist at some percent of the total population in most DNA preps. When the plasmid prep contains a high percent of mutated ITRs, yields are substantially reduced (up to 10-fold or more) and empty capsid percentage is increased. We recommend against use of such AAV preps to maintain experimental consistency and always checking for ITR integrity with SmaI/XmaI digests and/or next generation sequencing. For this reason, if it is known that a transgene will be extensively used in experimental studies, we recommend producing a Mega or Giga scale DNA prep, validating the integrity of the ITRs of this prep, aliquoting and using it for all subsequent AAV production runs.
- Was the detergent and nuclease lysis performed correctly (containing all components, and not for longer than 2 hours)?

High amounts of AAV in the flow-through and other HPLC purification issues

- Was purification carried out at room temperature?
- Was the sample brough to room temperature prior to purification?
- Was the resin/column new?
- Was the AAVX resin not allowed to dry out during storage?
- If not, was the resin/column previously regenerated with >15min pH1 Phosphoric acid and >15min 6M Guanidine?
- If so, has the resin been used more than 10 times?
    - While some data indicate that properly regenerated resins can be used for up to at least 20 times, we recommend switching to a new resin if flow-through issues emerge after roughly 10 uses.
- Did the resin get exposed to 0.1M NaOH or other strong alkaline agents?
- Is this a serotype validated to bind to AAVX or a new untested serotype?
- Clogged column:
    - Was the lysate clarified with centrifugation and filtration before loading onto the HPLC?
    - Was upflow selected during elution and column regeneration?
- Premature termination of sample loading:
    - Was the HPLC protocol set to “Inject all sample using air sensor” in the Sample Application step (on by default in the AAVX_HPLC_S1 protocol)?
    - If so, the system likely detected an air bubble in the sample line and proceeded to the following steps. We recommend purging the sample line as described in the Akta manual and repeating the purification.

  - Alternatively, Sample Application can be set to "Inject Fixed Sample Volume" – in this case sample volume must be accurately measured before to prevent underloading or loading air onto the column.
- Continuously increasing preC pressure/sample pump pressure during loading:
  - The column is being clogged. If the lysate was clarified with centrifugation and filtration before loading onto the HPLC and resin regenerated correctly, this could be a frit issue, if self-packed columns are used. We recommend replacing frits. If pre-packed column was used, it is a manufacturer issue or a column reaching the end of its lifespan. Either way, we recommend using a new column.
- Fluctuating UV line/preC line during loading:
  - There is likely an air bubble in the sample line. While purification can still work, it runs the risk of premature terminating the loading if "Inject all sample using air sensor" in the Sample Application step is selected. We recommend terminating the program, placing the Sample line into a separate tube of TBS, manually performing priming and purging, placing the line back into sample and re-starting the run.

Low final AAV yield/buffer exchange issues

- Was upflow used during HPLC elution?
  - Downflow decreases yields by approximately 20%.
- Was Pluronic F68 used in the elution buffer?
- Were elution fractions neutralized with pH8 Tris- 0.01% Pluronic F68?
- Were filters, plasticware and pipette tips coated with Final Formulation Buffer (or other Pluronic F68 containing buffer) during handling?
- Was buffer exchange carried out according to instructions, preventing overconcentration?
- AAV sedimentation (white cloudy particle formation):
  - Was buffer exchange carried out according to instructions, preventing overconcentration?
  - Some reports suggest some serotypes can be re-solubilized by gentle overnight shaking at room temperature. While most AAV serotypes are stable at room temperature for that duration, it is up to the user to decide whether this is worth trying.
- Broken Amicon Ultra 15 tubes during centrifugation:
  - Was centrifugation speed kept below 5000g?
- Low filtration speed at buffer exchange:
  - This is likely a high concentration prep, or contains additional molecules that are retained by the buffer exchange membrane. We recommend patiently continuing purification as reasonable or high yields can still be obtained. Alternatively, the sample can be diluted and split between more buffer exchange units if available.
- High filtration speed at buffer exchange:
  - The prep likely contains little to no AAV. Pure PBS filters fully in seconds. Decrease pressure or centrifugation speed.
- Liquid leakage under the cap of Amicon Ultra 15 units during centrifugation:
  - Cap overtightened or more than 14mL of media loaded onto the Amicon.

- This is a particular problem with fixed-angle rotors. We recommend using a swinging bucket rotor if available.
- Low buffer exchange efficiencies despite no clear technical flaw:
    - Take aliquots of all purification and buffer exchange steps to determine the exact step where loss occurs.
    - For high concentration preps, splitting the prep between multiple filtration/buffer exchange units can reduce loss.

Starting checklist

-Sample filtered?
-Aliquot of sample (cleared lysate) taken?
-Column attached?
    -Correct orientation?
    -Correct resin?
-All inlets in correct buffers?
    -A1: TBS
    -A2: 20%EtOH-TBS
    -A3: 2X TBS
    -A4: 6M Guanidine
    -A5: 20% EtOH
    -A6: Phosphoric acid 0.1M, pH1
    -B1: 0.2M Glycine-0.01% Pluronic F68
    -S1: Sample 1
    -S2: Sample 2...
    -Buffer: TBS
    -Outlet in outlet tube
-Enough buffer in each tube?
-Outlet in outlet?
-All inlets primed?
-All pump heads purged?
    -Purge confirmed?
-Sample pump purged?
    -Purge confirmed?
-Fractionation:
    -Fraction collector set to position 0?
    -Enough tubes added for fractions? Apprx 20 tubes per run
    -Tris-Pluronic added to collection tubes?
-Method
    -Correct method selected?
    -Correct outlets selected?
    -Correct sample application volume selected?

-Correct fraction volumes selected?
-Correct location for save file?
-Enough volume in the sample to match method?
-Waste empty?
-Everything double-checked?

HPLC run method

For users with Akta Pure systems we highly recommend importing the AAVX_HPLC_S1 and System_CIP protocols to avoid unwanted errors. The below is intended as a complete specification of run parameters for users of other HPLC systems.

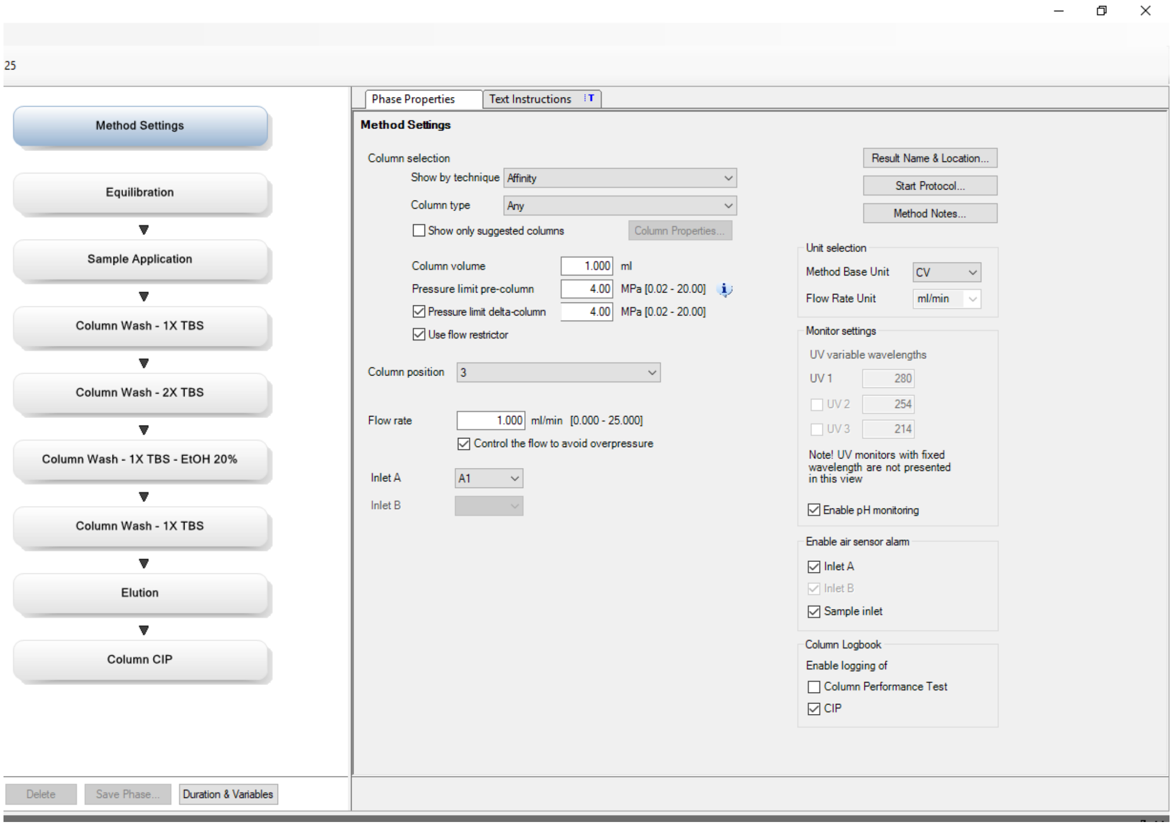

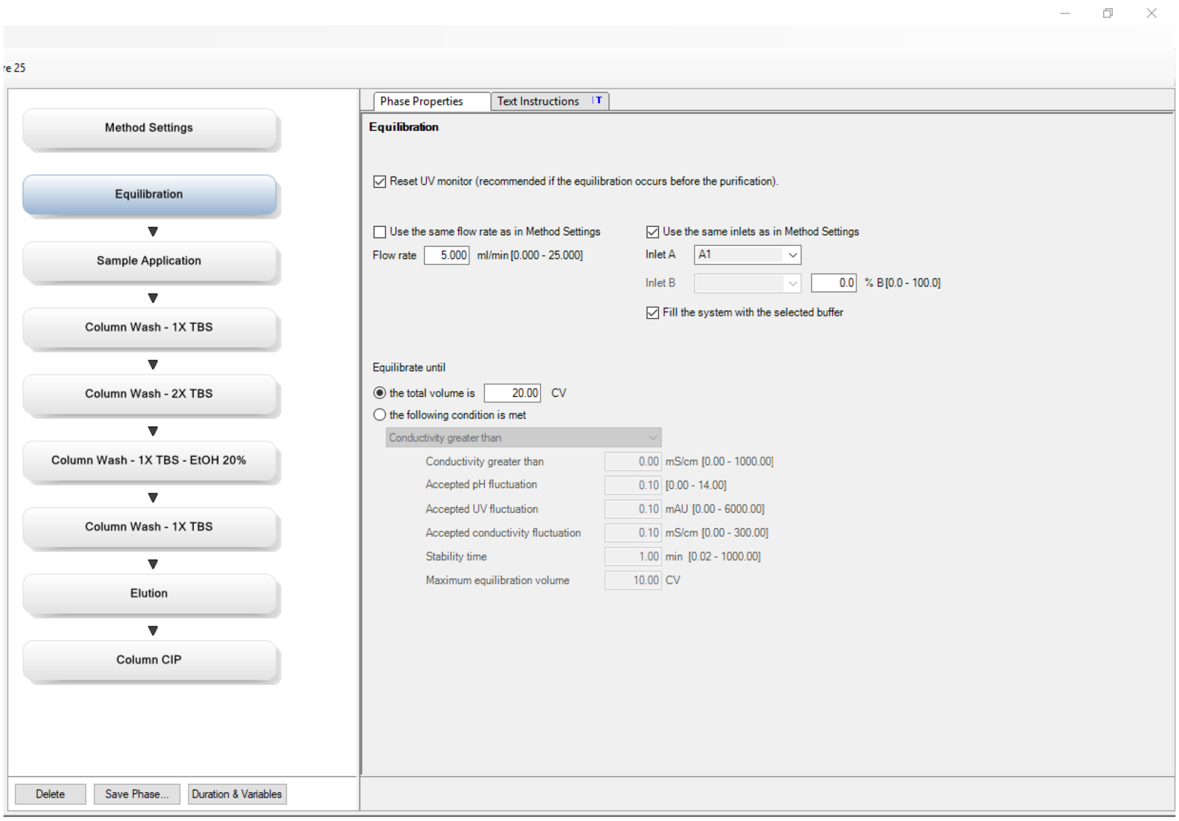
Method Settings
Equilibration
Sample Application
Column Wash - 1X TBS
Column Wash - 2X TBS
Column Wash - 1X TBS - EtOH 20%
Column Wash - 1X TBS
Elution
Column CIP
Delete
Save Phase...
Duration & Variables
Phase Properties
Text Instructions
Equilibration
Reset UV monitor (recommended if the equilibration occurs before the purification).
Use the same flow rate as in Method Settings
Flow rate 5.000 ml/min [0.000 - 25.000]
Use the same inlets as in Method Settings
Inlet A A1
Inlet B 0.0 % B [0.0 - 100.0]
Fill the system with the selected buffer
Equilibrate until
the total volume is 20.00 CV
the following condition is met
Conductivity greater than
Conductivity greater than 0.00 mS/cm [0.00 - 1000.00]
Accepted pH fluctuation 0.10 [0.00 - 14.00]
Accepted UV fluctuation 0.10 mAU [0.00 - 6000.00]
Accepted conductivity fluctuation 0.10 mS/cm [0.00 - 300.00]
Stability time 1.00 min [0.02 - 1000.00]
Maximum equilibration volume 10.00 CV

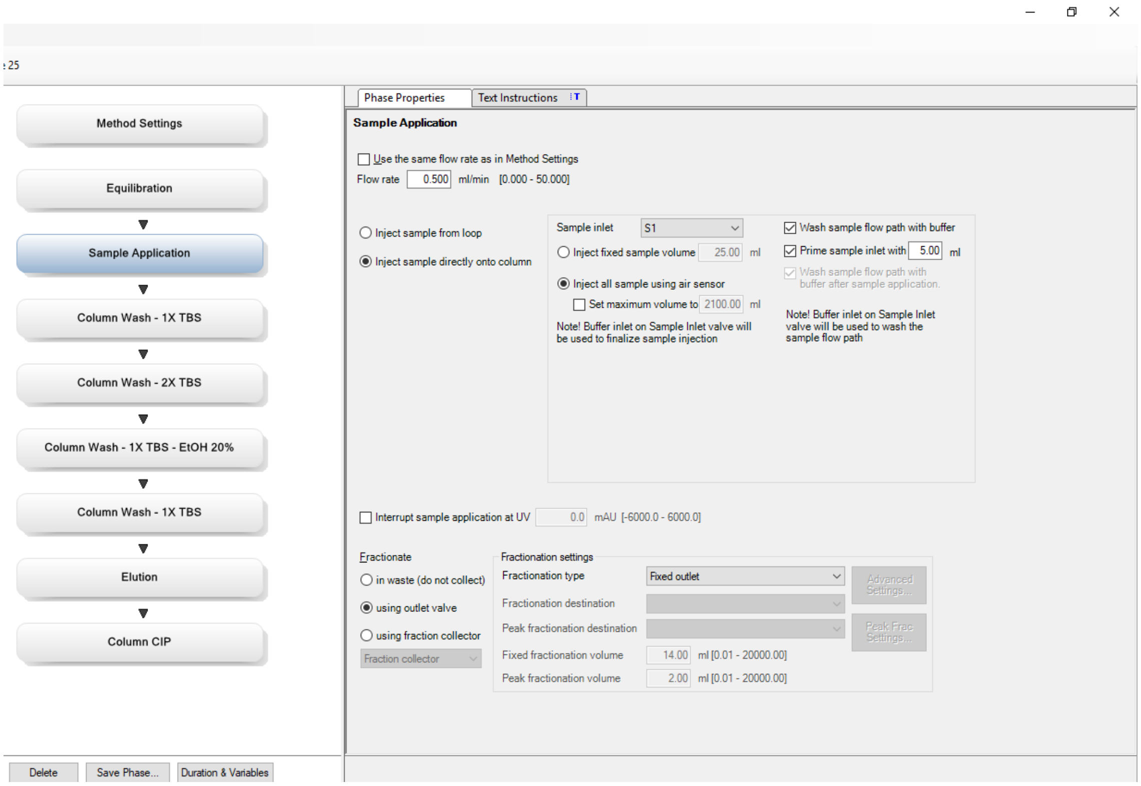
Method Settings
Equilibration
Sample Application
Column Wash - 1X TBS
Column Wash - 2X TBS
Column Wash - 1X TBS - EtOH 20%
Column Wash - 1X TBS
Elution
Column CIP
Delete
Save Phase...
Duration & Variables
Phase Properties
Text Instructions
Sample Application
Use the same flow rate as in Method Settings
Flow rate 0.500 ml/min [0.000 - 50.000]
Inject sample from loop
Inject sample directly onto column
Sample inlet S1
Inject fixed sample volume 25.00 ml
Inject all sample using air sensor
Set maximum volume to 2100.00 ml
Note! Buffer inlet on Sample Inlet valve will be used to finalize sample injection
Wash sample flow path with buffer
Prime sample inlet with 5.00 ml
Wash sample flow path with buffer after sample application.
Note! Buffer inlet on Sample Inlet valve will be used to wash the sample flow path
Interrupt sample application at UV 0.0 mAU [-6000.0 - 6000.0]
Fractionate
in waste (do not collect)
using outlet valve
using fraction collector
Fraction collector
Fractionation settings
Fractionation type Fixed outlet
Fractionation destination
Peak fractionation destination
Fixed fractionation volume 14.00 ml [0.01 - 20000.00]
Peak fractionation volume 2.00 ml [0.01 - 20000.00]
Advanced Settings...
Peak Frac Settings...

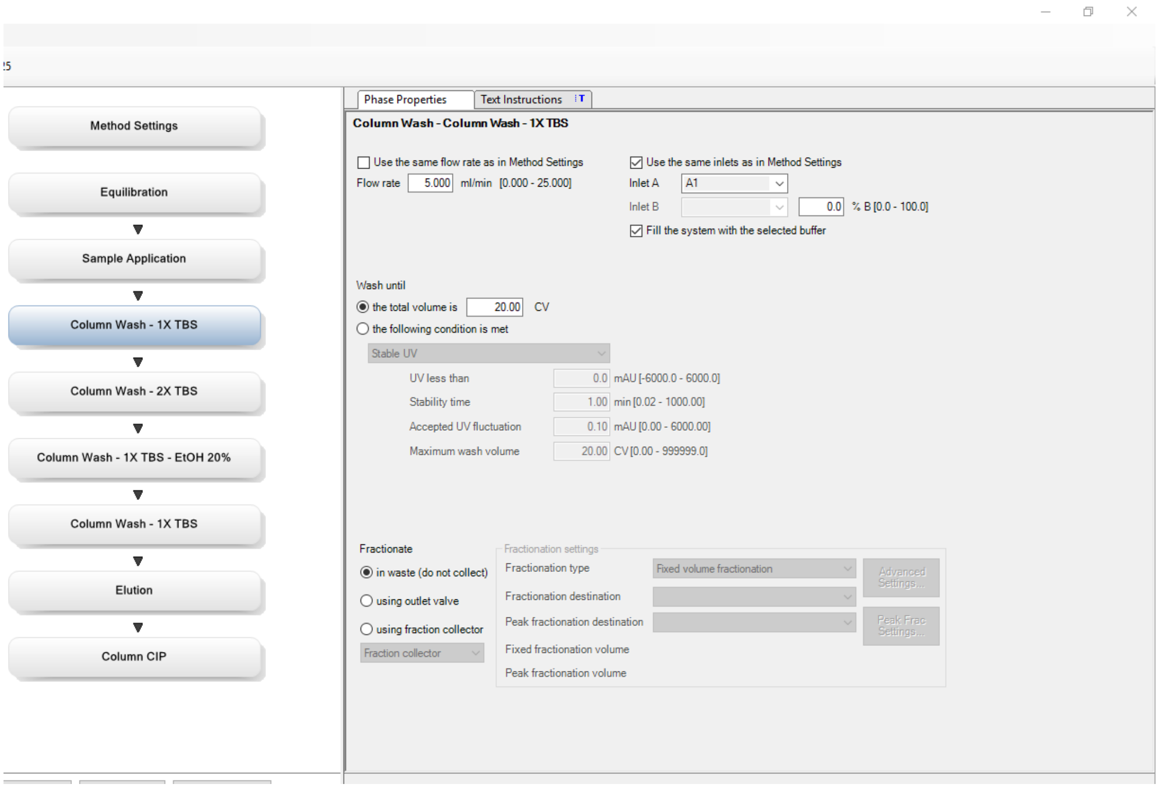
Method Settings
Equilibration
Sample Application
Column Wash - 1X TBS
Column Wash - 2X TBS
Column Wash - 1X TBS - EtOH 20%
Column Wash - 1X TBS
Elution
Column CIP
Phase Properties
Text Instructions
Column Wash - Column Wash - 1X TBS
Use the same flow rate as in Method Settings
Flow rate 5.000 ml/min [0.000 - 25.000]
Use the same inlets as in Method Settings
Inlet A A1
Inlet B
0.0 % B [0.0 - 100.0]
Fill the system with the selected buffer
Wash until
the total volume is 20.00 CV
the following condition is met
Stable UV
UV less than 0.0 mAU [-6000.0 - 6000.0]
Stability time 1.00 min [0.02 - 1000.00]
Accepted UV fluctuation 0.10 mAU [0.00 - 6000.00]
Maximum wash volume 20.00 CV [0.00 - 999999.0]
Fractionate
in waste (do not collect)
using outlet valve
using fraction collector
Fraction collector
Fractionation settings
Fractionation type
Fixed volume fractionation
Fractionation destination
Peak fractionation destination
Fixed fractionation volume
Peak fractionation volume
Advanced Settings...
Peak Frac Settings...

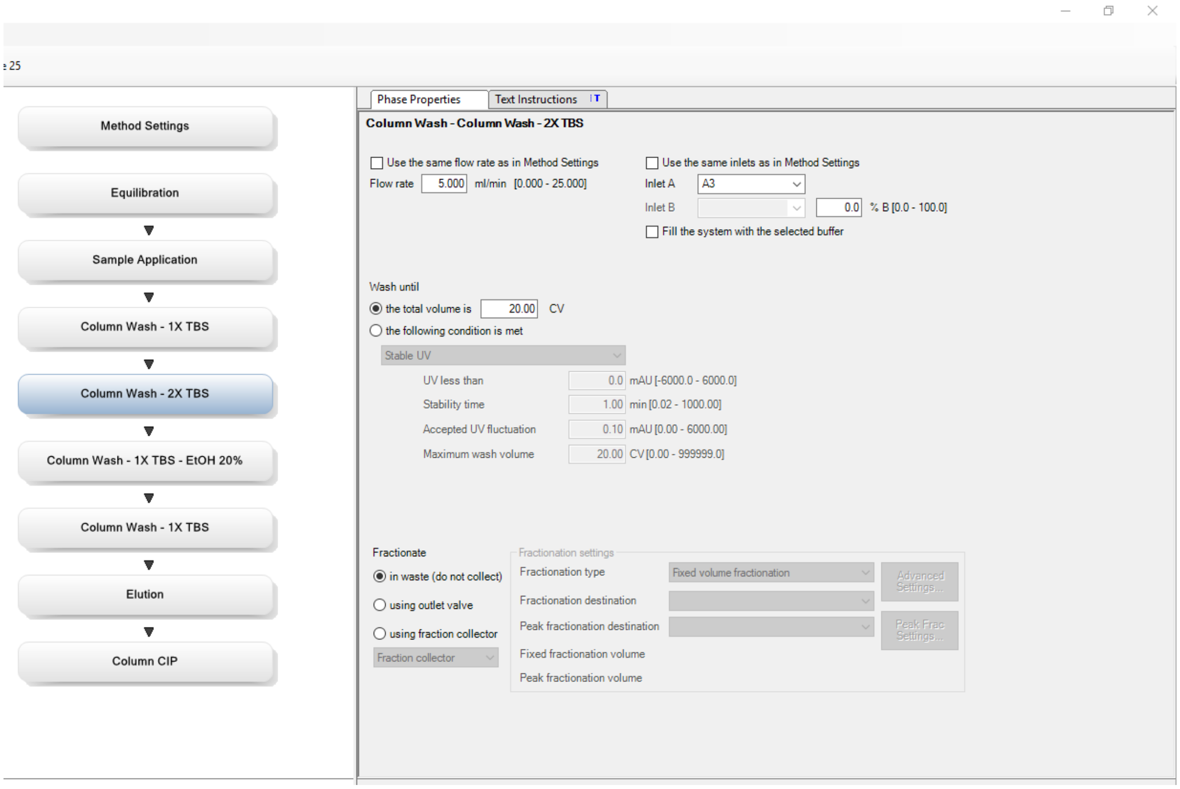
Method Settings
Equilibration
Sample Application
Column Wash - 1X TBS
Column Wash - 2X TBS
Column Wash - 1X TBS - EtOH 20%
Column Wash - 1X TBS
Elution
Column CIP
Phase Properties
Text Instructions
Column Wash - Column Wash - 2X TBS
Use the same flow rate as in Method Settings
Flow rate 5.000 ml/min [0.000 - 25.000]
Use the same inlets as in Method Settings
Inlet A A3
Inlet B
0.0 % B [0.0 - 100.0]
Fill the system with the selected buffer
Wash until
the total volume is 20.00 CV
the following condition is met
Stable UV
UV less than 0.0 mAU [-6000.0 - 6000.0]
Stability time 1.00 min [0.02 - 1000.00]
Accepted UV fluctuation 0.10 mAU [0.00 - 6000.00]
Maximum wash volume 20.00 CV [0.00 - 999999.0]
Fractionate
in waste (do not collect)
using outlet valve
using fraction collector
Fraction collector
Fractionation settings
Fractionation type
Fixed volume fractionation
Fractionation destination
Peak fractionation destination
Fixed fractionation volume
Peak fractionation volume
Advanced Settings...
Peak Frac Settings...

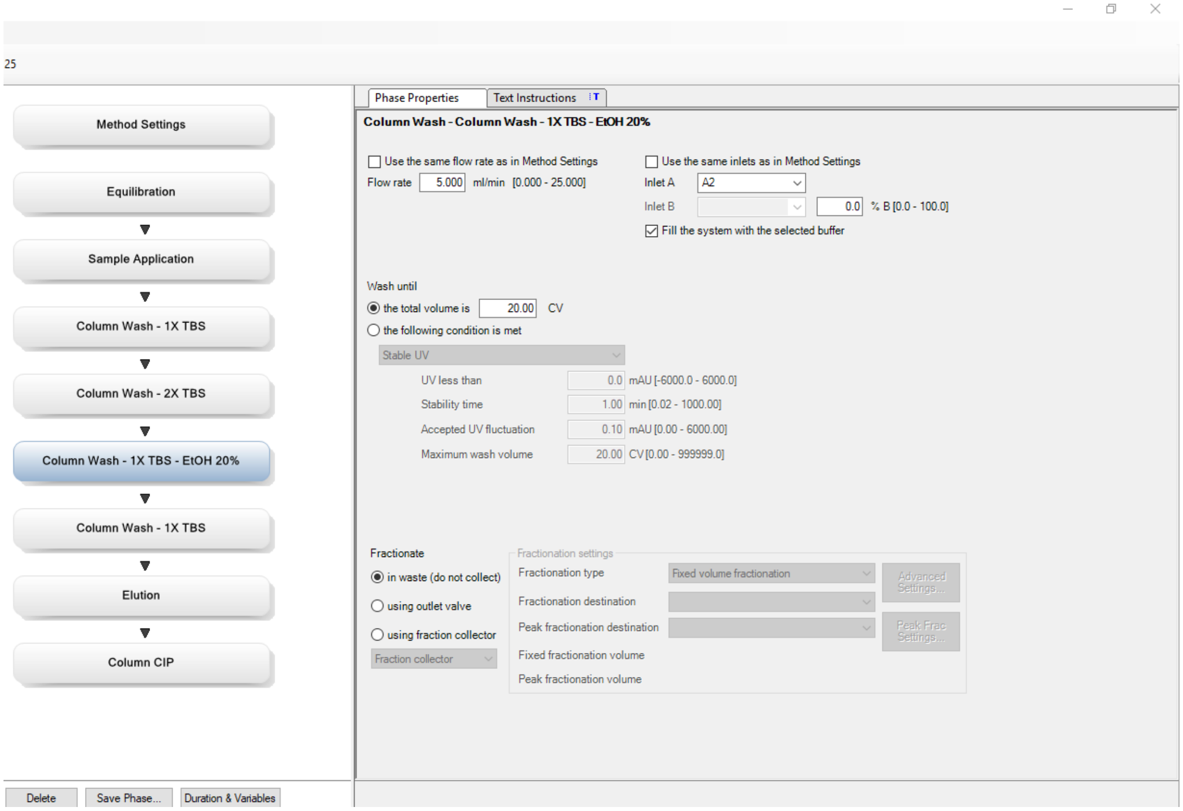
25
Method Settings
Equilibration
Sample Application
Column Wash - 1X TBS
Column Wash - 2X TBS
Column Wash - 1X TBS - EtOH 20%
Column Wash - 1X TBS
Elution
Column CIP
Delete
Save Phase...
Duration & Variables
Phase Properties
Text Instructions
Column Wash - Column Wash - 1X TBS - EtOH 20%
Use the same flow rate as in Method Settings
Flow rate 5.000 ml/min [0.000 - 25.000]
Use the same inlets as in Method Settings
Inlet A A2
Inlet B 0.0 % B [0.0 - 100.0]
Fill the system with the selected buffer
Wash until
the total volume is 20.00 CV
the following condition is met
Stable UV
UV less than 0.0 mAU [-6000.0 - 6000.0]
Stability time 1.00 min [0.02 - 1000.00]
Accepted UV fluctuation 0.10 mAU [0.00 - 6000.00]
Maximum wash volume 20.00 CV [0.00 - 999999.0]
Fractionate
in waste (do not collect)
using outlet valve
using fraction collector
Fraction collector
Fractionation settings
Fractionation type
Fixed volume fractionation
Fractionation destination
Peak fractionation destination
Fixed fractionation volume
Peak fractionation volume
Advanced Settings...
Peak Frac Settings...

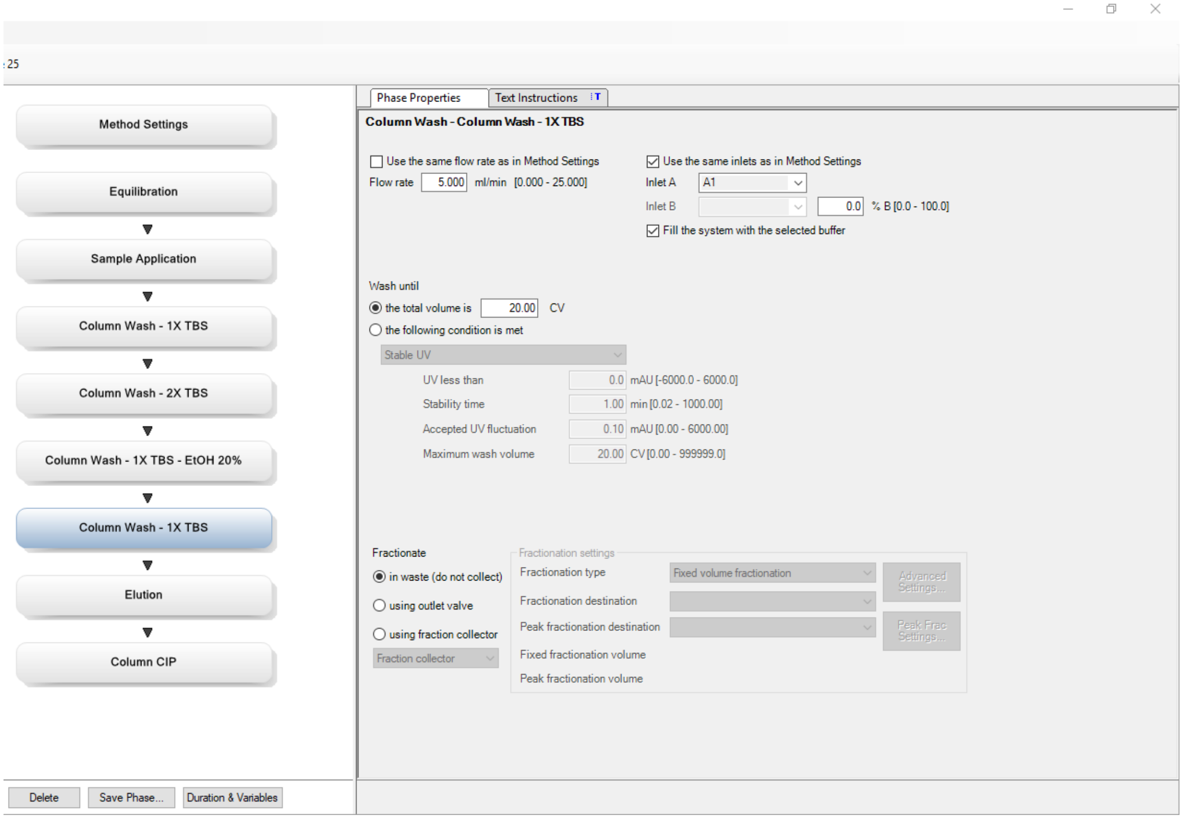
25
Method Settings
Equilibration
Sample Application
Column Wash - 1X TBS
Column Wash - 2X TBS
Column Wash - 1X TBS - EtOH 20%
Column Wash - 1X TBS
Elution
Column CIP
Delete
Save Phase...
Duration & Variables
Phase Properties
Text Instructions
Column Wash - Column Wash - 1X TBS
Use the same flow rate as in Method Settings
Flow rate 5.000 ml/min [0.000 - 25.000]
Use the same inlets as in Method Settings
Inlet A A1
Inlet B 0.0 % B [0.0 - 100.0]
Fill the system with the selected buffer
Wash until
the total volume is 20.00 CV
the following condition is met
Stable UV
UV less than 0.0 mAU [-6000.0 - 6000.0]
Stability time 1.00 min [0.02 - 1000.00]
Accepted UV fluctuation 0.10 mAU [0.00 - 6000.00]
Maximum wash volume 20.00 CV [0.00 - 999999.0]
Fractionate
in waste (do not collect)
using outlet valve
using fraction collector
Fraction collector
Fractionation settings
Fractionation type
Fixed volume fractionation
Fractionation destination
Peak fractionation destination
Fixed fractionation volume
Peak fractionation volume
Advanced Settings...
Peak Frac Settings...

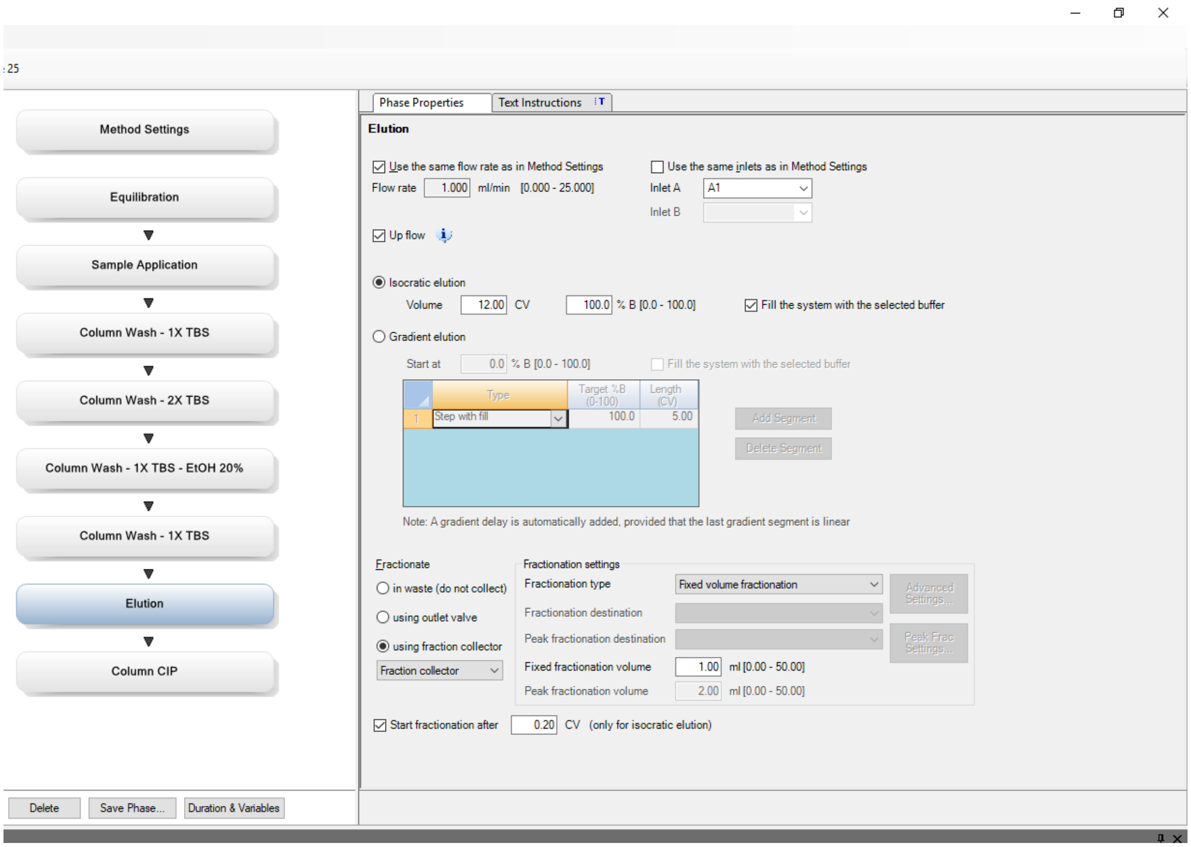
:25
Method Settings
Equilibration
Sample Application
Column Wash - 1X TBS
Column Wash - 2X TBS
Column Wash - 1X TBS - EtOH 20%
Column Wash - 1X TBS
Elution
Column CIP
Delete
Save Phase...
Duration & Variables
Phase Properties
Text Instructions
Elution
Use the same flow rate as in Method Settings
Flow rate 1.000 ml/min [0.000 - 25.000]
Use the same inlets as in Method Settings
Inlet A A1
Inlet B
Up flow
Isocratic elution
Volume 12.00 CV 100.0 % B [0.0 - 100.0]
Fill the system with the selected buffer
Gradient elution
Start at 0.0 % B [0.0 - 100.0]
Fill the system with the selected buffer
Type
Target %B (0-100)
Length (CV)
Step with fill 100.0 5.00
Add Segment
Delete Segment
Note: A gradient delay is automatically added, provided that the last gradient segment is linear
Fractionate
in waste (do not collect)
using outlet valve
using fraction collector
Fraction collector
Fractionation settings
Fractionation type Fixed volume fractionation
Fractionation destination
Peak fractionation destination
Fixed fractionation volume 1.00 ml [0.00 - 50.00]
Peak fractionation volume 2.00 ml [0.00 - 50.00]
Advanced Settings...
Peak Frac Settings...
Start fractionation after 0.20 CV (only for isocratic elution)

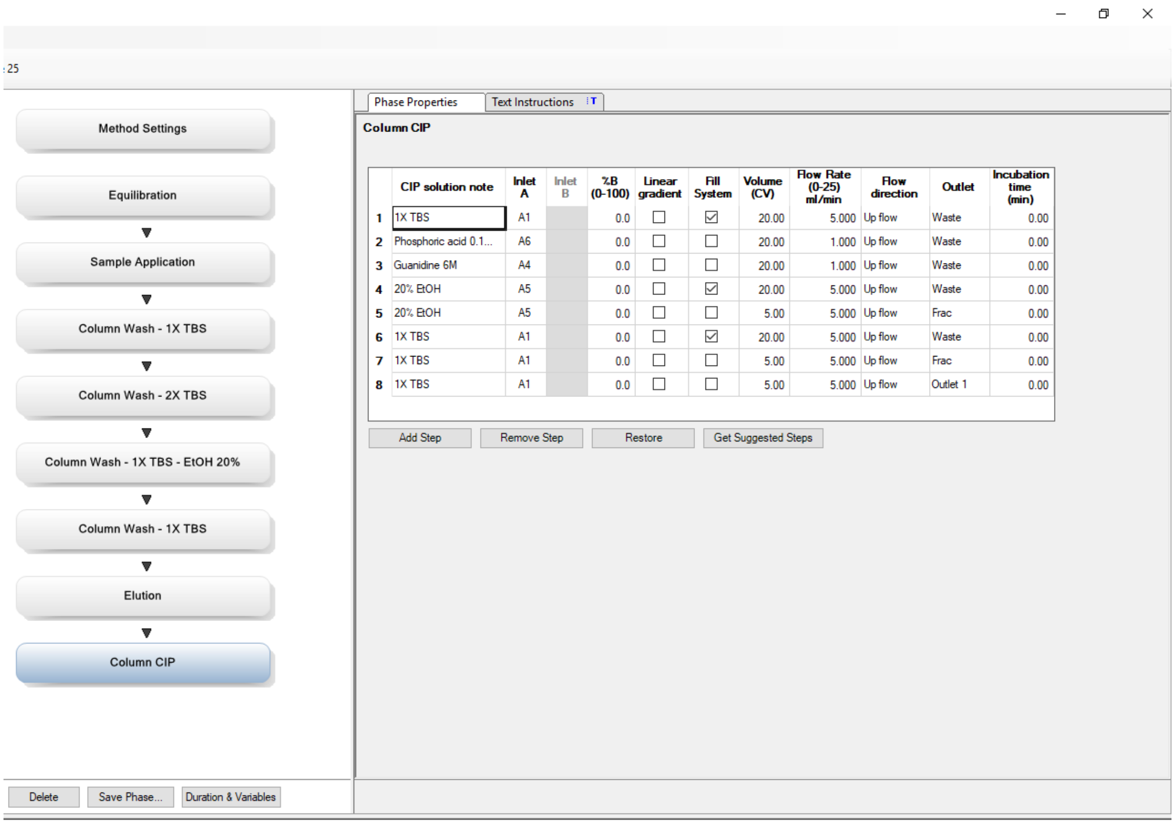
:25
Method Settings
Equilibration
Sample Application
Column Wash - 1X TBS
Column Wash - 2X TBS
Column Wash - 1X TBS - EtOH 20%
Column Wash - 1X TBS
Elution
Column CIP
Delete
Save Phase...
Duration & Variables
Phase Properties
Text Instructions
Column CIP
CIP solution note | Inlet A | Inlet B | %B (0-100) | Linear gradient | Fill System | Volume (CV) | Flow Rate (0-25) ml/min | Flow direction | Outlet | Incubation time (min)
1 1X TBS A1 0.0 20.00 5.000 Up flow Waste 0.00
2 Phosphoric acid 0.1... A6 0.0 20.00 1.000 Up flow Waste 0.00
3 Guanidine 6M A4 0.0 20.00 1.000 Up flow Waste 0.00
4 20% EtOH A5 0.0 20.00 5.000 Up flow Waste 0.00
5 20% EtOH A5 0.0 5.00 5.000 Up flow Frac 0.00
6 1X TBS A1 0.0 20.00 5.000 Up flow Waste 0.00
7 1X TBS A1 0.0 5.00 5.000 Up flow Frac 0.00
8 1X TBS A1 0.0 5.00 5.000 Up flow Outlet 1 0.00
Add Step
Remove Step
Restore
Get Suggested Steps

System CIP

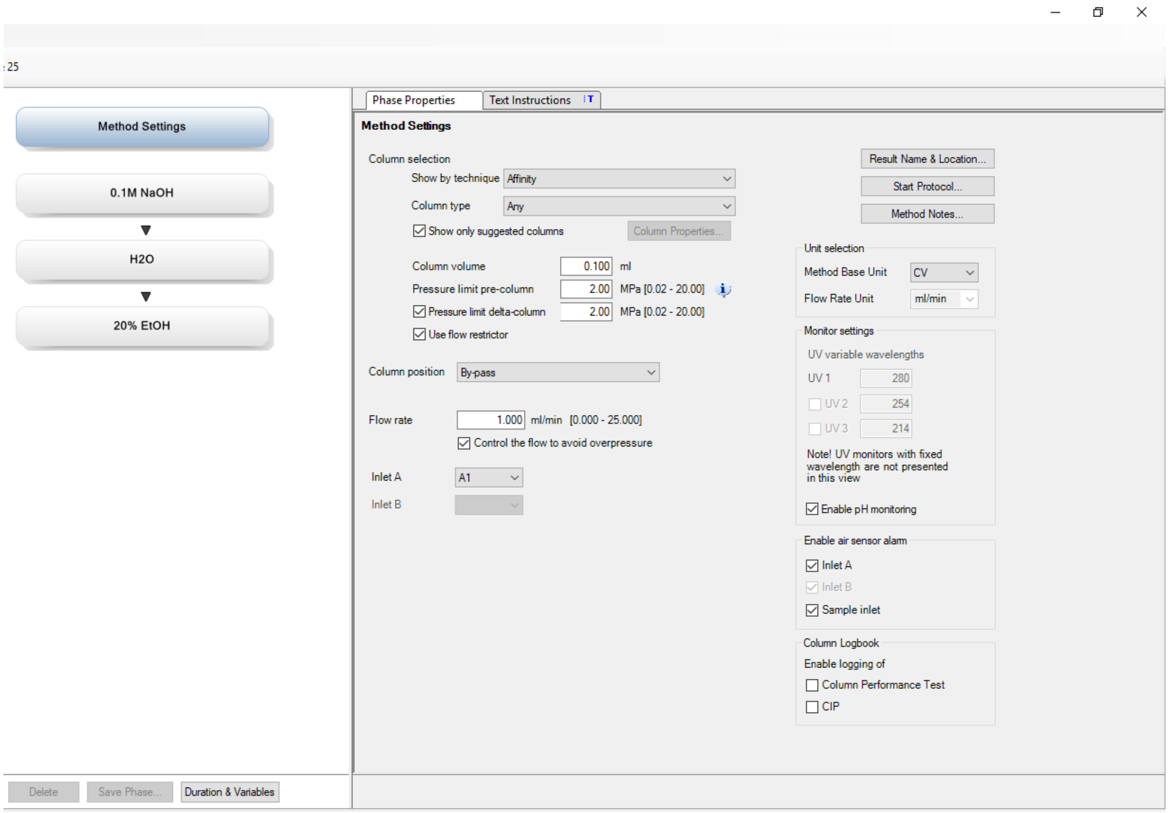

Method Settings
0.1M NaOH
H2O
20% EtOH
Delete
Save Phase...
Duration & Variables
Phase Properties
Text Instructions
Method Settings
Column selection
Show by technique
Affinity
Column type
Any
Show only suggested columns
Column Properties...
Column volume
0.100
ml
Pressure limit pre-column
2.00
MPa [0.02 - 20.00]
Pressure limit delta-column
2.00
MPa [0.02 - 20.00]
Use flow restrictor
Column position
By-pass
Flow rate
1.000
ml/min [0.000 - 25.000]
Control the flow to avoid overpressure
Inlet A
A1
Inlet B
Result Name & Location...
Start Protocol...
Method Notes...
Unit selection
Method Base Unit
CV
Flow Rate Unit
ml/min
Monitor settings
UV variable wavelengths
UV 1
280
UV 2
254
UV 3
214
Note! UV monitors with fixed wavelength are not presented in this view
Enable pH monitoring
Enable air sensor alarm
Inlet A
Inlet B
Sample inlet
Column Logbook
Enable logging of
Column Performance Test
CIP

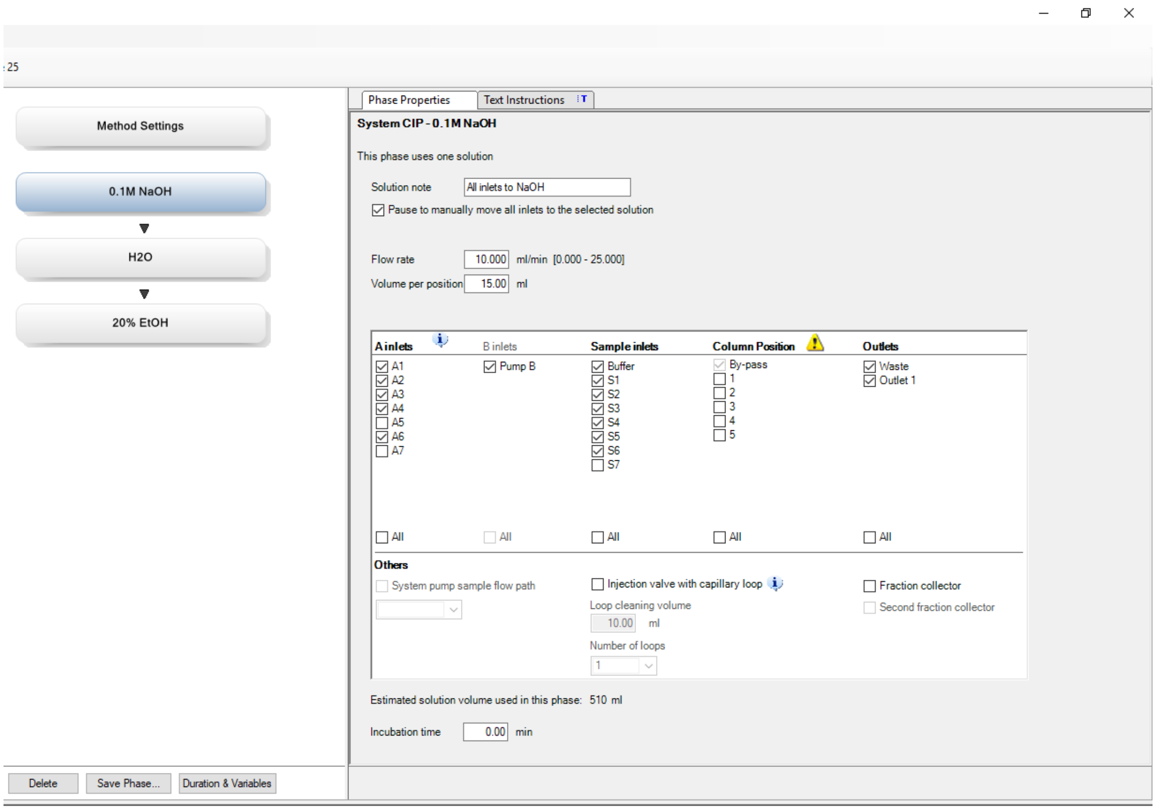

:25
Method Settings
0.1M NaOH
H2O
20% EtOH
Phase Properties
Text Instructions
System CIP - 0.1M NaOH
This phase uses one solution
Solution note
All inlets to NaOH
Pause to manually move all inlets to the selected solution
Flow rate 10.000 ml/min [0.000 - 25.000]
Volume per position 15.00 ml
A inlets
B inlets
Sample inlets
Column Position
Outlets
A1 A2 A3 A4 A5 A6 A7
Pump B
Buffer S1 S2 S3 S4 S5 S6 S7
By-pass 1 2 3 4 5
Waste Outlet 1
All
Others
System pump sample flow path
Injection valve with capillary loop
Loop cleaning volume 10.00 ml
Number of loops 1
Fraction collector
Second fraction collector
Estimated solution volume used in this phase: 510 ml
Incubation time 0.00 min
Delete
Save Phase...
Duration & Variables

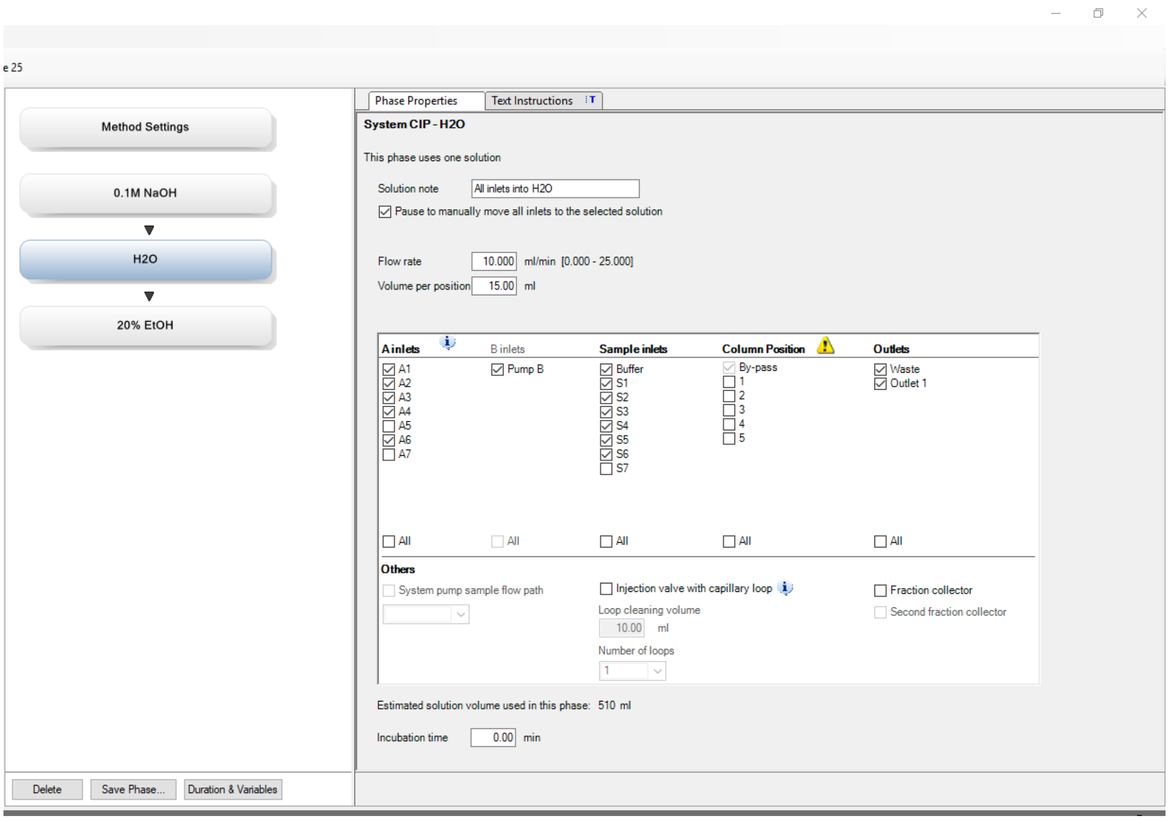
e 25
Method Settings
0.1M NaOH
H2O
20% EtOH
Delete
Save Phase...
Duration & Variables
Phase Properties
Text Instructions
System CIP - H2O
This phase uses one solution
Solution note
All inlets into H2O
Pause to manually move all inlets to the selected solution
Flow rate
10.000
ml/min [0.000 - 25.000]
Volume per position
15.00
ml
A inlets
A1
A2
A3
A4
A5
A6
A7
B inlets
Pump B
Sample inlets
Buffer
S1
S2
S3
S4
S5
S6
S7
Column Position
By-pass
1
2
3
4
5
Outlets
Waste
Outlet 1
All
Others
System pump sample flow path
Injection valve with capillary loop
Loop cleaning volume
10.00
ml
Number of loops
1
Fraction collector
Second fraction collector
Estimated solution volume used in this phase: 510 ml
Incubation time
0.00
min

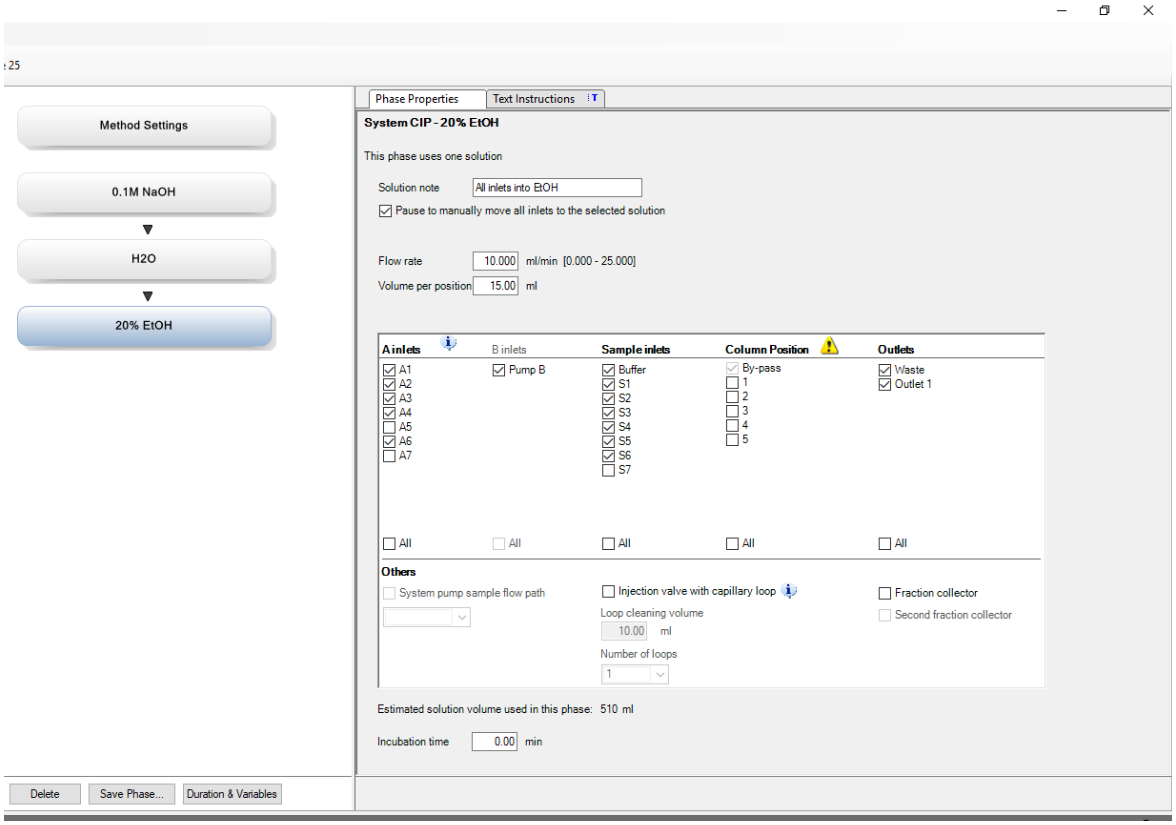
25
Method Settings
0.1M NaOH
H2O
20% EtOH
Delete
Save Phase...
Duration & Variables
Phase Properties
Text Instructions
System CIP - 20% EtOH
This phase uses one solution
Solution note
All inlets into EtOH
Pause to manually move all inlets to the selected solution
Flow rate
10.000
ml/min [0.000 - 25.000]
Volume per position
15.00
ml
A inlets
A1
A2
A3
A4
A5
A6
A7
B inlets
Pump B
Sample inlets
Buffer
S1
S2
S3
S4
S5
S6
S7
Column Position
By-pass
1
2
3
4
5
Outlets
Waste
Outlet 1
All
Others
System pump sample flow path
Injection valve with capillary loop
Loop cleaning volume
10.00
ml
Number of loops
1
Fraction collector
Second fraction collector
Estimated solution volume used in this phase: 510 ml
Incubation time
0.00
min
